# Cyano- and Ketone-Containing Selenoesters as Multi-Target Compounds against Resistant Cancers

**DOI:** 10.3390/cancers13184563

**Published:** 2021-09-11

**Authors:** Nikoletta Szemerédi, Simona Dobiasová, Noemi Salardón-Jiménez, Annamária Kincses, Márta Nové, Giyaullah Habibullah, Clotilde Sevilla-Hernández, Miguel Benito-Lama, Francisco-Javier Alonso-Martínez, Jitka Viktorová, Gabriella Spengler, Enrique Domínguez-Álvarez

**Affiliations:** 1 Department of Medical Microbiology, Albert Szent-Györgyi Health Center and Faculty of Medicine, University of Szeged, Semmelweis utca 6, 6725 Szeged, Hungary; szemeredi.nikoletta@med.u-szeged.hu (N.S.); kincses.annamaria90@gmail.com (A.K.); nove.marta@med.u-szeged.hu (M.N.); 2 Department of Biochemistry and Microbiology, Faculty of Food and Biochemical Technology, University of Chemistry and Technology Prague, Technická 3, 166 28 Prague 6, Czech Republic; dobiasoo@vscht.cz (S.D.); habibuli@vscht.cz (G.H.); 3 Instituto de Química Orgánica General (IQOG-CSIC), Consejo Superior de Investigaciones Científicas, Juan de la Cierva 3, 28006 Madrid, Spain; noemi.sj.95@gmail.com (N.S.-J.); clo.sh.1995@gmail.com (C.S.-H.); miguelbenitodelama@gmail.com (M.B.-L.); franalonso9112@gmail.com (F.-J.A.-M.)

**Keywords:** multidrug resistance, efflux pump, ABCB1, apoptosis, selenium, cancer

## Abstract

**Simple Summary:**

The search for novel anticancer agents has been the hot topic of interest in cancer research, due to the phenomenon of multidrug resistance (MDR) in cancer that can make cancer cells resistant to the current available chemotherapeutic agents. In this context, we have designed, synthesized, and biologically evaluated 15 novel selenoesters, with the aim to explore their activity against resistant cancer cell lines. Some of these described selenocompounds showed noteworthy cytotoxicity and selectivity, the ability to inhibit the ABCB1 efflux pump, the capacity to modulate the ATPase activity of this pump, the capability to trigger apoptotic events, the ability to interact in a synergistic manner with doxorubicin in resistant cancer cells, and the power to promote wound healing. Consequently, these results validate the design of these selenocompounds and justify further research to evaluate the possibilities of these compounds to be used in the future in the fight against resistant cancers.

**Abstract:**

Fifteen selenocompounds, comprising of eight ketone-containing selenoesters (**K1**–**K8**, also known as oxoselenoesters) and seven cyano-containing selenoesters (**N1**–**N7**, known also as cyanoselenoesters), have been designed, synthesized, and evaluated as novel anticancer agents. These compounds are derivatives of previously reported active selenoesters and were prepared following a three-step one-pot synthetic route. The following evaluations were performed in their biological assessment: cytotoxicity determination, selectivity towards cancer cells in respect to non-cancer cells, checkerboard combination assay, ABCB1 inhibition and inhibition of ABCB1 ATPase activity, apoptosis induction, and wound healing assay. As key results, all the compounds showed cytotoxicity against cancer cells at low micromolar concentrations, with cyanoselenoesters being strongly selective. All of the oxoselenoesters, except **K4**, were potent ABCB1 inhibitors, and two of them, namely **K5** and **K6**, enhanced the activity of doxorubicin in a synergistic manner. The majority of these ketone derivatives modulated the ATPase activity, showed wound healing activity, and induced apoptosis, with **K3** being the most potent, with a potency close to that of the reference compound. To summarize, these novel derivatives have promising multi-target activity, and are worthy to be studied more in-depth in future works to gain a greater understanding of their potential applications against cancer.

## 1. Introduction

The occurrence of multidrug resistance (MDR) to chemotherapeutic drugs has become a significant challenge in cancer therapy. One of the most important factors contributing to MDR is the overexpression of efflux pumps. P-glycoprotein (P-gp)—also known as multidrug resistance protein 1 (MDR1), ATP-binding cassette sub-family B member 1 (ABCB1), or the cluster of differentiation 243 (CD243)—was discovered in 1970 as a member of the ATP-binding cassette (ABC) transporter family [1,2]. These ABC transporters fulfil physiological functions in the gastrointestinal tract, liver, and lungs. They are localized in different barriers that separate blood vessels from specific organs, such as the blood–brain barrier (BBB), the blood–cerebrospinal fluid (B-CSF), the blood–retina barrier (BRB), the blood–testis barrier (BTB), and in the placenta [3]. In humans, this transporter is encoded by the MDR1, also known as *ABCB1* gene [4]. ABCB1 substrates are typically amphiphilic compounds. ABCB1 is comprised of two nucleotide-binding domains and 12 transmembrane domains which constitute a drug-binding pocket. This transporter is a natural cell protective protein whose function is the removal of xenobiotic compound out of the cells, as this compound can be toxic for the cells [5,6]. ABCB1 can remove various chemotherapeutic agents, e.g., daunorubicin, doxorubicin, vinblastine, vincristine, epirubicin, etoposide, imatinib, irinotecan, paclitaxel, or colchicine, thus leading to treatment failure in anticancer therapy [7]. It has been reported that ABCB1, together with the multidrug resistance-associated protein 1 (MRP1)/ATP Binding Cassette Subfamily C Member 1 (ABCC1) (encoded by *ABCC1* gene), is the major determinant of innate drug sensitivity, even at the lowest level of expression [8,9]. The design of inhibitors of efflux pumps, especially in regard to ABCB1, is a promising strategy in cancer therapy [10].

Selenium, and the organic and inorganic compounds that contain this element, are essential in various biological processes. It is known that selenium deficiency can cause disorders or augment risk of developing cancers [11]. Alternatively, epidemiological studies reported that dietary supplementation with selenium can reduce the incidence of certain types of cancers. These starting works in selenium supplementation led to the reporting of a wide variety of organic and inorganic selenocompounds with chemopreventive, antiproliferative, and cytotoxic activity against cancer [12]. Sodium selenite is probably the most deeply studied inorganic selenium salt with anticancer activity. Among organic selenocompounds with chemopreventive and anticancer activity, methylselenol, methylseleninic acid, selenocyanates, and diphenyl diselenide can be cited [13]. Finally, the number of works that study the anticancer and multidrug resistance reversing activities of selenium nanoparticles is significantly increasing nowadays [14].

Considering these lines of evidence, we have designed, synthesized, and determined the biological activity of selenium-containing anticancer agents, mostly of them selenoesters. It has been demonstrated that a selenoanhydride derivative and some selenoester derivatives have potent anticancer activity against ABCB1 expressing in MDR mouse T-lymphoma cells and MDR colon adenocarcinoma cells due to ABCB1 inhibition and apoptosis induction [15]. In addition, these derivatives exerted potent anticancer activity on sensitive and resistant breast cancer cell lines [16]. It has been confirmed that selenium compounds synergistically enhance the activity of anticancer drugs when they are administered in combination [17]. Furthermore, as a drug-repurposing approach, phenothiazines with a known pharmacological and toxicity profile were combined with the previously mentioned selenoanhydride and selenoester, and several Se-compounds exhibited synergistic activity in combination with promethazine, chlorpromazine, and thioridazine [18]. The synergistic effects observed suggest that selenium compounds are able to reverse MDR and potentiate the activity of reference anticancer drugs or compounds with well-defined anticancer activity.

**Figure 1:**
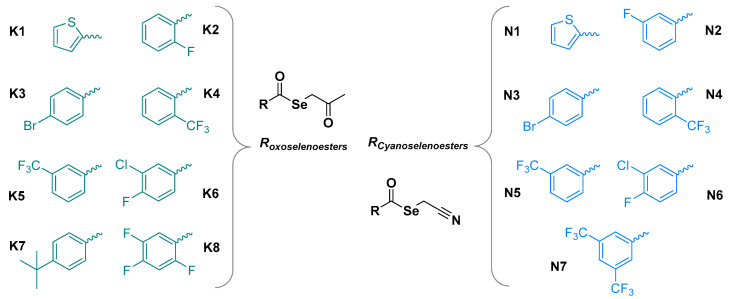
Structure of the oxoselenoesters, **K1–K8,** and of the cyanoselenoesters, **N1–N7,** presented in this work.

Based on these results, 15 newly synthesized selenoesters, shown in Figure 1, have been investigated in this study with regard to their anticancer and MDR reversing capacity in sensitive and resistant colon adenocarcinoma cell lines. Out of them, eight contain a ketone in the alkyl group directly bound to the selenium atom (oxoselenoesters or ketone-selenoesters, **K1**–**K8**); and seven contain a cyano group in the same alkyl moiety (cyanoselenoesters, **N1**–**N7**).

## 2. Materials and Methods

### 2.1. Chemical Reagents and Chemical Characterization

The chemical reagents, solvents, and materials used to synthesize the cyanoselenoesters and the oxoselenoesters presented herein were acquired at different vendors: Acros Organics and Alfa Aesar (both brands of Thermo Fisher Scientific, Geel, Belgium); Fluorochem (Hadfield, Derbyshire, UK); Honeywell Riedel de Haën (Seelze, Germany); Panreac Química S.L.U (Castellar del Vallés, Barcelona, Spain); Scharlab S.L. Spain (Sentmenat, Barcelona, Spain); and Sigma-Aldrich Merck S.L.U. Spain (Madrid, Spain).

To characterize the compounds, preliminary NMR spectra (Nuclear Magnetic Resonance) were taken using a Varian Inova-300 spectrometer (Agilent Technologies, Santa Clara, CA, USA) to monitor the reactions. The purity of the compounds, with a clean spectrum at the 300 MHz spectrometer, was assessed by means of elemental analysis using a LECO CHNS-932 microanalyser (LECO Europe B.V., Geleen, The Netherlands), at a temperature of 990 °C, using He as a carrier gas, and using silver capsules to introduce the sample in the analyser. Each compound must show a deviation of less than 0.40% in each element analysed (C, H, N, S) to be considered pure. The spectra included in the Supplementary Materials (Figures S1A–S15F) were taken with the following instruments: (i) NMR-^1^H, NMR-^13^C, and bidimensional COSY (Correlation spectroscopy); HSQC (Heteronuclear Single-Quantum Correlation spectroscopy); HMBC (Heteronuclear Multiple-Bond Correlation spectroscopy); and a Bruker Avance III HD-400 (Billerica, MA, USA) spectrometer using TMS as the internal standard. Next, (ii) mass spectra with a direct insertion probe (MS-DIP); and a quadrupole HP 5973 MSD spectrometer (Hewlett Packard, now Agilent Technologies, Santa Clara, CA, USA) with a direct insertion probe and electronic impact (EI) in positive mode as the ionization source, at a 70 eV ionization energy and with an *m*/*z* precision of ±0.05. Finally, (iii) Infrared spectroscopy (IR), using a Spectrum One B (Perkin-Elmer, Waltham, MA, USA) spectrophotometer. Solid samples were assayed preparing KBr films, whereas liquid samples were assayed between NaCl crystals. Finally, melting points have been determined in a Reichert-Kofler heating system coupled with a microscope; they are provided as obtained by visual inspection, without correction.

### 2.2. Synthetic Procedure

A synthetic procedure with three consecutive reactions in the same pot has been followed to obtain these 2-oxopropyl selenoesters and cyanomethyl selenoesters. In a first step, an equivalent of selenium grey powder is suspended in 20 mL of water, and 2 equivalents of sodium borohydride are added slowly over it and left stirring, usually for 15 min, or until the end of the gas release. Subsequently, an equivalent of the corresponding acyl chloride is added, and the reaction is stirred for 90 min at 50–70 °C. Then, the crude reaction mixture is filtered to eliminate the formed boron salts, and an equivalent of the appropriate alkyl halide are added at that time over the filtrate. The mixture is then kept reacting until reaction completion, which normally requires 1 h at 50 °C and an additional hour, initially at room temperature, and later ice-cooled.

The desired selenoester is isolated and purified by application of the most adequate techniques in each case, namely, as precipitation if the compound is solid, and extraction plus column chromatography if it is liquid. The purification followed for each compound is described in Section 2.3.

Starting acyl chlorides, if not commercially available at a reasonable cost, can be synthesized through the chlorination of the corresponding carboxylic acid with thionyl chloride. In this case, the appropriate derivative of benzoic acid is solved in an excess of thionyl chloride (at least 5:1 in molar ratio) and kept refluxing while stirring for 5 h. Then, thionyl chloride is removed in the rotary evaporator and the crude is washed 3 times with 50 mL of toluene, removing the toluene each time in the rotary evaporator to take away the remaining amounts of thionyl chloride. The structure of the synthesized acyl chloride is verified by ^1^H-NMR, as compared with published data (data not shown). Then, the reagent is used in the synthesis of the respective selenocompound without further purification.

### 2.3. Chemical Description of the Compounds

#### 2.3.1. *Se-(2-Oxopropyl) Thiophene-2-carboselenoate* (**K1**)

The reagents used: sodium borohydride (0.119 g, 3.15 mmol), grey selenium (0.120 g, 1.52 mmol), thiophene-2-carbonyl chloride (0.219 g, 0.16 mL, 1.5 mmol), and chloroacetone (0.139 g, 0.12 mL, 1.5 mmol). The final compound precipitated as a yellow solid powder that was isolated by filtration and washed with water, rendering 181 mg (**49%**). **MW:** 247.17. **Mp**: 52–53 °C. **DIP-MS** *m*/*z* (abundance %): 57.05 (3); 83.05 (7); 111.05 (100); and 245.95/2.47.95 (1/2, Se, M^+^). **IR** (KBr) (cm^−1^): 3082 (m, C-H_Ar_); 2958, 2922 (m, C-H); 1704 (m, C=O ketone); 1661 (s, C=O selenoester); and 1645, 1514 (m, C-C_Ar_). **^1^H-NMR** (400 MHz, CDCl_3_); δ: 7.83 (dd, J_3-4_= 3.9 Hz, J_3-5_= 1.0 Hz, 1H, H_3_); 7.72 (dd, *J_4-5_*= 5.0 Hz, 1H, H_5_); 7.16 (dd, 1H, H_4_); 3.90 (s, 2H, SeCH_2_); and 2.33 (s, 3H, COCH_3_). **^13^C-NMR** (101 MHz, CDCl_3_, δ: 203.6 (COCH_3_); 183.0 (COSe); 142.7 (C_2_); 134.2 (C_5_); 132.5 (C_3_); 128.3 (C_4_); 34.5 (SeCH_2_); and 28.7 (COCH_3_). **Elemental analysis** for C_8_H_8_O_2_SSe, calculated/found (%): C: 38.88/39.18; H: 3.26/3.41; and S:12.97/12.93.

#### 2.3.2. *Se-(2-Oxopropyl) 2-Fluorobenzoselenoate* (**K2**)

The reagents used: sodium borohydride (0.122 g, 3.22 mmol), grey selenium (0.123 g, 1.55 mmol), 2-fluorobenzoyl chloride (0.239 g, 0.18 mL, 1.51 mmol), and chloroacetone (0.139 g, 0.12 mL, 1.50 mmol). The final compound was obtained as a pale-yellow liquid, rendering 59 mg (**15%**). **MW:** 259.14. **Mp**: Liquid RT. **DIP-MS** *m*/*z* (abundance %): 75.05 (16); 95.05 (38); 123.15 (100); 161.05 (8); and 255.95/256.95/257.95/259.95/261.95 (0/0/1/2/0, Se, M^+·^). **IR** (KBr) (cm^−1^): 3067, 3005 (s, C-H_Ar_); 2925 (m, C-H); 1707 (m, C=O ketone); 1656 (s, C=O selenoester); and 1609, 1577, 1482, 1452 (m, C-C_Ar_). **^1^H-NMR** (400 MHz, CDCl_3_), δ: 7.86 (td, *J_6-5_* = 7.6 Hz, *J_6-4_* = 1.8 Hz, 1H, H_6_); 7.58 (dddd, *J_4-3_*= 8.3 Hz, *J_4-5_* = 7.5 Hz, *J_4-F_* = 5.0 Hz, *J_4-6_* = 1.7 Hz, 1H, H_4_); 7.26 (td, 1H, H_5_), 7.19 (ddd, *J_3-F_* = 11.0 Hz, 1H, H_3_); 3.90 (s, 2H, SeCH_2_); and 2.35 (s, 3H, COCH_3_). **^13^C-NMR** (101 MHz, CDCl_3_, δ: 203.7 (COCH_3_); 188.4 (d, *J_COSe-F_* = 5.2 Hz, COSe); 160.9 (d, *J_C(F)-F_* = 258.7 Hz, C_2(F)_); 135.4 (d, *J_6-F_* = 9.2 Hz, C_6_); 129.5 (d, *J_5-F_* = 1.4 Hz, C_5_); 126.0 (d, *J_1-F_* = 10.4 Hz, C_1_); 124.8 (d, *J_4-F_* = 3.6 Hz, C_4_); 117.2 (d, *J_3-F_* = 22.2 Hz, C_3_); 34.8 (d, *J_SeCH2-F_* = 6.5 Hz, SeCH_2_); and 28.9 (COCH_3_). **Elemental analysis** for C_10_H_9_FO_2_Se, calculated/found (%): C: 46.35/46,54; and H: 3.50/3.55.

#### 2.3.3. *Se-(2-Oxopropyl) 4-Bromobenzoselenoate* (**K3**)

The reagents used: sodium borohydride (0.200 g, 5.29 mmol), grey selenium (0.200 g, 2.53 mmol), 4-bromobenzoyl chloride (0.551 g, 2.51 mmol), and chloroacetone (0.230 g, 0.20 mL 2.51 mmol). The final compound precipitated as a white-pale-pink solid that was isolated by filtration, washed with water, and dried, rendering 331 mg (**41%**). **MW:** 320.04. **Mp**: 55–57 °C. **DIP-MS** *m*/*z* (abundance %): 50.15 (10); 75.05 (16); 76.05 (18); 154.95/156.95 (31/30, Br); 182.95/184.95 (100/97, Br); and 317.90/319.90/31.85 (0/1/1, Br + Se, M^+^−2/M^+^/M^+·^+ 2). **IR** (KBr) (cm^−1^): 2954, 2917 (w, C-H_Alk_); 1706 (m, C=O ketone); 1665 (s, C=O selenoester); and 1582, 1564, 1481 (m, C-C_Ar_). **^1^H-NMR** (400 MHz, CDCl_3_); δ: 7.76 (td, *J_2-3,6-5_* = 8.7 Hz, *J_2-Br,6-Br_=* 2.3 Hz, 2H, H_2_+H_6_); 7.62 (td, *J_3-Br,5-Br_*= 2.3 Hz, 2H, H_3_+H_5_); 3.91 (s, 2H, SeCH_2_); and 2.34 (s, 3H, COCH_3_). **^13^C-NMR** (101 MHz, CDCl_3_, δ: 203.7 (COCH_3_); 192.3 (COSe); 137.2 (C_4Ph(Br)_); 132.8 (C_3Ph_+C_5Ph_); 129.8 (C_1Ph_); 129.2 (C_2Ph_+C_6Ph_); 35.1 (SeCH_2_); and 29.2 (COCH_3_). **Elemental analysis** for C_10_H_9_BrO_2_Se, calculated/found (%): C: 37.53/37.53; and H: 2.83/2.78.

#### 2.3.4. *Se-(2-Oxopropyl) 2-(Trifluoromethyl)benzoselenoate* (**K4**)

The reagents used: sodium borohydride (0.197 g, 5.21 mmol), grey selenium (0.195 g, 2.47 mmol), 2-(trifluoromethyl)benzoyl chloride (0.523 g, 0.37 mL, 2.51 mmol), and chloroacetone (0.239 g, 0.20 mL, 2.5 mmol). The final compound was extracted with dichloromethane (4 × 30 mL), dried, and evaporated in vacuum. A column using dichloromethane–toluene (3:1) was performed to purify the compound, rendering 158 mg of a pale-yellow liquid (**21%**). **MW:** 247.17. **Mp**: Liquid RT. **DIP-MS** *m*/*z* (abundance %): 75.05 (5); 95.05 (8); 125.05 (6); 126.05 (3); 145.05 (59); 173.15 (100); and 307.95/309.95 (0/0, Se, M^+·^). **IR** (KBr) (cm^−1^): 3074, 3006 (m, C-H_Ar_); 2925 (m, C-H_Alk_); 1697 (s, broad, overlapping bands of selenoester and ketone C=O); and 1601, 1582, 1448 (w, C-C_Ar_). **^1^H-NMR** (400 MHz, CDCl_3_); δ: 7.76–7.79 (m, 2H, H_3Ph_+H_6Ph_); 7.63–7.69 (m, 2H, H_4Ph_+H_5Ph_); 3.93 (s, 2H, SeCH_2_); and 2.35 (s, 3H, COCH_3_). **^13^C-NMR** (101 MHz, CDCl_3_, δ: 203.3 (COCH_3_); 193.3 (COSe); 138.6 (q, *J_1-CF3_* = 1.8 Hz, C_1Ph_); 132.1 (d, *J_4-CF3_* = 0.7 Hz, C_4Ph_); 132.0 (C_5Ph_), 128.4 (C_6Ph_); 127.4 (q, *J_3-CF3_ =* 5.3 Hz, C_3Ph_);126.4 (q, *J_6-CF3_*= 33.0 Hz, C_2Ph_); 121.8 (q, *J_C-F[CF3]_* = 274.5 Hz, CF_3_); 36.0 (SeCH_2_); and 28.6 (COCH_3_). **Elemental analysis** for C_9_H_11_F_3_O_2_Se, calculated/found (%): C: 42.74/42.42; and H: 2.93/3.09.

#### 2.3.5. *Se-(2-Oxopropyl) 3-(Trifluoromethyl)benzoselenoate* (**K5**)

The reagents used: sodium borohydride (0.119 g, 3.15 mmol), grey selenium (0.120 g, 1.52 mmol), 3-(trifluoromethyl)benzoyl chloride (0.313 g, 0.23 mL, 1.53 mmol), and chloroacetone (0.139 g, 0.12 mL, 1.50 mmol). The final compound was extracted with dichloromethane (4 × 30 mL), dried, and evaporated in vacuum. A column using dichloromethane–toluene (3:1) was performed to purify the compound, rendering 133 mg of a pale-yellow liquid (**29%**). **MW:** 238.15. **Mp**: Liquid RT. **DIP-MS** *m*/*z* (abundance %): 75.05 (6); 95.05 (9); 125.05 (7); 126.05 (5); 145.05 (74); 173.15 (100); and 305.95/306.95/307.95/309.95/311.95 (0/0/1/2/0, Se, M^+·^). **IR** (KBr) (cm^−1^): 3073, 3007 (m, C-H_Ar_); 2925 (m, C-H_Alk_); 1712 (s, C=O ketone); 1679 (s, C=O selenoester); and 1611, 1590, 1484 (m, C-C_Ar_). **^1^H-NMR** (400 MHz, CDCl_3_); δ: 8.14 (s, 1H, H_2Ph_); 8.08 (d, *J_6-5_*= 7.9 Hz, 1H, H_6Ph_); 7.88 (d, *J_4-5_* = 7.8 Hz, 1H, H_4Ph_); 7.64 (t, 1H, H_5Ph_); 3.96 (s, 2H, SeCH_2_); and 2.36 (s, 3H, COCH_3_). **^13^C-NMR** (101 MHz, CDCl_3_, δ: 203.1 (COCH_3_); 192.1 (COSe); 138.8 (C_1Ph_); 131.9 (q, *J_3-CF3_* = 33.3 Hz, C_3Ph_); 130.7 (d, *J_6-CF3_* = 1.0 Hz, C_6Ph_); 130.6 (q, *J_4-CF3_* = 3.5 Hz, C_4Ph_); 129.9 (C_5Ph_); 124.2 (q, *J_2-CF3_* = 3.9 Hz, C_2Ph_); 123.5 (q, *J_C-F[CF3]_* = 272.5 Hz, CF_3_); 35.0 (SeCH_2_); and 28.9 (COCH_3_). **Elemental analysis** for C_9_H_6_ClNOSe, calculated/found (%): C: 42.74/42.43; and H: 2.93/3.05. The acyl chloride (3-fluorobenzoyl chloride) was synthesized from 3-fluorobenzoic acid (15.859 g, 113.19 mmol) and thionyl chloride (50 mL, excess), obtaining 11.675 g of the acyl chloride (65% yield). Complete yield of the full synthetic route is then 19%.

#### 2.3.6. *Se-(2-Oxopropyl) 3-Chloro-4-fluorobenzoselenoate* (**K6**)

The reagents used: sodium borohydride (0.159 g, 4.20 mmol), grey selenium (0.161 g, 2.04 mmol), 3-chloro-4-fluorobenzoyl chloride (0.389 g, 2.02 mmol), and chloroacetone (0.186 g, 0.16 mL, 2.01 mmol). The final compound precipitated as a white solid that was isolated by filtration, washed with water, and purified in the column chromatography using dichloromethane as the eluent; obtaining, after column, 106 mg of a white solid powder (**18%**). **MW:** 238.15. **Mp**: 42–44 °C. **DIP-MS** *m*/*z* (abundance %): 74.05 (3); 93.05 (5); 94.05 (7); 109.05/110.95 (5/2, Cl); 129.05/130.95 (9/5, Cl); 157.05/158.95 (100/33, Cl); and 289.95/290.95/291.95/293.95/295.95 (0/0/1/1/0, Se, M^+·^). **IR** (KBr) (cm^−1^): 3097, 3050 (m, C-H_Ar_); 2978, 2898 (m, C-H); 1717 (m, C=O ketone); 1672 (s, C=O selenoester); and 1591, 1494 (m, C-C_Ar_). **^1^H-NMR** (400 MHz, CDCl_3_); δ: 7.97 (dd, *J_2-F_* = 6.9 Hz, *J_2-6_ =* 2.2 Hz, 1H, H_2Ph_); 7.82 (ddd, *J_6-5_* = 8.6 Hz, *J_6-F_* = 4.4 Hz, 1H, H_6Ph_); 7.25 (t, 1H, H_5Ph_); 3.93 (s, 2H, SeCH_2_); and 2.34 (s, 3H, COCH_3_). **^13^C-NMR** (101 MHz, CDCl_3_, δ: 203.1 (COCH_3_); 190.6 (COSe); 161.7 (d, *J_C4(F)-F_* = 258.7 Hz, C_4Ph(F)_); 135.3 (d, *J_1-F_* = 3.7 Hz, C_1Ph_); 130.1 (d, *J_2-F_* = 1.1 Hz, C_2Ph_); 127.9 (d, *J_6-F_* = 8.6 Hz, C_6Ph_); 122.6 (d, *J_3-F_* = 18.5 Hz, C_3Ph(Cl)_); 117.4 (d, *J_5-F_* = 22.1 Hz, C_5Ph_); 35.1 (SeCH_2_); and 28.9 (COCH_3_). **Elemental analysis** for C_10_H_8_ClFO_2_Se, calculated/found (%): C: 40.91/40.65; and H: 2.75/2.83. The acyl chloride (3-chloro-4-fluorobenzoyl chloride) was synthesized from 3-chloro-4-fluorobenzoic acid (9.426 g, 50 mmol) and thionyl chloride (35 mL, excess), obtaining 9.602 g of the acyl chloride (99.5% yield). Complete yield of the full synthetic route is then 18%.

#### 2.3.7. *Se-(2-Oxopropyl) 4-(Tert-butyl)benzoselenoate* (**K7**)

The reagents used: sodium borohydride (0.119 g, 3.15 mmol), grey selenium (0.119 g, 1.51 mmol), 4-*tert-*butylbenzoyl chloride (0.292 g, 1.47 mmol), and chloroacetone (0.139 g, 0.12 mL, 1.50 mmol). The final compound was extracted with dichloromethane (4 × 30 mL), dried, and evaporated in vacuum. A column using dichloromethane–hexane (4:1) was performed to purify the compound, rendering 30 mg of a pale-yellow liquid (**7%**). **MW:** 297.26. **Mp**: Liquid RT. **DIP-MS** *m*/*z* (abundance %): 65.05 (1); 77.05 (4); 91.05 (9); 105.05 (5); 118.05 (15); 133.15 (2); 146.05 (17); 161.15 (100); and 298.05 (0, Se, M^+·^). **IR** (KBr) (cm^−1^): 2964, 2907, 2870 (s, C-H); 1710 (s, C=O ketone); 1678 (s, C=O selenoester); and 1601, 1567, 1465 (m, C-C_Ar_). **^1^H-NMR** (400 MHz, CDCl_3_); δ: 7.85 (d*, J_2-3,6-5_* = 8.6 Hz, 2H, H_2_+H_6_); 7.49 (d, 2H, H_3_+H_5_); 3.89 (s, 2H, SeCH_2_); 2.33 (s, 3H, COCH_3_); and 1.34 (s, 9H, C_4_H_9_). **^13^C-NMR** (101 MHz, CDCl_3_, δ: 204.1 (COCH_3_); 192.2 (COSe); 158.4 (C_1_); 135.5 (C_4_); 127.5 (C_2_+C_6_); 126.1 (C_3_+C_5_); 35.5 (**C**(CH_3_)_3_); 34.2 (SeCH_2_); 31.2 (C(**C**H_3_)_3_); and 28.6 (s, COCH_3_). **Elemental analysis** for C_14_H_18_O_2_Se, calculated/found (%): C: 56.57/56.52; and H: 6.10/5.97.

#### 2.3.8. *Se-(2-Oxopropyl) 2,4,5-Trifluorobenzoselenoate* (**K8**)

The reagents used: sodium borohydride (0.199 g, 5.26 mmol), grey selenium (0.200 g, 2.53 mmol), 2,4,5-trifluorobenzoyl chloride (0.483 g, 2.48 mmol), and chloroacetone (0.244 g, 0.21 mL, 2.63 mmol). The final compound precipitated as a yellow solid powder that was isolated by filtration and washed with water, rendering 306 mg (**42%**). **MW:** 295.12. **Mp**: 40–43 °C. **DIP-MS** *m*/*z* (abundance %): 81.05 (10); 92.95(1); 112.05 (1); 131.05 (20); 159.05 (100); and 291.95/292.95/293.95/295.95/297.95 (0/0/1/1/0, Se, M^+·^. **IR** (KBr) (cm^−1^): 3058 (s, C-H_Ar_); 2963, 2921 (m, C-H); 1708 (s, C=O ketone); 1650 (s, C=O selenoester); and 1623, 1511 (s, C-C_Ar_). **^1^H-NMR** (400 MHz, CDCl_3_); δ: 7.71 (ddd, *J_6-F5_* = 10.1 Hz, *J_6-F4_=* 8.6 Hz, J_6-F2_= 6.4, 1H, H_6_); 7.07 (td, *J_3-F4,3-F2_* = 9.8 Hz, *J_3-F5_*= 6.1 Hz, 1H, H_3_); 3.92 (s, 2H, SeCH_2_); and 2.35 (s, 3H, COCH_3_). **^13^C-NMR** (101 MHz, CDCl_3_, δ: 203.1 (s, COCH_3_); 186.5 (s, COSe); 157.2 (ddd, *J_2-F2_* = 257.6 Hz, *J_2-F4_* = 10.1 Hz, *J_2-F5_* = 2.5 Hz, C_2(F)_); 153.6 (ddd, *J_4-F4_* = 261.5 Hz, *J_4-F5_* = 14.7 Hz, *J_4-F2_* = 12.5 Hz, C_4(F)_); 147.1 (ddd, *J_5-F5_* = 249.5 Hz, *J_5-F4_* = 12.9 Hz, *J_5-F2_* = 3.3 Hz, C_2(F)_); 122.4 (ddd, *J_1-F2_* = 13.1 Hz, *J_1-F5_* = 4.3 Hz, *J_1-F4_* = 3.8 Hz, C_1(F)_); 117.3 (dt, *J_6-F5_* = 20.5 Hz, *J_6_-_F2,6-F4_* = 3.1 Hz, C_6_); 107.3 (dd, *J_3-F4_* = 28.6 Hz, *J_3_-_F6_* = 21.3 Hz, C_3_); 35.1 (SeCH_2_); and 29.1 (COCH_3_). **Elemental analysis** for C_10_H_7_F_3_O_2_Se, calculated/found (%): C: 40.70/40.61; and H: 2.39/2.45. The acyl chloride (2,4,5-trifluorobenzoyl chloride) was synthesized from 2,4,5-trifluorobenzoic acid (8.805 g, 50 mmol) and thionyl chloride (35 mL, excess), obtaining 9.538 g of the acyl chloride (98% yield). Complete yield of the full synthetic route is then 41%.

#### 2.3.9. *Se-(Cyanomethyl) Thiophene-2-carboselenoate* (**N1**)

The reagents used: sodium borohydride (0.197 g, 5.21 mmol), grey selenium (0.194 g, 2.46 mmol), thienyl chloride (0.370 g, 0.27 mL, 2.54 mmol), and chloroacetonitrile (0.191 g, 0.16 mL, 2.53 mmol). The final compound precipitated as a beige solid that was isolated by filtration and washed with water, rendering 85 mg (**15%** yield). **MW:** 230.14. **Mp**: 50–52 °C. **DIP-MS** *m*/*z* (abundance %): 57.05 (9); 83.05 (21); 111.15 (100); and 230.95 (0, M^+·^). IR (KBr, cm^−1^): 3111, 3082, 3068 (w, C-H_Ar_); 2990, 2935 (m, C-H_Alk_); 2246 (m, C≡N); 1662 (s, C=O); and 1512, 1406 (m, C-C_Thiophene_). **^1^H-NMR** (400 MHz, CDCl_3_); δ: 7.81 (dd, *J_2-3_* = 3.9 Hz, *J_2-4_* = 1.1 Hz, 1H, H_2Tp_); 7.81 (dd, *J_2-4_* = 5.0 Hz, 1H, H_4Tp_); 7.19 (dd, 1H, H_3Tp_); and 3.71 (s, 2H, SeCH_2_). **^13^C-NMR** (101 MHz, CDCl_3_, δ: 180.4 (CO); 141.5 (C_1Tp_); 135.2 (C_4Tp_); 133.0 (C_2Tp_); 128.5 (C_3Tp_); 117.2 (C≡N); and 5.5 (SeCH_2_). **Elemental analysis** for C_7_H_5_NOSSe, calculated/found (%): C: 36.53/36.69; H: 2.19/2.32; N: 6.09/6.22; and S: 13.93/13.89.

#### 2.3.10. *Se-(Cyanomethyl) 3-Fluorobenzoselenoate* (**N2**)

The reagents used: sodium borohydride (0.199 g, 5.26 mmol), grey selenium (0.198 g, 2.51 mmol), 3-fluorobenzoyl chloride (0.391 g, 0.30 mL, 2.47 mmol), and chloroacetonitrile (0.190 g, 0.16 mL, 2.53 mmol). The final compound was extracted with dichloromethane (4 × 30 mL), dried, and evaporated in vacuum. A column using dichloromethane–hexane (4:1) was performed to purify the compound, rendering 138 mg (**23%**) of a pale-yellow liquid. **MW:** 242.11. **Mp**: Liquid at room temperature. **DIP-MS** *m*/*z* (abundance %): 50.15 (6); 69.05 (6); 75.15 (35); 95.15 (93); 123.15 (100); and 242.95 (0, M^+^). **IR** (NaCl, cm^−1^): 3075 (w, C-H_Ar_); 2998, 2944 (m, C-H_Alk_); 2246 (m, C≡N); 1690 (s, C=O); and 1589, 1482, 1437 (m, C-C_Ar_). **^1^H-NMR** (400 MHz, CDCl_3_); δ: 7.66 (td, *J_6-5_* = 7.8 Hz, *J_6-2_* = 1.0 Hz, 1H, H_6Ph_); 7.55 (dt, *J_2-F_* = 9.1 Hz, *J_2-4_* = 2.3 Hz, 1H, H_2Ph_); 7.50 (td, *J_5-4,5-6_* = 8.2 Hz, *J_5-F_* = 5.6 Hz, 1H, H_5Ph_); 7.37 (tdd, 1H, H_4Ph_); and 3.71 (s, 2H, SeCH_2_). **^13^C-NMR** (101 MHz, CDCl_3_, δ: 189.5 (CO); 163.0 (d, *J_3-F_* = 250.4 Hz, C_3Ph_); 139.1 (d, *J_2-F_* = 6.6 Hz, C_1Ph_); 131.1 (d, *J_5-F_* = 7.8 Hz, C_5Ph_); 123.5 (d, *J_6-F_* = 3.1 Hz, C_6Ph_); 121.9 (d, *J_4-F_* = 21.5 Hz, C_4Ph_); 117.1 (C≡N), 114.2 (d, *J_2-F_* = 23.3 Hz, C_2Ph_); and 5.7 (SeCH_2_). **Elemental analysis** for C_9_H_6_FNOSe, calculated/found (%): C: 44.65/44.42; H: 2.50/2.62; and N: 5.79/6.83. The acyl chloride (3-fluorobenzoyl chloride) was synthesized from 3-fluorobenzoic acid (15.859 g, 113.19 mmol) and thionyl chloride (50 mL, 82 g, excess), obtaining 11.675 g of the acyl chloride (65% yield). Complete yield of the full synthetic route is then 15%.

#### 2.3.11. *Se-(Cyanomethyl) 4-Bromobenzoselenoate* (**N3**)

The reagents used: sodium borohydride (0.200 g, 5.29 mmol), grey selenium (0.199 g, 2.52 mmol), 4-bromobenzoyl chloride (0.551 g, 2.51 mmol), and chloroacetonitrile (0.191 g, 0.16 mL, 2.53 mmol). The final compound precipitated as a white solid that was isolated by filtration and washed with water, rendering 340 mg (**45%**). **MW:** 303.02. **Mp**: 105–107 °C. **DIP-MS** *m*/*z* (abundance %): 50.10 (14); 75.00 (23); 76.00 (23); 154.90/156.90 (37/37, Br); and 182.90/184.90 (100/98, Br). **IR** (KBr, cm^−1^): 3082, 3007 (m, C-H_Ar_); 2241 (m, C≡N); 1667 (s, C=O); and 1586, 1565, 1394 (m, C-C_Ar_). **^1^H-NMR** (400 MHz, CDCl_3_); δ: 7.73 (td, *J_2-3,6-5_* = 8.7 Hz, *J_2-Br,6-Br_=* 2.0 Hz, 2H, H_2_+H_6_); 7.66 (td, *J_3-Br,5-Br_*= 2.1 Hz, 2H, H_3_+H_5_); and 3.70 (s, 2H, SeCH_2_). **^13^C-NMR** (101 MHz, CDCl_3_, δ: 189.6 (COSe); 136.0 (C_4Ph(Br)_); 131.7 (C_3Ph_+C_5Ph_); 130.2 (C_1Ph_); 128.9 (C_2Ph_+C_6Ph_); 117.1 (C≡N); and 5.6 (SeCH_2_). **Elemental analysis** for C_9_H_6_BrNOSe, calculated/found (%): C: 35.67/35.37; H: 2.00/2.03; and N: 4.62/4.72.

#### 2.3.12. *Se-(Cyanomethyl) 2-(Trifluoromethyl)benzoselenoate* (**N4**)

The reagents used: m sodium borohydride (0.197 g, 5.21 mmol), grey selenium (0.197 g, 2.49 mmol), 2-(trifluoromethyl)benzoyl chloride (0.524 g, 2.51 mmol), and chloroacetonitrile (0.191 g, 0.16 mL, 2.53 mmol). The final compound precipitated as a white powder that was isolated by filtration and washed with water, rendering 294 mg (**40%**). **MW:** 292.12. **Mp**: 65–67 °C. **DIP-MS** *m*/*z* (abundance %): 50.15 (4); 75.05 (11); 95.05 (15); 125.05 (11); 126.05 (5); 145.05 (88); and 173.15 (100). **IR** (KBr, cm^−1^): 2992, 2936 (w, C-H_Alk_); 2239 (s, C≡N); 1706 (s, C=O); and 1584, 1390 (m, C-C_Ar_). **^1^H-NMR** (400 MHz, CDCl_3_); δ: 7.81 (dd, *J_6-5_* = 5.4 Hz, *J_6-4_* = 3.7 Hz, 1H, H_6Ph_); 7.76 (dd, *J_3-4_* = 5.5 Hz, *J_3-5_* = 3.8 Hz, 1H, H_3Ph_); 7.71 (t, 1H, H_4Ph_); 7.69 (t, 1H, H_5Ph_); and 3.72 (s, 2H, SeCH_2_). **^13^C-NMR** (101 MHz, CDCl_3_, δ: 191.2 (COSe); 137.3 (C_1Ph_); 132.6 (C_5Ph_); 132.4 (d, *J_4-CF3_* = 0.7 Hz, C_4Ph_); 128.4 (C_6Ph_); 127.6 (q, *J_5-CF3_ =* 5.3 Hz, C_3Ph_); 126.8 (q, *J_6-CF3_*= 33.1 Hz, C_2Ph_); 123.0 (q, *J_C-F[CF3]_* = 274.1 Hz, CF_3_); 116.8 (C≡N); and 7.0 (SeCH_2_). **Elemental analysis** for C_10_H_6_F_3_NOSe, calculated/found (%): C: 41.12/41.08; H: 2.07/2.15; and N: 4.79/4.75.

#### 2.3.13. *Se-(Cyanomethyl) 3-(Trifluoromethyl)benzoselenoate* (**N5**)

The reagents used: sodium borohydride (0.198 g, 5.23 mmol), grey selenium (0.198 g, 2.51 mmol), 3-(trifluoromethyl)benzoyl chloride (0.526 g, 2.52 mmol), and chloroacetonitrile (0.191 g, 0.16 mL, 2.53 mmol). The final compound was a liquid non-miscible with water that was separated by decantation. Crude liquid was dissolved in 100 mL of dichloromethane and treated with silica, activated charcoal, and anhydrous sodium sulfate. After filtration and removal of the solvent in a rotary, 157 mg (**21%**) of a pale-yellow liquid was obtained. **MW:** 292.12. **Mp**: Liquid at room temperature. **DIP-MS** *m*/*z* (abundance %): 50.15 (5); 75.05 (10); 95.05 (13); 125.05 (9); 126.05 (5); 145.05 (85); 173.15 (100); and 292.95 (0, M^+·^). **IR** (KBr, cm^−1^): 3074, 3000 (s, C-H_Ar_); 2946 (m, C-H_Alk_); 2247 (s, C≡N); 1683 (s, C=O); and 1612, 1440 (s, C-C_Ar_). **^1^H-NMR** (400 MHz, CDCl_3_); δ: 8.11 (s, 1H, H_2Ph_); 8.05 (d, *J_6-5_*= 7.9 Hz, 1H, H_6Ph_); 7.93 (d, *J_4-5_* = 7.8 Hz, 1H, H_4Ph_); 7.68 (t, 1H, H_5Ph_); and 3.74 (s, 2H, SeCH_2_). **^13^C-NMR** (101 MHz, CDCl_3_, δ: 189.7 (COSe); 137.9 (C_1Ph_); 132.2 (q, *J_3-CF3_* = 33.5 Hz, C_3Ph_); 131.2 (q, *J_4-CF3_* = 3.5 Hz, C_4Ph_); 130.8 (d, *J_6-CF3_* = 1.0 Hz, C_6Ph_); 130.2 (C_5Ph_); 124.3 (q, *J_2-CF3_* = 3.8 Hz, C_2Ph_); 123.4 (q, *J_C-F[CF3]_* = 272.8 Hz, CF_3_); 116.9 (CN); and 5.9 (SeCH_2_). **Elemental analysis** for C_10_H_6_F_3_NOSe, calculated/found (%): C: 41.12/41.13; H: 2.07/2.12; and N: 4.79/4.83.

#### 2.3.14. *Se-(Cyanomethyl) 3-Chloro-4-fluorobenzoselenoate* (**N6**)

The reagents used: sodium borohydride (0.200 g, 5.29 mmol), grey selenium (0.197 g, 2.49 mmol), 3-chloro-4-fluorobenzoyl chloride (0.484 g, 2.51 mmol), and chloroacetonitrile (0.191 g, 0.16 mL, 2.53 mmol). The final compound precipitated as a white powder that was isolated by filtration and washed with water, rendering 279 mg (**41%**). **MW:** 276.55. **Mp**: 73–75 °C. **DIP-MS** *m*/*z* (abundance %): 74.00 (4); 93.00 (8); 94.00 (9); 109.00/110.95 (7/2, Cl); 129.00/130.95 (38/12, Cl); and 156.90/158.90 (100/33, Cl). **IR** (KBr, cm^−1^): 3101, 3051, 3010 (s, C-H_Ar_); 2953 (m, C-H_Alk_); 2242 (s, C≡N); 1679 (s, C=O); and 1587, 1495, 1398 (s, C-C_Ar_). **^1^H-NMR** (400 MHz, CDCl_3_); δ: 7.94 (dd, *J_2-F_* = 6.8 Hz, *J_2-6_ =* 2.2 Hz, 1H, H_2Ph_); 7.79 (ddd, *J_6-5_* = 8.5 Hz, *J_6-F_* = 4.3 Hz, 1H, H_6Ph_); 7.29 (t, 1H, H_5Ph_); and 3.71 (s, 2H, SeCH_2_). **^13^C-NMR** (101 MHz, CDCl_3_, δ: 188.2 (COSe); 162.1 (d, *J_C4(F)-F_* = 259.9 Hz, C_4Ph(F)_); 134.3 (d, *J_1-F_* = 3.7 Hz, C_1Ph_); 130.3 (d, *J_2-F_* = 1.3 Hz, C_2Ph_); 128.1 (d, *J_6-F_* = 8.7 Hz, C_6Ph_); 123.0 (d, *J_3-F_* = 18.6 Hz, C_3Ph(Cl)_); 117.7 (d, *J_5-F_* = 22.2 Hz, C_5Ph_); 116.9 (CN); and 5.9 (SeCH_2_). **Elemental analysis** for C_9_H_5_ClFNOSe, calculated/found (%): C: 39.09/39.12; H: 1.82/1.89; and N: 5.06/5.03. The acyl chloride (3-chloro-4-fluorobenzoyl chloride) was synthesized from 3-chloro-4-fluorobenzoic acid (9.426 g, 50 mmol) and thionyl chloride (35 mL, excess), obtaining 9.602 g of the acyl chloride (99.5% yield). Complete yield of the full synthetic route is then 40%.

#### 2.3.15. *Se-(Cyanomethyl) 3,5-Bis(trifluoromethyl)benzoselenoate* (**N7**)

The reagents used: sodium borohydride (0.197 g, 5.22 mmol), grey selenium (0.197 g, 2.49 mmol), 3,5-bis(trifluoromethyl)benzoyl chloride (0.6874 g, 2.48 mmol), and chloroacetonitrile (0.191 g, 0.16 mL, 2.53 mmol). The final compound precipitated as a white solid powder that was isolated by filtration and washed with water, rendering 209 mg (23**%**). **MW:** 360.12. **Mp**: 63–65 °C. **DIP-MS** *m*/*z* (abundance %): 75.00 (3); 125.00 (2); 144.00 (5); 192.90 (2); 193.95 (4); 213.00 (45); 221.95 (2); and 241.00 (100). **IR** (KBr, cm^−1^): 3098, 3029, 3004 (m, C-H_Ar_); 2948 (s, C-H_Alk_); 2246 (m, C≡N); 1687 (s, C=O); and 1616, 1462 (m, C-C_Ar_). **^1^H-NMR** (400 MHz, CDCl_3_); δ: 8.28 (s, 2H, H_2Ph_+H_6Ph_); 8.17 (s, 1H, H_4Ph_); and 3.79 (s, 2H, SeCH_2_). **^13^C-NMR** (101 MHz, CDCl_3_; δ: 188.9 (CO); 139.0 (C_1Ph_); 133.3 (q, *J_3-CF3(3),5-CF3(5)_* = 34.5 Hz, C_3Ph_+C_5Ph_); 127.9 (p, *J_4-CF3(3),4-CF3(5)_* = 3.5 Hz, C_4Ph_); 127.4 (q, *J_2-CF3(3),6-CF3(5)_* = 2.9 Hz, C_2Ph_+C_6Ph_); 122.6 (q, *J_C(CF3)-F_* = 273.3 Hz, C_CF3(3)_+C_CF3(5)_); 116.4 (CN); and 6.4 (SeCH_2_). **Elemental analysis** for C_11_H_5_F_6_NOSe, calculated/found (%): C: 36.69/36.61; H: 1.40/1.43; and N: 3.89/3.89. The acyl chloride (3,5-bis(trifluoromethyl)benzoyl chloride) was obtained from 3,5-bis(trifluoromethyl)benzoic acid (12.906 g, 50 mmol) and thionyl chloride (35 mL, excess), obtaining 7.422 g of the acyl chloride (54% yield). Complete yield of the full synthetic route is then 13%.

### 2.4. Reagents, Medias, and Chemicals Used in the Biological Evaluation

Analytical grade (to enable its use without further purification) rhodamine 123 (R123); sodium dodecyl sulfate (SDS); 3-(4.5-dimethylthiazol-2-yl)-2.5-diphenyltetrazolium bromide (MTT); Dulbecco’s Modified Eagle’s medium–high glucose (DMEM) with 10% fetal bovine serum (FBS) and 1% penicillin-streptomycin mixture; resazurin sodium salt; trypsin-EDTA solution; allantoin; and tariquidar and dimethyl sulfoxide (DMSO) were acquired at Sigma-Aldrich (St. Louis, MO, USA). Doxorubicin hydrochloride was acquired from Teva Pharmaceuticals (Petah Tikva, Israel). Eagle’s Minimal Essential Medium (EMEM, Sigma-Aldrich) containing 4500 mg/L glucose, supplemented with a non-essential amino acid (NEAA) mixture (Sigma-Aldrich); a selection of vitamins and 10% heat-inactivated FBS; 2 mM L-glutamine (Sigma-Aldrich); 1 mM Na-pyruvate (Sigma-Aldrich); nystatin (Sigma-Aldrich); a penicillin-streptomycin mixture at concentrations of 100 U/L and 10 mg/L; RPMI 1640 medium (Sigma-Aldrich), supplemented with 10% FBS; 2 mM L-glutamine; 1 mM Na-pyruvate; 100 mM HEPES (Sigma-Aldrich); nystatin; and a penicillin-streptomycin mixture at concentrations of 100 U/L and 10 mg/L were used in the biological evaluation. Pgp-Glo™ Assay Systems (Promega), and an Annexin V-FITC Apoptosis Detection Kit were used (Calbiochem, EMD Biosciences. Inc. La Jolla, CA, USA).

### 2.5. Preparations of Compounds for Biological Assays

The fifteen selenoesters (Figure 1) evaluated in this work, whose synthesis and characterization have been described above, were dissolved in dimethyl sulfoxide (DMSO) to obtain stock solutions with a 10 mM concentration to perform their biological evaluation.

### 2.6. Cell Lines and Their Maintenance

Three cell lines have been used in this study: the doxorubicin-sensitive Colo 205 (CCL-222, ATCC, Manassas, VA, USA) human colonic adenocarcinoma cell line; the multidrug resistant Colo 320/MDR-LRP expressing P-gp (MDR1)-LRP (CCL-220.1, ATCC) human colonic adenocarcinoma cell line; and the MRC-5 human embryonal lung fibroblast cell line (CCL-171, ATCC). The colon adenocarcinoma cell lines were purchased from LGC Promochem (Teddington, UK), and the MRC-5 cell line was purchased from Sigma-Aldrich (Merck KGaA, Darmstadt, Germany). Their culture conditions are as follows: Colo 205 (CCL-222, ATCC) and Colo 320/MDR-LRP expressing P-gp (MDR1)-LRP (CCL-220.1, ATCC) human colon adenocarcinoma cell lines were cultured in RPMI 1640 medium, supplemented with 10% FBS, 2 mM L-glutamine, 1 mM Na-pyruvate, and 100 mM HEPES. The cell lines were incubated at 37 °C, 5% CO_2_, and 95% air atmosphere. The semi-adherent human colon cancer cells were detached with a Trypsin-Versene (EDTA) solution for 5 min at 37 °C. MRC-5 human embryonal lung fibroblast cells were cultured in EMEM containing 4.5 g/L of glucose and supplemented with a non-essential amino acid mixture, a selection of vitamins, and 10% FBS. The cell lines were incubated at 37 °C, 5% CO_2_, and 95% air atmosphere.

The activity of selenoesters was tested on several cancer cell lines, including HepG2 (hepatocellular carcinoma, CCL-23TM, ATCC); HeLa (cervical adenocarcinoma, CCL-2TM, ATCC); B16 (skin melanoma, CCL-6322TM, ATCC); and a non-cancerous cell line, HDF (human dermal fibroblasts, Sigma-Aldrich). HepG2, HeLa, B16, and HDF cell lines were cultivated in EMEM medium supplemented with 10% FBS, 2 mM L-glutamine, and 1% penicillin-streptomycin mixture. The HaCaT cell line was cultivated in DMEM medium supplemented with 10% FBS and 1% penicillin-streptomycin mixture. All of the cells were cultivated in a CO_2_ incubator (5% CO_2_, 37 °C). Twice per week, the cell lines were passaged according to a standardized protocol with a trypsin-EDTA solution.

Wound healing activity was realized using human keratinocyte (HaCaT, Thermo Fisher Scientific, Waltham, MA, USA).

### 2.7. Cytotoxicity

The effects of increasing concentrations of the compounds on cell growth were tested in 96-well flat-bottomed microtiter plates. Moreover, 10^4^ of human colonic adenocarcinoma cells in 100 μL of the medium (RPMI-1640) were added to each well, except for the medium control wells. The adherent human embryonic lung fibroblast cell line was seeded in the EMEM medium for 4 h before the assay. The two-fold serial dilutions of the compounds were made in a separate plate (0.19–100 μM), and then transferred to the plates containing the adherent corresponding cell line. Culture plates were incubated at 37 °C for 24 h. At the end of the incubation period, 20 μL of MTT (thiazolyl blue tetrazolium bromide) solution (from a 5 mg/mL stock solution) were added to each well. After incubation at 37 °C for 4 h, 100 μL of sodium dodecyl sulfate (SDS) solution (10% SDS in 0.01 M HCl) was added to each well, and the plates were further incubated at 37 °C overnight. Cell growth was determined by measuring the optical density (OD) at 540 nm (ref. 630 nm) with a Multiscan EX ELISA reader (Thermo Labsystems, Cheshire, WA, USA). Inhibition of cell growth was expressed as IC_50_ values, defined as the inhibitory dose that reduces the growth of the cells exposed to the tested compounds by 50%. IC_50_ values and the SD of triplicate experiments were calculated by using GraphPad Prism software version 5.00 for Windows, with a non-linear regression curve fit (GraphPad Software, San Diego, CA, USA; www.graphpad.com, accessed on 12 July 2021). Doxorubicin (from a 2 mg/mL stock solution, Teva Pharmaceuticals) was used as a positive control. The solvent (DMSO) did not have any effect on the cell growth in the tested concentrations.

The selectivity indexes (SI) were calculated as the ratio of the IC_50_ value in the non-tumour cells and the IC_50_ in the cancer cell lines. The compound’s activity towards cancer cells is considered as strongly selective if the selectivity index (SI) value is higher than 6, moderately selective if 3 < SI < 6, slightly selective if 1 < SI < 3, and non-selective if SI is lower than 1 [16].

### 2.8. Checkerboard Combination Assay

A checkerboard microplate method was applied to study the effect of drug interactions between the selenocompounds and the chemotherapeutic drug doxorubicin on resistant Colo 320 colon adenocarcinoma cells expressing the ABCB1 transporter. Results were expressed in terms of the combination index (CI) values at 50% growth inhibition (ED_50_), which were determined by using CompuSyn software to plot 4 or 5 data points for each ratio. CI values were calculated by means of the median-effect equation, where CI < 1, CI = 1, and CI > 1 represent synergism, an additive effect (or no interaction), and antagonism, respectively (Table 1).

To perform the experiment, the dilutions of doxorubicin were made in a horizontal direction in 100 µL, and the dilutions of the Se-compounds were made vertically in the microtiter plate in a volume of 50 µL. The cells were re-suspended in the culture medium and distributed into each well in 50 µL portions containing 6000 cells. The plates were incubated for 72 h at 37 °C in a CO_2_ incubator. The cell growth rate was determined after MTT staining. At the end of the incubation period, 20 μL of MTT solution was added to each well. After incubation at 37 °C for 4 h, 100 μL of SDS was added to each well and the plates were further incubated at 37 °C overnight. The optical density (OD) was measured at 540/630 nm with the Multiscan EX ELISA reader.

### 2.9. ABCB1 Inhibition in the Presence of Selenoesters

The inhibition of the ABCB1 multidrug efflux pump ABCB1 by the tested compounds was evaluated using flow cytometry, measuring the retention of rhodamine 123 by ABCB1 (P-glycoprotein) in Colo 320 colonic adenocarcinoma cells. Briefly, the cell number of colonic adenocarcinoma cells were adjusted to 2 × 10^6^ cells/mL, re-suspended in serum-free RPMI-1640 medium in the case of colonic adenocarcinoma cells, and distributed in 0.5 mL aliquots into Eppendorf centrifuge tubes. The tested compounds were added at different concentrations (0.2 and 2 µM; from 1 and 10 mM stock solutions, respectively), and the samples were incubated for 10 min at room temperature. Tariquidar was applied as the positive control (0.2 µM final concentration), and DMSO was used as the solvent control (at 2 *v/v*%). Next, 10 µL (5.2 µM final concentration) of the fluorochrome rhodamine 123 (Sigma, St. Louis, MO, USA) was added to the samples and the cells were incubated for 20 min at 37 °C. After the incubation period, the cells were washed twice and re-suspended in 0.5 mL PBS for analysis. The fluorescence of the gated cell population was measured with a Partec CyFlow^®^ flow cytometer (Partec, Münster, Germany). The percentage of the mean fluorescence intensity was calculated for the treated MDR cells as compared with the untreated cells. The results were obtained from a representative flow cytometry experiment in which at least 20,000 individual cells of the overall population were evaluated for the rhodamine 123 retained inside the cells. The fluorescence activity ratio (FAR) was calculated based on the following equation, which relates the measured fluorescence values:

$$\mathrm{FAR}=\frac{{\mathrm{Colo}320}_{\mathrm{treated}}{\;/\;\mathrm{Colo}320}_{\mathrm{control}}}{{\mathrm{Colo}205}_{\mathrm{treated}}{\;/\;\mathrm{Colo}205}_{\mathrm{control}}}$$

### 2.10. *P-gp ATPase Activity Assay*

P-glycoprotein ATPase activity was determined using the Pgp-Glo^TM^ Assay System (Promega, WI, USA). The assay was performed according to the manufacturer’s instructions. Next, 20 µL of recombinant human P-gp membranes (1.25 mg/mL) were incubated for 5 min in 20 µL of the Pgp-Glo^TM^ assay buffer at 37 °C. Compounds were tested at 25 µM. Sodium orthovanadate (Na_3_VO_4_, 0.25 mM) was applied as an inhibitor control, and verapamil was used as a substrate control (0.5 mM). DMSO at 2% was applied as a solvent control. The reaction was initiated by adding 10 µL of 25 mM MgATP, and incubated at 37 °C for 40 min. The reaction was stopped after adding 50 µL of ATP Detection Reagent. Then, the samples and controls were incubated at room temperature for 20 min. The emitted luciferase-generated luminescent signal was measured in a CLARIOstar Plus plate reader (BMG Labtech, UK) at 580 nm. The relative ATPase activity was calculated based on the ratio between the luminescence measured of the P-gp ATPase activity of each compound and the basal P-gp ATPase activity according to the following equation:

$$\mathrm{Relative}\;\mathrm{ATPase}\;\mathrm{activity}=\frac{{\mathrm{Lum}}_{\mathrm{treated}}-{\mathrm{Lum}}_{\mathrm{untreated}}}{{\mathrm{Lum}}_{{\mathrm{Na}}_{3}{\mathrm{VO}}_{4}\;}{-\;\mathrm{Lum}}_{\mathrm{untreated}}}$$

The effect of the tested compounds was evaluated according to the instructions of the manufacturer (Table 2).

### 2.11. Apoptosis

The assay was carried out using an Annexin V-FITC Apoptosis Detection Kit Cat. No. PF 032 from Calbiochem (EMD Biosciences. Inc. La Jolla. CA), according to the manufacturer’s instructions. The concentration of the Colo 320 cell suspension was adjusted to approximately 1 × 10^6^ cells/mL. The cell suspension was distributed into 1 mL aliquots (1 × 10^6^ cells) into a 24-well microplate and incubated overnight at 37 °C, with 5% CO_2_. On the following day, the medium was removed and a fresh medium was added to the cells. The cells were incubated in the presence of Se-compounds for 3 h at 37 °C. The concentration for the apoptosis induction (2 µM) was selected based on previous cytotoxicity results (IC_50_ values). Moreover, 12*H*-benzo(α)phenothiazine (M627) was used as positive control at 20 µM final concentration. After a 3 h induction period, the culture medium was removed, the cells were washed with PBS, and a fresh medium was added to the cells. The 24-well plates were incubated overnight at 37 °C, with 5% CO_2_. After the incubation, the supernatant was collected in a microfuge tube and 200 μL of 0.25 trypsin (Trypsin-Versene) was added to the wells until the cells detached from the surfaces of the wells. The cells were centrifuged at 2000× *g* for 2 min at room temperature, the supernatant was removed, and the cells were re-suspended in fresh serum-free medium. After this procedure, apoptosis assay was carried out according to the rapid protocol of the kit using Annexin V-FITC and propidium iodide staining. The fluorescence was analysed immediately using a ParTec CyFlow^®^ flow cytometer (Partec, Münster, Germany), and the results were obtained from a representative flow cytometry experiment in which at least 20,000 individual cells of the overall population in a sample were evaluated. The data were analysed by FlowJo^TM^ software (BD Biosciences, San Jose, NJ, USA).

### 2.12. Wound Healing Assay

Wound healing was measured in vitro by scratch assay, as the migration rate of the cells to close the gap was created, as described by Ling et al. [19], with slight modifications. Briefly, HaCaT cells at the concentration of 5×10^5^ cells were seeded into 12-well plates and incubated at 37 °C and 5% CO_2_ until the cells formed a uniform layer. After layer formation, the cells were washed with PBS and then scratched with a sterile micropipette tip. The debris was removed by further gentle washing with PBS, then the samples (IC_10_), along with the positive control (Allantoin, 50 µg/mL), were mixed with DMEM and added directly to the cells. The images of the scratch area were captured using an inverted microscope in 4× magnification at different time intervals (0 h, 24 h, 48 h) and evaluated using ImageJ software (National Institutes of Health, Bethesda, MD, USA). All of the experiments were performed in four replicates, and a minimum of five measurements were considered from each image captured. Dean–Dixon’s test was used to remove the outliners in the values measured, and further statistical significances between the groups were established by *t*-test (Excel, Microsoft Office, Redmond, WA, USA). The wound closure was calculated as the ratio of the gap size at the beginning minus the gap size at the evaluated time.

## 3. Results

### 3.1. Synthesis and Characterization of the Compounds

The ketone derivatives have been designed as derivatives of the potent oxoselenoesters previously reported [15,16,17,18], attempting to improve their activity and selectivity. The compounds published in these works were the Se-(2-oxopropyl) 4-clorobenzoselenoate (compound 9), the Se-(3,3-dimethyl-2-oxobutyl) 4-clorobenzoselenoate (compound 10), and the Se-(3,3-dimethyl-2-oxobutyl) 3,5-dimethoxybenzoselenoate (compound 11). Cyanoselenoesters have been designed as a variation of the ones included in a previous patent of the group [18], with the same improvement aim of the activity showed by the initial cyanoselenoesters included in this patent.

Seeing the noteworthy activity in previous works [15,16,17,18] shown by derivatives that contain halogens, we have considered herein different halogenated substituents (bromine, fluorine, trifluoromethyl, chlorine), as well their polysubstitution, to determine which ones enhanced the biological effects. Additionally, compounds with a thiophene ring or with a 4-*tert*-butyl substituent are included to evaluate the activity of compounds that contain heterocycles or electron-donating substituents, respectively.

The new 15 compounds reported in this work were pure and chemically stable at room temperature. All of them were pure in the Elemental Analysis, following the threshold of a maximum variation of ±0.4% for every element tested (C, H, N, S). The yield is markedly affected by the state of the matter of the product: generally, liquid compounds require purification by column chromatography, and its handling is more troublesome, which is reflected in the yields. Typically, solid compounds showed yields in the range from 40% to 50%, with the exception of compound **K6** (18%); whereas liquid compounds have lower yields, from 7% to 29%. As mentioned above, the synthetic route consisted of three steps: an initial in situ preparation of the selenating agent, and the attack of this agent to the suitable benzoyl or thienyl chloride to form an acyl selenide salt that exerts a nucleophilic attack over a suitable alkyl halide (chloroacetone for derivatives **K1**–**K8** and chloroacetonitrile for compounds **N1**–**N7**). A few derivatives needed a preliminary step to prepare the required benzoyl chloride from the respective benzoic acid. These compounds were: **K5** and **N5** from 3-(trifluoromethyl)benzoic acid; **K6** and **N6** from 3-chloro-4-fluorobenzoic acid; **K8** from 2,4,5-trifluorobenzoic acid; and **N7** from 3,5-bis(trifluoromethyl)benzoic acid.

Compounds **K1**–**K8** have, as a common feature the –COSeCH_2_COCH_3_ moiety bound to the phenyl or thienyl ring. In ^1^H-NMR, the CH_2_ appears as a singlet accompanied by two small satellite peaks (due to the influence of the adjacent selenium atom) in the range 3.89–3.96 ppm, and the CH_3_ in the range 2.33–2.36 ppm. Alternatively, in ^13^C-NMR, the CH_2_ again appears as a singlet accompanied by two small satellite peaks (due to the influence of the adjacent selenium atom) in the range 34.5–36.0 ppm, and the CH_3_ in the range 28.6–29.2 ppm. The carbonyls appear in different ranges in ^13^C-NMR: there is a higher and narrower range of 203.1–204.1 ppm for the ketone carbonyl, and a less displaced and wider range of 183.0–193.3 ppm for the carbonyl group of the selenoester. The width of the ranges is logical, as the width increases when the substituted phenyl ring is closer. Similarly, in the case of the **N1**–**N7** derivatives, the common structural moiety is the -COSeCH_2_CN bound to the phenyl or thienyl ring. The –CH_2_ bound to the selenium and to the cyano group, which appears in ^1^H-NMR in the range from 3.70 to 3.79 ppm. This carbon is seen in ^13^C-NMR at a very low displacement (ranging from 5.5 to 7.0 ppm). The -CN group is observed in a very narrow range (116.4–117.2 ppm), and the -COSe appears at a slightly lower range than the -COSe of the ketone derivatives (180.4–191.2). The remaining signals depend on the ring (thienyl for **K1** and **N1**, and phenyl for the remaining compounds), and on the substituents (and their position) bound to the phenyl ring. For a few compounds (**K1**, **K3**, **K7**, **K8**, **N1**, and **N5**), bidimensional NMR spectra as ^1^H-^1^H COSY, ^1^H-^13^C HSQC, and ^1^H-^13^C HMBC have been recorded to help in the correct assignment of the signals. Of these bidimensional spectra, HMBC was of particular interest, as the spectra obtained proof of the -COSeCH_2_CN or the -COSeCH_2_COCH_3_ thanks to the long-distance interactions between the different atoms involved (see spectra in Supplementary: Figures S1H, S3H, S7G, S8I, S9H, and S13I).

In mass spectrometry, the most abundant ion is always the product of the breakage of the carbon–selenium bond of the selenoester, releasing as a positive ion the acyl cation (which is the peak with 100% of abundance) and an anion that includes the selenium atom. From this acyl cation, different cations are also observed, including the release of the thiophenium cation (compounds **K1** and **N1**) or the appropriate phenyl cation. The latest overcomes different releases (for example: HF, HCl, and HBr) depending on the substituents present at the ring. The easiness of the breakage of the C–Se bond implies that the molecular ion always has a very low abundance, and it was even not observed in four compounds (**N3**, **N4**, **N6**, and **N7**). Additionally, the molecular ion showed an abundance below 0.5% in 5 compounds of the 11 remaining. Interestingly, abundances of the molecular ion were always higher at ketone-selenoesters (from 0% to 2%) than at cyanoselenoesters (not observed or below 0.5%). Perhaps this fact may point out that, at least in the conditions of the ionization chamber, the first are more stable.

Regarding infrared spectroscopy, all ketone-selenoesters showed two peaks in the range of 1720–1650, where the C=O stretch of carbonyl compounds are usually observed. The carbonyl of the selenoester had a lower (from 1650 to 1679 cm^−1^), and a wider range than the one experimentally determined for the carbonyl of the ketone (from 1697 to 1712 cm^−1^), due to the fact that it was the carbonyl group in the molecule which is closer to the substituents than the ketone carbonyl. The exception was the compound **K4**, which produced a wider overlapping signal that incorporated the two carbonyl compounds. Alternatively, the IR spectra of the nitrile derivatives have, as a common feature, the C≡N stretch and the C=O stretch of the selenoester. They appeared in the ranges 2239–2247 cm^−1^ and 1662–1706 cm^−1^, respectively.

### 3.2. Cytotoxicity

Based on the obtained results, ketone-selenoesters showed a potent cytotoxic effect on both sensitive and resistant colon cancer cell lines, where the IC_50_ values were between 1 and 4 µM on both cell lines. In addition, similar toxic activity was observed on MRC-5 normal lung fibroblast cells, indicating that ketone-selenoesters have no selectivity towards cancer cells. Cyanoselenoesters showed no effect on the MRC-5 cell line (IC_50_ was more than 100 µM); however, they were very toxic on both colon cancer cell lines. Comparing the sensitive Colo 205 (IC_50_: 1.98–2.96 µM) to the resistant Colo 320 cells (IC_50_: 3.78–7.64 µM), cyanoselenoesters were less potent on the resistant cells. Furthermore, both ketone- and cyanoselenoesters were more active on cancer cell lines than the positive control doxorubicin. Selectivity indexes (SI) were calculated as described above. Cyanoselenoesters showed high selectivity (>6) in each case, and ketone-selenoesters showed moderate (3 < SI < 6) and slight (1 < SI < 3) selectivity, except **K2** (SI < 1). (Table 3).

Similar results were obtained in cancer cells from other locations, such as liver, cervix, and skin. Ketone-selenoesters showed the following range of IC_50_ values in these cancer cells (Table 4): from 2.2 to 4.3 µM towards hepatocellular carcinoma (HepG2) cells, from 1.9 to 2.7 µM towards cervical adenocarcinoma (HeLa) cells, and from 1.1 to 2.0 µM towards skin murine melanoma (B16) cells. Regarding cyanoselenoesters, the observed range of IC_50_ values in the abovementioned cell lines were from 5.2 to 11.8 µM towards HepG2 cells, from 1.3 to 5.2 µM towards the HeLa cell line, and from 1.4 to 2.6 µM towards B16 cells. SI values were again higher than 6 for all of the cyanoselenoesters in HepG2 and HeLa cells, indicating that all of the cyanoselenoesters were strongly selective towards the cancer cells in respect to MRC-5 cells. In contrast, none of the ketone-selenoesters showed an SI value higher than three (moderately selective) for these two additional human cancer cells.

### 3.3. Checkerboard Combination Assay

The checkerboard combination assay is a widely used and convenient *in vitro* method for the assessment of drug interactions among various pharmacological agents. This program enables the calculation of the combination indices, and also allows the determination of the most effective ratios of combinational agents. Ketone- and cyanoselenoesters were combined with doxorubicin, and their interactions were determined after MTT staining. The data obtained were analysed using Calcusyn software.

Six ketone-selenoesters (**K1**, **K3**, **K4**, **K5**, **K6**, **K8**) were found to interact synergistically with doxorubicin. Furthermore, **K5** and **K6** showed a synergistic interaction with doxorubicin in all ratios (Table 5). Additionally, a synergistic effect can be seen for five cyanoselenoesters (**N1**, **N2**, **N3**, **N4**, **N7**) (Table 6). The type of interaction in the combination studies was evaluated using the Chou–Talalay method for drug combination, which is based on the median-effect equation. The prerequisite for the calculation is the dose-effect curves for each drug alone. The combination of two drugs at a certain ratio behaves like a third drug to the cells. In this way, the parameters can be obtained for the mixture, just in case the of the single drugs, by using the automated median-effect plot with computer software. Applying this method, several ratios can be tested and different types of interactions can be obtained, allowing for a more precise description of the interaction of the compounds [20].

### 3.4. ABCB1 Inhibition in the Presence of Selenoesters

**Figure 2:**
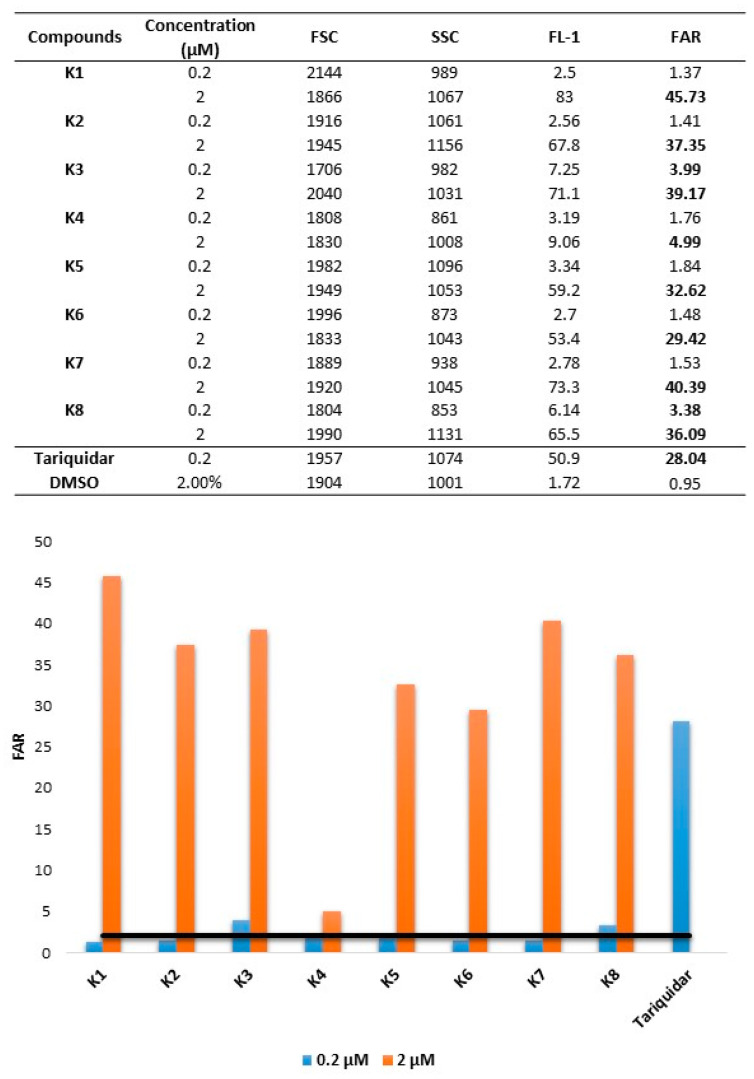
ABCB1 (P-gp) inhibition in the presence of selenoesters in MDR Colo 320 cells measuring the intracellular accumulation of the ABCB1 substrate rhodamine 123 by flow cytometry. The FAR (fluorescence activity ratio) values were calculated based on the equation given in Section 2.9. Tariquidar was applied as positive control; DMSO was used as solvent control. Results above FAR 2 (black line, highlighted in bold) are considered effective.

The inhibition of the ABCB1 transporter was assessed by measuring the intracellular accumulation of its fluorescent substrate rhodamine 123 at 0.2 and 2 µM. Based on the flow cytometric evaluation, ketone-selenoesters inhibited the activity of the ABCB1 transporter, and the most potent derivatives were **K1**, **K2**, **K3**, **K7**, and **K8**, exhibiting a FAR value of 45.73, 37.35, 39.17, 40.38, and 36.09 at 2 μM, respectively. Even the less potent derivatives showed similar activity at 2 µM: in the case of **K5**, the FAR value was 32.61. Furthermore, in the presence of **K6**, a FAR of 29.4 was obtained. The less active derivative was **K4**, with a FAR value of 4.99 at 2 µM. **K3** and **K8** were effective at 0.2 µM and 2 µM concentration (FAR at 0.2; µM at 3.99 and 3.38, respectively), while the other compounds were only effective at 2 µM concentration (Figure 2). Cyanoselenoesters did not show ABCB1 modulating activity.

The parameters evaluated from flow cytometry experiments were: Forward Scatter Count (FSC, provides information about cell size); Side Scatter Count (SSC, proportional to cell granularity or internal complexity); FL-1 (Mean fluorescence of the cells), and Fluorescence Activity Ratio (FAR), which was calculated by the equation given above.

### 3.5. P-gp ATPase Activity Assay

**Figure 3:**
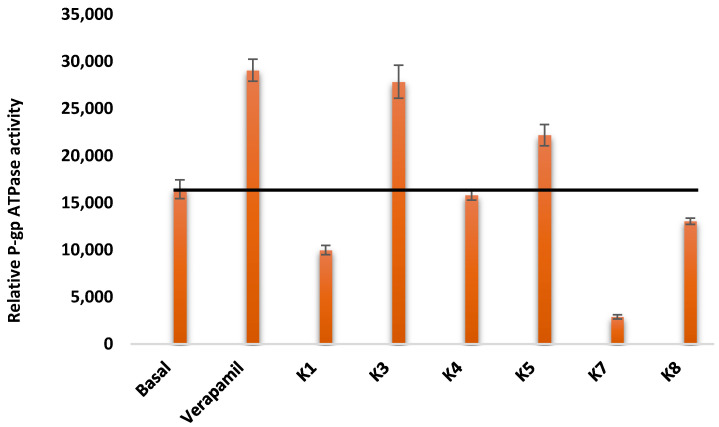
Relative ABCB1 (P-gp) ATPase inhibition activity of selected ketone-selenoesters. The effects are presented as the relative ATPase activity. The decrease in luminescence of untreated samples compared to samples treated with sodium-vanadate represents basal P-gp ATPase activity. The decrease in luminescence of verapamil-treated samples represents verapamil-stimulated P-gp ATPase activity. The lower the relative ATPase activity, the better the inhibitor. Results are calculated as the means ± SD from experiments performed in triplicate. The level of basal activity is presented as a black line.

Only the compounds with ABCB1 inhibitory activity were tested in this assay. As shown in Figure 3, ΔRLU of **K1**, **K7**, and **K8** were significantly lower than ΔRLU_basal_, demonstrating that these compounds are inhibitors of P-gp ATPase activity. The rest of the compounds are stimulators of P-gp ATPase activity. Verapamil is a P-gp substrate that stimulates ATPase activity and served as a control in this assay. In the cases of **K2** and **K6,** the P-gp ATPase activity could not be determined (Figure 3).

### 3.6. Induction of Apoptosis

**Figure 4:**
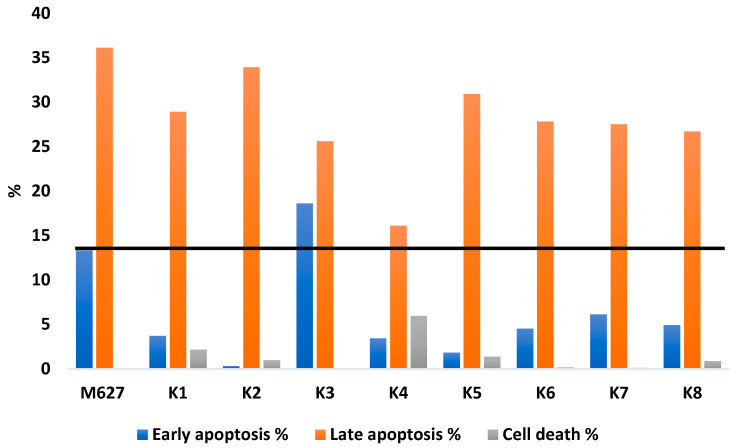
Apoptosis induction by ketone-selenoesters on MDR Colo 320 adenocarcinoma cells. The 12*H*- benzo(α)phenothiazine (M627) was applied as a positive control. The cell populations were analysed by flow cytometry after Annexin V-FITC and propidium iodine staining. The figure represents the percentage of early apoptotic cells (annexin positive, propidium iodide negative); late apoptotic cells (annexin positive, propidium iodide positive) (A+, PI+); and dead cells (annexin negative, propidium iodide positive) (A−, PI+). The black line demonstrates the percentage of early apoptosis in the presence of the positive control M627.

The potent ABCB1 inhibiting ketone-selenoesters were tested regarding their apoptosis-inducing activity on MDR Colo 320 colon adenocarcinoma cells (Table 7 and Figure 4).

The results were compared to 12*H*-benzo(α)phenothiazine (M627) as a positive control. The most potent compound, **K3**, could induce early apoptosis in 18.6% of the cell population, showing a higher capacity to trigger these early apoptotic events than the reference. Interestingly, all derivatives contributed to late apoptosis, and their activity ranged from 16.1 to 33.9%. **K3** induced late apoptosis in 25.6% of the cell population. Considering together the early and late apoptosis (as total apoptotic events), all of the compounds, with the exception of **K4**, induced apoptotic events in more than 30% of the gated cells. After defining the apoptosis quotient as the quotient of the total apoptotic events of the compound divided by the reference and expressed as a percentage, all of the compounds, except **K4**, showed an apoptosis induction ability equal to 64–90% more than the reference, and a concentration 10-fold lower than the reference. Compound **K3** was the most potent with 89.5%, followed by **K2** with 69.2%. The least effective was **K4**, with 39.5%. During early apoptosis, phosphatidyl serine (PS) appears on the outer membrane, which can be detected by annexin V; however, the membrane has not yet disintegrated. During late apoptosis, the cell membrane is already damaged (in this case, annexin is also able to bind to PS), resulting in the release of DNA, to which propidium iodide is able to bind.

### 3.7. Wound Healing Assay

Wound healing activity of selenoesters was evaluated as the ability of the cells to migrate and close the gap created on the monolayer of human keratinocytes (HaCaT cell line). The tested substances were applied in the highest possible non-toxic concentration (IC_10_, concentration inhibiting 10% of the population). In general, ketone-selenoesters were more toxic (except **K4** and **K1**) than cyano-selenoesters (except **N4**); therefore, they were applied at lower doses (0.17–0.30 µM) when compared to cyanoselenoesters (0.36–0.64 µM). Compounds **K1**, **K4**, and **N4** were applied at a concentration of 0.30, 0.46, and 0.20 µM, respectively. As could be seen in Table 8, all ketone-selenoesters stimulated wound healing more effectively in comparison to both untreated control (*p* < 0.0000005) and allantoin (*p* < 0.00005). As a result, all of the tested ketone-selenoesters exhibited around 80% of gap closure within 24 h. After 48 h, samples **K1**, **K2**, **K3**, **K5**, **K7**, and **K8** exhibited complete gap closure, and samples **K4** and **K6** were able to close the gap with a closure percentage of 86% and 93%, respectively. In contrast, cyano-selenoesters were less active than ketone-selenoesters, as none of the samples were able to close the gap completely. Samples **N3**, **N5**, and **N6** exhibited slower closure of the wound, and was slower than in the untreated control (*p* < 0.005) within 24 h (closure percentage was only around 45%). Only samples **N1**, **N2**, **N4**, and **N7** closed effectively the damaged wound in 24 h, with the gap closure around 70%. Despite the fact that cyano-selenoesters were less potent in exhibiting the cell mobility for the wound healing, the samples **N1**, **N4**, and **N7** were still even slightly better than the positive control (*p* < 0.005, *p* < 0.05, and *p* < 0.05, respectively), and other cyano-selenoesters, with a closure percentage of around 80%.

## 4. Discussion

### 4.1. Synthesis and Characterization of the Compounds

As mentioned in the Results section, the yield of the synthesis depends mostly on the purification procedure and on the matter state of the compound: generally, liquids are required to perform column chromatography, which reduces the yield. Solids showed a yield between 40% and 50%, and liquids showed a yield between 7% and 29%. Even for solids, the yield tends to be low. This may be related to the solubility of the compounds in water. As compounds are obtained (when they are solid) as a precipitate that is formed in the reaction media, part of the compound can remain in solution. Even knowing from previous works that selenoesters are poorly soluble in water, the usage of a significant volume during washings can be reflected in a significantly lower yield than the one expected.

The spectral data are fully described in the compound descriptions of Section 2.3, which discusses the chemical description of the compounds. Spectra enabled the correct assignments and the proper characterization of the structure of the compounds. As mentioned in the Results section, HMBC spectra were of particular interest to ensure the formation of the selenoester. A second proof was the isotopic pattern observed in the mass spectra of the oxoselenoesters in which the molecular ion has a higher abundance, as indicated by **K2**, **K5**, **K6**, and **K8**. Another indirect proof is the observation in ^13^C-NMR that the range of the peak chemical shift is always narrower for the carbonyl of the ketone or for the cyano group than for the carbonyl of the selenoesters. This is logical as the ketone or the cyano group are at a higher distance from the ring substituents, which are more responsible for the observed variations, than the carbonyl of the selenoester. A similar observation was found with the range of the bond stretches, C=O (COCH_3_) and C≡N, as compared to the C=O (COSe) stretch.

### 4.2. Anticancer Activity

Oxoselenoesters, in general (a few exceptions are observed), showed a more potent cytotoxicity than cyanoselenoesters in MDR Colo 320, HepG2, and B16 cancer cell lines. This difference of activity is particularly marked in Colo 320 and HepG2 cells. Cyanoselenoesters showed no activity on the MRC-5 cells; however, they were very toxic on both colon cancer cell lines, and were less potent on the resistant cells. Regarding the most active compound in each cell line (the second and third most active in brackets after cell line), these were as follows: **K4** in Colo 205 (**K1**, **N4**); **K1** in Colo 320 (**K4**, **K6**); **K2** in HepG2 (**K1**, **K5**); **N6** in HeLa (**K4**, **K3**); and **K5** in B16 cells (**K2**, **K6**). In line with the overall impression, the majority of them are oxoselenoesters, and among them, all appeared at least once, except **K7** and **K8**. This may point out that the presence of substituents without halogen(s)—as is the case of **K7**—reduces the activity, as the compounds that include a trifluoromethyl group or that have one or two halogens bound to the phenyl ring showed better activity. However, the inclusion of a third fluorine at the phenyl ring (**K8**) is also less profitable for the cytotoxicity. On the other hand, the recurrent appearance of **K1** and **K4** among the most active compounds (three times each) against each cell line points out that the thionyl ring favours the cytotoxicity, as well as the presence of a trifluoromethyl group in ortho position (in the phenyl ring) in respect to the selenoester. Interestingly, **K5** (which has a 3-CF_3_ substituent at the ring) also exerts noteworthy activity, and the cyanoselenoester, **N4**, with a 2-CF_3_ substituent at the ring, is perhaps the most active derivative among the cyanoselenoesters. This supports the empirical observation of the relevance of this trifluoromethyl group (preferable in ortho position, although the compound with this substituent in meta position has a comparable activity in all cell lines, except in HepG2, in which it is significantly less active) for the cytotoxic activity.

The ketone-containing selenoesters showed toxicity towards normal MRC-5 cells, whereas none of the cyano-containing derivatives exerted toxicity against this cell line at concentrations below 100 μM. This implies that all of the cyanoselenoesters were strongly selective (SI > 6) towards cancer cells. They were especially selective towards B16 skin melanoma cells, as **N4** and **N5** showed an SI higher than 71.4, and **N7** showed an SI higher than 62.5. Compound **N6** was also extremely selective towards HeLa cervical adenocarcinoma cells (SI >76.9). More compounds showed SI values higher than 50 towards cancer cells: **N1** and **N6** in B16 cells, **N1** in HeLa cells, and **N4** and **N7** in Colo 205 cells. Ketone-selenoesters, in contrast, were much less selective. Only **K4** showed a moderate selectivity towards Colo205 cells (3 < SI < 6). The remaining compounds were slightly selective (1 < SI < 3) towards the tested cancers cells in respect to MRC-5 non-cancer cells. Even a few ones were non-selective (SI < 1): **K3**, **K4**, and **K6** towards HepG2 cells, and **K2** towards Colo205 cells. This indicates a risk of side effects, and further research is necessary to find compounds with a similar potency and more selectivity.

Regarding the combination of ketone-selenoesters and cyanoselenoesters with the cytotoxic drug doxorubicin, eleven of the fifteen selenoesters evaluated interacted in a synergistic manner with doxorubicin in at least one of the ratios tested: **K1**, **K3**, **K4**, **K5**, **K6**, **K8**, **N1**, **N2**, **N3**, **N4**, and **N7**. No logical structure-activity relationships (SARs) can be extracted, as **K5** and **K6** interacted in different synergism degrees with doxorubicin in all of the ratios, whereas their nitrile equivalents (**N5** and **N6**) showed antagonistic interactions for five of the six ratios tested. Similarly, the thiophene cyanoselenoester **N1** showed a synergistic interaction with doxorubicin in four of the six ratios assayed, while its ketone analogue **K1** only showed synergism in one ratio out of five. This **N1** derivative was also capable of interacting with a strong synergism, at a 54.4:1 ratio with doxorubicin.

To reverse efflux-related MDR, the inhibition of the ABCB1 pump was investigated in the presence of selenocompounds. The 2-oxopropyl moiety of the ketone-selenoesters is crucial for the activity, as all ketone-selenoesters, except **K4**, were more potent ABCB1 inhibitors, whereas none of the cyanoselenoesters showed ABCB1 inhibiting activity. This is also in line with previous works [15,16], in which both this moiety and a 3,3-dimethyl-2-oxobutyl moiety showed very potent activity.

The most active compound is **K1**, which contains a thiophene ring instead of a phenyl ring. In this case, the insertion of a bulky trifluoromethyl group at the two-position of the phenyl ring significantly reduced the activity (**K4**): simply moving this group to the three-position and eliminating the steric hindrance produced a sixfold increase of the activity (**K5**). Interestingly, two oxoselenoesters that showed a less marked cytotoxicity (**K7**, with a 4-tert-butylphenyl ring; and **K8**, with a 2,4,5-trifluorophenyl ring) were strong inhibitors of ABCB1, with a similar activity to **K2** and **K3**, with 2-fluorophenyl and 4-bromophenyl moieties, respectively.

Regarding the P-gp ATPase activity, only the oxoselenoesters were tested as they were the only ones with ABCB1 inhibitory activity. Among them, the P-gp ATPase activity of **K2** and **K6** could not be determined, and all of the remaining tested compounds, except **K4** (whose activity was similar to the one observed for the basal control), modulated the ATPase activity. Interestingly, **K1** and **K7**, which where the most potent ABCB1 inhibitors, inhibited the ATPase activity, especially in the case of **K7**. Besides, **K8** exerted a milder inhibition of the ATPase. Since the activity of ABCB1 can protect the cells from apoptosis, the inhibition of the energy supply of this pump can promote apoptosis, as demonstrated by our results [21]. The abovementioned derivatives (**K1**, **K7**, and **K8**) induced late apoptosis in MDR Colo 320 cells, confirming the connection between ABCB1 inhibition and apoptosis induction.

On the other hand, K3 stimulated the P-gp ATPase with a comparable intensity to the reference verapamil, whereas **K5** produced a milder stimulation. Then—keeping in mind that the data available are scarce—with this data, the thienyl ring and the 4-tert-butylphenyl and 2,4,5-trifluorophenyl moieties inhibited the ATPase activity, whereas the 4-bromophenyl and the 3-(trifluoromethyl)phenyl moiety stimulated it. Finally, moving the trifluoromethyl group from the three- to the two-position eliminated this promotion of the ATPase activity.

The connection between ABCB1 inhibition and apoptosis induction was investigated in the case of ketone-selenoesters. The ketone-selenoesters showed a significant ability to trigger apoptotic events, with the exception of **K4**. Compound **K3** was more effective than the reference phenothiazine in the induction of early apoptosis, and is the one that, considering together the early and late apoptosis, induced apoptosis with a potency closer to the reference (89.5%). As all of the remaining derivatives assayed showed a similar ability to induce apoptosis, no SARs can be extracted with the available data. Besides, it is evident that the inclusion of a bromine atom at the four-position of the phenyl ring increases the apoptosis induction (**K3**), and the inclusion of a bulky substituent at the two-position (as the trifluoromethyl moiety) of the phenyl ring reduces the ability to trigger apoptotic events. In addition, the presence of selenium can induce the formation of free radicals, resulting in apoptosis and cell death in cancer cells [22,23,24]. The functions of MDR transporter proteins (most notably ABCB1) have been described in apoptosis evasion, mediated by a dampening of the extrinsic apoptotic pathway (through suppression of TRAIL protein and caspases three and eight) and the stabilization of cell membrane phospholipids (through acting as an outwardly directed flippase). The inter-relatedness of overexpressed efflux pumps and programmed cell death may explain the results obtained in the apoptosis detection assay [21].

In general, selenium in the form of selenoproteins and selenocompounds has always been known as an excellent candidate for wound healing, as some of them have exhibited their ability to act as antioxidants and inhibitors of inflammation. Selenocompounds act as inhibitors of cytokines and eliminators of peroxynitrate, which is a super radical ion in the inflammatory phase [25]. As described in the results, all of the eight ketone-selenoesters stimulated an effective wound healing process, demonstrating a better healing ability than the positive control allantoin. Six of them (**K1**, **K2**, **K3**, **K5**, **K7**, and **K8**) were even capable of repairing the wound completely. Only the compounds with a bulky substituent in the *ortho* position (trifluoromethyl, **K4**) or with two different substituents (**K6**, 3-chloro-4-fluorophenyl) were not capable of completely closing the wound. Regarding the cyano-selenoesters, they were significantly less effective in this assay than the ketone-selenoesters. Only one of the seven cyano-containing derivatives, compound **N1**, displayed better closure than allantoin (especially after 24). Three additional ones, **N2**, **N4**, and **N7**, were more effective than the negative control. In this case, no reliable SARs can be extracted, but the removal of one of the two trifluoromethyl substituents from compound **N7** resulted in significant wound healing activity loss, which serves as the main reason for the inability of compound **N5** to close the gap. Similarly, compound **N6** and **N3** had the same effect as compound **N5**.

If a few compounds would need to be selected among all derivatives to proceed to more in-depth studies, perhaps the most promising are **K1**, **K3**, and **K5**. The oxoselenoester **K1**, which has a thiophene ring bound to the carbonyl of the selenoester, is a potent cytotoxic compound that exerts a strong inhibition of the ABCB1 efflux pump and of the ATPase activity, and also has the ability of apoptosis induction and the capacity to promote a complete closure of a wound. Similarly, the 2-oxopropyl 4-bromobenzoselenoate (**K3**) has a noteworthy cytotoxic activity, and strongly inhibits the ABCB1 efflux pump, but stimulates the ATPase activity. Besides, it is the most potent apoptosis inducer among the tested compounds and also manages to complete the closure of a wound in 48 h. Finally, the 2-oxopropyl 3-(trifluoromethyl)benzoselenoate (**K5**) has a similar effect than **K3**, but with a less potent apoptosis-inducing ability and less capacity to enhance P-gp ATPase activity. In exchange, it could interact in a synergistic manner with doxorubicin when administered in combination, in the six ratios tested. Between these three derivatives, all of the activities tested are covered, as are the two ways of action in the case of the ATPase assay. The compound 2-oxopropyl 3-(trifluoromethyl)benzoselenoate (**K4**) seemed to be a promising derivative in cytotoxicity assay, but later showed a poor effectivity in ABCB1 inhibition, ATPase modulation, apoptosis induction, and wound healing, so it is clearly a less multitarget compound than **K1**, **K3**, and **K5**. Perhaps the presence of a bulky substituent in a position close to the selenoester can affect the interaction of the selenium atom with the different cellular targets. On the other hand, it may affect its hydrolysis, as this is the hypothesized mechanism of action for these compounds, according to previous works [16].

In contrast, the cyanoselenoesters are more selective compounds, but they are generally less cytotoxic, weaker promoters of wound healing, interacted in a less synergistic manner with doxorubicin in combination assay, and did not inhibit the ABCB1 protein.

## 5. Conclusions

Herein, we described the design, synthesis, and characterization of fifteen novel selenoesters, as well as the evaluation of their activity against a wide selection of different targets related to cancer multidrug resistance. Of these selenoesters, the alkyl moiety of eight included a ketone group, whereas the seven remaining contained a cyano group. All of the compounds showed IC_50_ values between 1 and 12 μM in the five cancer cell lines evaluated. The oxoselenoesters were generally more cytotoxic, while the cyanoselenoesters were more selective towards cancer cells in respect to non-cancer cells. Besides, the majority of the obtained oxoselenoesters were potent ABCB1 inhibitors, enhanced the activity of doxorubicin in a synergistic manner (at least in any of the ratio concentrations tested), and modulated the P-gp ATPase activity. All of the oxoselenoesters showed an apoptosis induction capacity and an ability to promote wound healing. Therefore, these novel selenocompounds have shown noteworthy multi-target anticancer activity that converts them into a promising starting point to develop more effective and selective anticancer agents.

## Figures and Tables

**Table 1:** Type of interactions based on the combination indexes.

Combination Index (CI)	Type of Interaction	Combination	Type of Interaction
0–0.1	very strong synergism	0.9–1.1	additive effect
0.1–0.3	strong synergism	1.1–1.2	slight antagonism
0.3–0.7	synergism	1.2–1.45	moderate antagonism
		1.45–3.3	antagonism
0.7–0.85	moderate synergism	3.3–10	strong antagonism
0.85–0.9	slight synergism	>10	very strong antagonism

**Table 2:** Evaluation of Pgp ATPase activity.

∆RLU_TC_ > ∆RLU_basal_	the tested compound is a stimulator of Pgp ATPase activity
∆RLU_TC_ = ∆RLU_basal_	the tested compound has no effect on Pgp ATPase activity
∆RLU_TC_ < ∆RLU_basal_	the tested compound is an inhibitor of Pgp ATPase activity

RLU = Relative Light Unit; TC = tested compound. The difference in luminescent signal between Na_3_VO_4_-treated samples and untreated samples represents the basal Pgp ATPase activity.

**Table 3:** Cytotoxic effect of selenocompounds on sensitive (Colo 205) and resistant (Colo 320) colon adenocarcinoma, and MRC-5 normal embryonal fibroblast cell lines and selectivity indexes (SI). Doxorubicin was used as a positive control.

Cpds.	Colo 205 (IC_50_ µM)		Colo 320 (IC_50_ µM)		MRC-5 (IC_50_ µM)		SI	SI
	Mean	SD	Mean	SD	Mean	SD	MRC-5/Colo 205	MRC-5/Colo 320
**K1**	1.53	±0.46	1.47	±0.02	2.24	±0.29	1.46	1.52
**K2**	3.35	±0.58	2.38	±0.23	2.53	±0.40	0.76	1.06
**K3**	2.28	±0.05	2.15	±0.03	2.86	±0.36	1.25	1.33
**K4**	1.05	±0.04	1.48	±0.06	3.63	±0.37	3.46	2.45
**K5**	2.14	±0.08	2.17	±0.27	3.11	±3.93	1.45	1.43
**K6**	2.10	±0.02	2.10	±0.05	3.62	±0.41	1.73	1.72
**K7**	2.69	±0.07	2.57	±0.15	3.72	±0.17	1.38	1.45
**K8**	2.24	±0.16	2.37	±0.11	2.50	±0.06	1.12	1.05
**N1**	2.37	±0.27	7.64	±0.15	>100	-	>6 *	>6 *
**N2**	2.96	±0.09	7.01	±0.69	>100	-	>6 *	>6 *
**N3**	2.10	±0.06	4.37	±0.10	>100	-	>6 *	>6 *
**N4**	1.97	±0.14	5.57	±0.23	>100	-	>6 *	>6 *
**N5**	2.10	±0.10	5.22	±0.08	>100	-	>6 *	>6 *
**N6**	2.24	±0.07	5.19	±0.37	>100	-	>6 *	>6 *
**N7**	1.98	±0.16	3.78	±0.23	>100	-	>6 *	>6 *
**Dox.**	3.46	±0.34	7.61	±0.29	2.73	±0.34	0.79	0.36

Data are presented as the average of three measurements with the respective standard error of the mean. Dox. = doxorubicin. * The derivatives were not toxic on normal human fibroblast (MRC-5). For this reason, the numeric value of selectivity could not be precisely determined; however, all derivatives proved to be selective.

**Table 4:** Cytotoxic effect of selenocompounds on hepatocellular carcinoma (HepG2), cervical adenocarcinoma (HeLa), and skin melanoma (B16) cell lines and selectivity indexes (SI).

Cpds.	HepG2 (IC_50_ µM)		HeLa (IC_50_ µM)		B16 (IC_50_ µM)		SI	SI	SI
	Mean	SD	Mean	SD	Mean	SD	MRC-5/HepG2	MRC-5/HeLa	MRC-5/B16
**K1**	2.3	±0.2	2.5	±0.2	1.4	±0.1	1.6	1.5	2.6
**K2**	2.2	±0.215	2.5	±0.5	1.2	±0.1	1.1	1.0	2.1
**K3**	3.1	±0.2	2.1	±0.2	1.7	±0.2	0.7	1.1	1.3
**K4**	4.3	±0.1	1.9	±0.0	2.0	±0.2	0.7	1.5	1.4
**K5**	2.4	±0.2	2.5	±0.1	1.1	±0.1	1.3	1.2	2.8
**K6**	2.9	±0.3	2.3	±0.1	1.3	±0.1	1.3	1.6	2.8
**K7**	3.7	±0.3	2.7	±0.3	1.4	±0.1	1.0	1.4	2.7
**K8**	4.0	±0.2	2.0	±0.0	1.4	±0.1	0.6	1.3	1.8
**N1**	11.3	±0.9	2.0	±0.1	1.9	±0.4	>6 *	>6 *	>6 *
**N2**	5.6	±0.3	5.2	±0.5	2.6	±0.4	>6 *	>6 *	>6 *
**N3**	9.6	±0.9	2.4	±0.1	2.8	±0.5	>6 *	>6 *	>6 *
**N4**	5.2	±0.3	2.5	±0.0	1.4	±0.3	>6 *	>6 *	>6 *
**N5**	9.8	±0.6	2.5	±0.2	1.4	±0.3	>6 *	>6 *	>6 *
**N6**	9.6	±0.4	1.3	±0.1	1.7	±0.3	>6 *	>6 *	>6 *
**N7**	11.8	±1.2	2.1	±0.1	1.6	±0.4	>6 *	>6 *	>6 *

* The derivatives were not toxic on normal human fibroblast cells (MRC-5) up to 100 µM. For this reason, the numeric value of selectivity could not be precisely determined; however, all derivatives proved to be selective.

**Table 5:** Interaction of ketone-selenoesters with doxorubicin on MDR Colo 320 cells.

Compounds	Starting Conc. (µM)	Ratio *	CI at ED_50_	SD (±)	Type of Interaction
**K1**	5	0.6:1	2.6	0.73	Antagonism
		1.2:1	1.03	0.11	Additive effect
		2.4:1	0.94	0.09	Additive effect
		4.8:1	0.88	0.13	**Slight synergism**
		9.6:1	1.18	0.15	Slight antagonism
**K2**	6	0.7:1	1.77	0.22	Antagonism
		1.4:1	2.95	0.16	Antagonism
		2.8:1	1.2	0.22	Slight antagonism
		5.6:1	1.02	0.22	Additive effect
		11.2:1	1.5	0.27	Antagonism
		22.4:1	2.34	0.59	Antagonism
**K3**	6	0.7:1	1.32	0.8	Moderate antagonism
		1.4:1	0.37	0.15	**Synergism**
		2.8:1	0.73	0.1	**Moderate synergism**
		5.6:1	1.5	0.24	Antagonism
		11.2:1	1.3	0.07	Moderate antagonism
		22.4:1	1.72	0.08	Antagonism
**K4**	5	0.6:1	0.54	0.07	**Synergism**
		1.2:1	1.03	0.05	Additive effect
		2.4:1	1.1	0.05	Additive effect
		4.8:1	0.74	0.1	**Moderate synergism**
		9.6:1	0.85	0.06	**Slight synergism**
		19.2:1	0.97	0.12	Additive effect
**K5**	6	0.7:1	0.51	0.06	**Synergism**
		1.4:1	0.81	0.05	**Moderate synergism**
		2.8:1	0.55	0.04	**Synergism**
		5.6:1	0.58	0.02	**Synergism**
		11.2:1	0.64	0.02	**Synergism**
		22.4:1	0.68	0.06	**Synergism**
**K6**	6	0.7:1	0.51	0.06	**Synergism**
		1.4:1	0.81	0.05	**Moderate synergism**
		2.8:1	0.55	0.04	**Synergism**
		5.6:1	0.58	0.02	**Synergism**
		11.2:1	0.64	0.02	**Synergism**
		22.4:1	0.68	0.06	**Synergism**
**K7**	6	0.7:1	1.4	0.2	Moderate antagonism
		1.4:1	3.1	0.41	Antagonism
		2.8:1	1.36	0.2	Moderate antagonism
		5.6:1	1.3	0.07	Moderate antagonism
		11.2:1	2.8	0.15	Antagonism
		22.4:1	2.28	0.15	Antagonism
**K8**	6	0.7:1	0.12	0.09	**Strong synergism**
		1.4:1	2.4	0.62	Antagonism
		2.8:1	3.3	0.8	Antagonism
		5.6:1	2.01	0.97	Antagonism
		11.2:1	3.3	0.74	Antagonism

* Ratio: the applied combination and the concentration of selenoester–doxorubicin combination. CI at ED_50_: combination index value (CI) at the 50% growth inhibition dose (ED_50_). The most effective interactions (types of synergism) for each derivative are highlighted in bold.

**Table 6:** Interaction of cyanoselenoesters with doxorubicin on MDR Colo 320 colon adenocarcinoma cells.

Compounds	Starting Conc. (µM)	Ratio *	CI at ED_50_	SD (±)	Type of Interaction
**N1**	15	1.7:1	1.9	0.2	Antagonism
		3.4:1	0.34	0.04	**Synergism**
		6.8:1	0.51	0.04	**Synergism**
		13.6:1	0.95	0.15	Additive effect
		27.2:1	0.56	0.09	**Synergism**
		54.4:1	0.21	0.21	**Strong synergism**
		54.4:1	0.21	0.21	Strong synergism
**N2**	15	1.7:1	0.62	0.19	**Synergism**
		3.4:1	3.1	0.38	Antagonism
		6.8:1	0.58	0.03	**Synergism**
		13.6:1	1.36	0.05	Moderate antagonism
		27.2:1	2.8	0.12	Antagonism
		54.4:1	2.3	0.15	Antagonism
**N3**	10	1.2:1	1.7	0.44	Antagonism
		2.4:1	3.7	0.5	Strong antagonism
		4.8:1	0.85	0.1	**Moderate synergism**
		9.6:1	1.01	0.15	Additive effect
		19.2:1	1.19	0.17	Slight antagonism
		38.4:1	1.3	0.21	Moderate antagonism
**N4**	10	1.2:1	1.7	0.44	Antagonism
		2.4:1	3.7	0.5	Strong antagonism
		4.8:1	0.85	0.1	**Moderate synergism**
		9.6:1	1.01	0.15	Additive effect
		19.2:1	1.19	0.17	Slight antagonism
		38.4:1	1.3	0.21	Moderate antagonism
**N5**	10	1.2:1	5.7	1.6	Strong antagonism
		2.4:1	3.7	0.5	Strong antagonism
		4.8:1	1.2	0.95	Slight antagonism
		9.6:1	1.01	0.15	Additive effect
		19.2:1	1.19	0.17	Slight antagonism
		38.4:1	1.3	0.21	Moderate antagonism
**N6**	10	1.2:1	2.9	0.31	Antagonism
		2.4:1	4.5	0.5	Additive effect
		4.8:1	1.5	0.1	Antagonism
		9.6:1	1.78	0.21	Antagonism
		19.2:1	1.96	0.11	Antagonism
		38.4:1	1.96	0.105	Antagonism
**N7**	8	0.9:1	2.7	0.23	Antagonism
		1.8:1	5.3	1.02	Strong antagonism
		3.6:1	0.9	0.23	**Slight synergism**
		7.2:1	1.84	0.18	Antagonism
		14.4:1	1.89	0.29	Antagonism
		28.8:1	0.92	0.6	Additive effect

* Ratio: the applied combination and the concentration of selenocompound–doxorubicin. CI at ED_50_: combination index value (CI) at the 50% growth inhibition dose (ED_50_). The most effective interactions (types of synergism) for each derivative are highlighted in bold.

**Table 7:** Apoptosis induction by ketone-selenoesters on MDR Colo 320 adenocarcinoma cells. The 12*H*- benzo(α)phenothiazine (M627) was applied as a positive control.

	Conc. (μM)	Early Apoptosis (%)	Late Apoptosis (%)	Cell Death (%)	Total Apoptotic Events (Early + Late, %)	Apoptosis Quotient (%)
		Treated Sample-Untreated Sample	Treated Sample-Untreated Sample	Treated Sample-Untreated Sample		
**M627**	20	13.3	36.1	0.00	49.4	100%
**K1**	2	3.7	28.9	2.15	32.6	66.0%
**K2**	2	0.3	33.9	0.95	34.2	69.2%
**K3**	2	**18.6**	25.6	0.00	44.2	89.5%
**K4**	2	3.4	16.1	5.95	19.5	39.5%
**K5**	2	1.8	30.9	1.35	32.7	66.2%
**K6**	2	4.5	27.8	0.17	32.3	65.4%
**K7**	2	6.1	27.5	0.10	33.6	68.0%
**K8**	2	4.9	26.7	0.85	31.6	64.0%

Apoptotic quotient is defined as the quotient of the total apoptotic events determined for the respective compound and the reference M627, expressed in percentage. The most effective early apoptosis induction is highlighted in bold.

**Table 8:** Wound healing activity determined as keratinocytes’ (HaCaT) wound closure (%) after 24 and 48 h. Allantoin (50 µg/mL) served as a positive control. Data represent the average of twenty repetitions (four biological, five technical) with corresponding standard error of the mean. The data were analysed with *t*-test, where the difference between the group and negative control was considered statistically significant when *p* < 0.0000005 (******), 0.000005 (*****), 0.00005 (****), 0.0005 (***), 0.005 (**), and 0.05 (*).

	Dose	24 h			48 h		
Compound	IC_10_ (µM)	Closure (%)	SEM	*t*-Test	Closure (%)	SEM	*t*-Test
**K1**	0.30	83.64	1.03	******	100.00		******
**K2**	0.20	85.90	0.97	******	100.00		******
**K3**	0.24	87.49	1.92	******	100.00		******
**K4**	0.46	79.17	1.81	******	86.29	2.47	*****
**K5**	0.25	88.56	1.44	******	100.00		******
**K6**	0.22	83.02	1.54	******	92.65	1.34	******
**K7**	0.24	82.71	1.22	******	100.00		******
**K8**	0.17	88.55	1.84	******	100.00		******
**N1**	0.40	75.53	1.52	******	85.41	1.63	******
**N2**	0.36	72.23	1.20	******	77.51	1.98	*
**N3**	0.64	46.46	2.00	******	75.70	1.44	
**N4**	0.20	73.65	1.92	******	83.84	2.09	******
**N5**	0.60	53.25	1.86	**	64.21	2.78	*****
**N6**	0.60	46.13	1.25	******	60.98	3.13	*****
**N7**	0.61	74.84	1.77	******	82.41	1.50	******
**PC**		70.37	2.56	******	84.63	2.21	******
**NC**		56.33	1.75		75.55	1.71	

SEM, standard error of the mean; *t*-test, 2-tailed *t*-test with unequal variances.

## Data Availability

The data presented in this study are available in this article (and supplementary material). Additional data are available on request from the corresponding author.

## Supplementary Materials

The following are available online at https://www.mdpi.com/article/10.3390/cancers13184563/s1. Figure S1: Compound **K1**: Se-(2-oxopropyl) thiophene-2-carboselenoate, Figure S2: Compound **K2**: Se-(2-oxopropyl) 2-fluorobenzoselenoate, Figure **S3**: Compound K3: Se-(2-oxopropyl) 4-bromobenzoselenoate, Figure S4: Compound **K4**: Se-(2-oxopropyl) 2-(trifluoromethyl)benzoselenoate, Figure S5: Compound **K5**: Se-(2-oxopropyl) 3-(trifluoromethyl)benzoselenoate, Figure S6: Compound **K6**: Se-(2-oxopropyl) 3-chloro-4-fluorobenzoselenoate, Figure S7: Compound **K7**: Se-(2-oxopropyl) 4-(tert-butyl)benzoselenoate, Figure S8: Compound **K8**: Se-(2-oxopropyl) 2,4,5-trifluorobenzoselenoate, Figure S9: Compound **N1**: Se-(cyanomethyl) thiophene-2-carboselenoate, Figure S10: Compound **N2**: Se-(cyanomethyl) 3-fluorobenzoselenoate, Figure S11: Compound **N3**: Se-(cyanomethyl) 4-bromobenzoselenoate, Figure S12: Compound **N4**: Se-(cyanomethyl) 2-(trifluoromethyl)benzoselenoate, Figure S13. Compound **N5**: Se-(cyanomethyl) 3-(trifluoromethyl)benzoselenoate, Figure S14. Compound **N6**: Se-(cyanomethyl) 3-chloro-4-fluorobenzoselenoate, Figure S15. Compound **N7**: Se-(cyanomethyl) 3,5-bis(trifluoromethyl)benzoselenoate.
