# Supplementary material for: Cyano- and Ketone-Containing Selenoesters as Multi-Target Compounds against Resistant Cancers
Source: Cancers (Basel). 2021 Sep 11;13(18):4563. doi: 10.3390/cancers13184563 (PMC8465942; doi:10.3390/cancers13184563)
Supplement: Supplementary file 1 [file cancers-13-04563-s001.zip › cancers-1316675-supplementary.pdf]

cancers

Supplementary Materials: Cyano- and ketone-containing selenoesters as multi-target compounds against resistant cancers

Nikoletta Szemerédi, Simona Dobiasová, Noemi Salardón-Jiménez, Annamária Kincses, Márta Nové, Giyaullah Habibullah, Clotilde Sevilla-Hernández, Miguel Benito-Lama, Francisco-Javier Alonso-Martínez, Jitka Viktorová, Gabriella Spengler and Enrique Domínguez-Álvarez

Contents

All compounds have FTIR, MS-DIP, ^{1}H-NMR and ^{13}C-NMR spectra. Zooms of the aromatic region of ^{1}H-NMR have been added for all compounds, and when necessary, also zooms of the aromatic peaks in ^{13}C-NMR are provided. Bidimensional COSY, HSQC and HMBC have been provided for selected compounds.

Spectra of the ketone selenoesters (K1-K8) and methylcyano selenoesters (N1-N7). Page 2
S1. Compound **K1**: *Se*-(2-oxopropyl) thiophene-2-carboselenoate. Page 2
S2. Compound **K2**: *Se*-(2-oxopropyl) 2-fluorobenzoselenoate. Page 6
S3. Compound **K3**: *Se*-(2-oxopropyl) 4-bromobenzoselenoate. Page 9
S4. Compound **K4**: *Se*-(2-oxopropyl) 2-(trifluoromethyl)benzoselenoate. Page 13
S5. Compound **K5**: *Se*-(2-oxopropyl) 3-(trifluoromethyl)benzoselenoate. Page 16
S6. Compound **K6**: *Se*-(2-oxopropyl) 3-chloro-4-fluorobenzoselenoate. Page 19
S7. Compound **K7**: *Se*-(2-oxopropyl) 4-(*tert*-butyl)benzoselenoate. Page 22
S8. Compound **K8**: *Se*-(2-oxopropyl) 2,4,5-trifluorobenzoselenoate. Page 26
S9. Compound **N1**: *Se*-(cyanomethyl) thiophene-2-carboselenoate. Page 31
S10. Compound **N2**: *Se*-(cyanomethyl) 3-fluorobenzoselenoate. Page 35
S11. Compound **N3**: *Se*-(cyanomethyl) 4-bromobenzoselenoate. Page 38
S12. Compound **N4**: *Se*-(cyanomethyl) 2-(trifluoromethyl)benzoselenoate. Page 41
S13. Compound **N5**: *Se*-(cyanomethyl) 3-(trifluoromethyl)benzoselenoate. Page 44
S14. Compound **N6**: *Se*-(cyanomethyl) 3-chloro-4-fluorobenzoselenoate. Page 49
S15. Compound **N7**: *Se*-(cyanomethyl) 4-(*tert*-butyl)benzoselenoate. Page 52

Spectra of the ketone selenoesters (K1-K8) and methylcyano selenoesters (N1-N7).

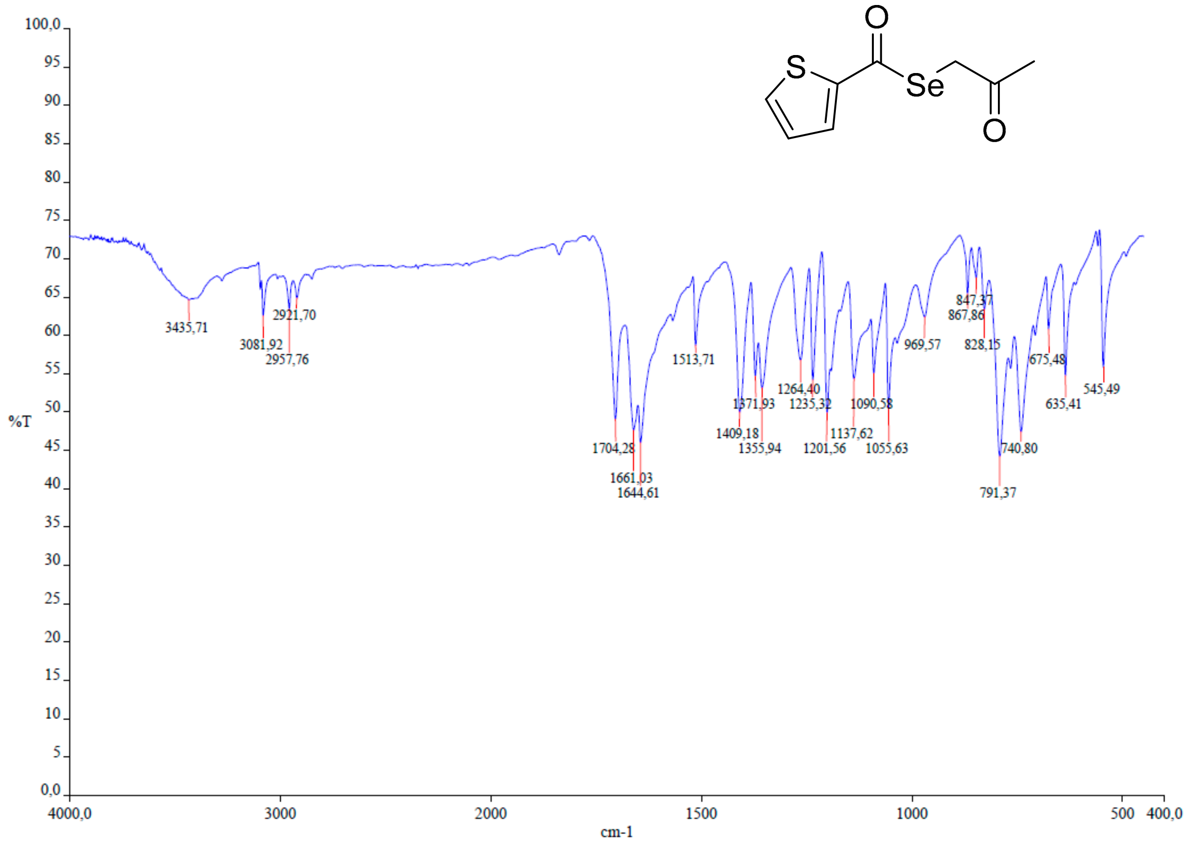

Figure S1. Compound **K1**: Se-(2-oxopropyl) thiophene-2-carboselenoate. S1**A**. IR spectrum (KBr) of **K1**.

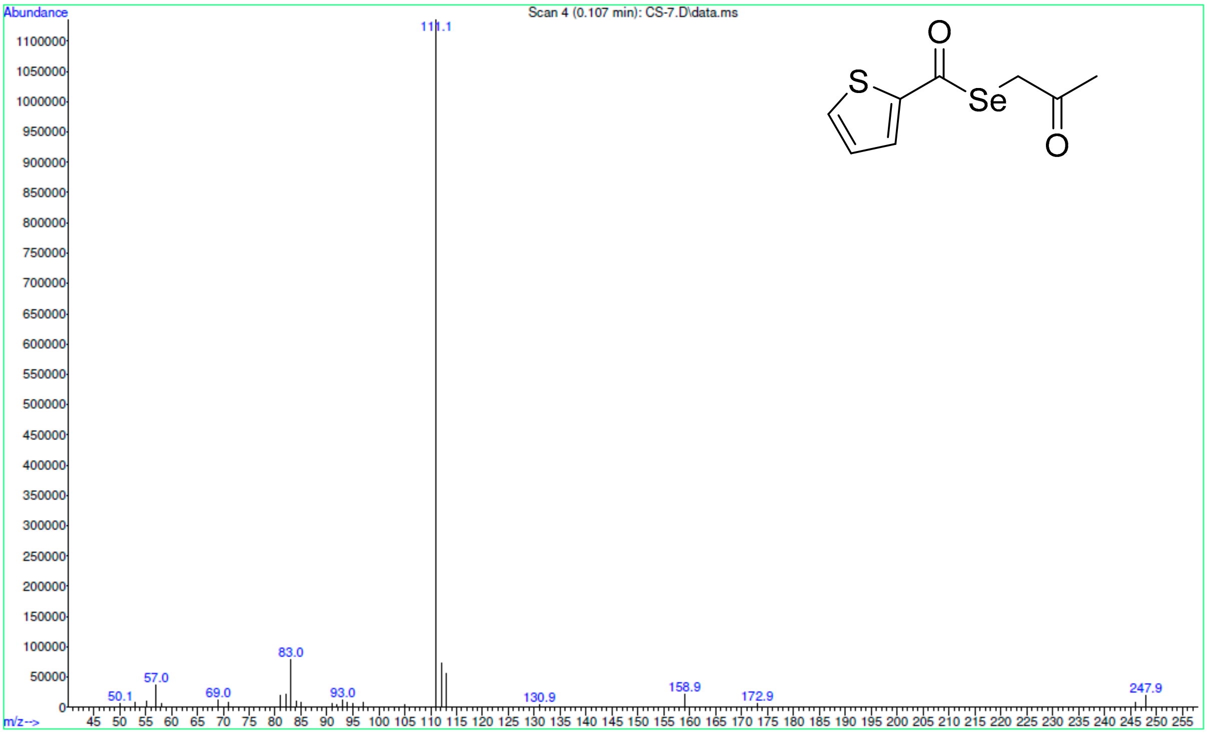

Figure S1B. DIP-MS spectrum of **K1**.

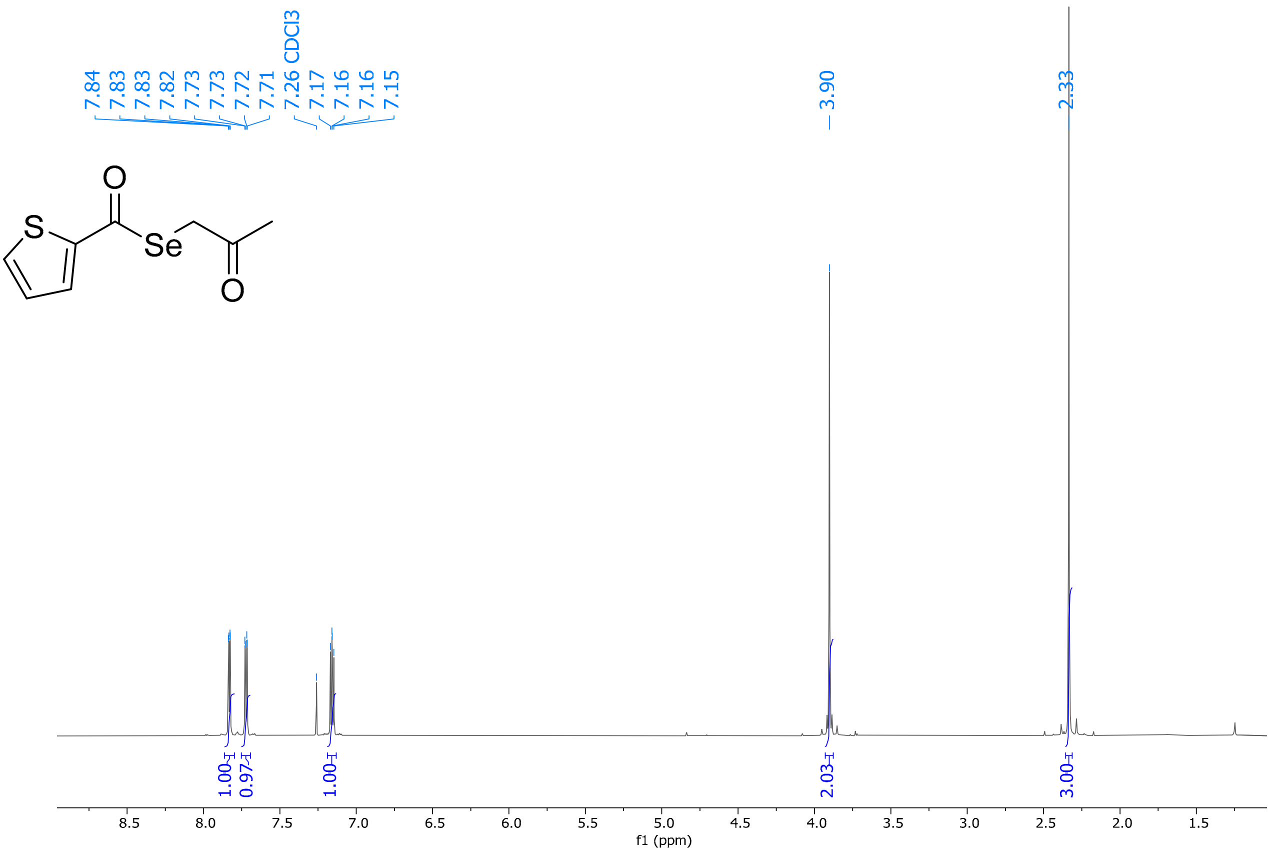

Figure S1C. ^{1}H-NMR spectrum ($CDCl_3$, 400 MHz) of **K1**.

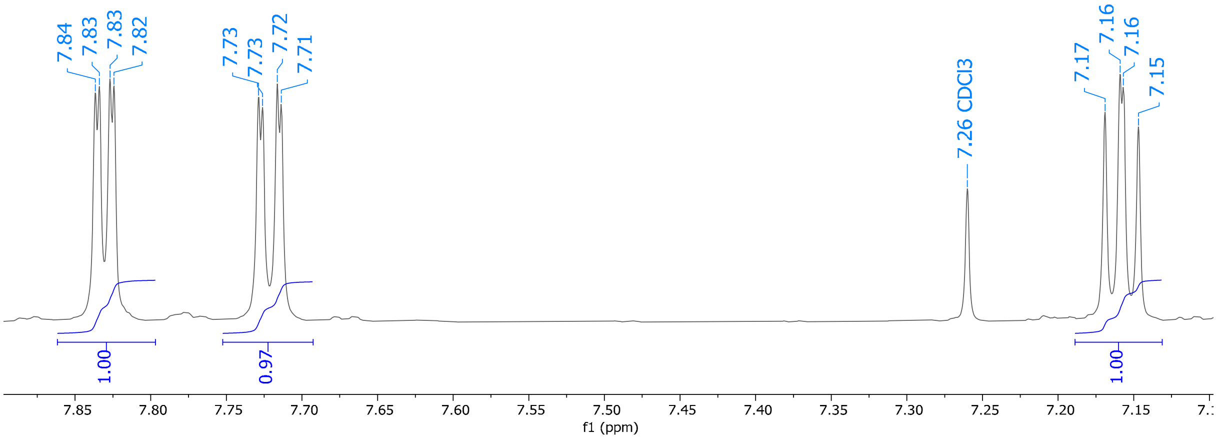

Figure S1D. ^{1}H-NMR spectrum ($CDCl_3$, 400 MHz) of **K1** (aromatics).

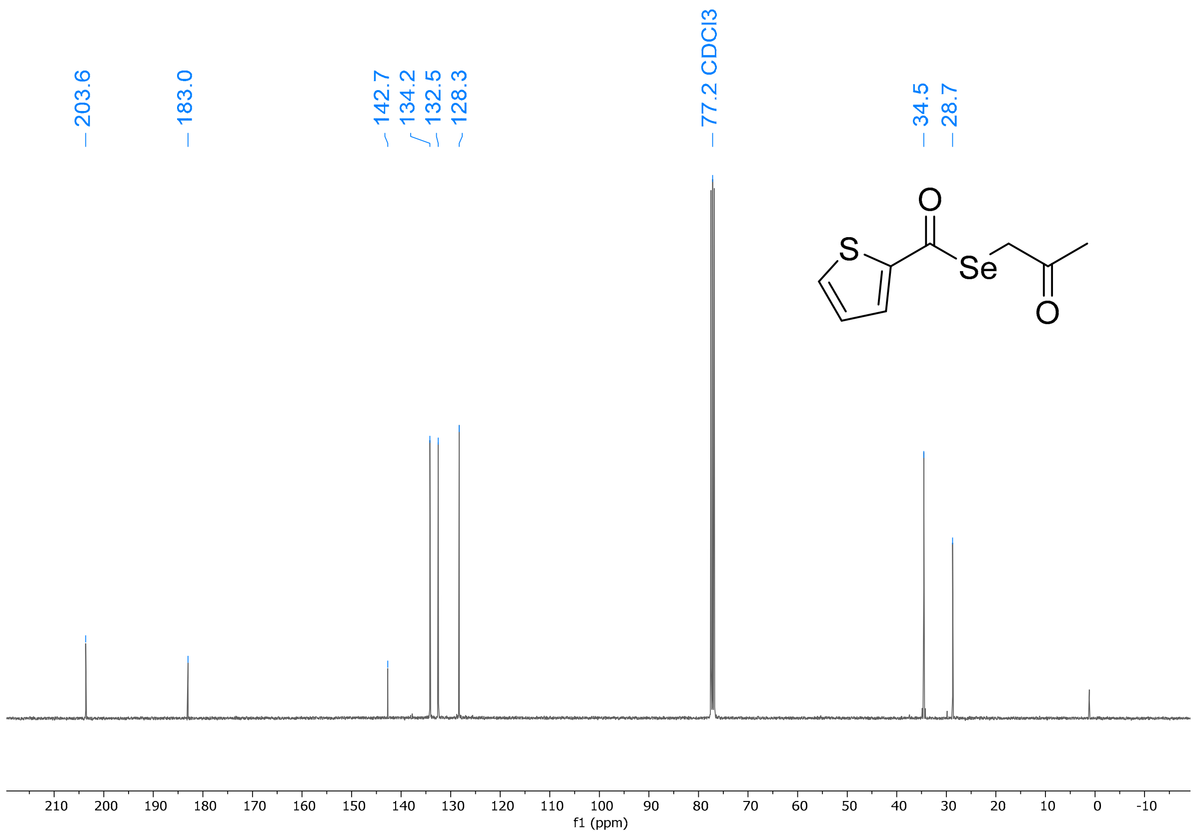

Figure S1E. ^{13}C-NMR spectrum ($CDCl_3$, 101 MHz) of **K1.**

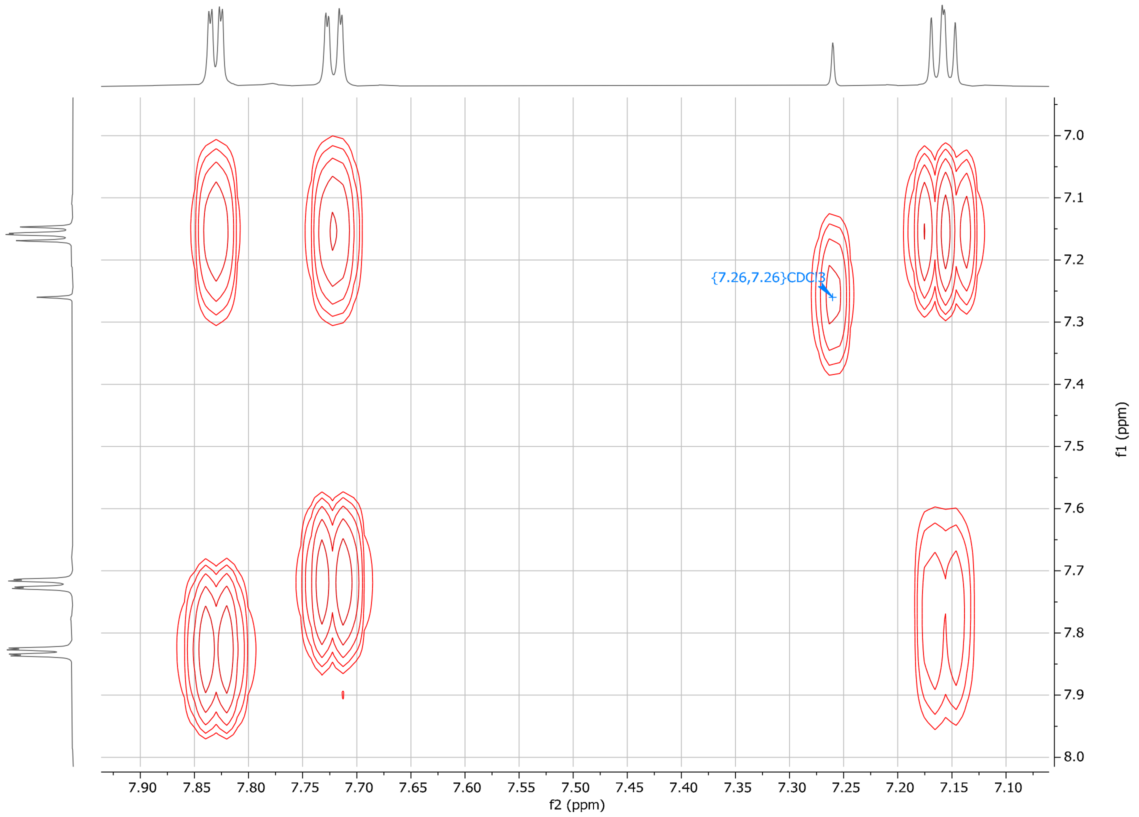

Figure S1F. ^{1}H-^{1}H COSY NMR spectrum ($CDCl_3$) of **K1** (aromatics).

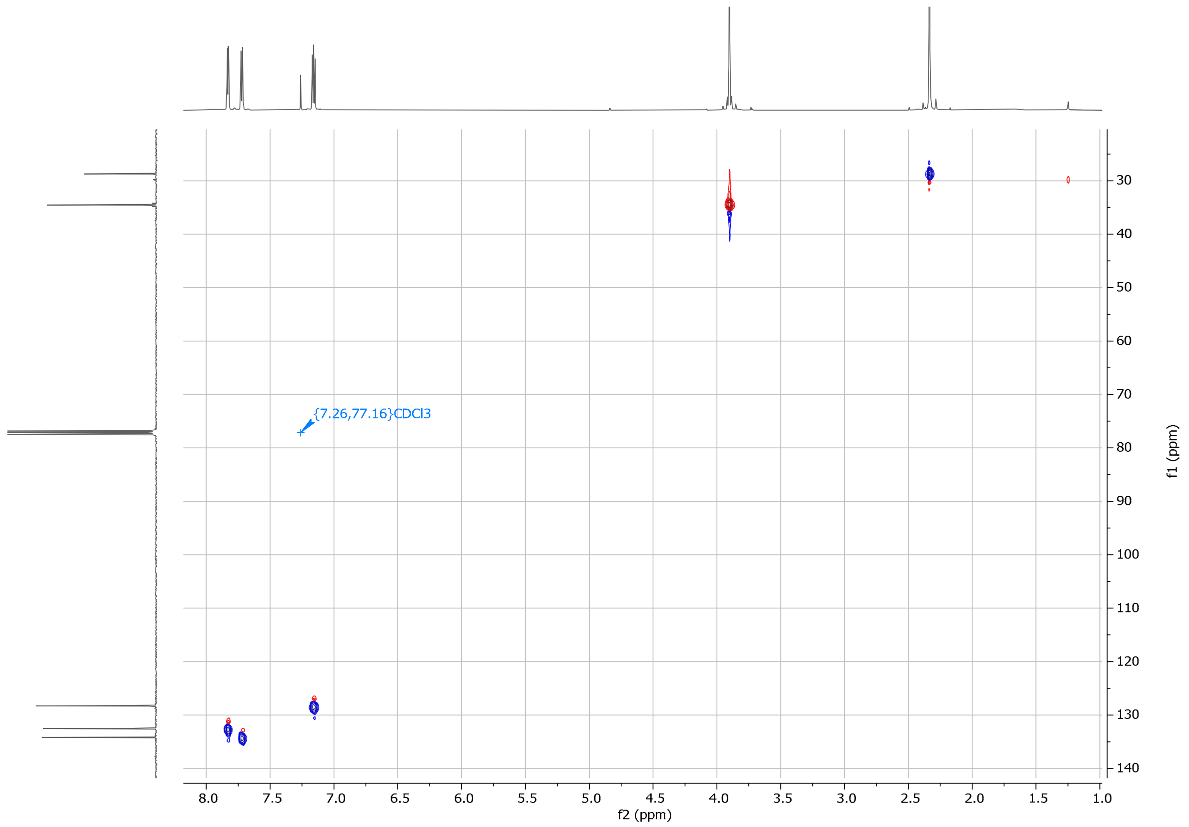

Figure S1G. ^{1}H-^{13}C HSQC NMR spectrum ($CDCl_3$) of **K1**.

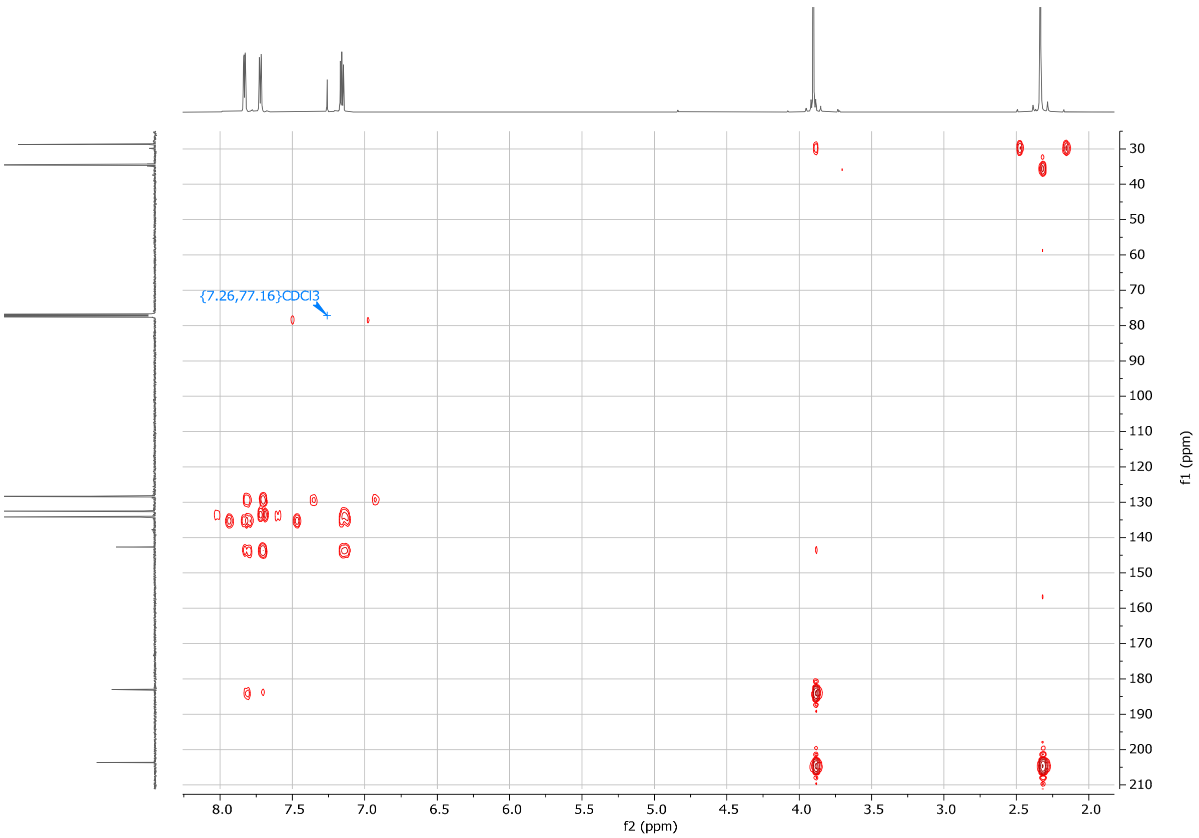

Figure S1H. ^{1}H-^{13}C HMBC NMR spectrum ($CDCl_3$) of **K1**.

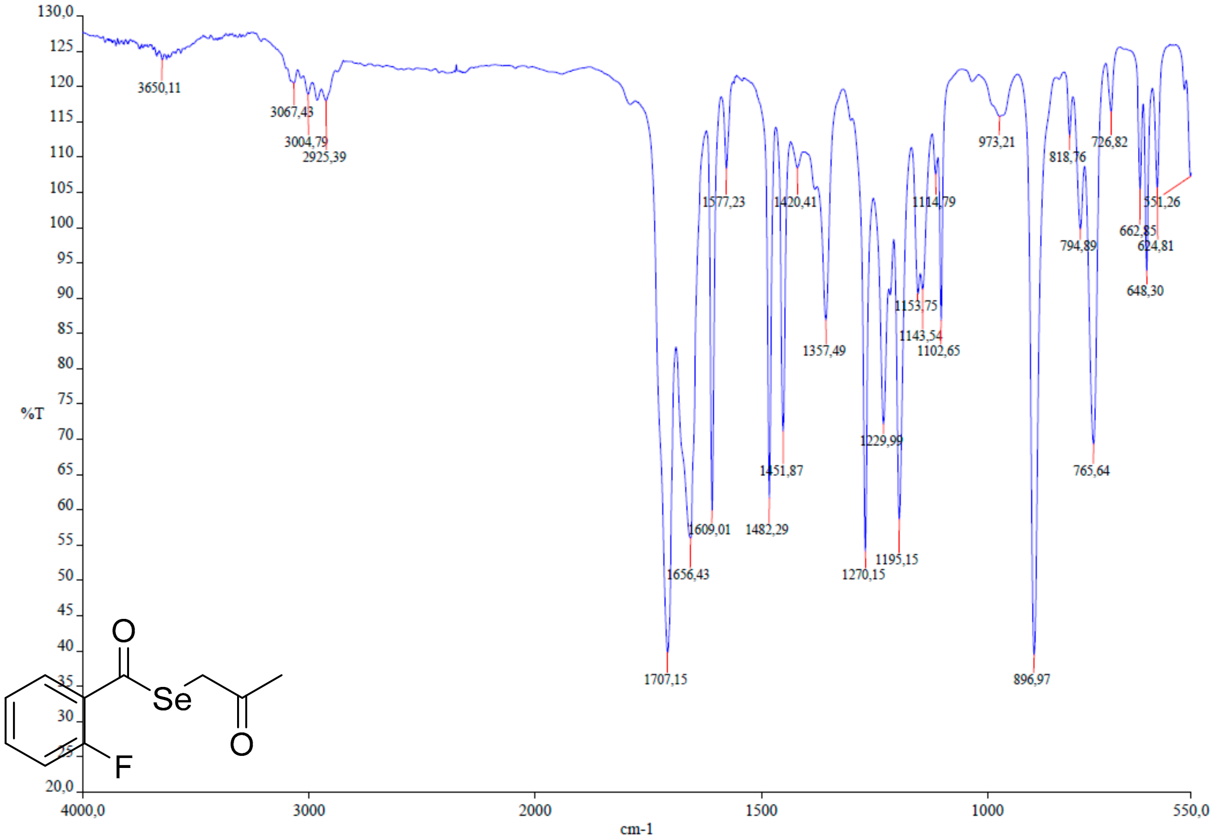

Figure S2. Compound **K2**: Se-(2-oxopropyl) 2-fluorobenzoselenoate. S2**A**. IR spectrum (NaCl) of **K2**.

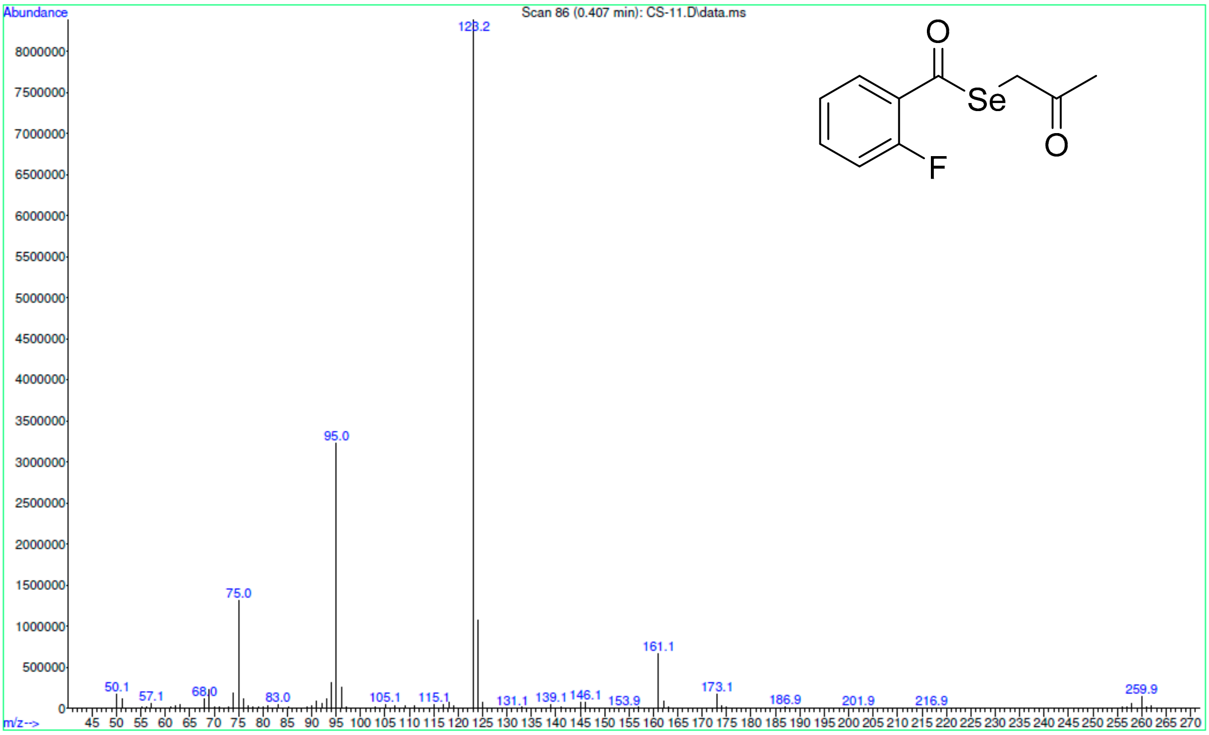

Figure S2B. DIP-MS spectrum of **K2**.

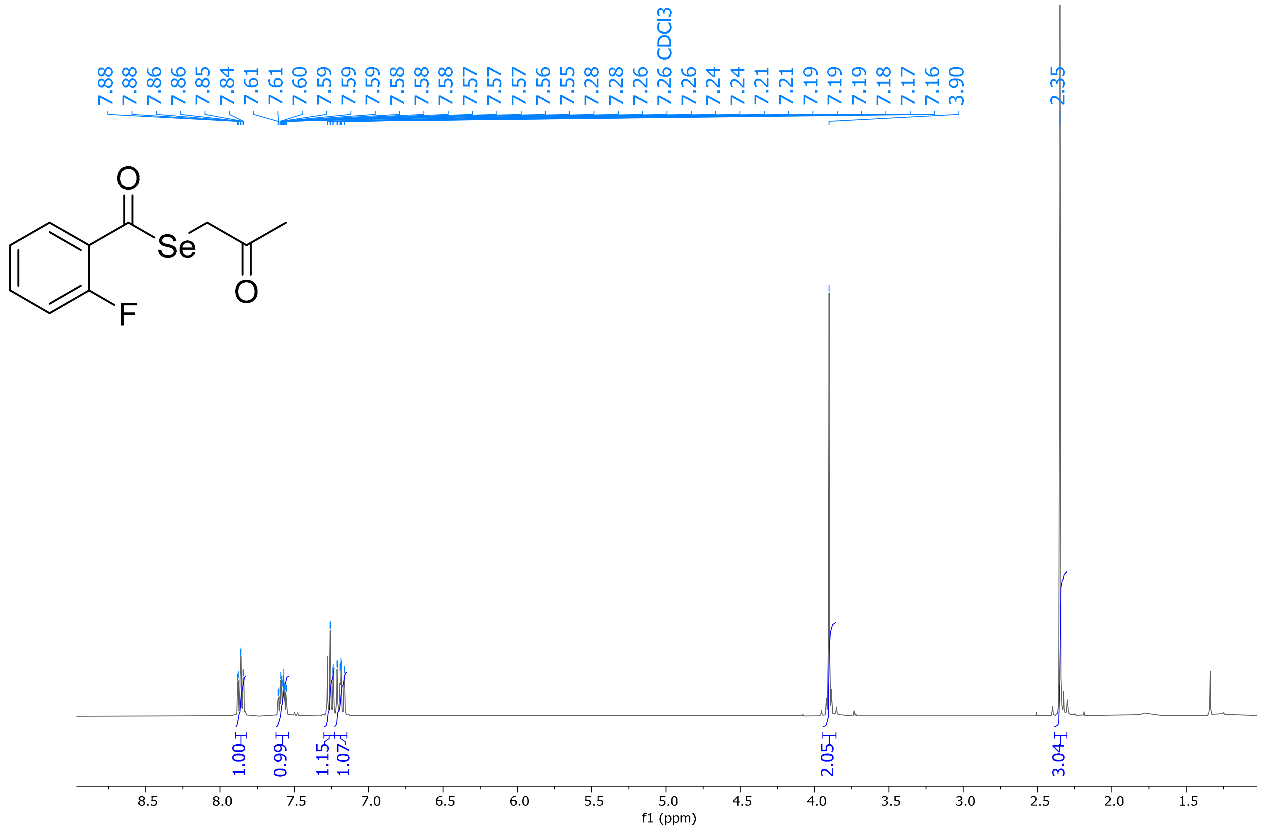

Figure S2C. ^{1}H-NMR spectrum ($CDCl_3$, 400 MHz) of **K2**.

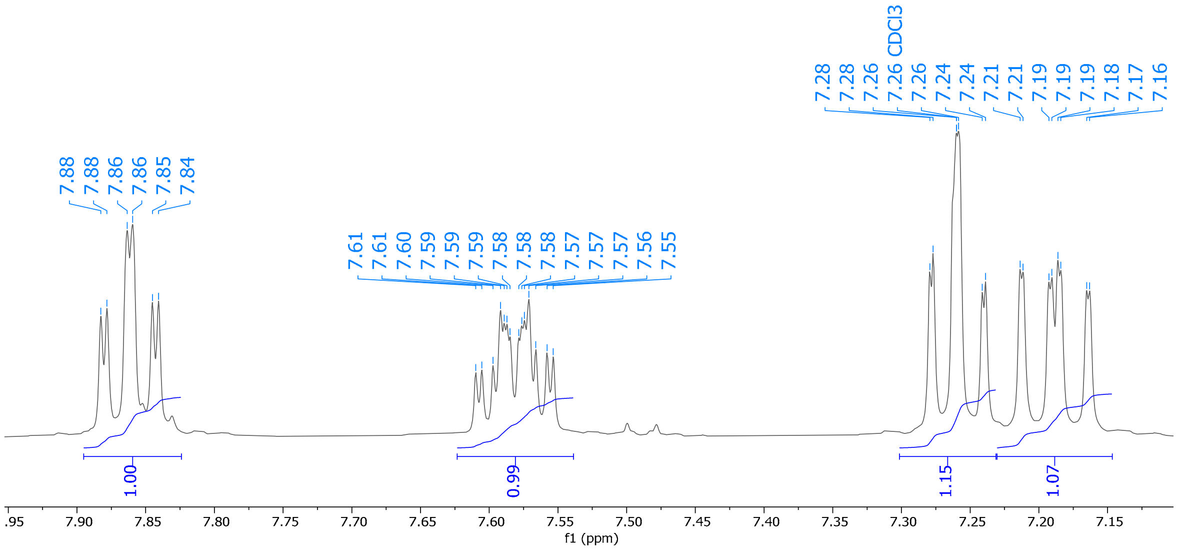

Figure S2D. ^{1}H-NMR spectrum ($CDCl_3$, 400 MHz) of **K2** (aromatics).

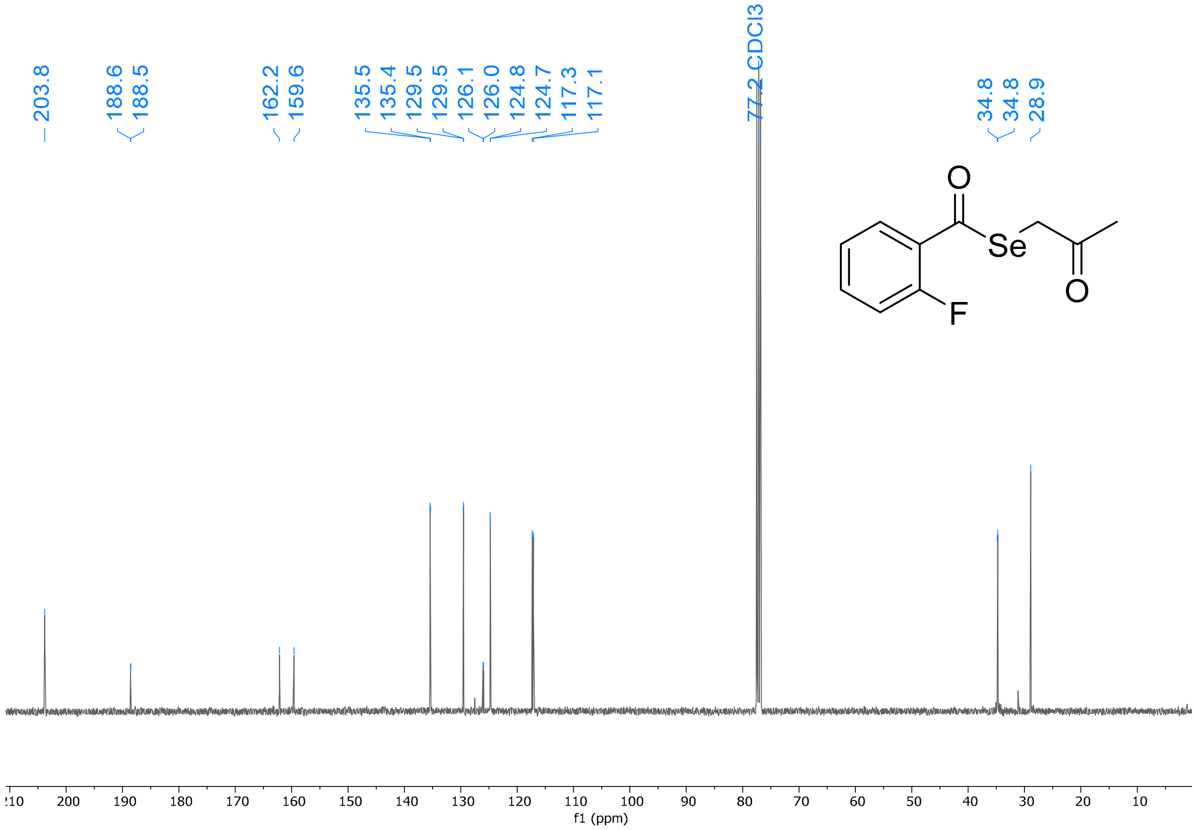

Figure S2E. ^{13}C-NMR spectrum ($CDCl_3$, 101 MHz) of **K2**.

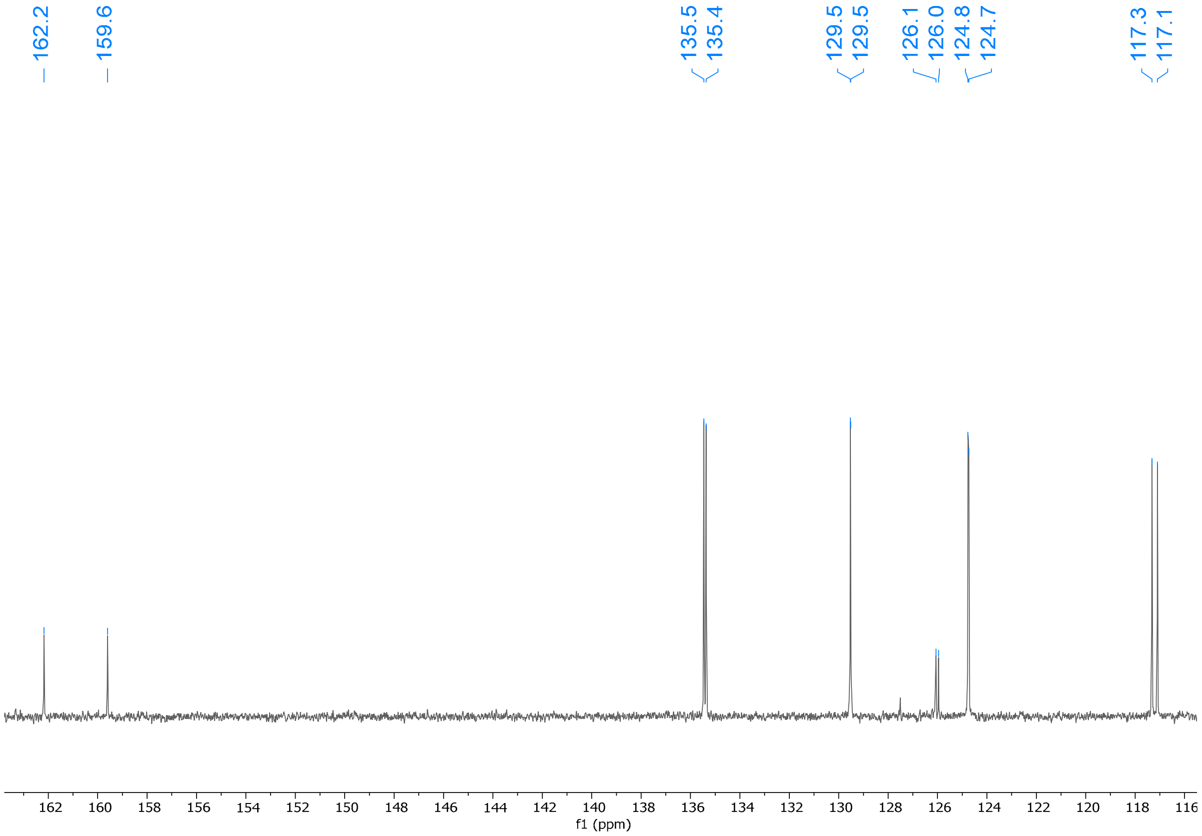

Figure S2F. ^{13}C-NMR spectrum ($CDCl_3$, 101 MHz) of **K2** (aromatics).

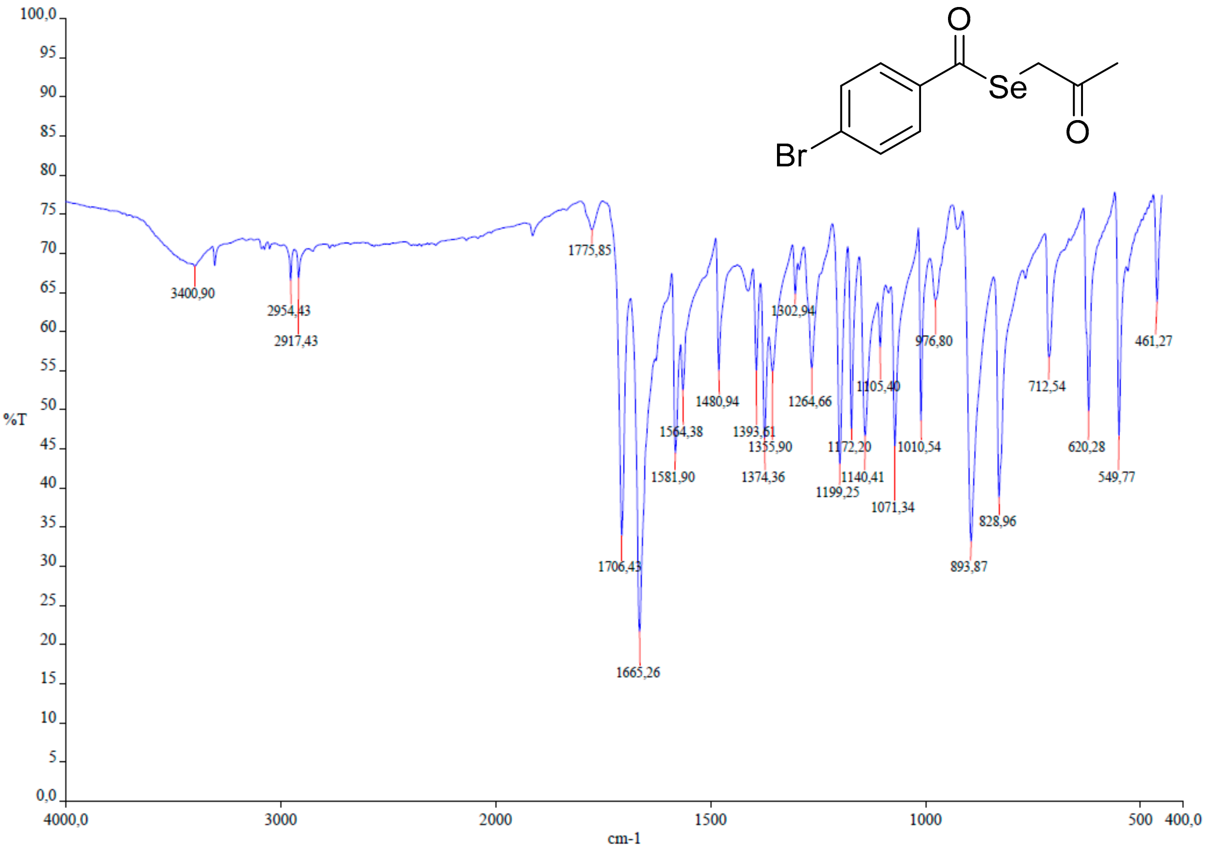

Figure S3. Compound **K3**: Se-(2-oxopropyl) 4-bromobenzoselenoate. S3**A**. IR spectrum (KBr) of **K3**.

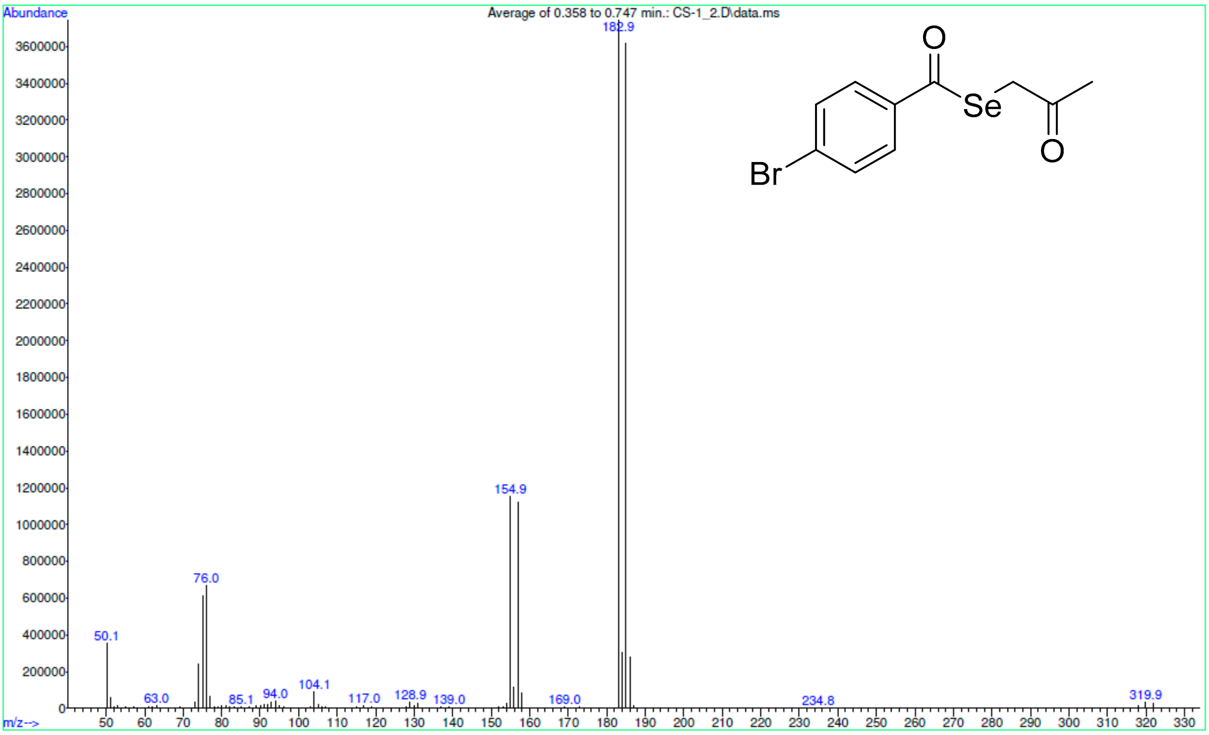

Figure S3B. DIP-MS spectrum of **K3**.

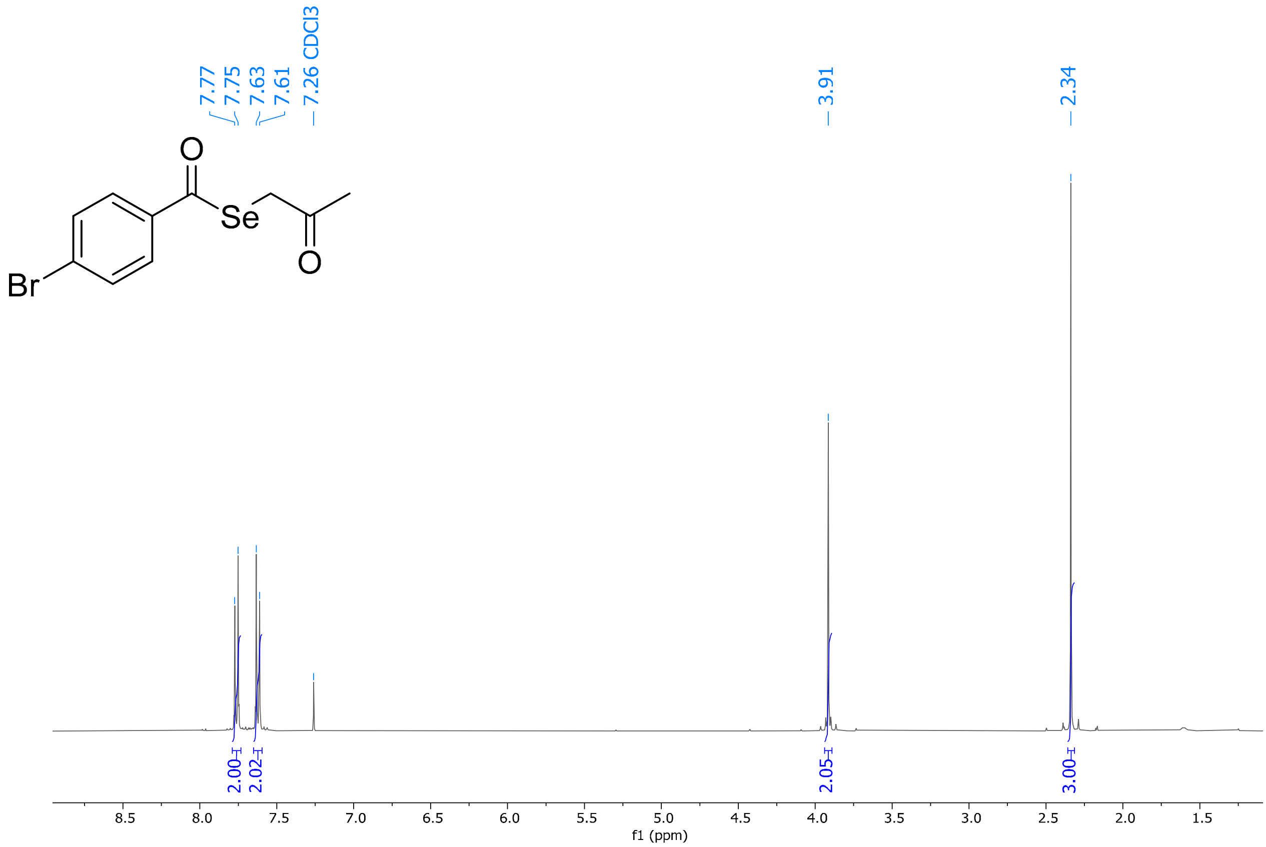

Figure S3C. ^{1}H-NMR spectrum ($CDCl_3$, 400 MHz) of **K3**.

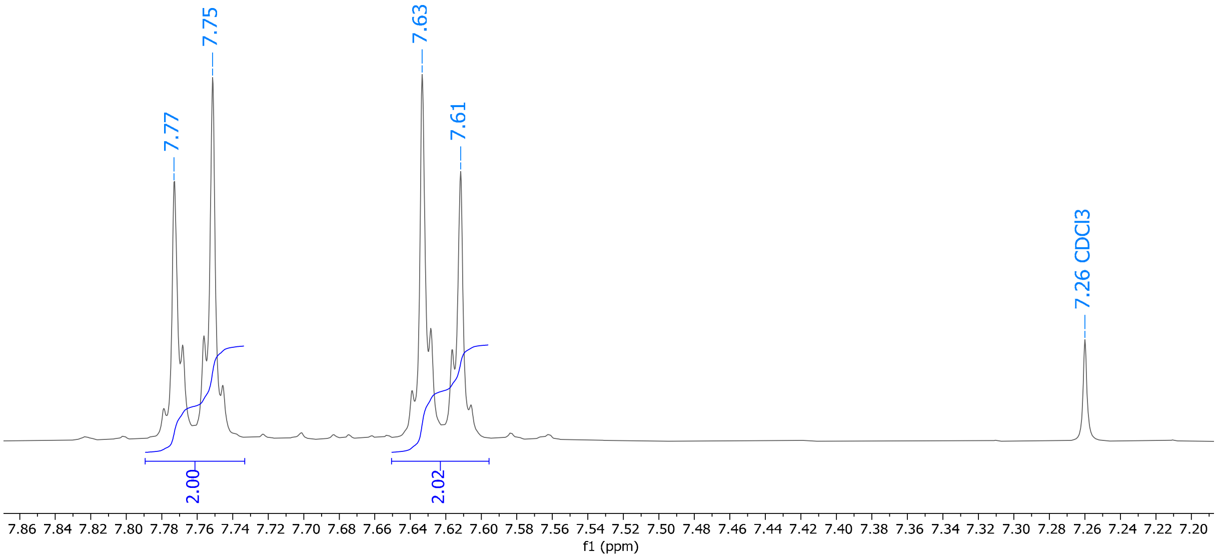

Figure S3D. ^{1}H-NMR spectrum ($CDCl_3$, 400 MHz) of K3 (aromatics).

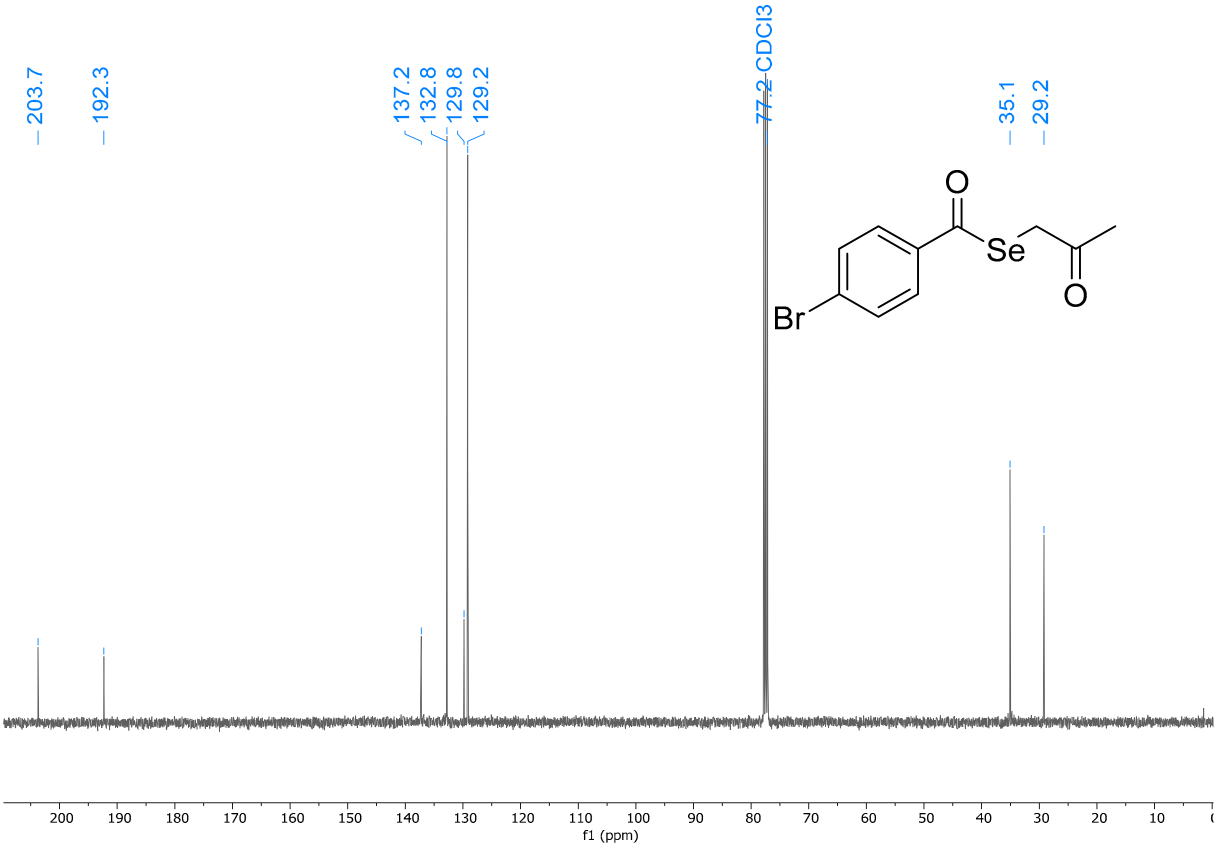

Figure S3E. ^{13}C-NMR spectrum ($CDCl_3$, 101 MHz) of **K3**.

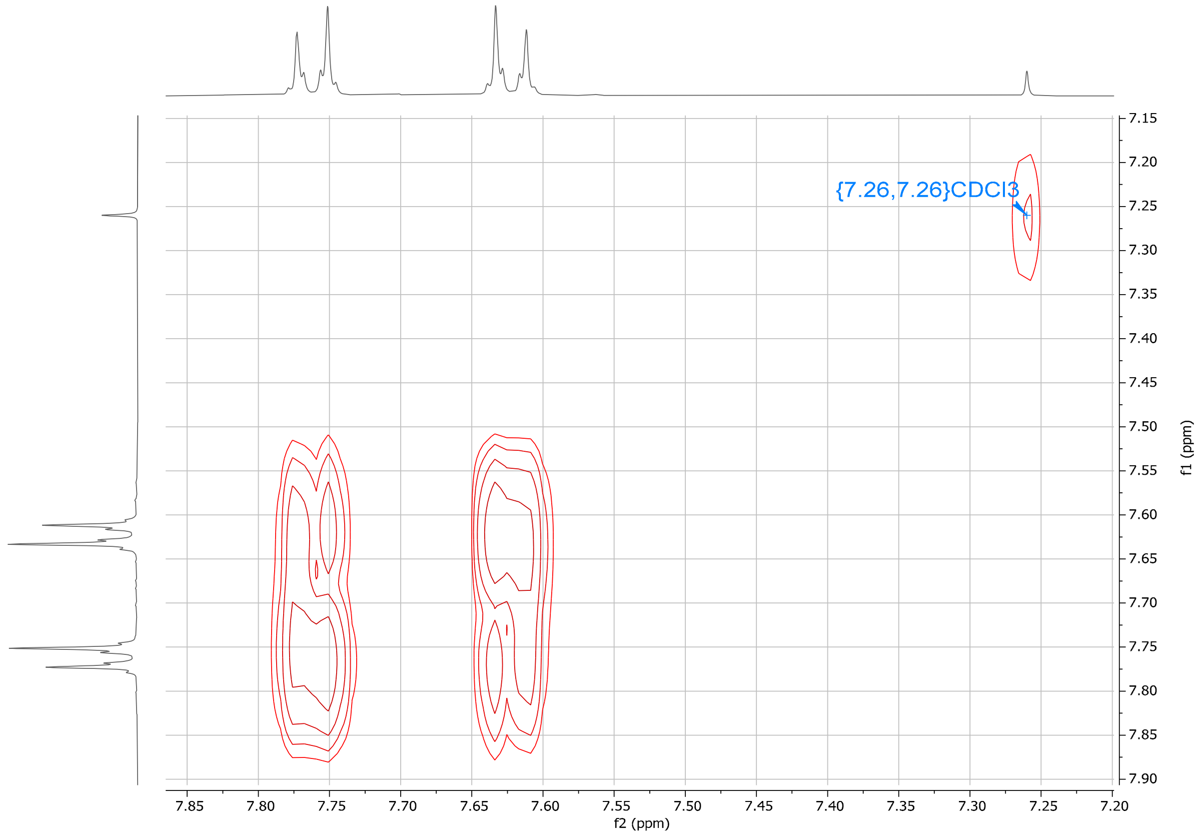

Figure S3F. ^{1}H-^{1}H COSY NMR spectrum ($CDCl_3$) of **K3** (aromatics).

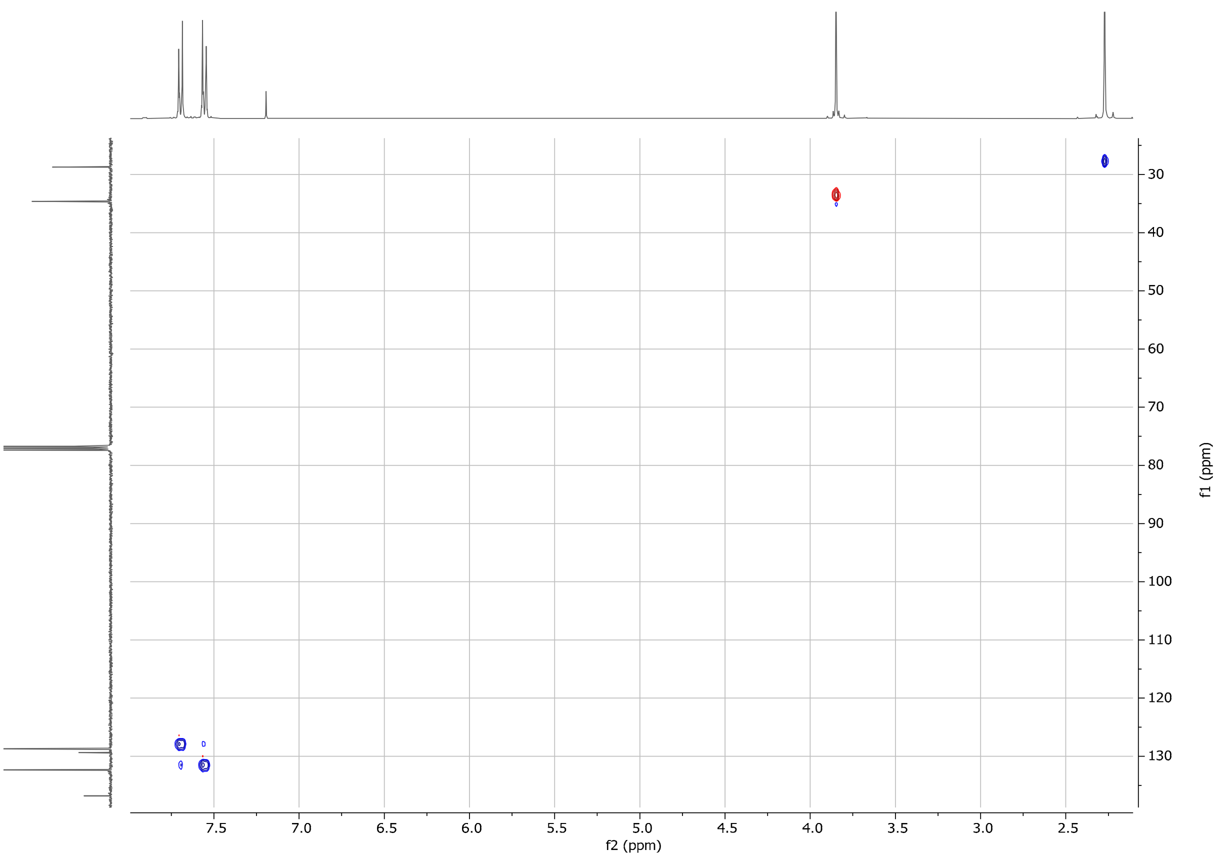

Figure S3G. ^{1}H-^{13}C HSQC NMR spectrum ($CDCl_3$) of **K3.**

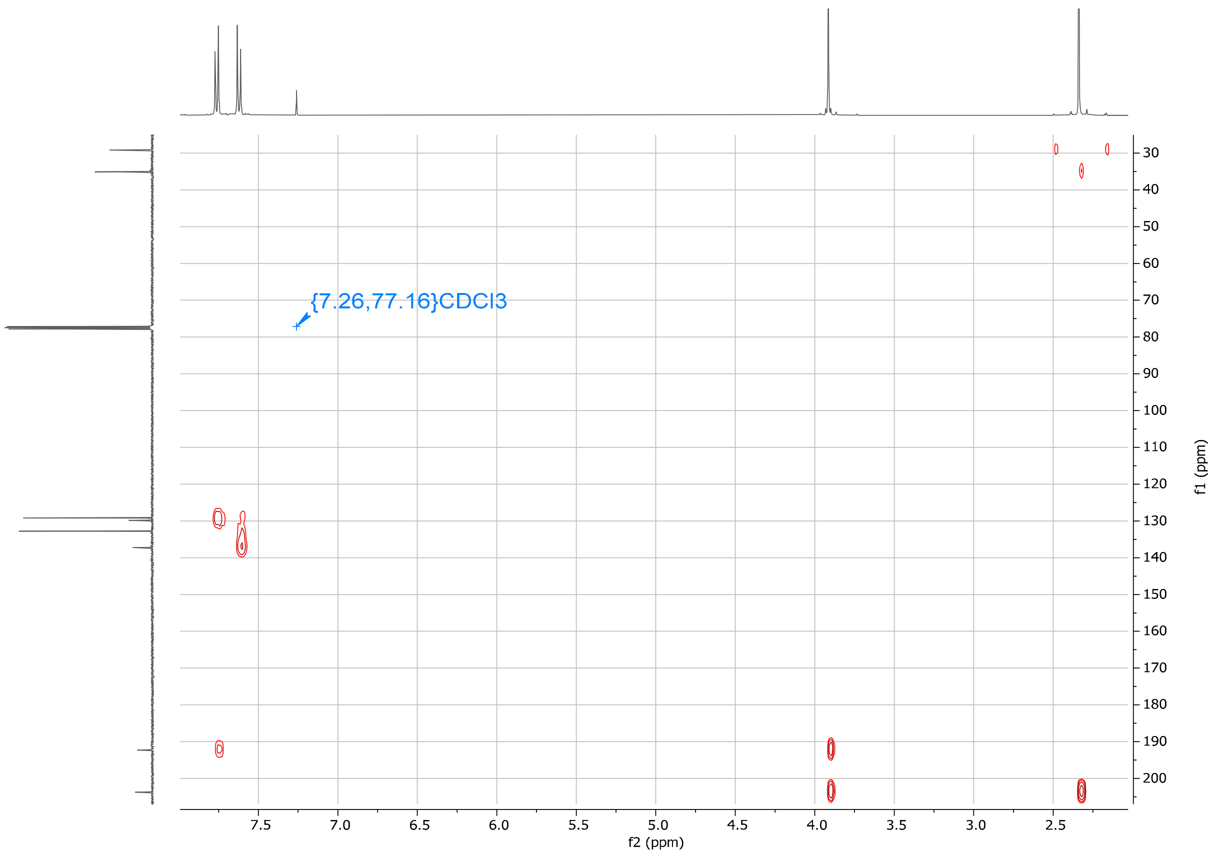

Figure S3H. ^{1}H-^{13}C HMBC NMR spectrum ($CDCl_3$) of **K3**.

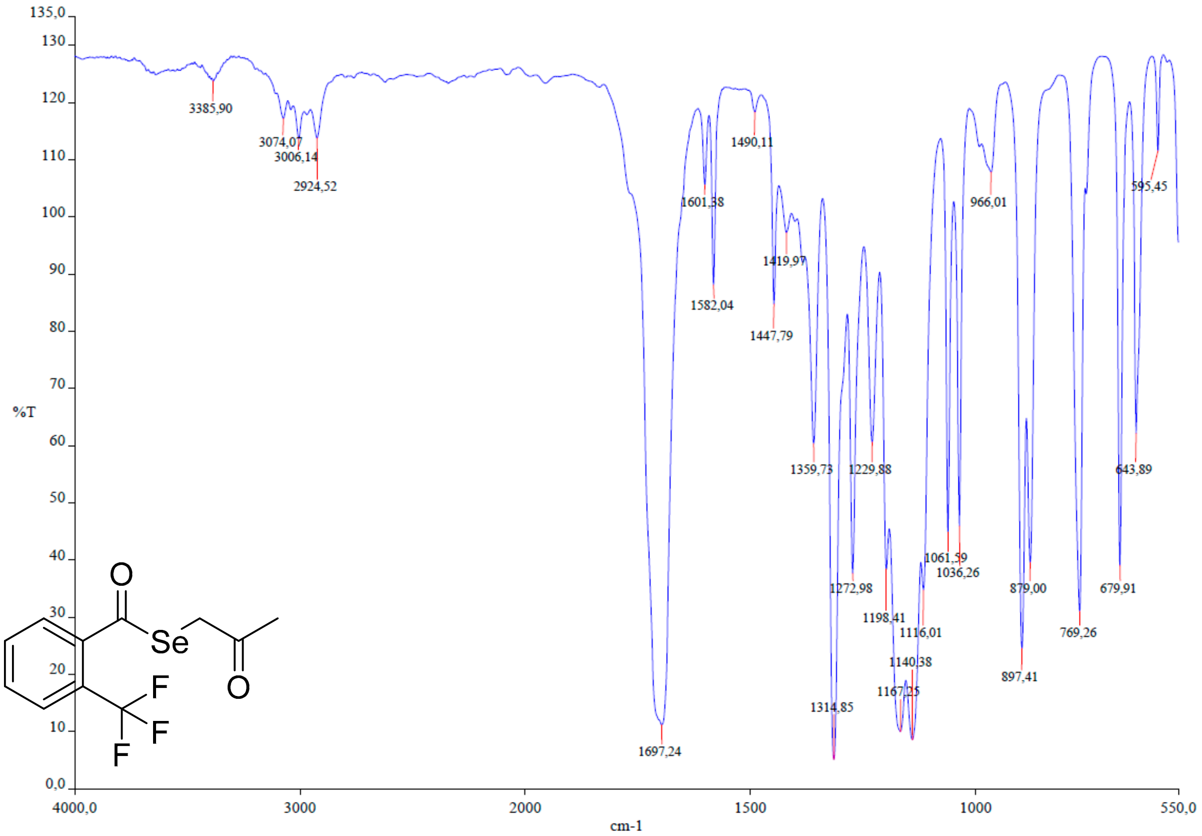

Figure S4. Compound **K4**: Se-(2-oxopropyl) 2-(trifluoromethyl)benzoselenoate. S4A. IR spectrum (NaCl) of **K4**.

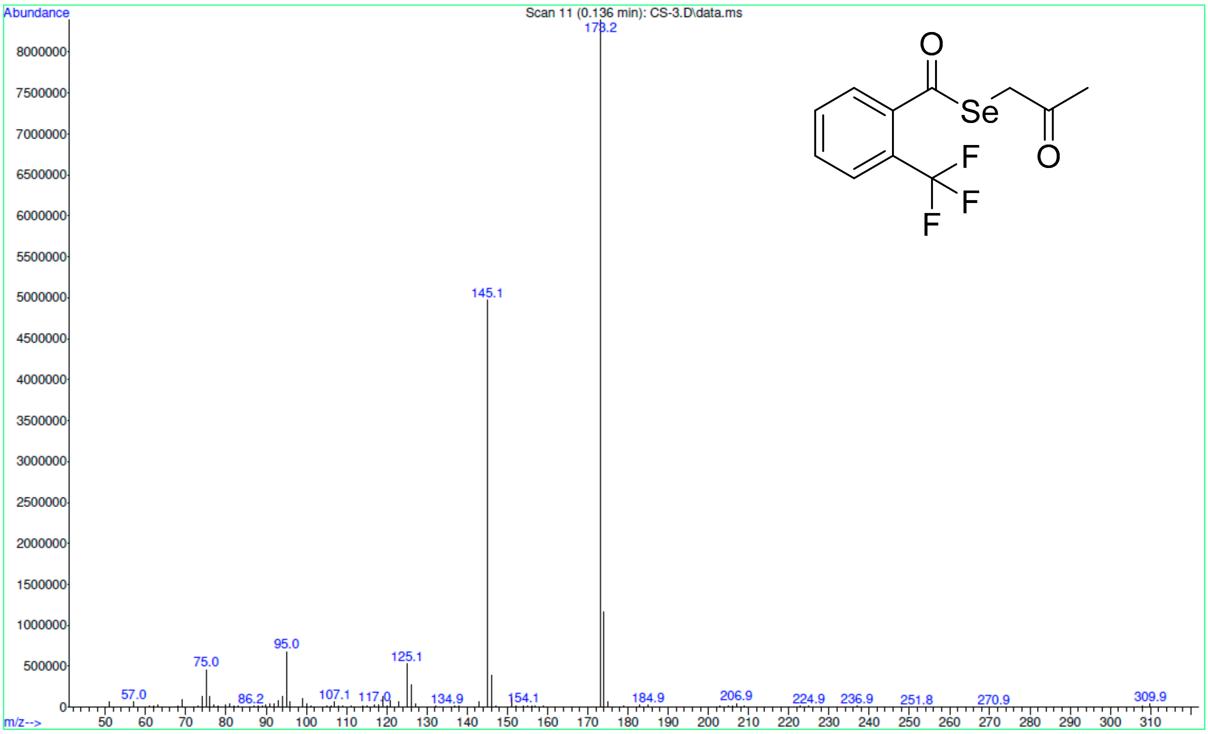

Figure S4B. DIP-MS spectrum of **K4**.

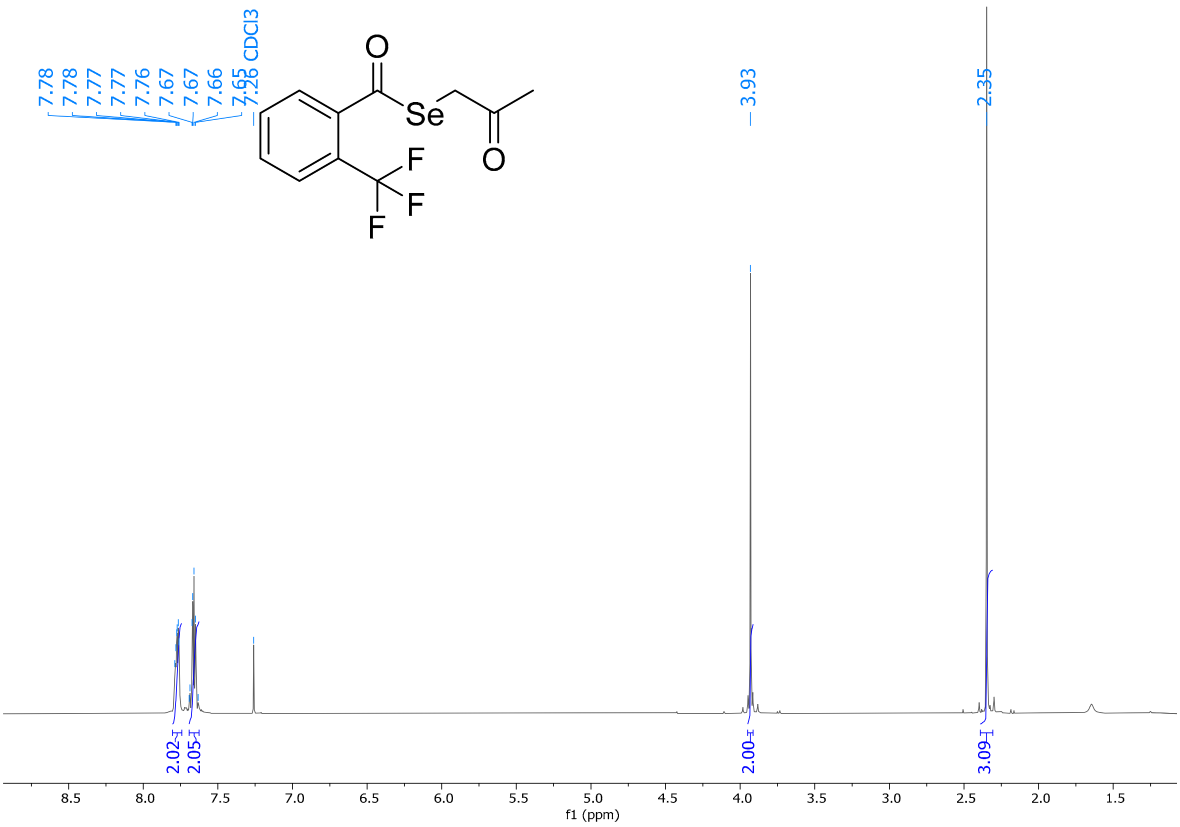

Figure S4C. ^{1}H-NMR spectrum ($CDCl_3$, 400 MHz) of **K4**.

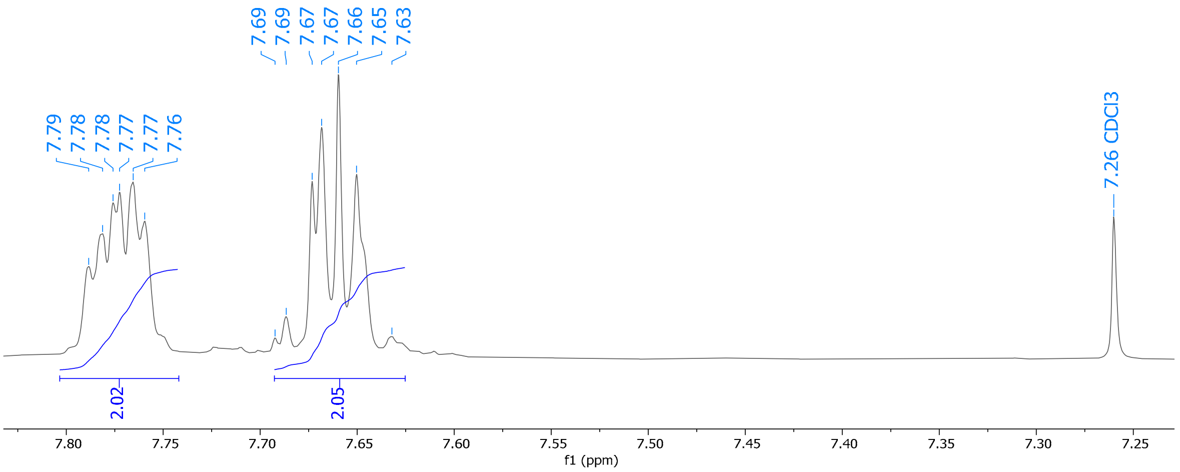

Figure S4D. ^{1}H-NMR spectrum ($CDCl_3$, 400 MHz) of **K4** (aromatics).

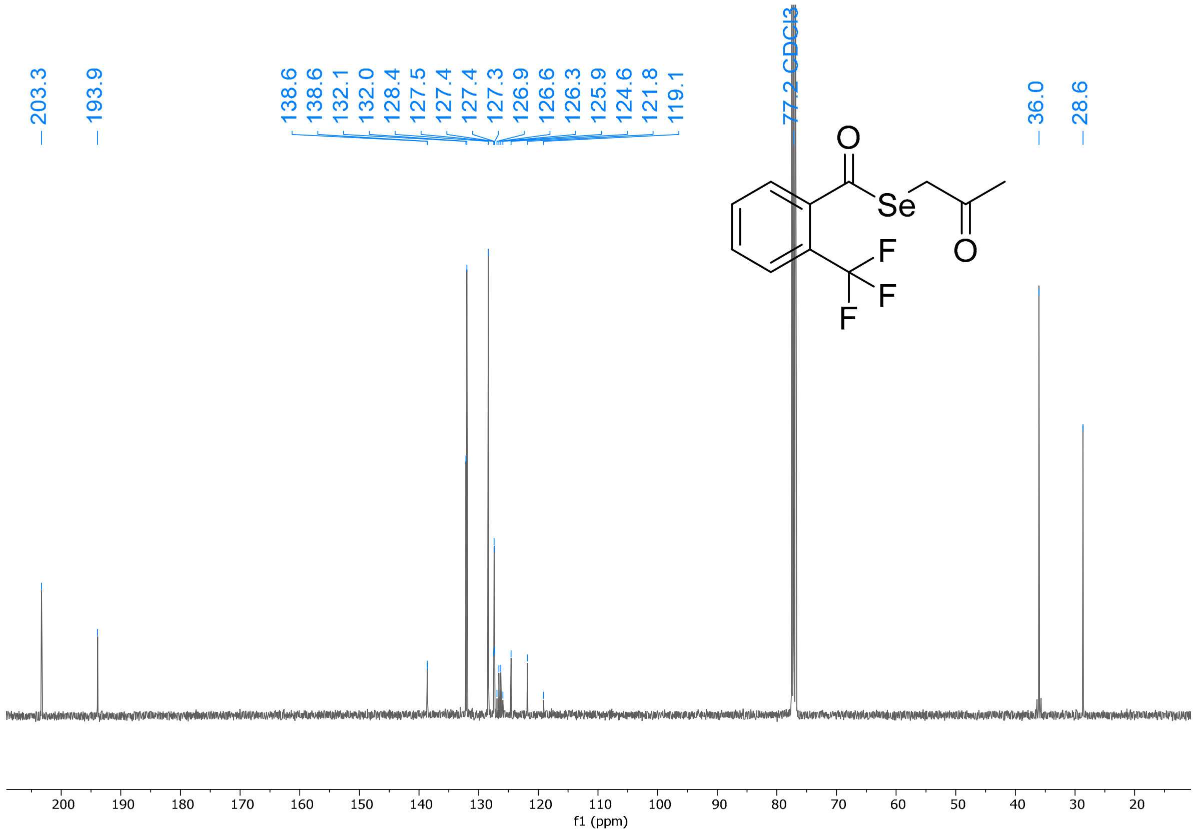

Figure S4E. ^{13}C-NMR spectrum ($CDCl_3$, 101 MHz) of **K4**.

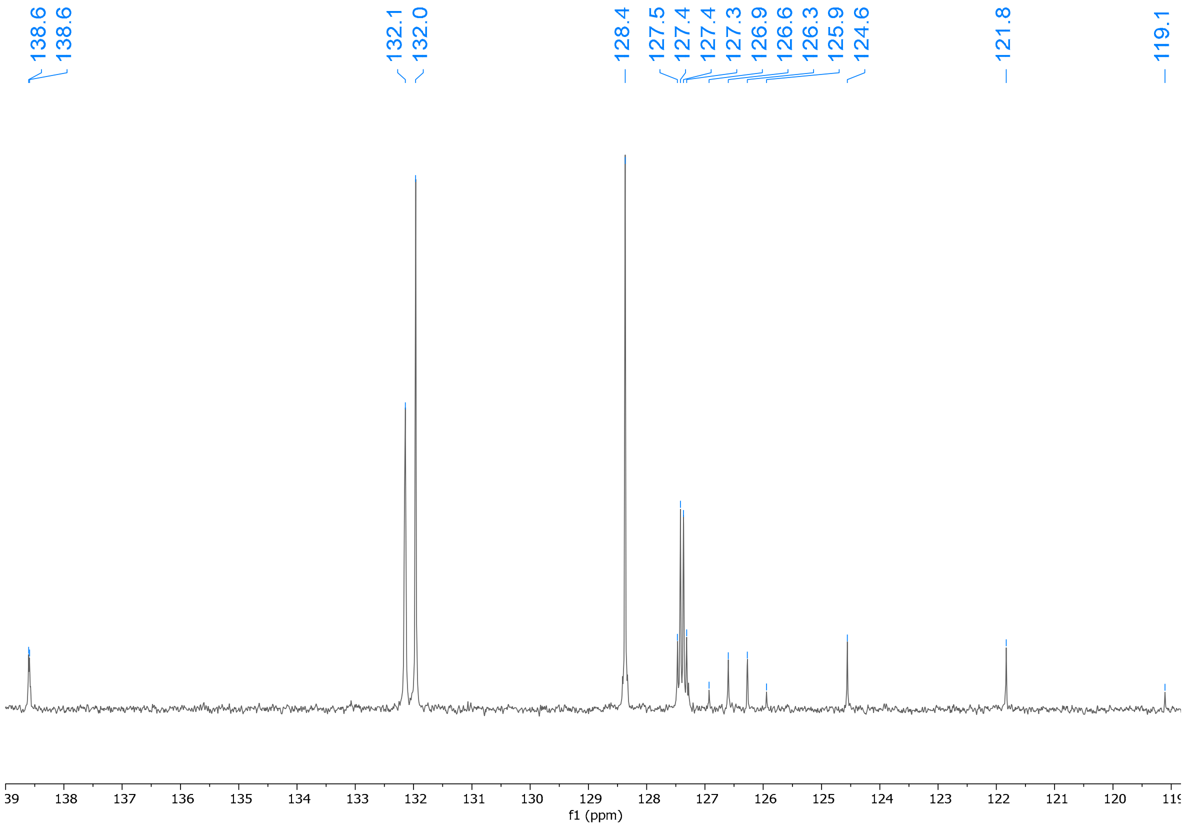

Figure S4F. ^{13}C-NMR spectrum ($CDCl_3$, 101 MHz) of **K4** (aromatics).

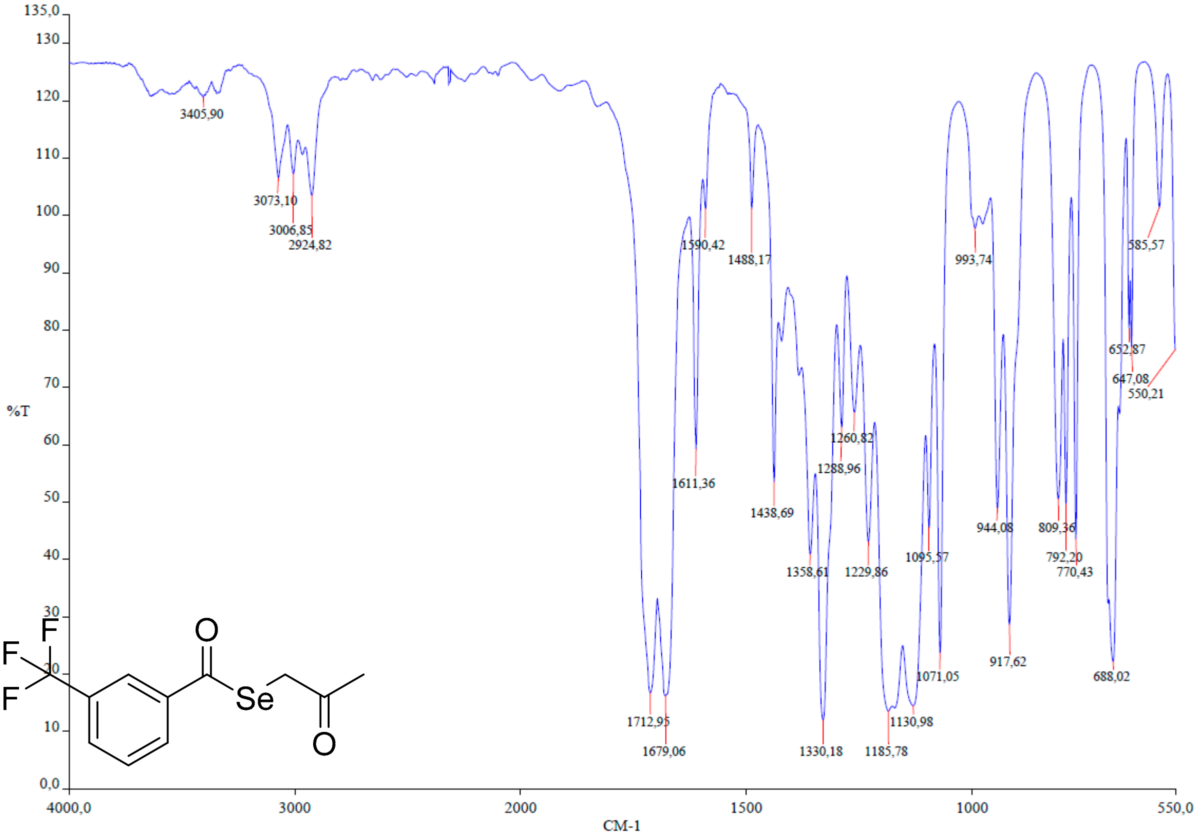

Figure S5. Compound **K5**: Se-(2-oxopropyl) 3-(trifluoromethyl)benzoselenoate. S5**A**. IR spectrum (NaCl) of **K5**.

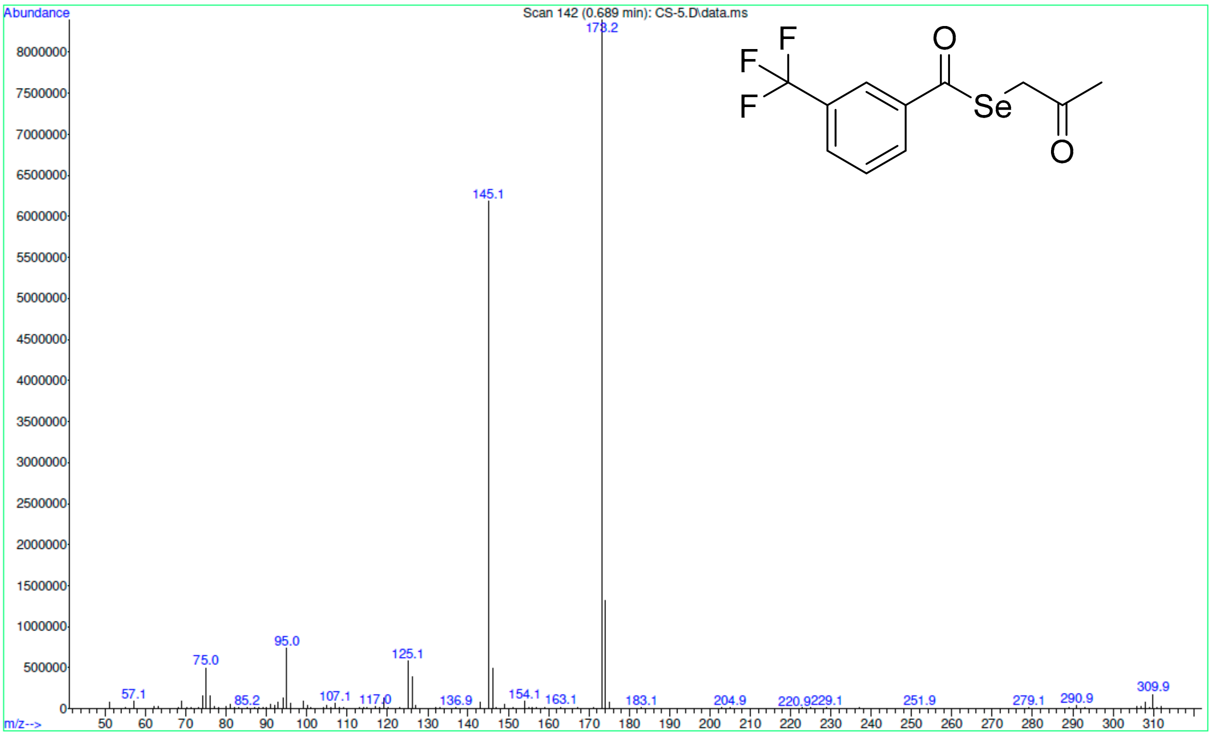

Figure S5B. DIP-MS spectrum of **K5**.

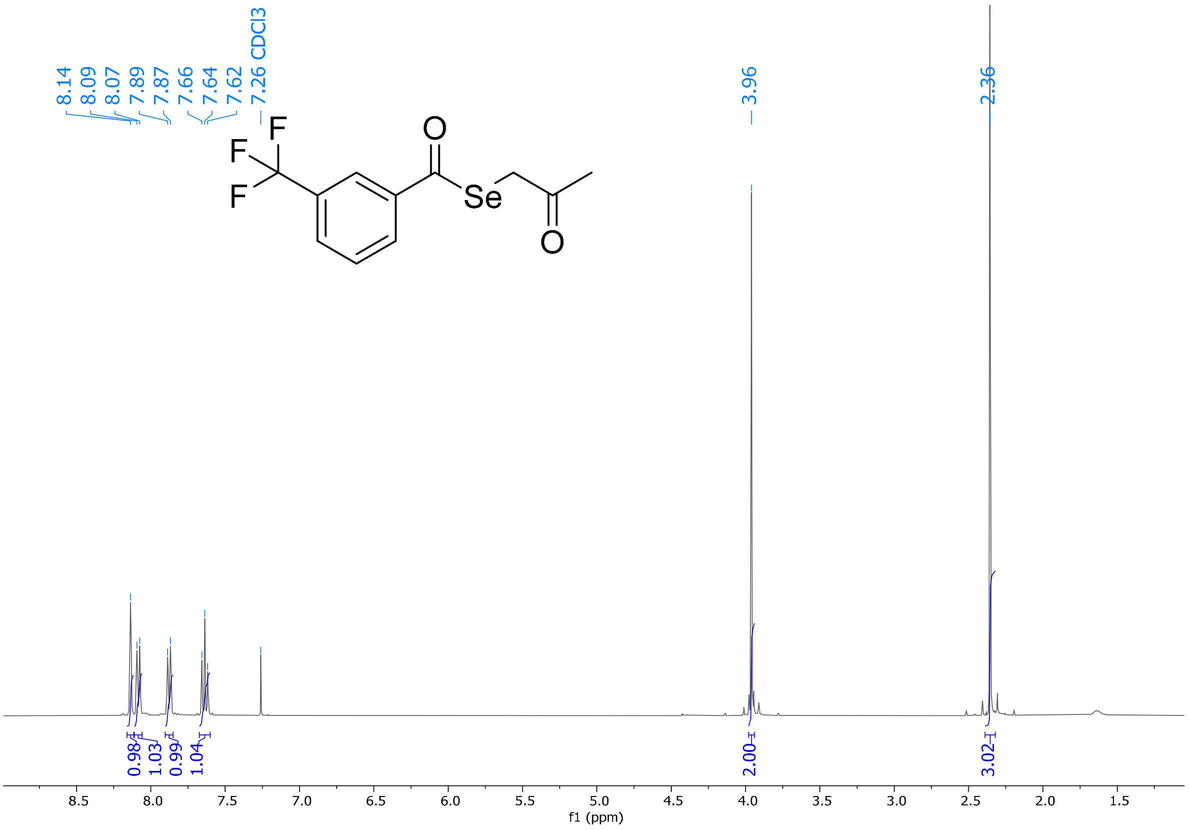

Figure S5C. ^{1}H-NMR spectrum ($CDCl_3$, 400 MHz) of **K5**.

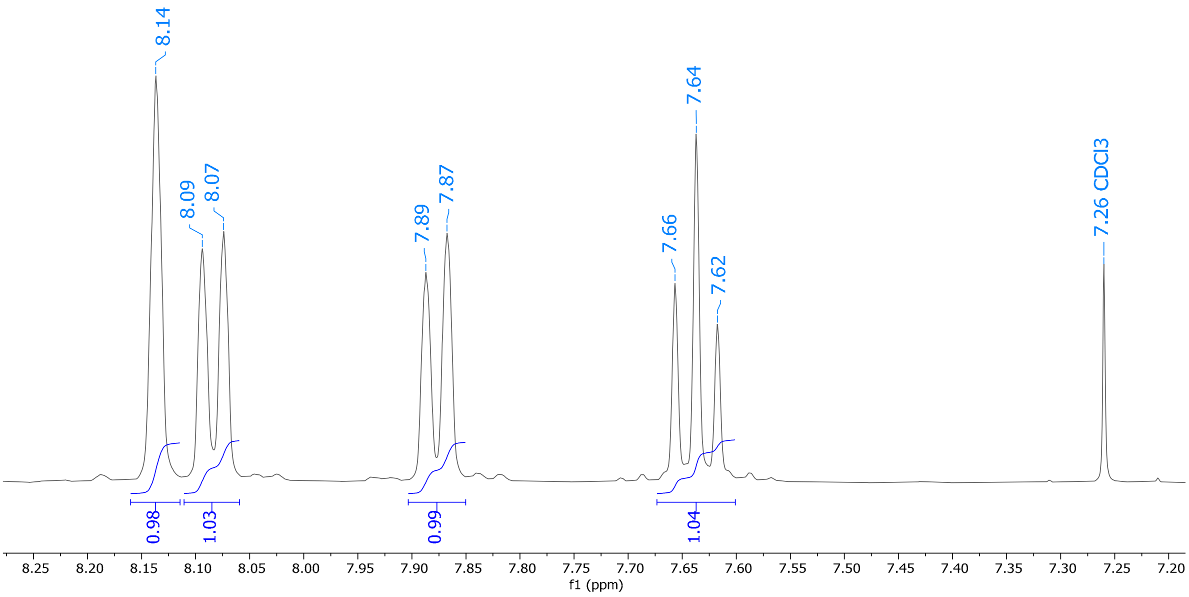

Figure S5D. ^{1}H-NMR spectrum ($CDCl_3$, 400 MHz) of **K5** (aromatics).

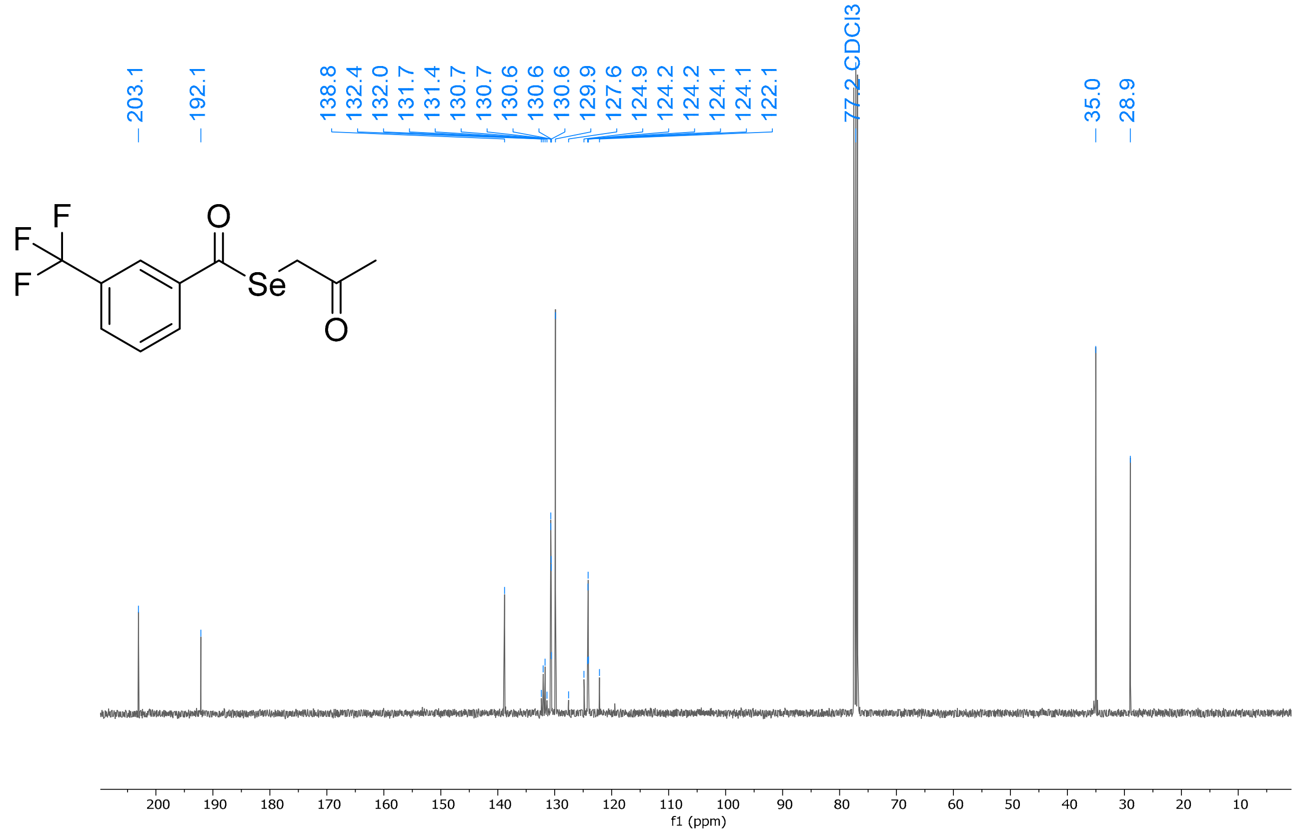

Figure S5E. ^{13}C-NMR spectrum ($CDCl_3$, 101 MHz) of **K5**.

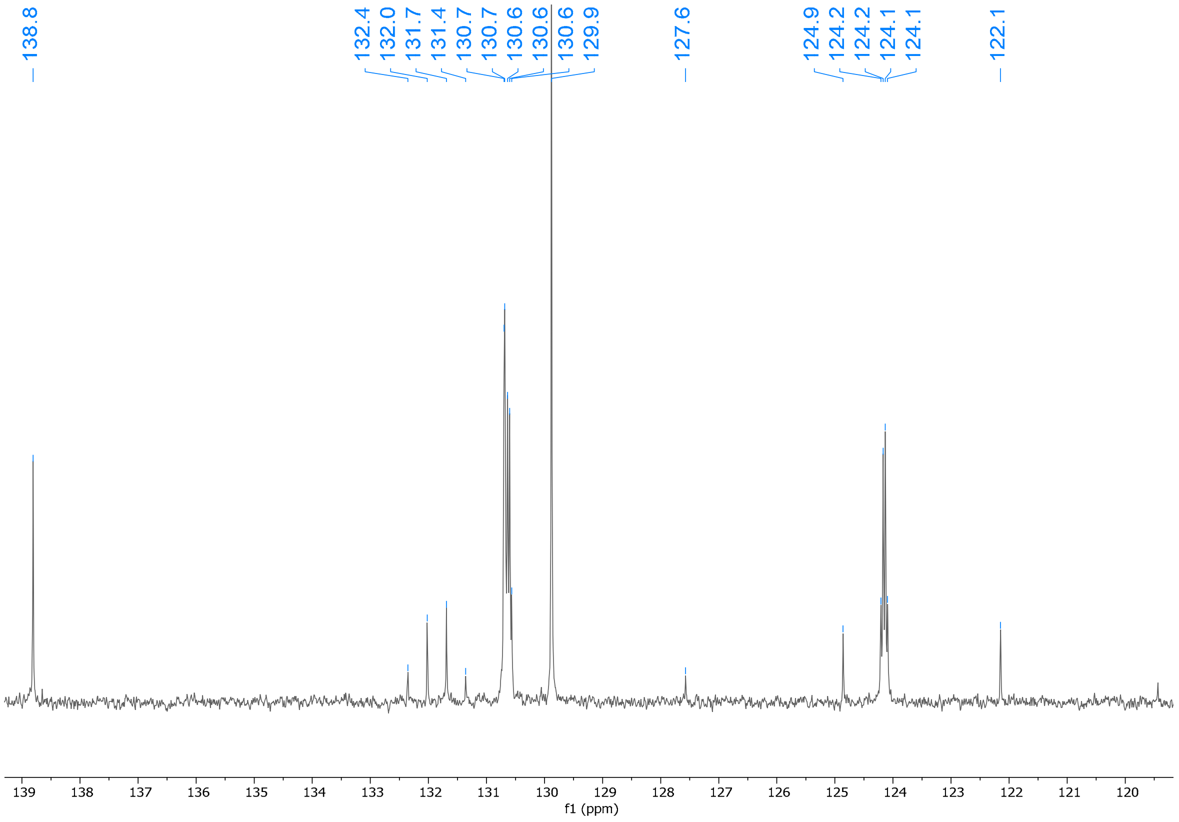

Figure S5F. ^{13}C-NMR spectrum ($CDCl_3$, 101 MHz) of **K5** (aromatics).

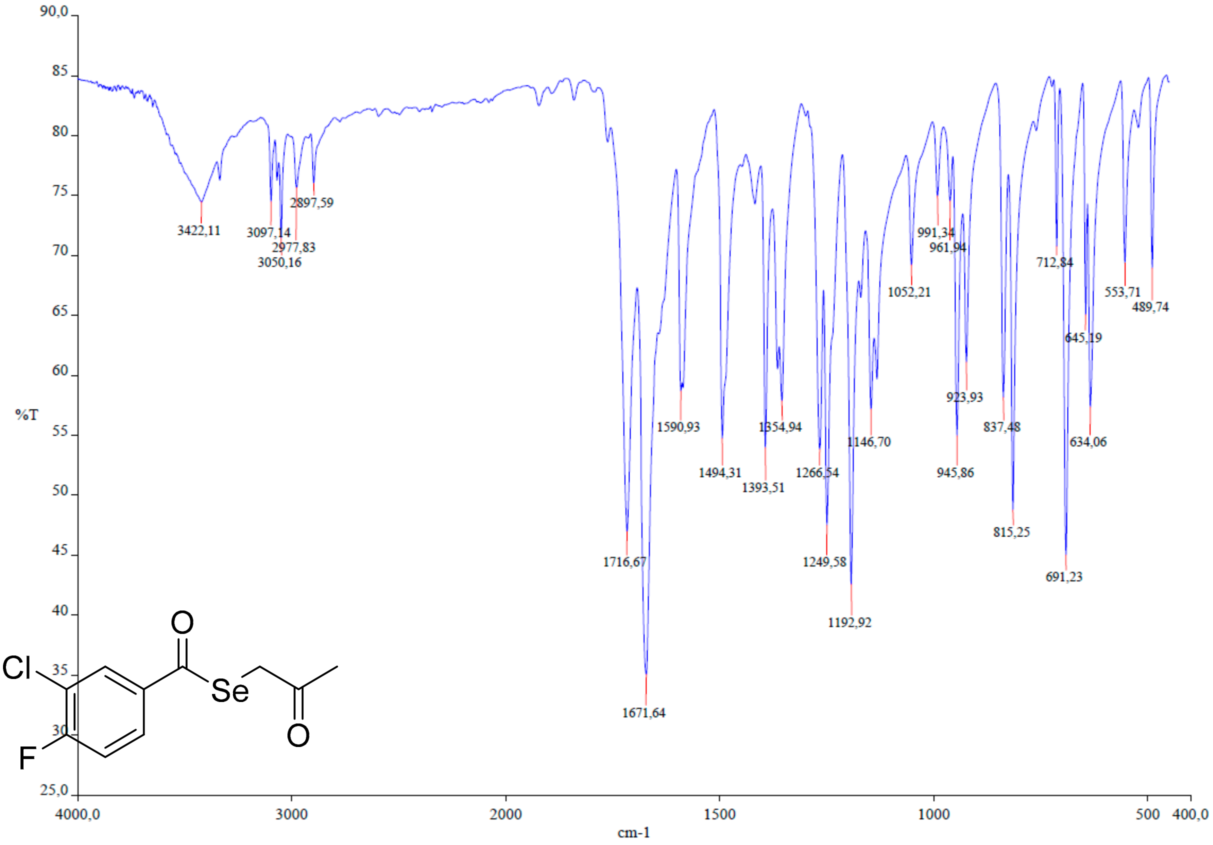

Figure S6. Compound **K6**: Se-(2-oxopropyl) 3-chloro-4-fluorobenzoselenoate. S6**A**. IR spectrum (KBr) of **K6**.

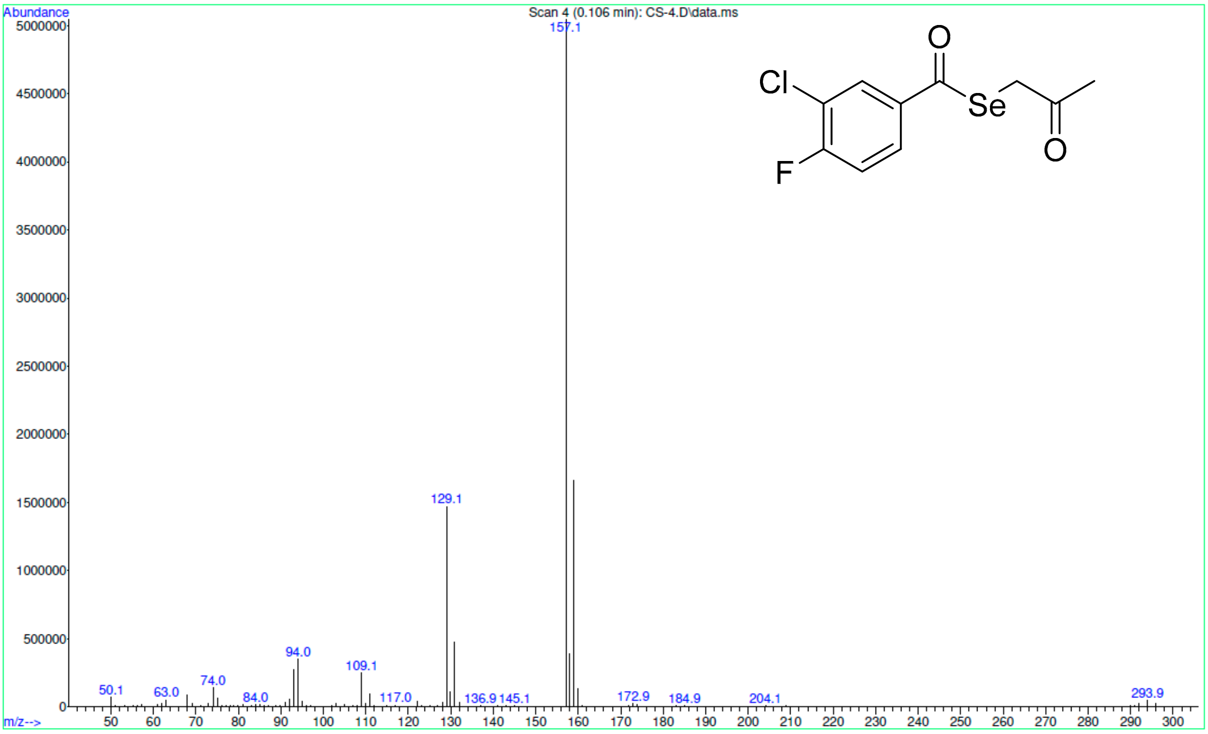

Figure S6B. DIP-MS spectrum of **K6**.

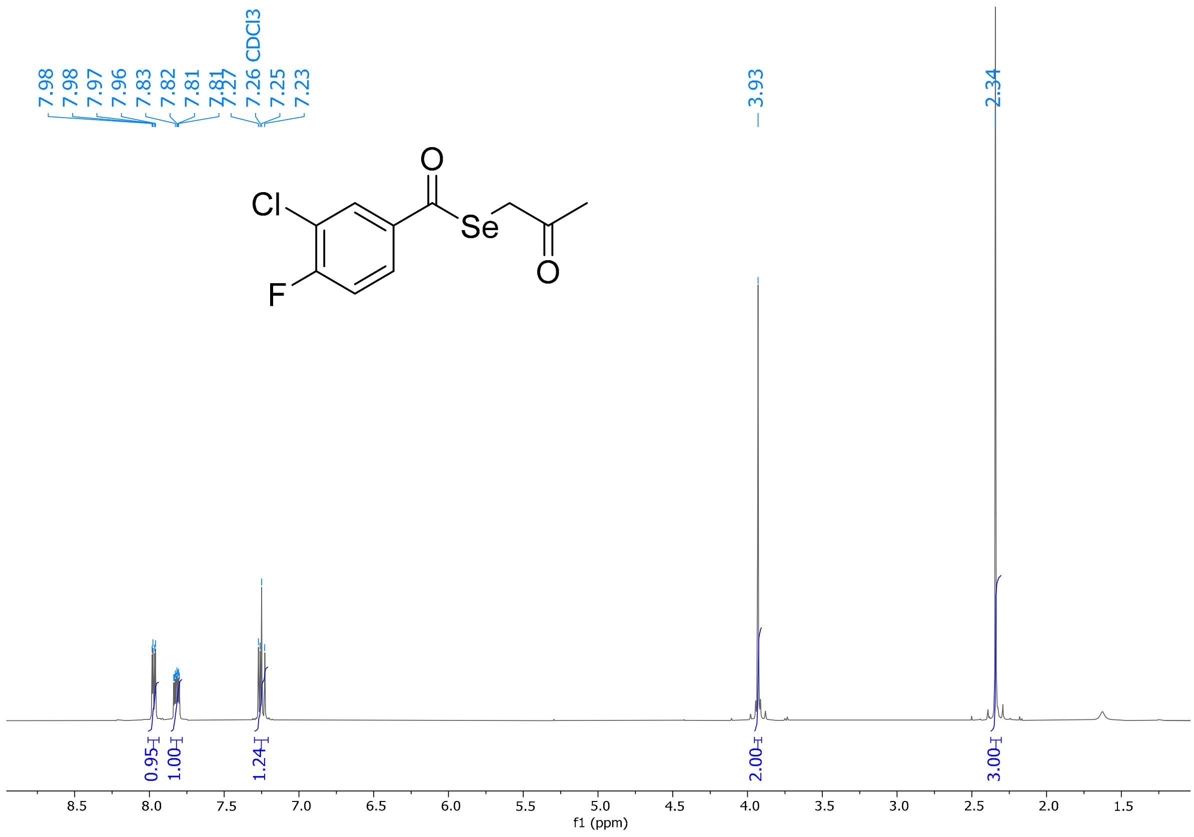

Figure S6C. ^{1}H-NMR spectrum ($CDCl_3$, 400 MHz) of **K6**.

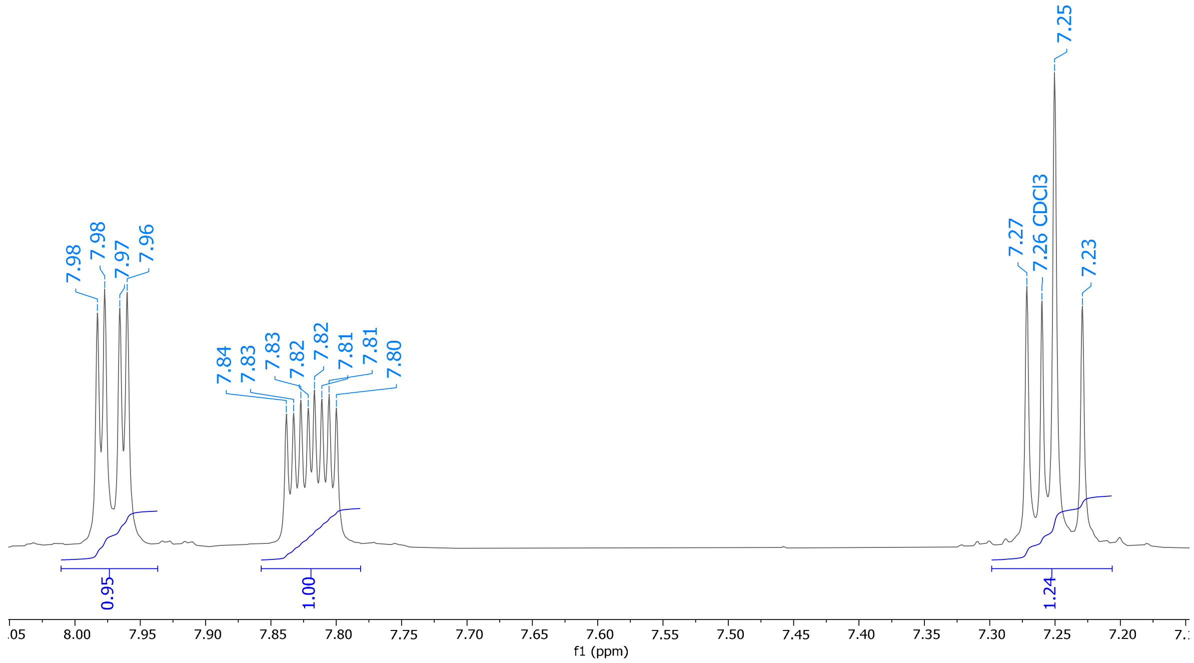

Figure S6D. ^{1}H-NMR spectrum ($CDCl_3$, 400 MHz) of **K6** (aromatics).

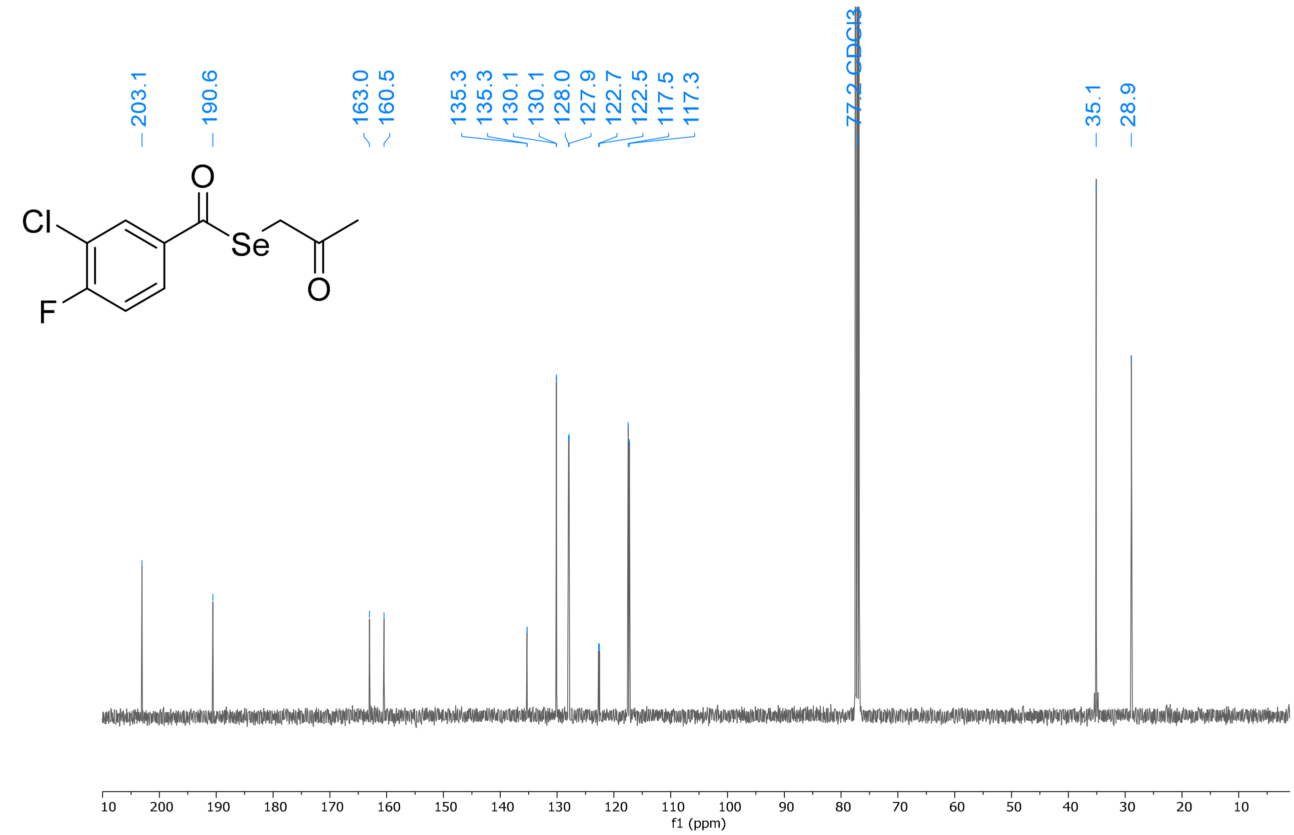

Figure S6E. ^{13}C-NMR spectrum ($CDCl_3$, 101 MHz) of **K6**.

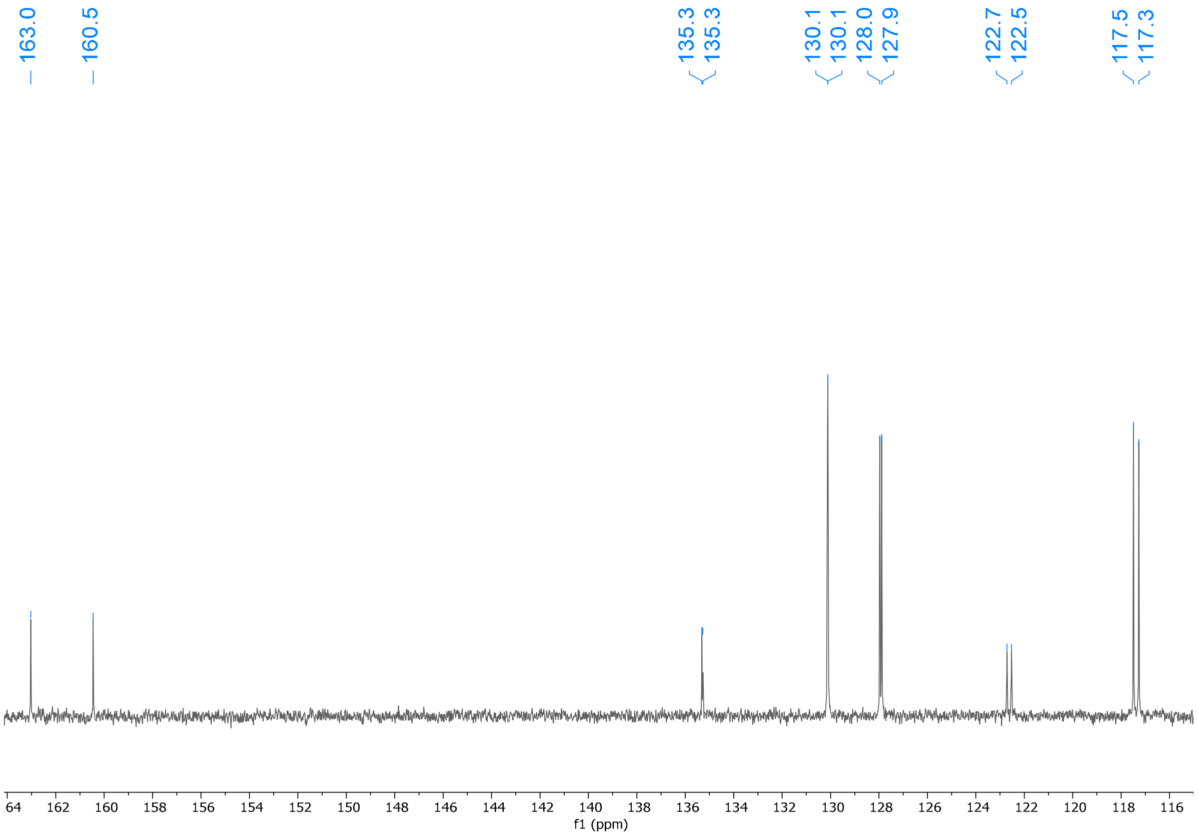

Figure S6F. ^{13}C-NMR spectrum ($CDCl_3$, 101 MHz) of **K6** (aromatics).

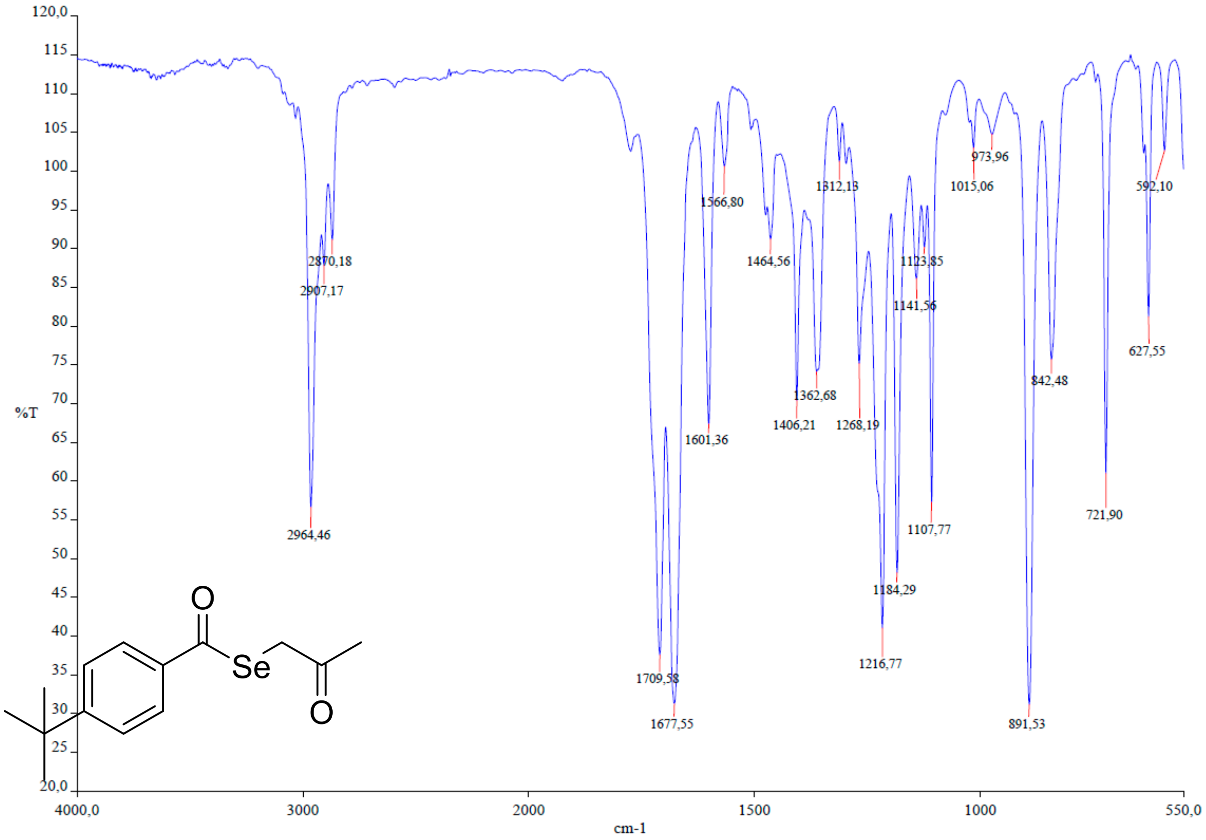

Figure S7. Compound **K7**: Se-(2-oxopropyl) 4-(tert-butyl)benzoselenoate. S7**A**. IR spectrum (NaCl) of **K7**.

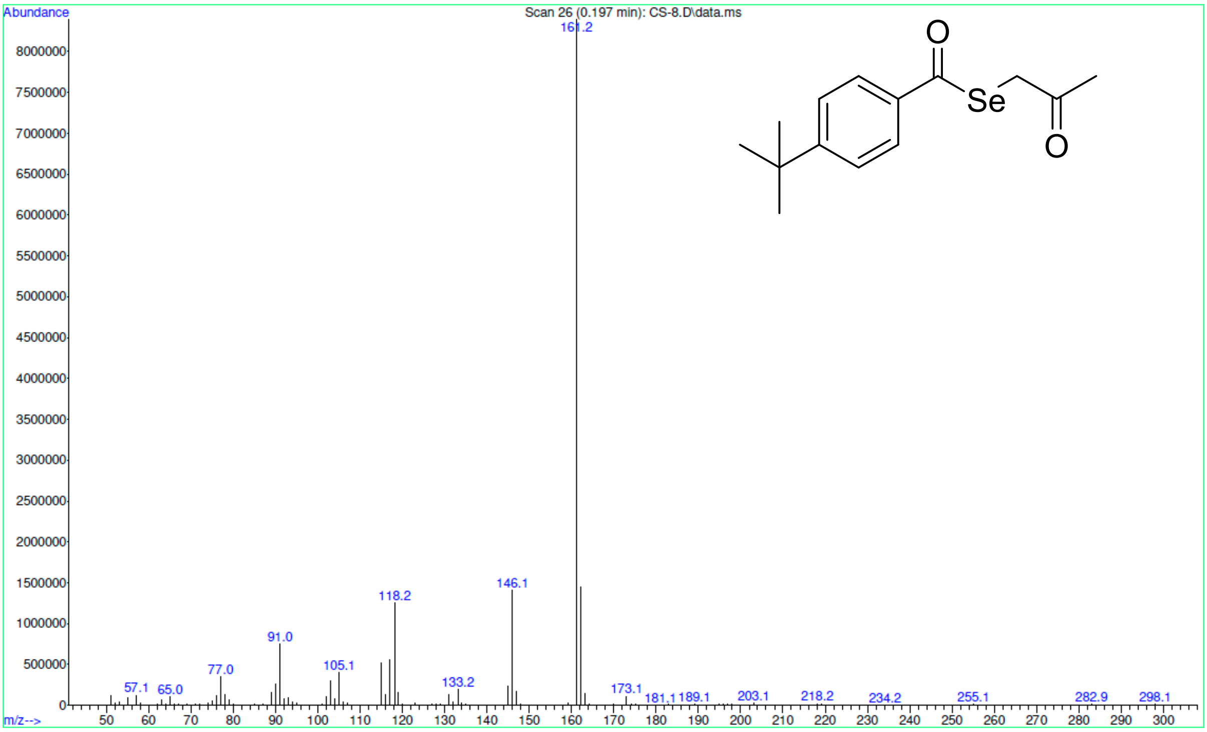

Figure S7B. DIP-MS spectrum of **K7**.

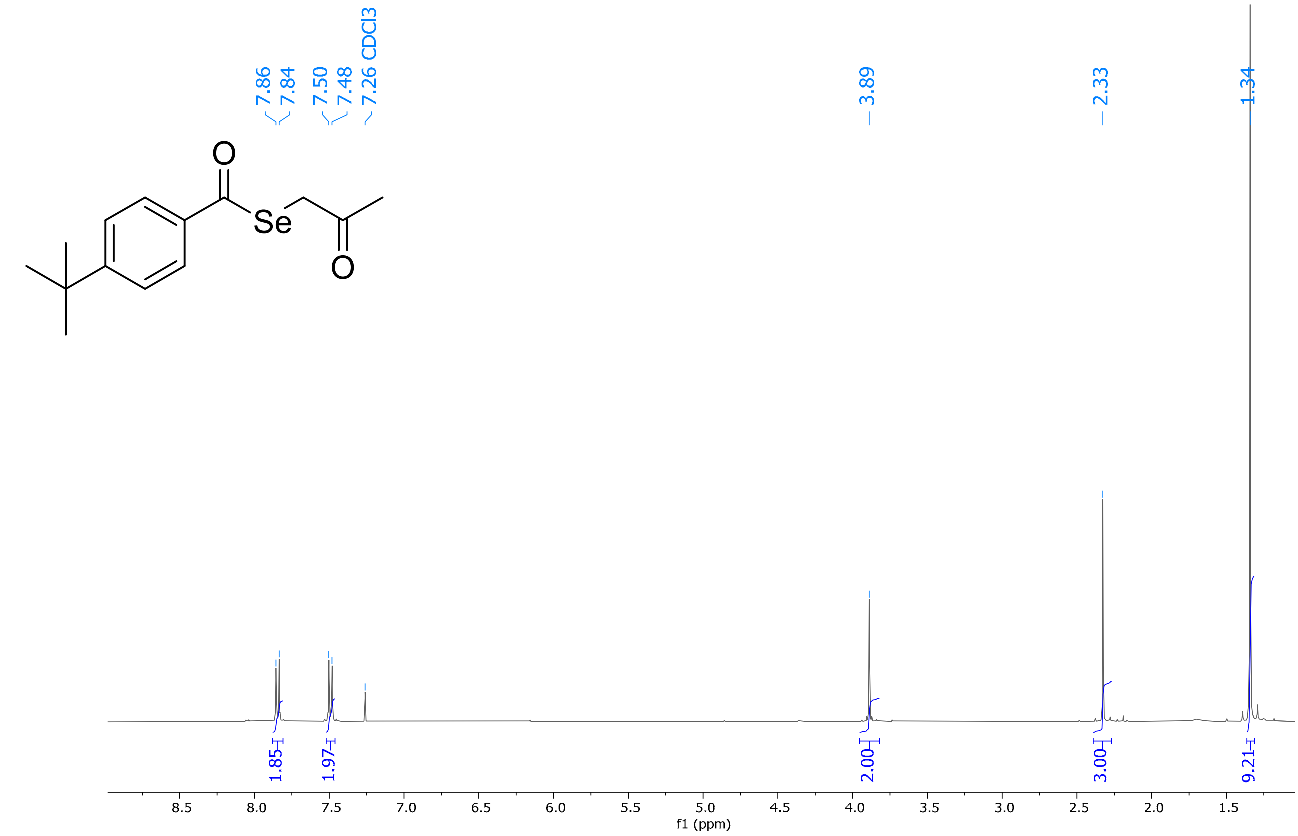

Figure S7C. ^{1}H-NMR spectrum ($CDCl_3$, 400 MHz) of **K7**.

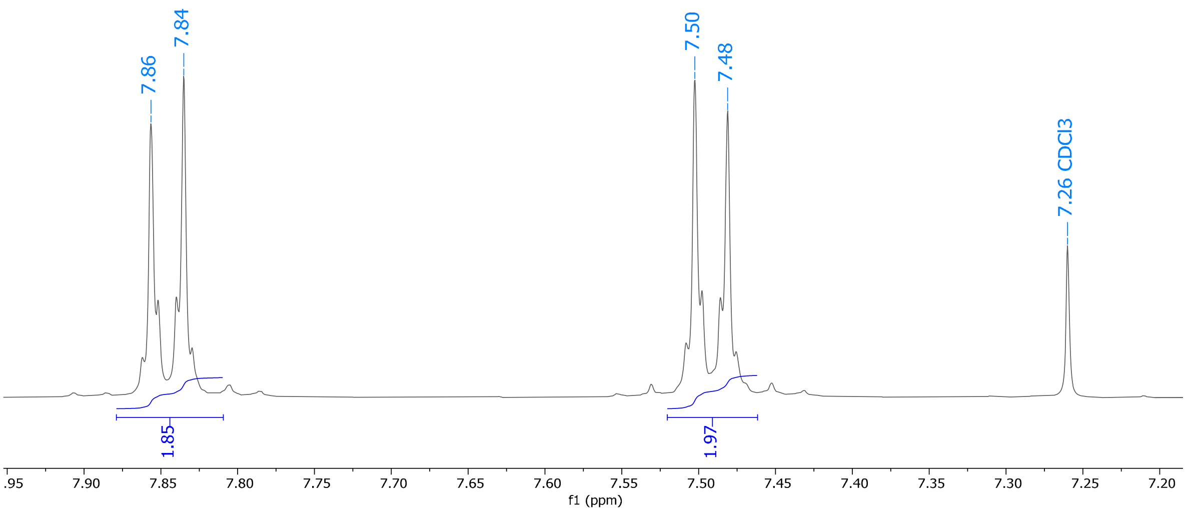

Figure S7D. ^{1}H-NMR spectrum ($CDCl_3$, 400 MHz) of **K7** (aromatics).

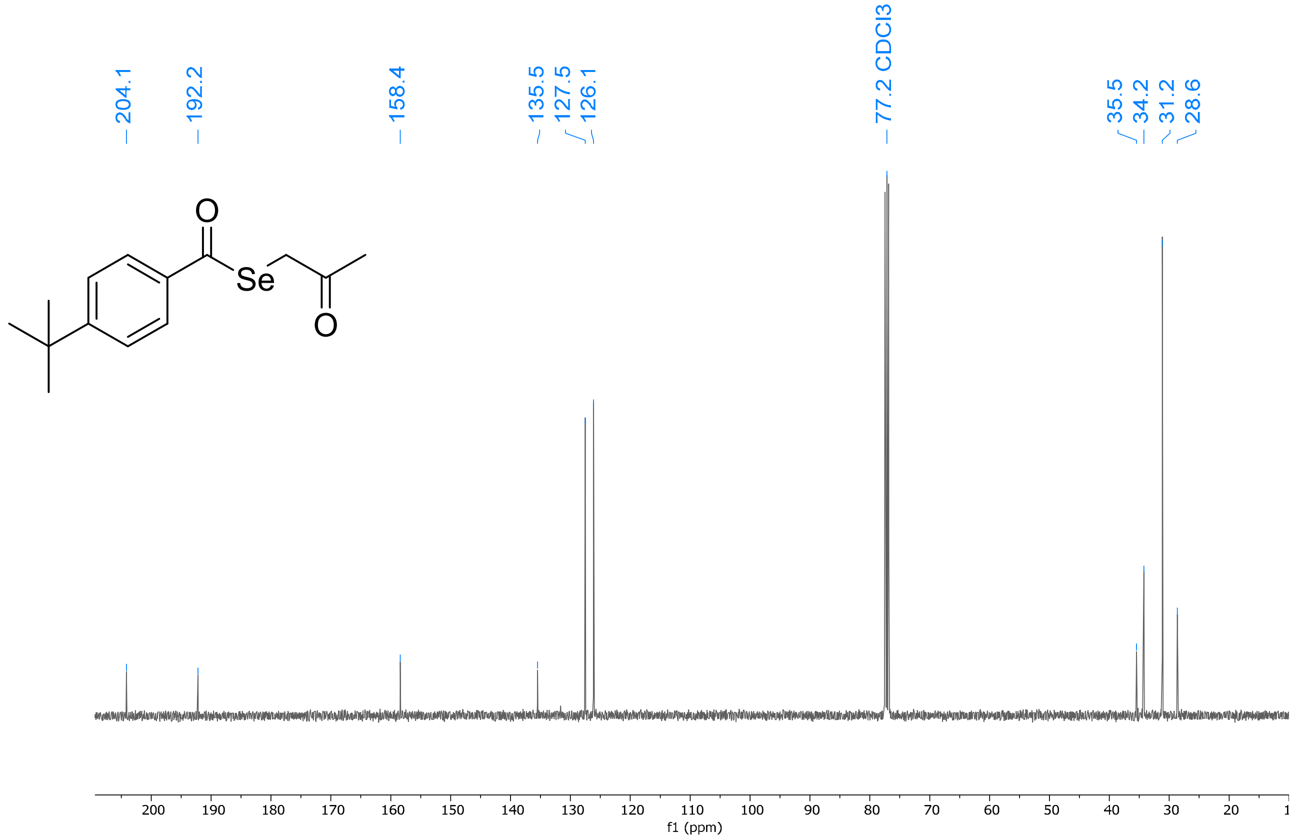

Figure S7E. ^{13}C-NMR spectrum ($CDCl_3$, 101 MHz) of **K7**.

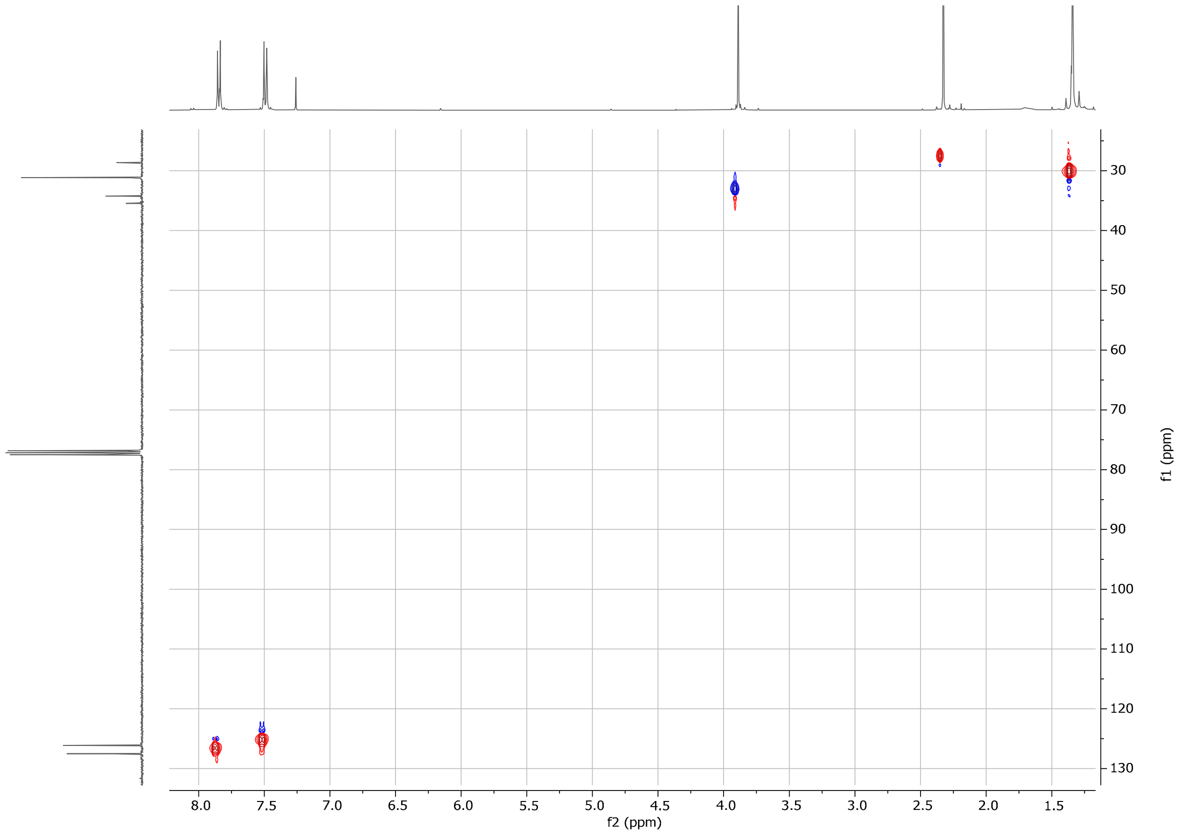

Figure S7F. ^{1}H-^{13}C HSQC NMR spectrum ($CDCl_3$) of **K7**.

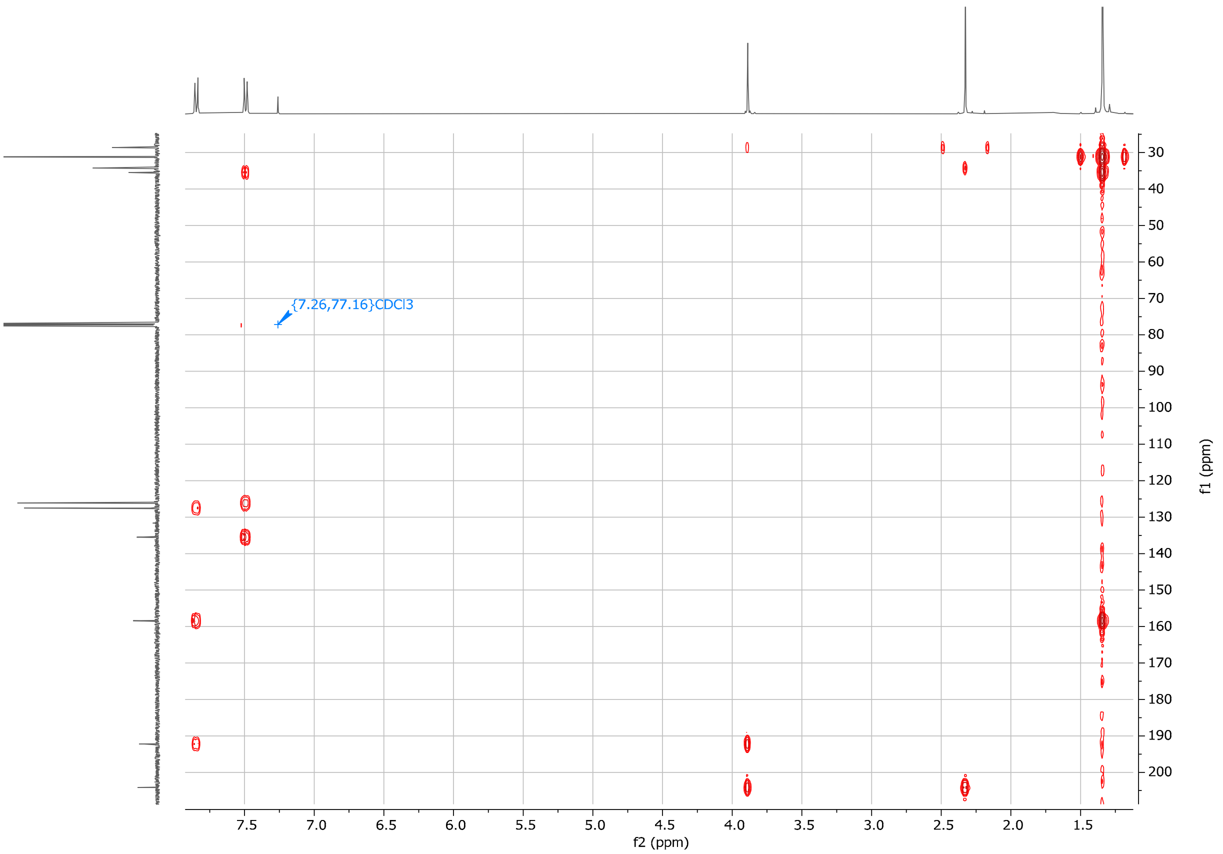

Figure S7G. ^{1}H-^{13}C HMBC NMR spectrum ($CDCl_3$) of **K7**.

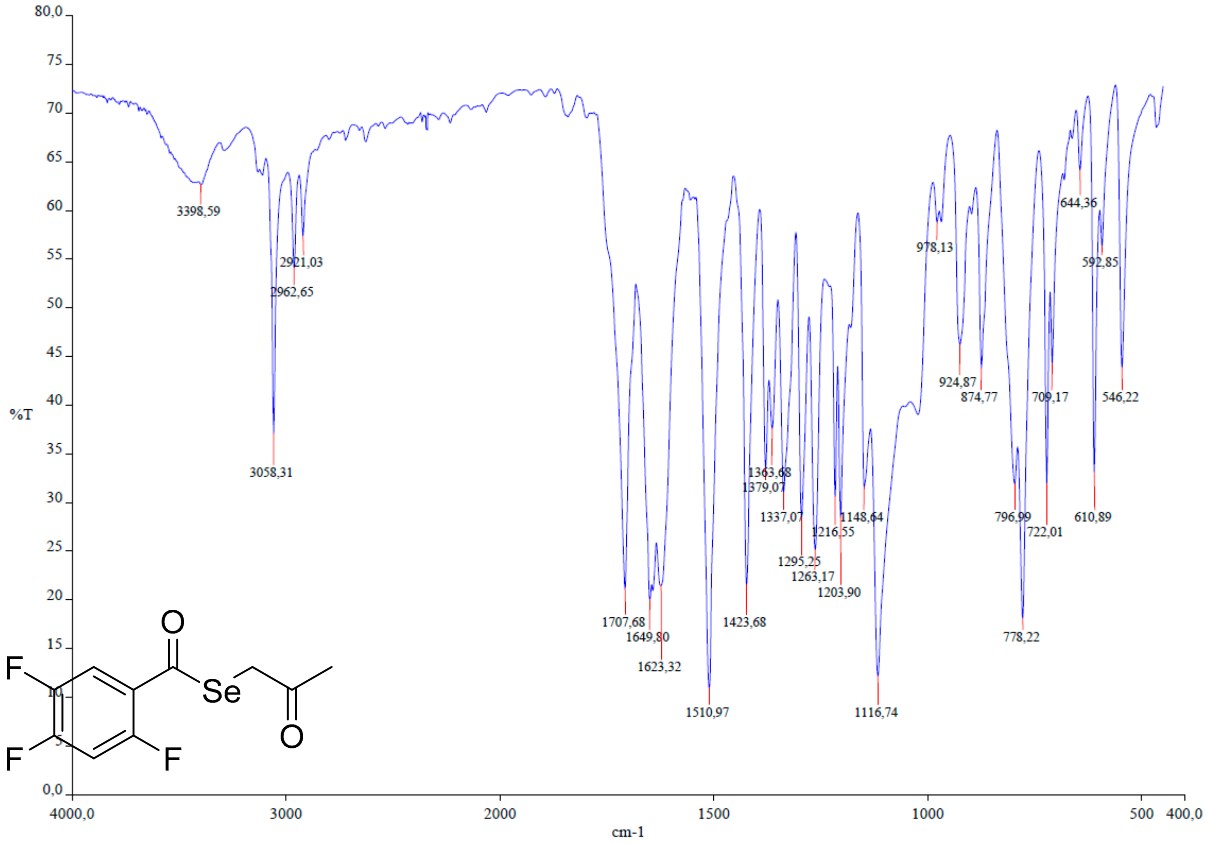

Figure S8. Compound **K8**: Se-(2-oxopropyl) 2,4,5-trifluorobenzoselenoate. S8A. IR spectrum (KBr) of **K8**.

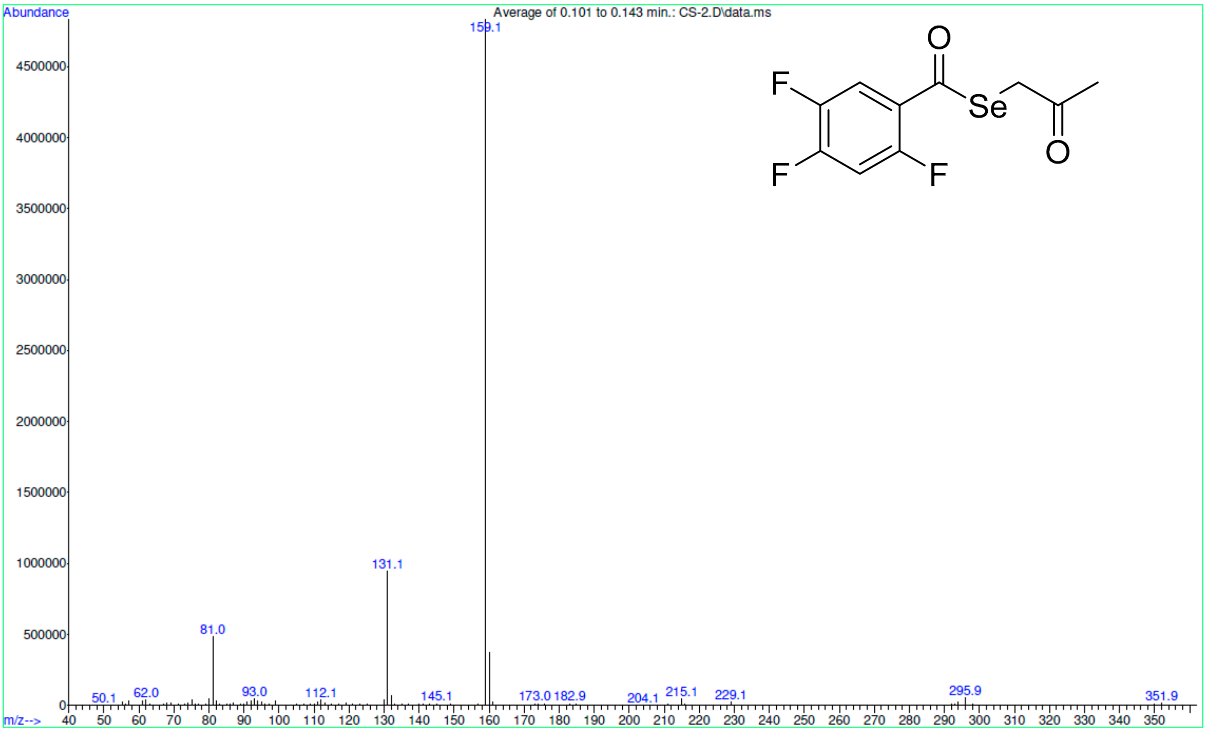

Figure S8B. DIP-MS spectrum of **K8**.

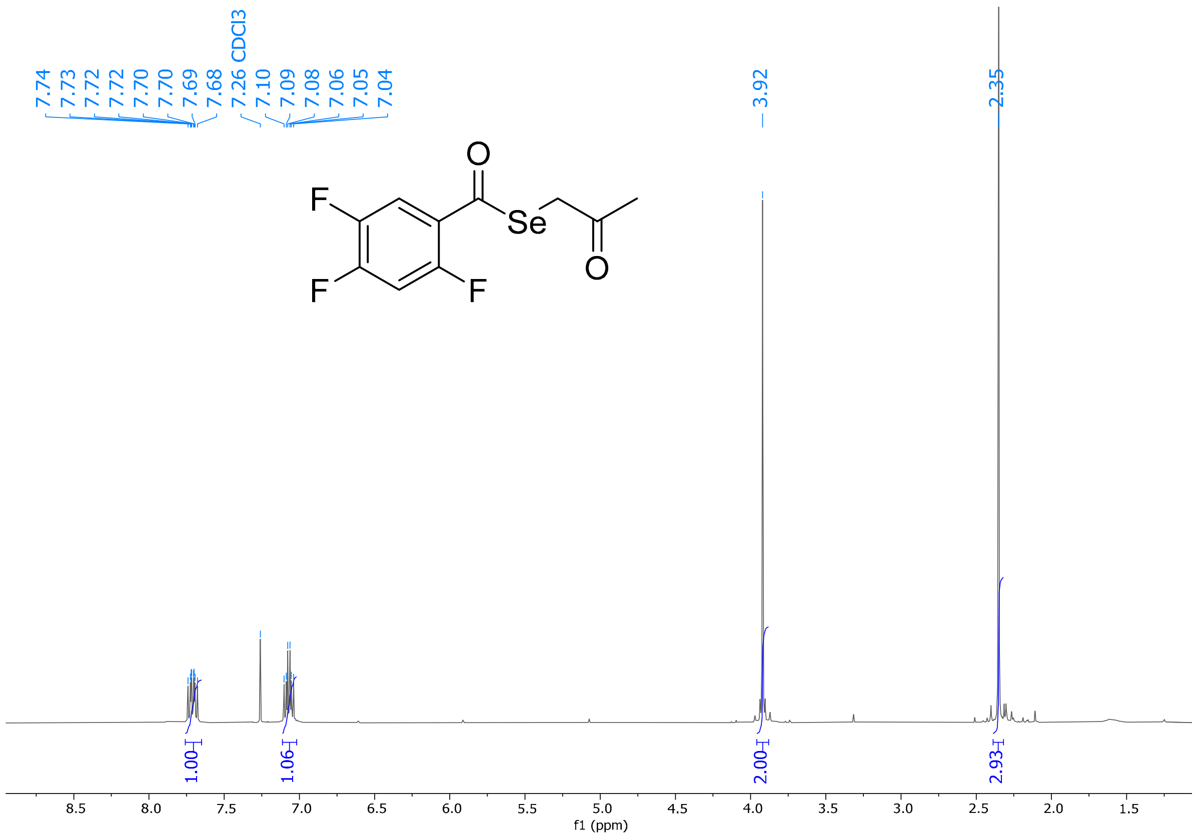

Figure S8. C. ^{1}H-NMR spectrum ($CDCl_3$, 400 MHz) of **K8**.

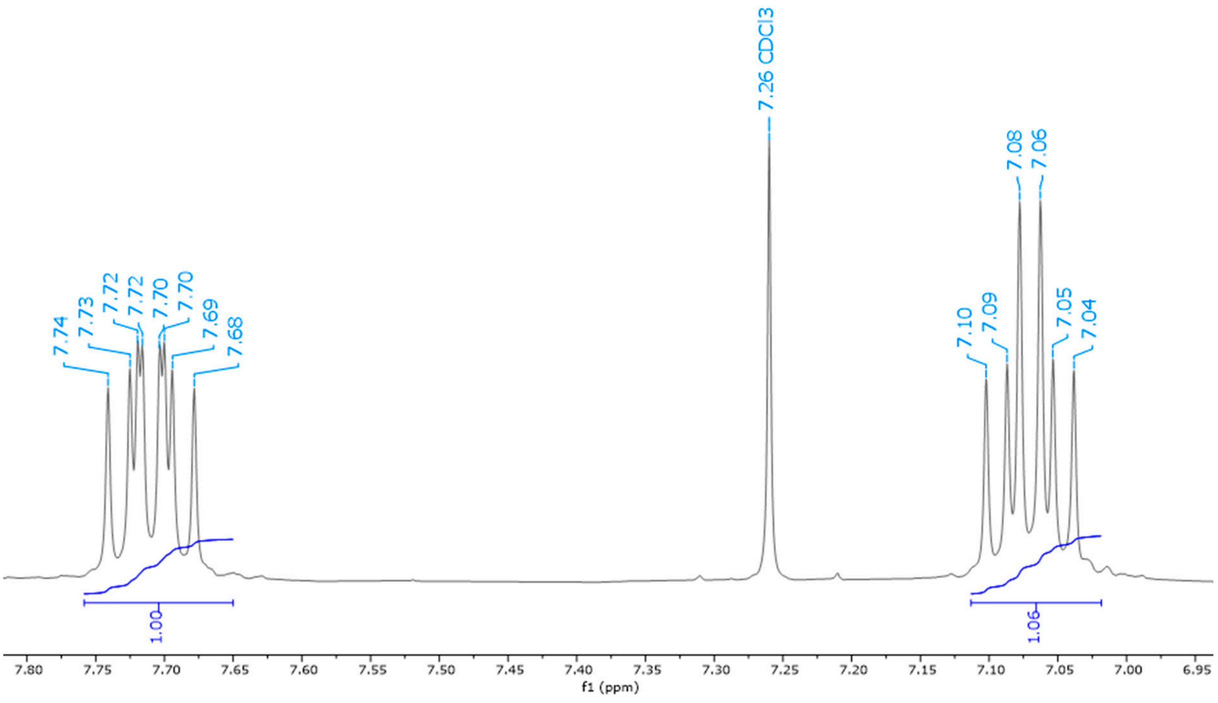

Figure S8D. ^{1}H-NMR spectrum ($CDCl_3$, 400 MHz) of **K8** (aromatics).

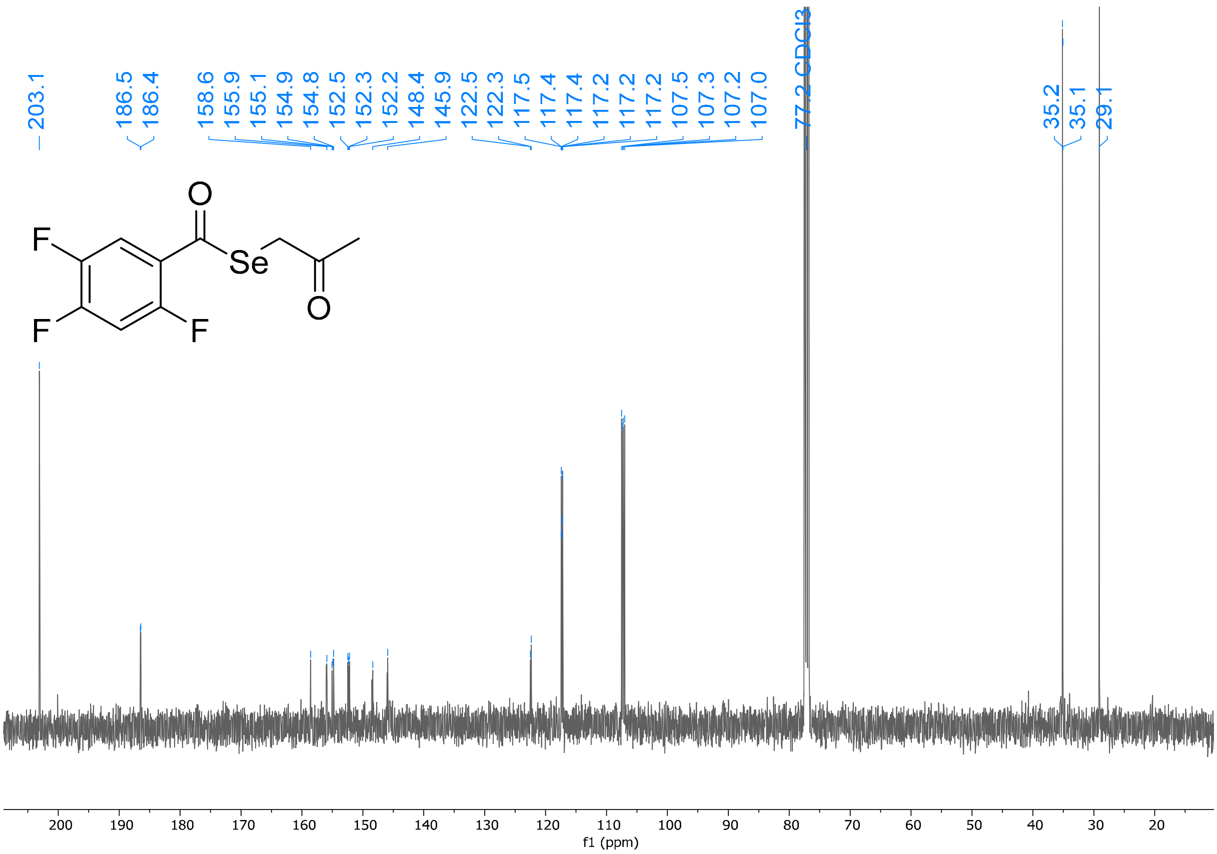

Figure S8E. ^{13}C-NMR spectrum ($CDCl_3$, 101 MHz) of **K8**.

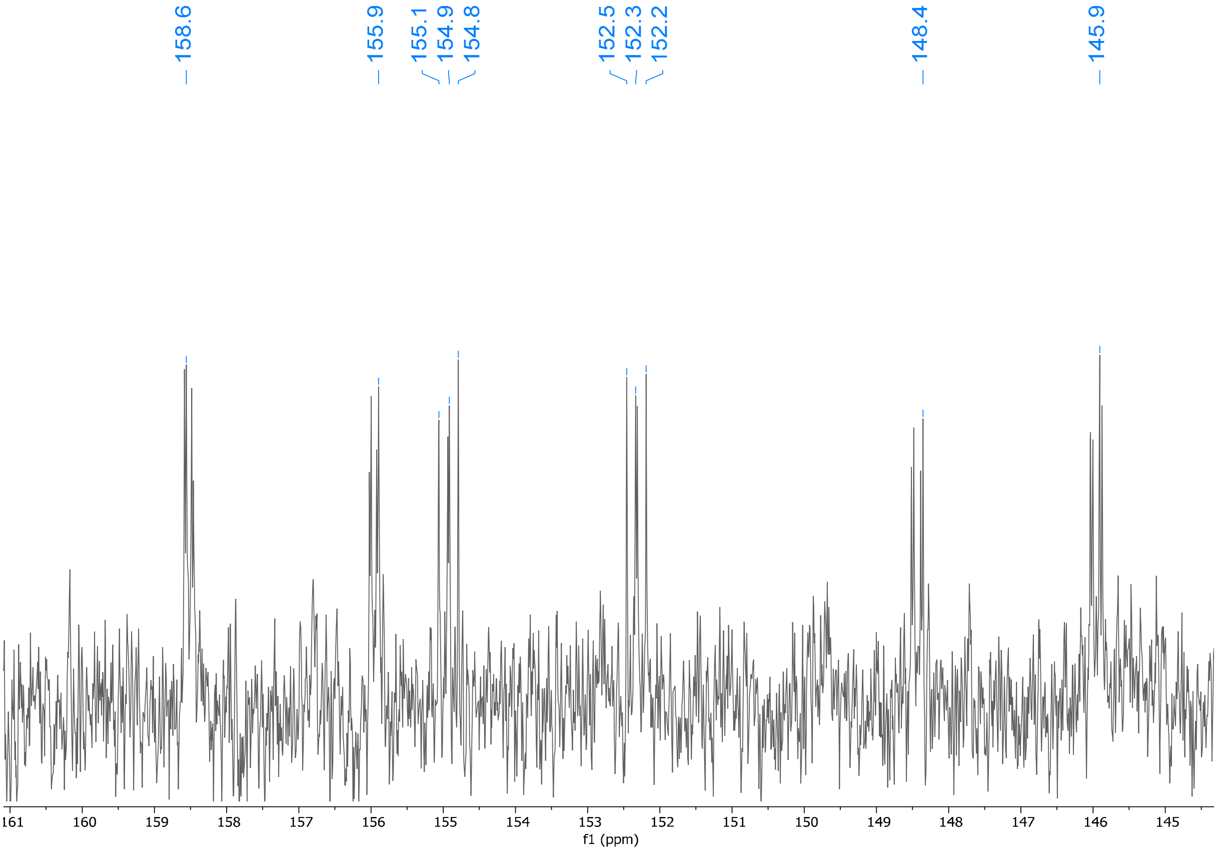

Figure S8F. ^{13}C-NMR spectrum ($CDCl_3$, 101 MHz, aromatic carbons bound to fluorine) of **K8**.

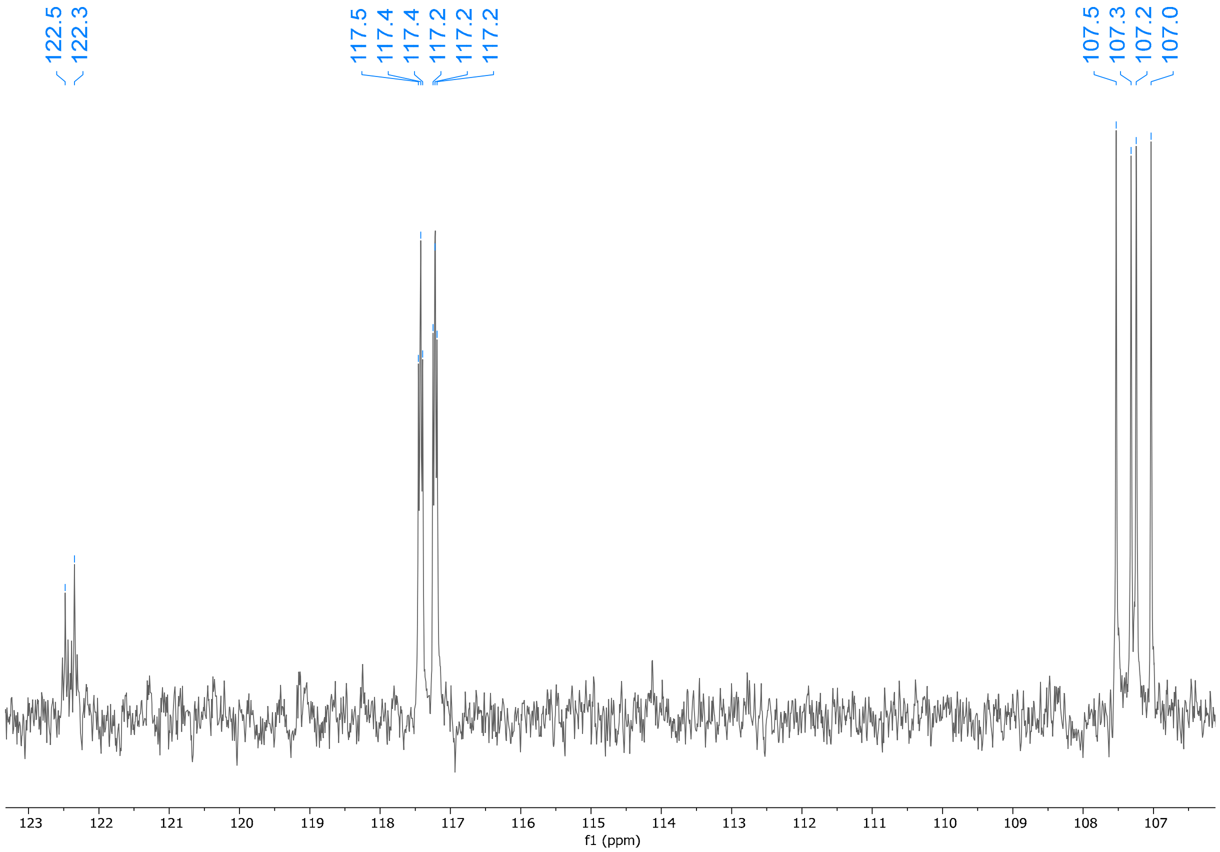

Figure S8G. ^{13}C-NMR spectrum ($CDCl_3$, 101 MHz, aromatic carbons not bound to fluorine) of **K8.**

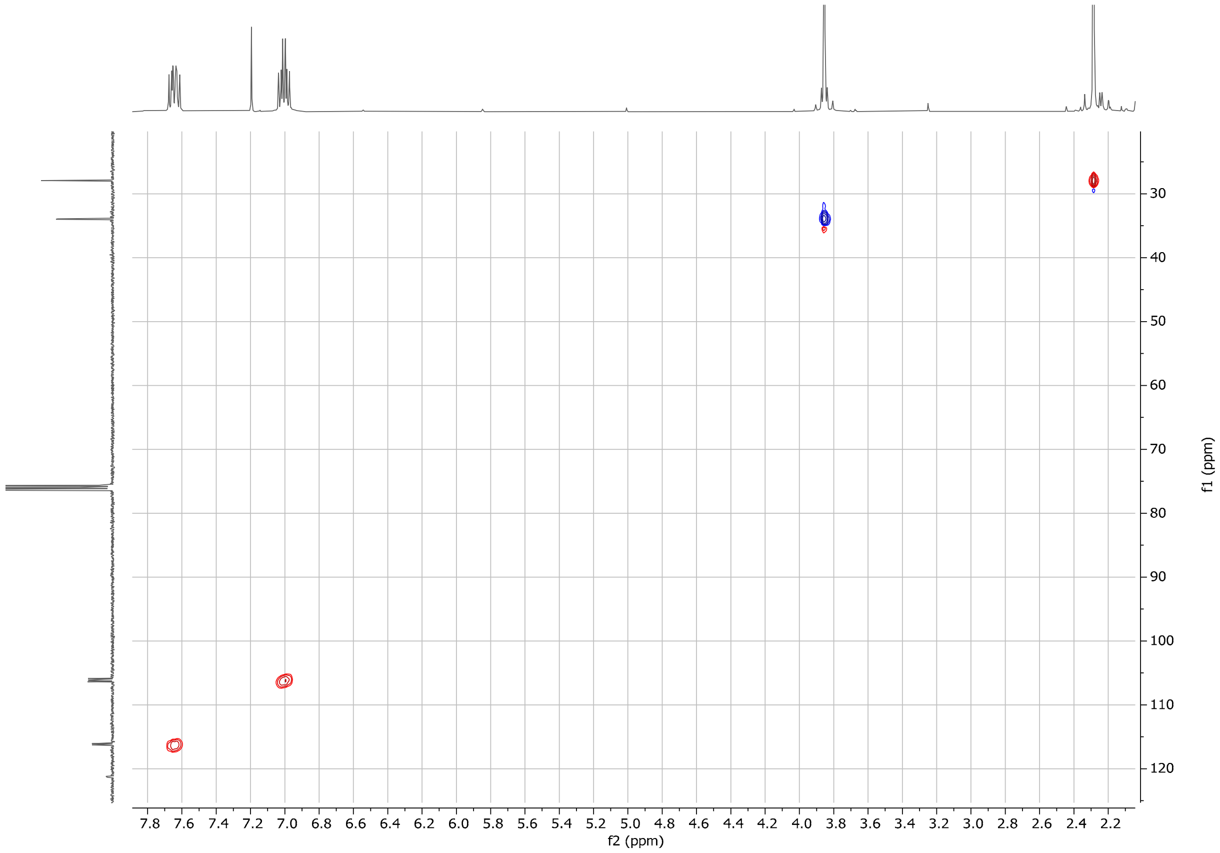

Figure S8H. ^{1}H-^{13}C HSQC NMR spectrum ($CDCl_3$) of **K8**.

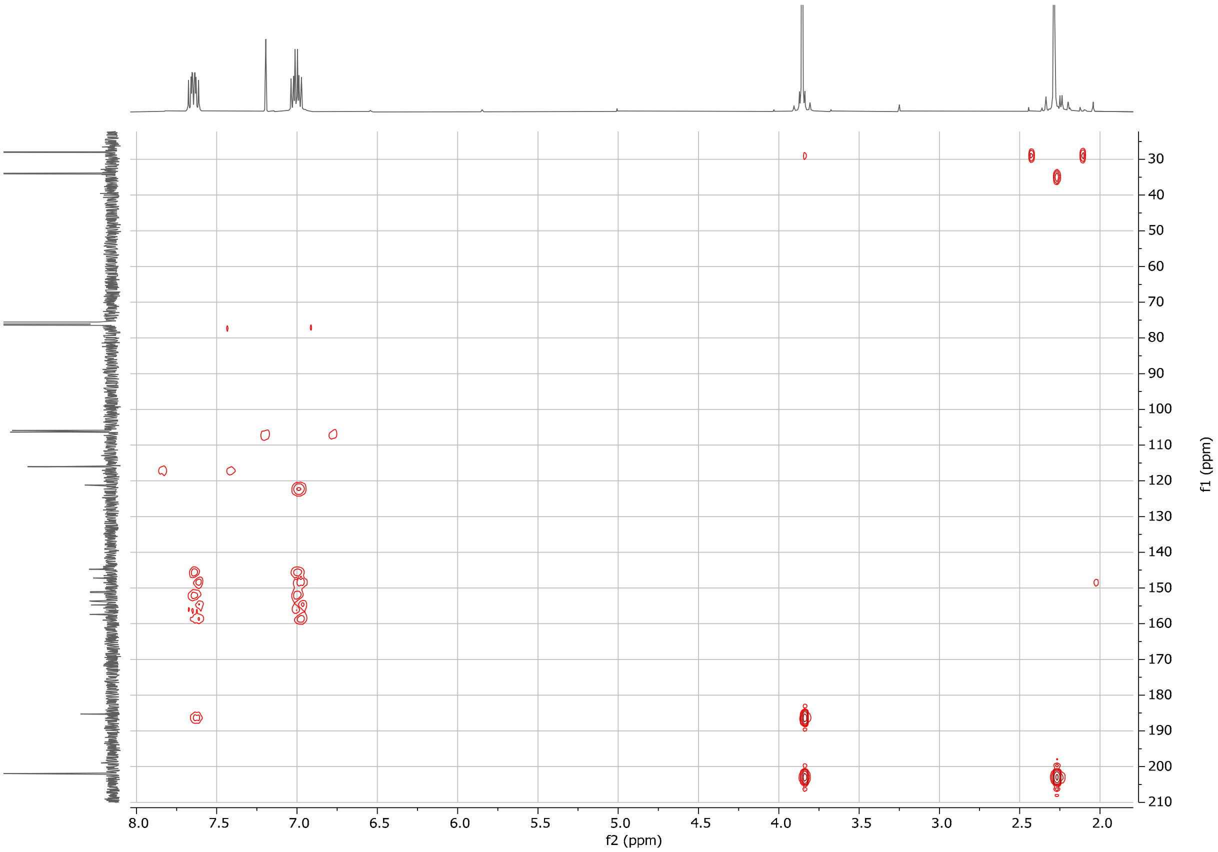

Figure S8I. ^{1}H-^{13}C HMBC NMR spectrum ($CDCl_3$) of **K8**.

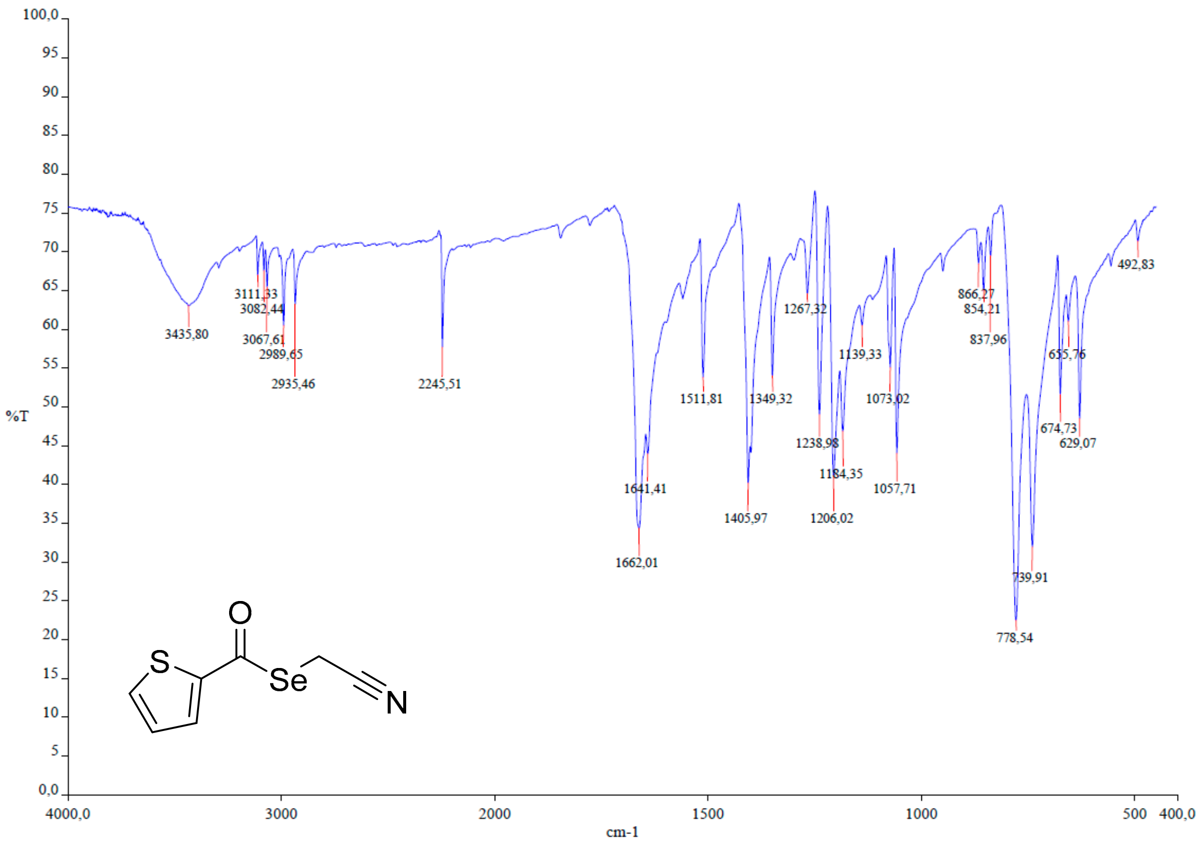

Figure S9. Compound **N1**: Se-(cyanomethyl) thiophene-2-carboselenoate. S9**A**. IR spectrum (KBr) of **N1**.

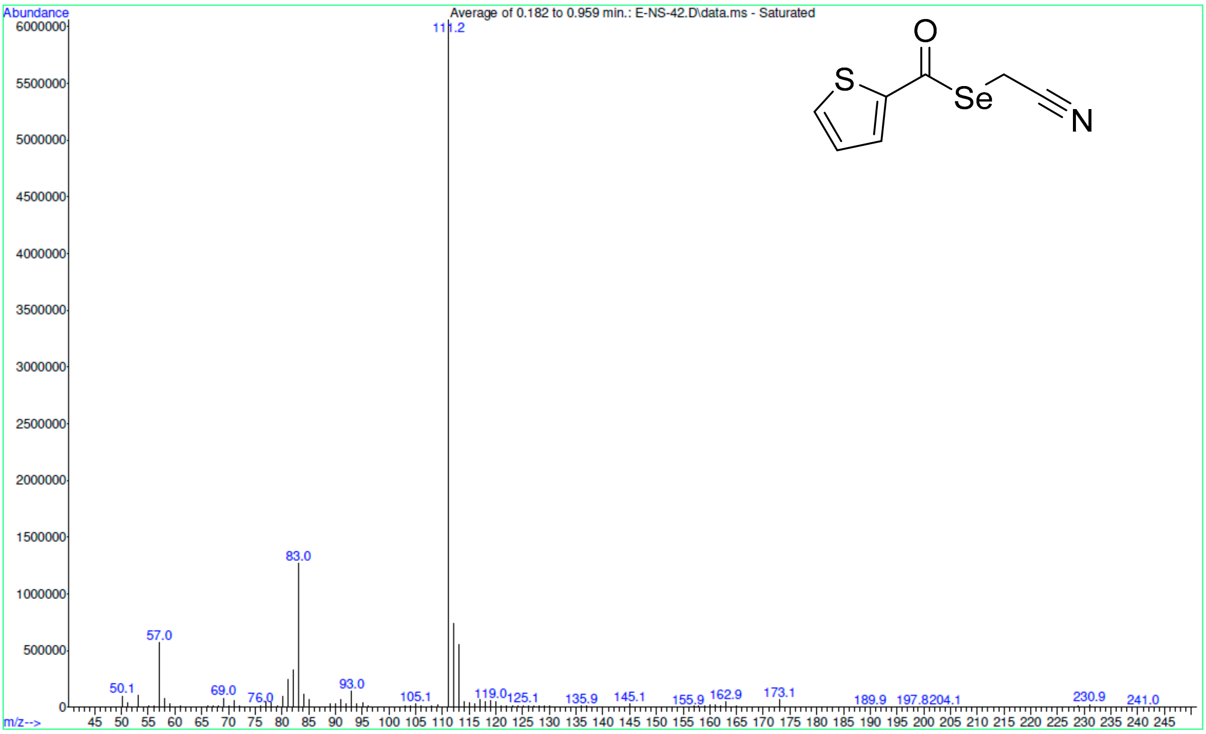

Figure S9B. DIP-MS spectrum of **N1**.

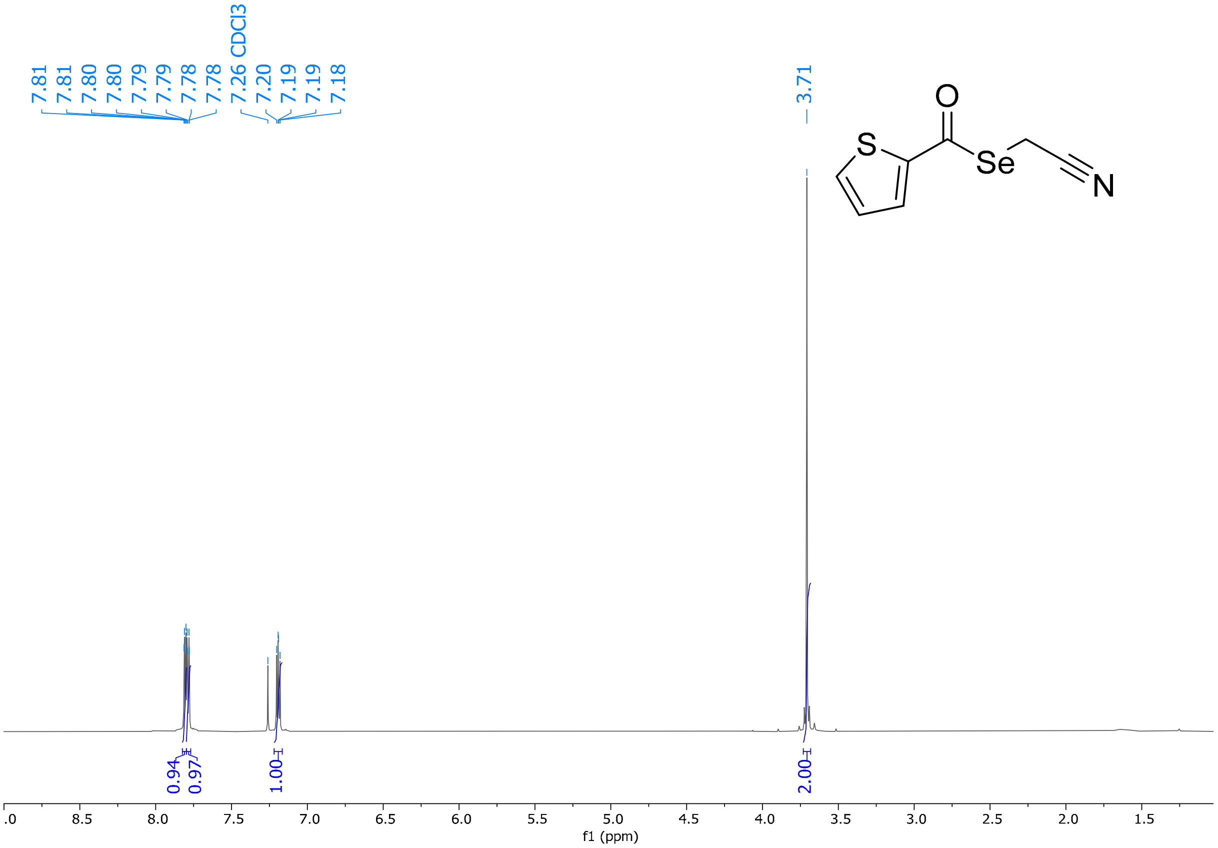

Figure S9C. ^{1}H-NMR spectrum ($CDCl_3$, 400 MHz) of **N1**.

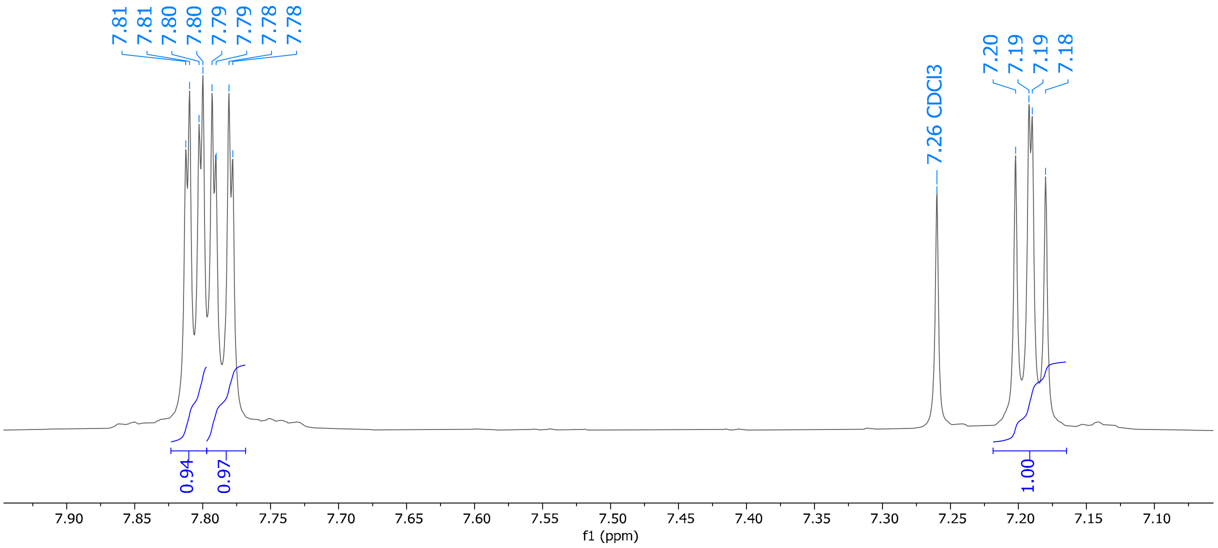

Figure S9D. ^{1}H-NMR spectrum ($CDCl_3$, 400 MHz) of **N1** (aromatics).

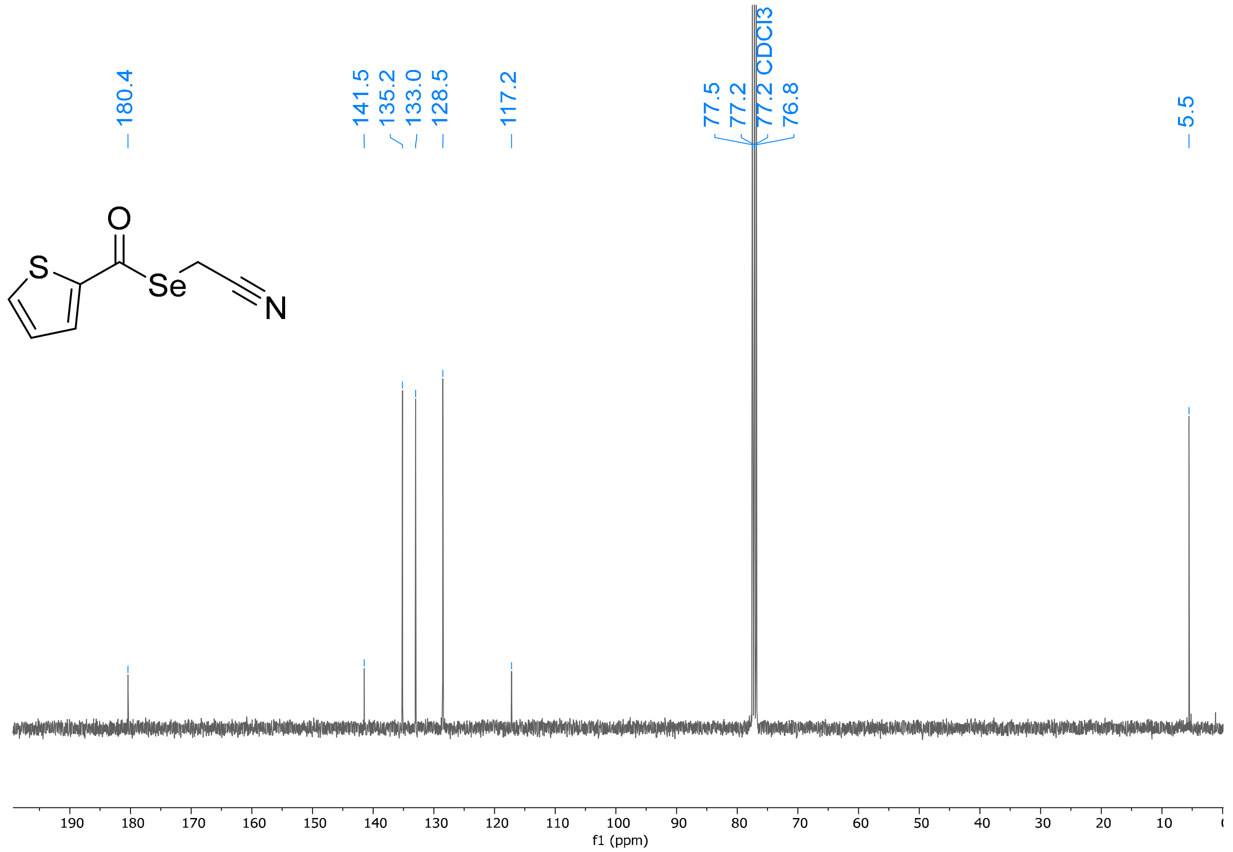

Figure S9E. ^{13}C-NMR spectrum ($CDCl_3$, 101 MHz) of **N1**.

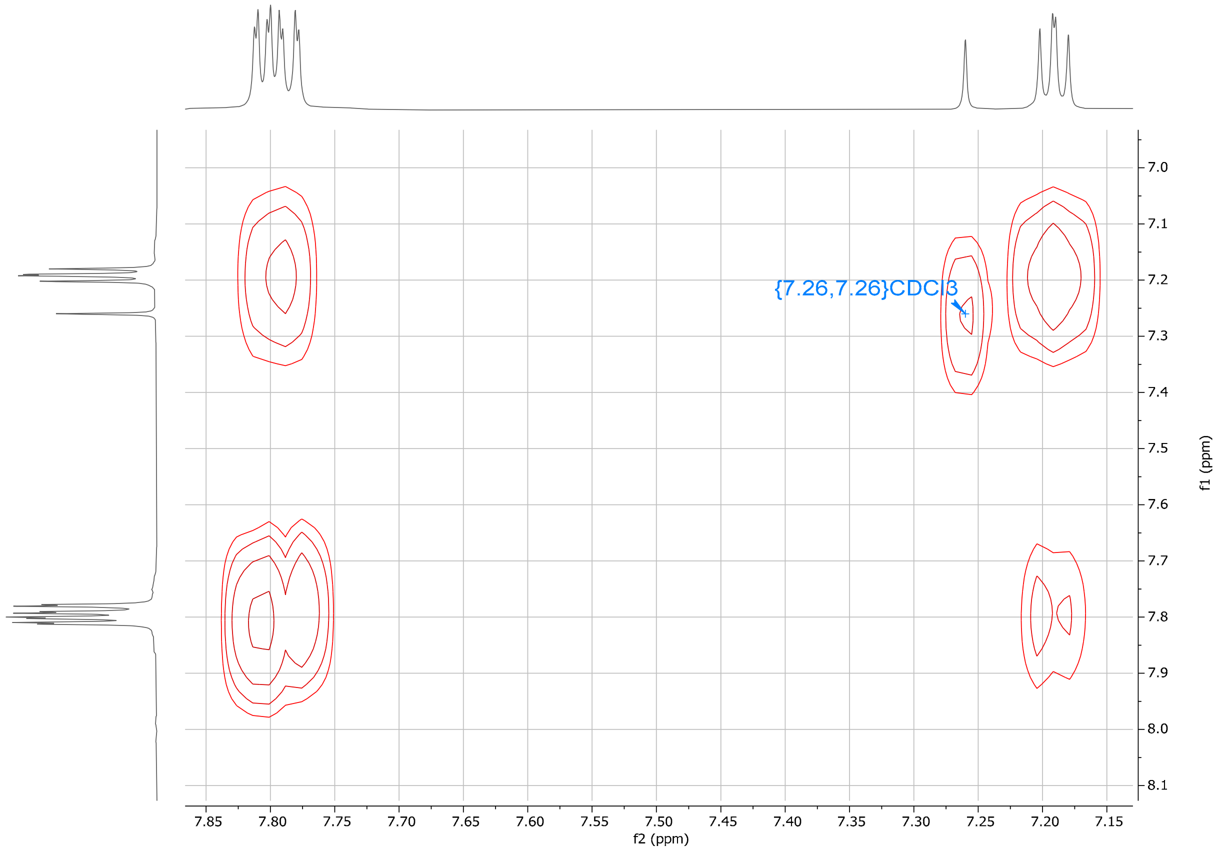

Figure S9F. ^{1}H-^{1}H COSY NMR spectrum ($CDCl_3$) of **N1** (aromatics).

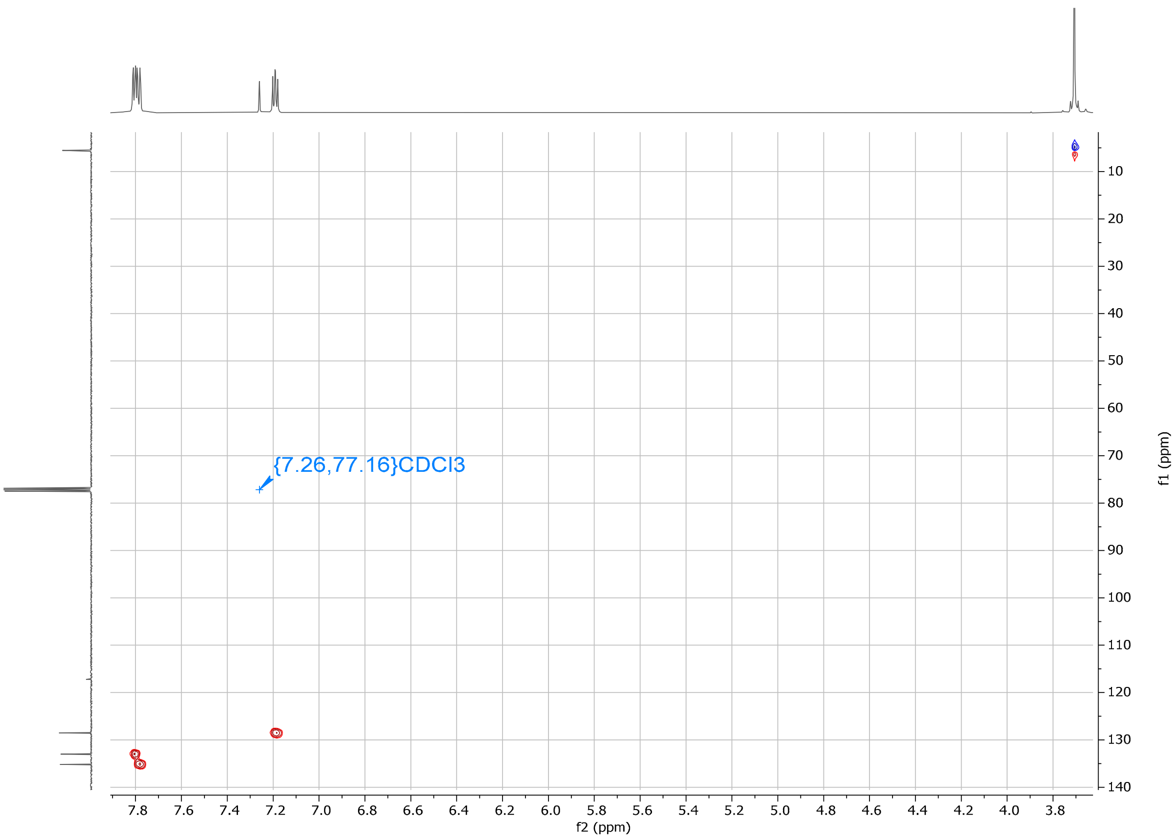

Figure S9G. ^{1}H-^{13}C HSQC NMR spectrum ($CDCl_3$) of **N1**.

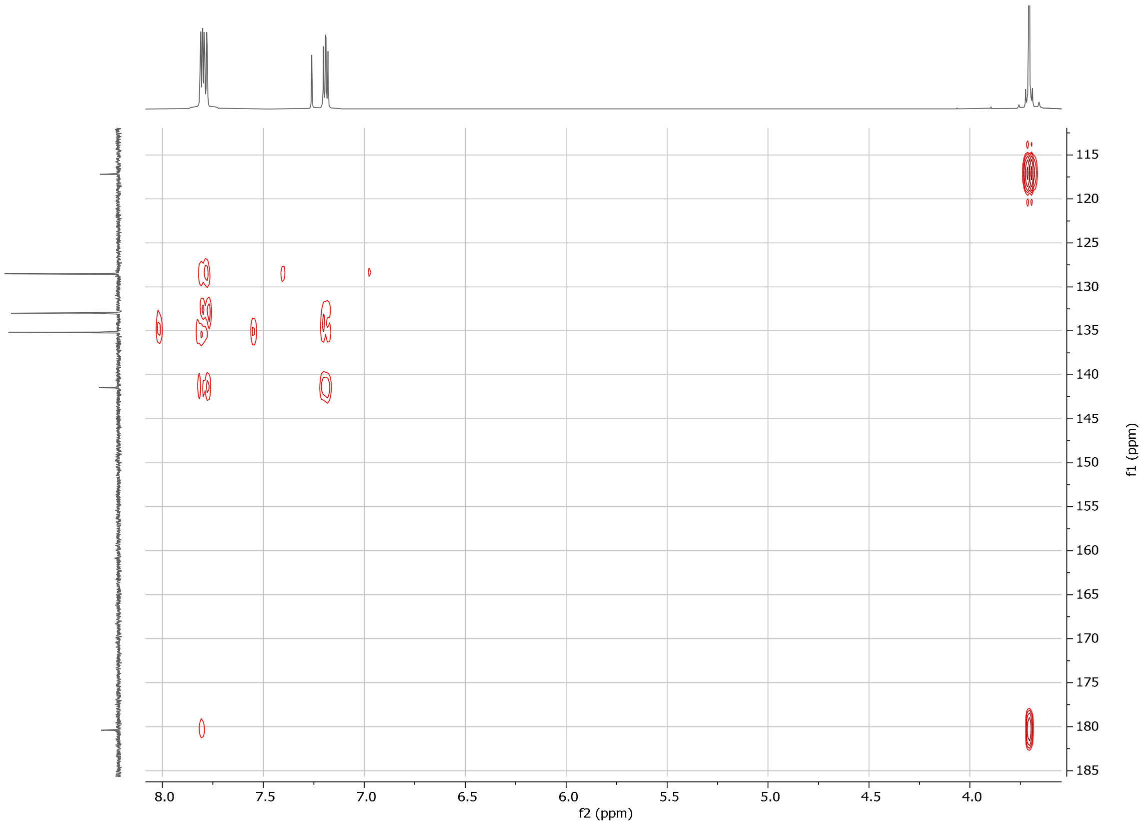

Figure S9H. ^{1}H-^{13}C HMBC NMR spectrum ($CDCl_3$) of **N1**.

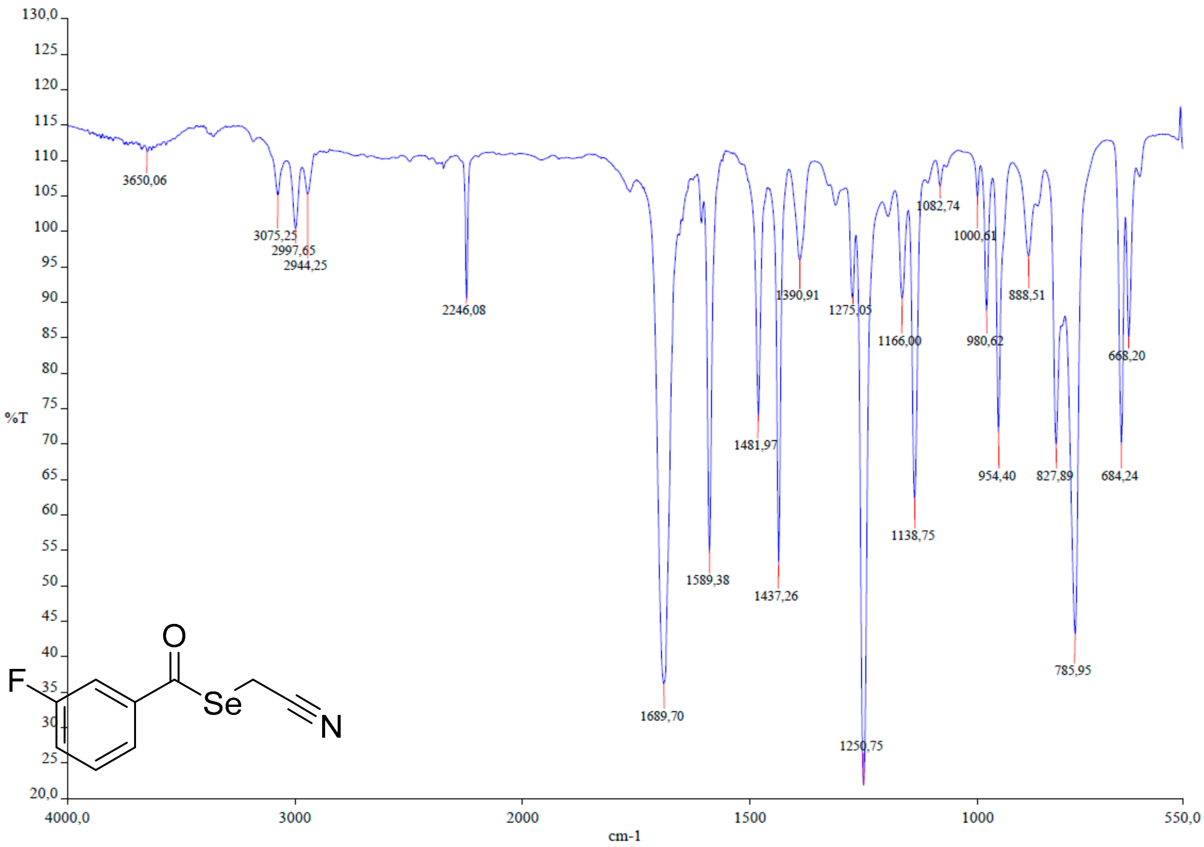

Figure S10. Compound **N2**: Se-(cyanomethyl) 3-fluorobenzoselenoate. S10**A**. IR spectrum (NaCl) of **N2**.

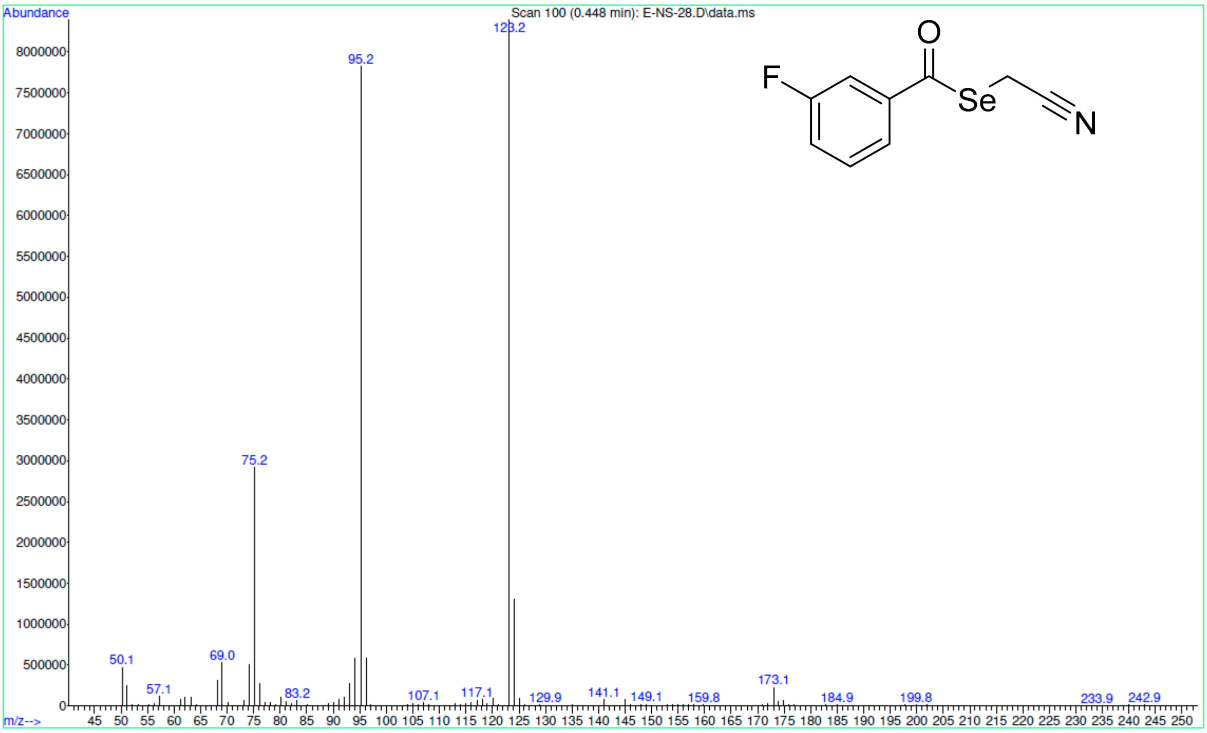

Figure S10B. DIP-MS spectrum of **N2**.

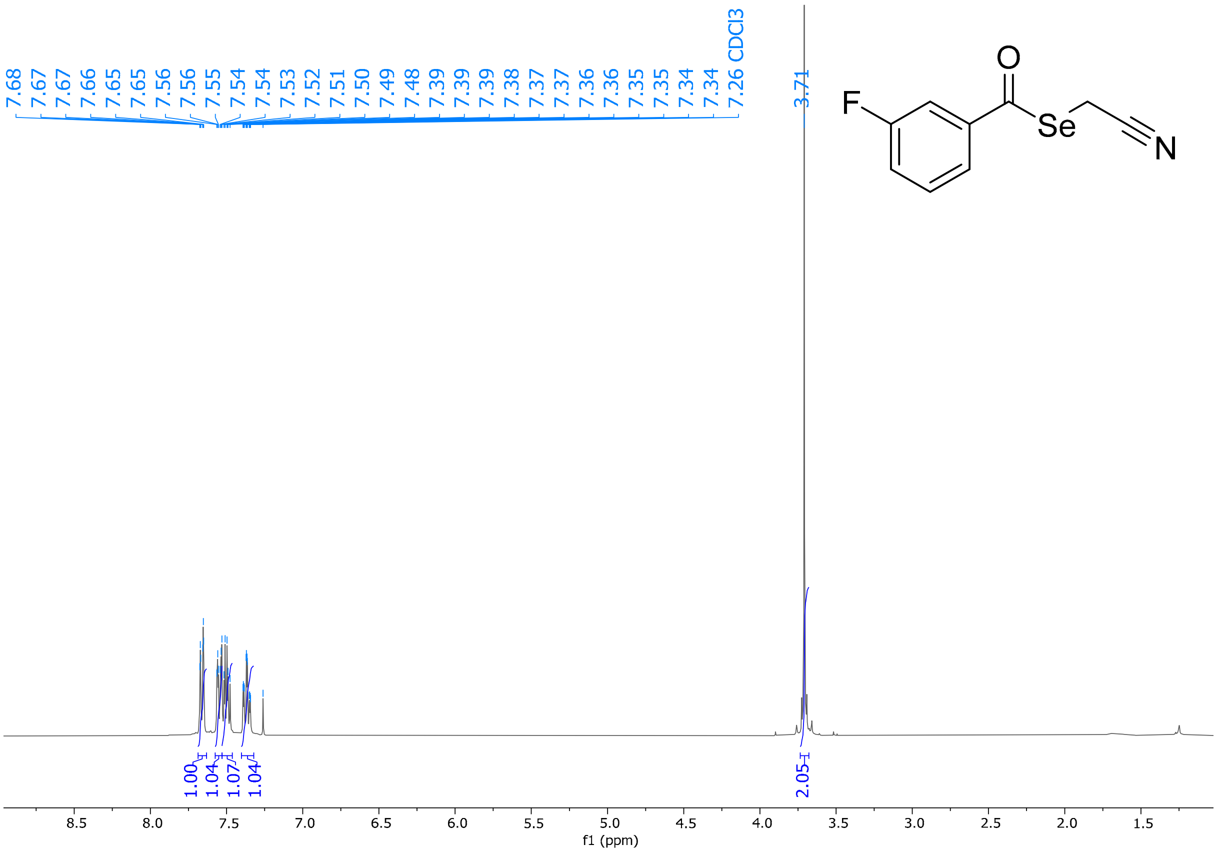

Figure S10C. ^{1}H-NMR spectrum ($CDCl_3$, 400 MHz) of **N2**.

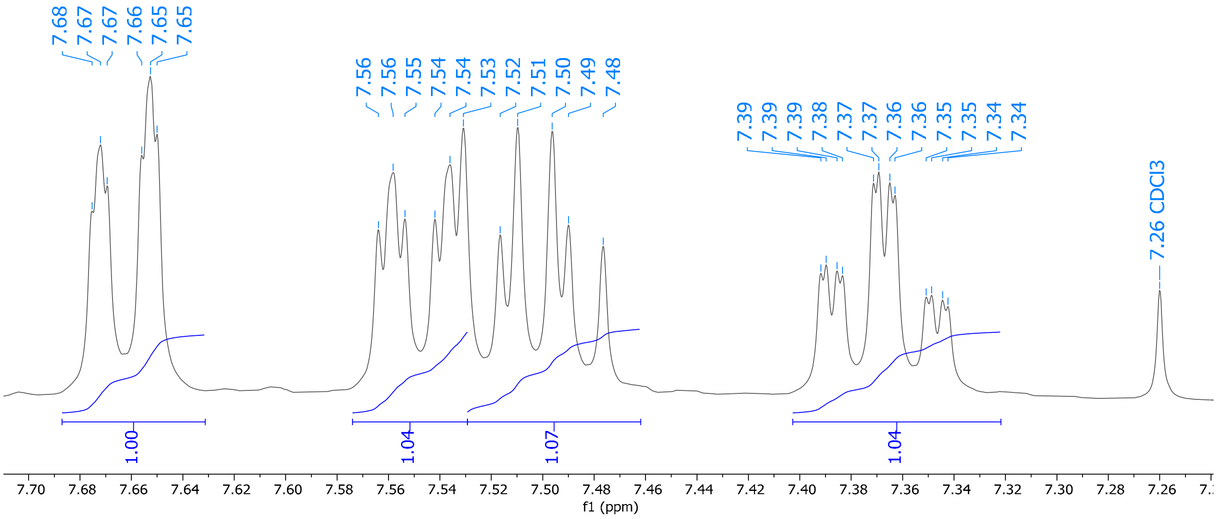

Figure S10D. ^{1}H-NMR spectrum ($CDCl_3$, 400 MHz) of **N2** (aromatics).

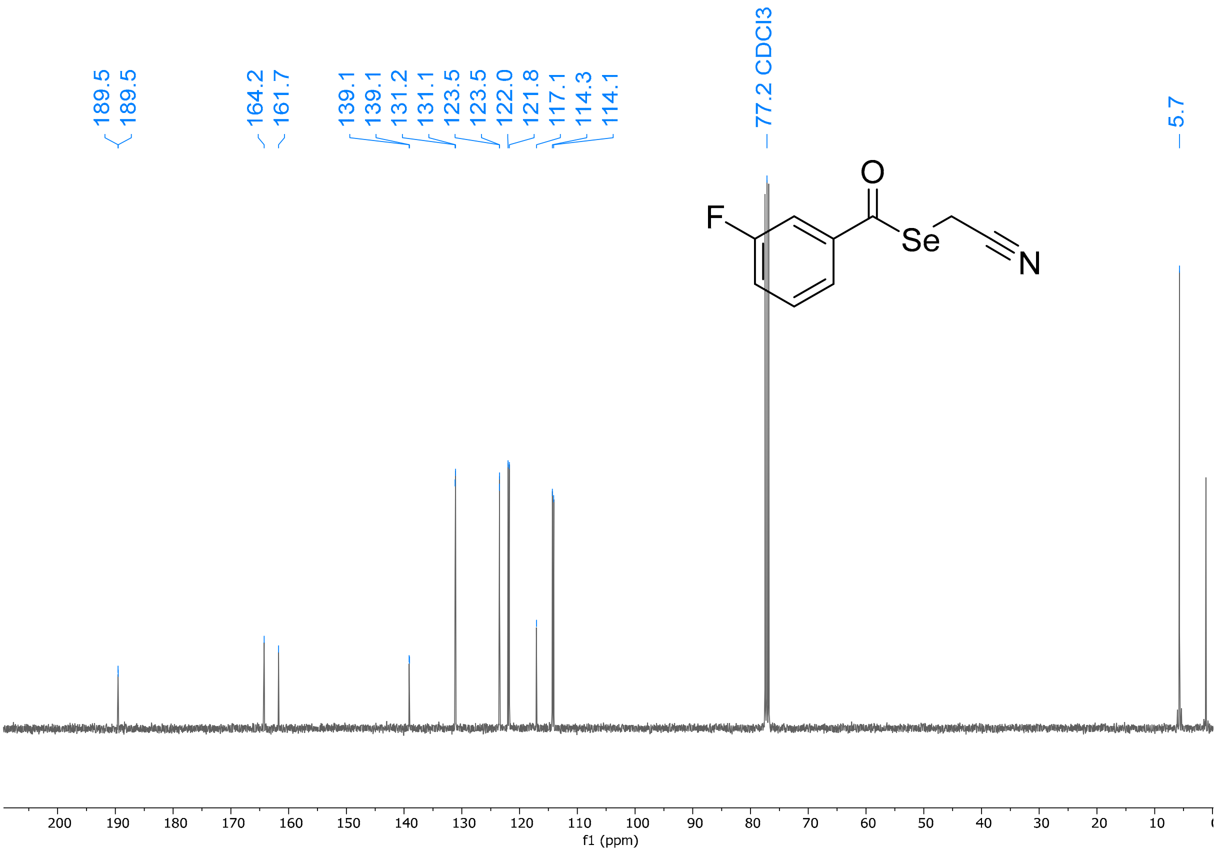

Figure S10E. ^{13}C-NMR spectrum ($CDCl_3$, 101 MHz) of **N2**.

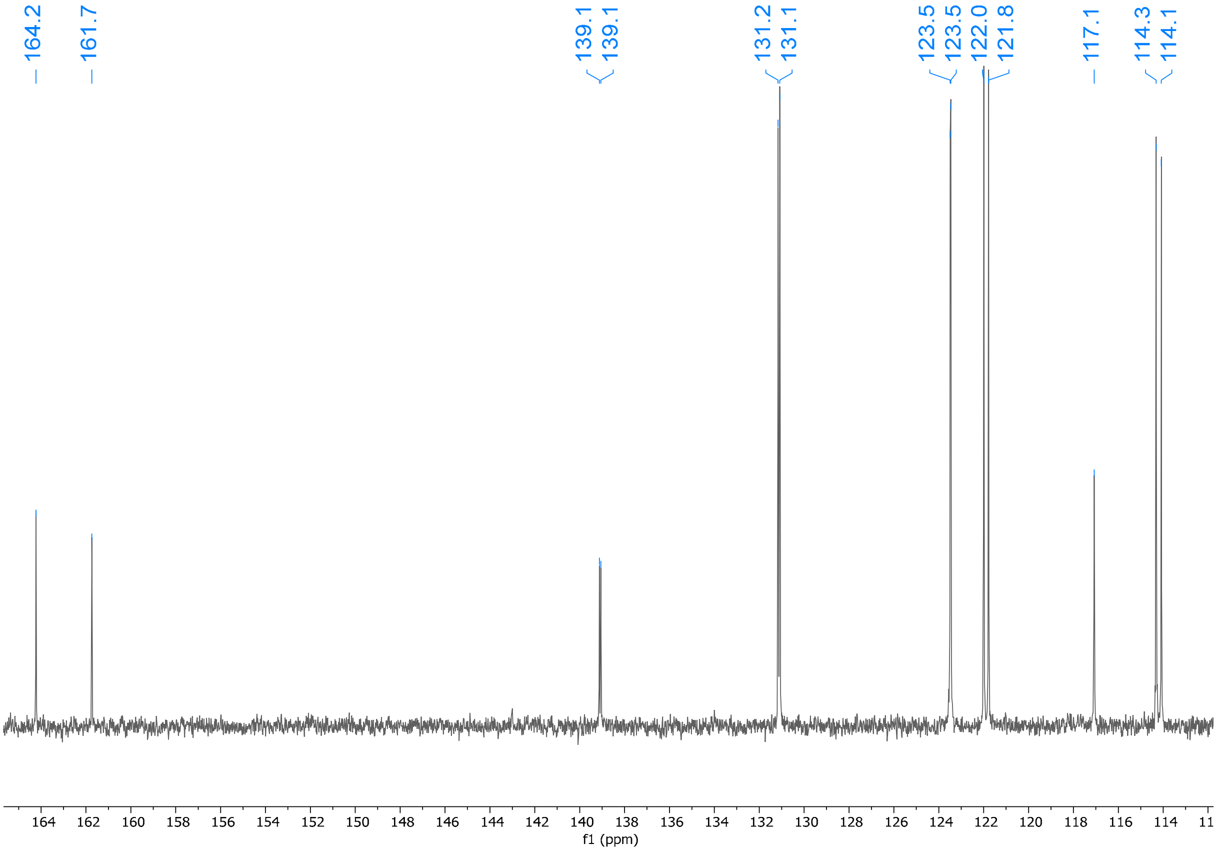

Figure S10F. ^{13}C-NMR spectrum ($CDCl_3$, 101 MHz) of **N2** (aromatics, CN).

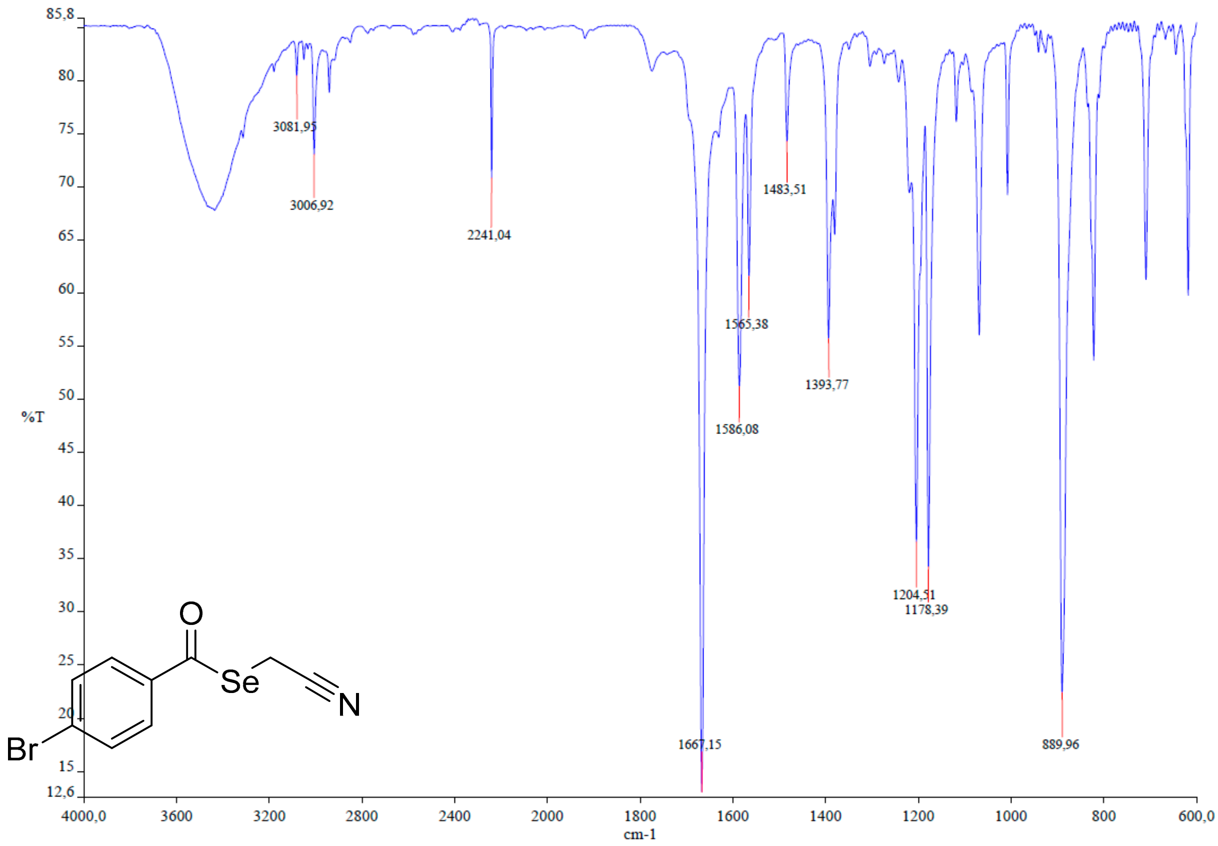

Figure S11. Compound **N3**: Se-(cyanomethyl) 4-bromobenzoselenoate. S11**A**. IR spectrum (KBr) of **N3**.

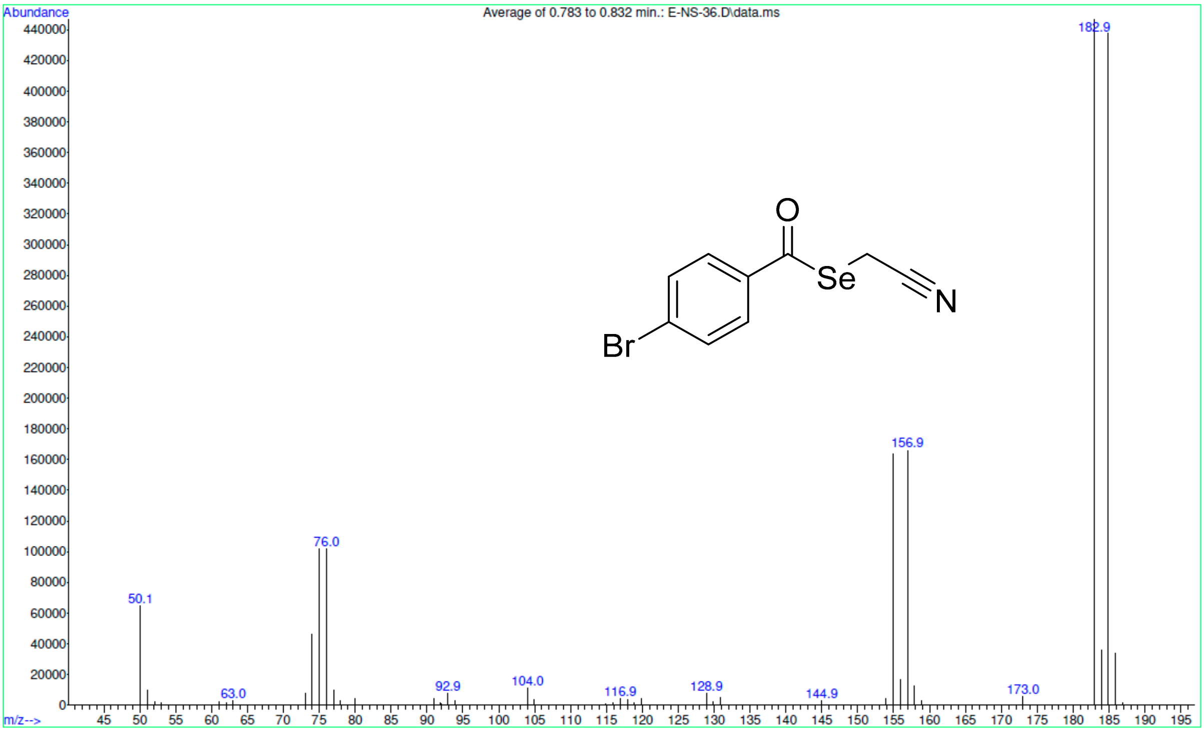

Figure S11B. DIP-MS spectrum of **N3**.

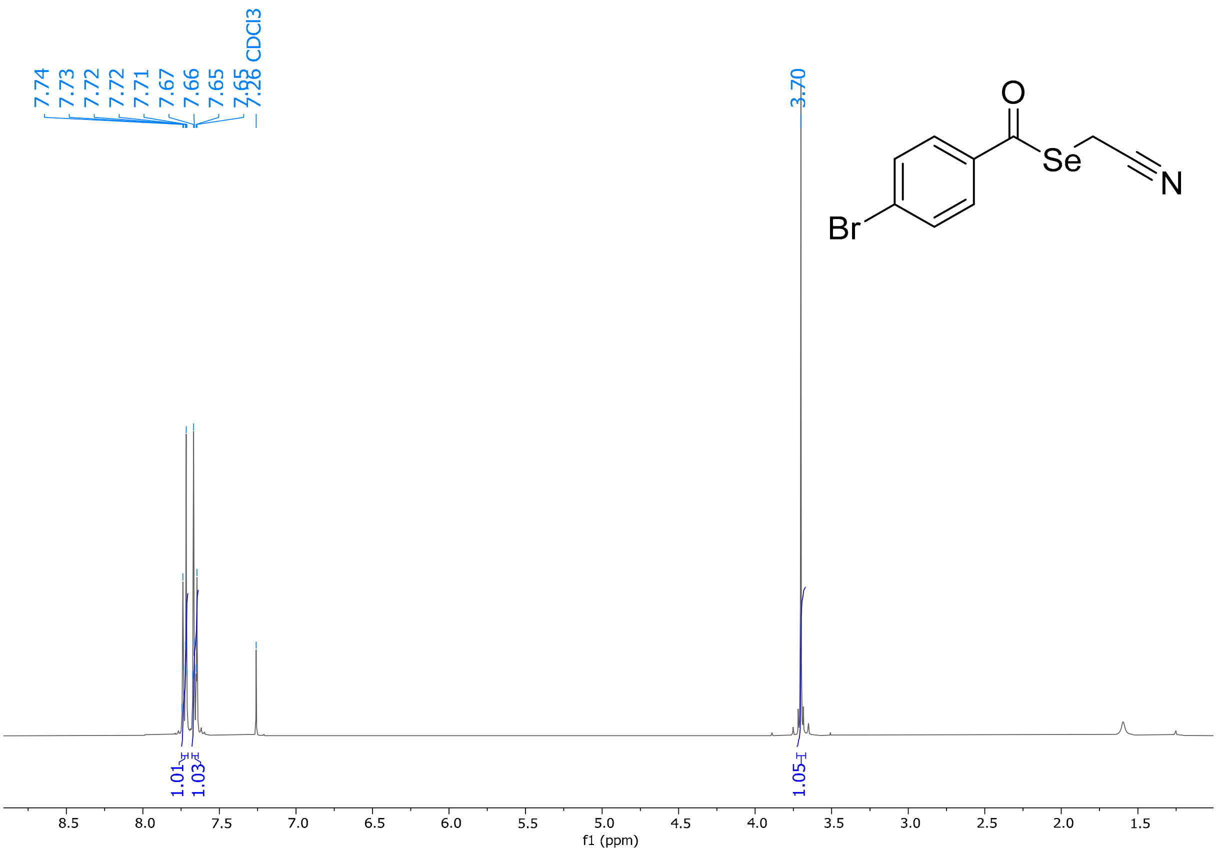

Figure S11C. ^{1}H-NMR spectrum ($CDCl_3$, 400 MHz) of **N3**.

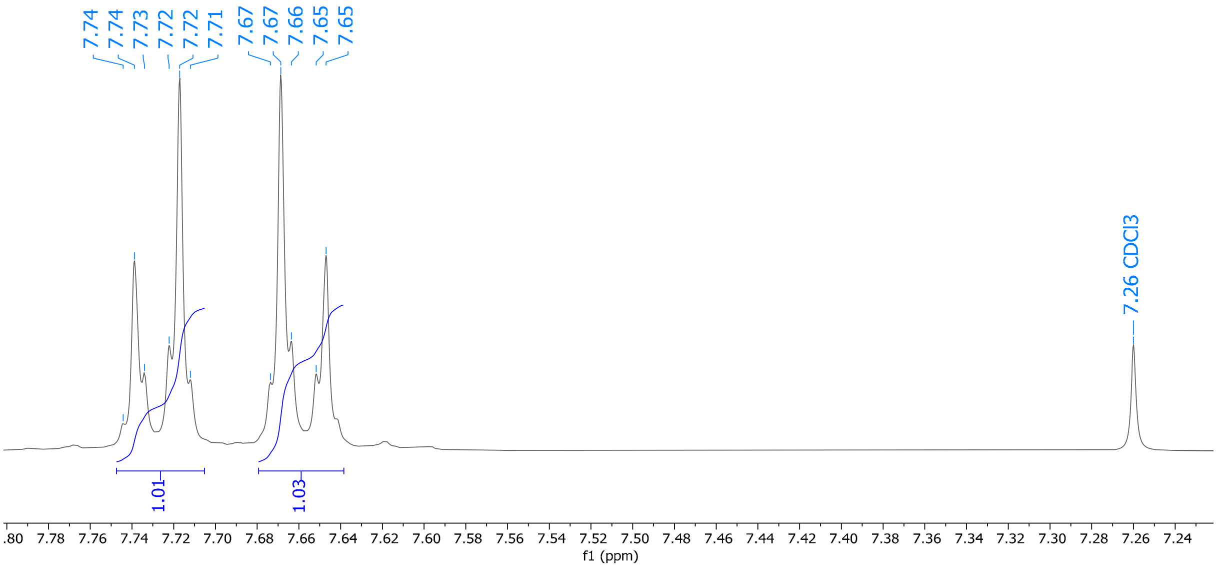

Figure S11D. ^{1}H-NMR spectrum ($CDCl_3$, 400 MHz) of **N3** (aromatics).

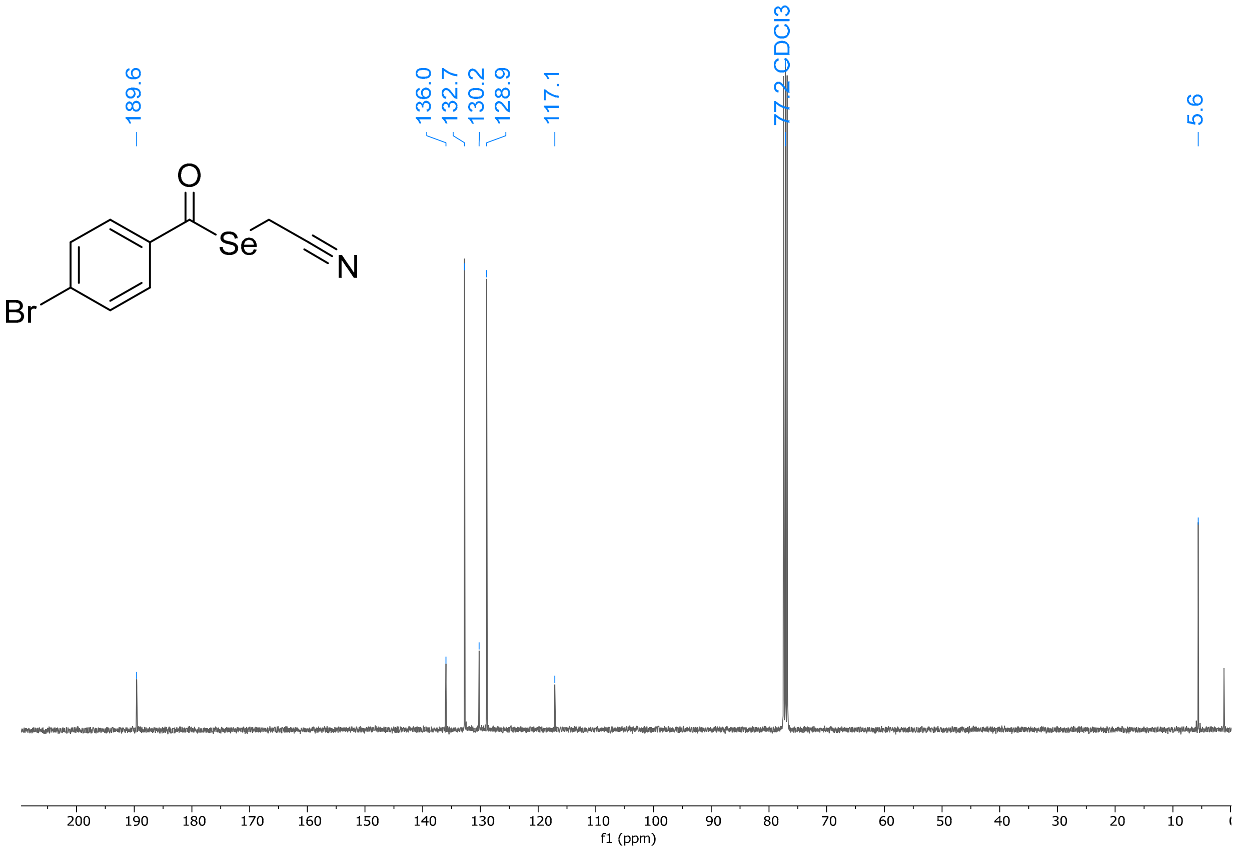

Figure S11E. ^{13}C-NMR spectrum ($CDCl_3$, 101 MHz) of **N3**.

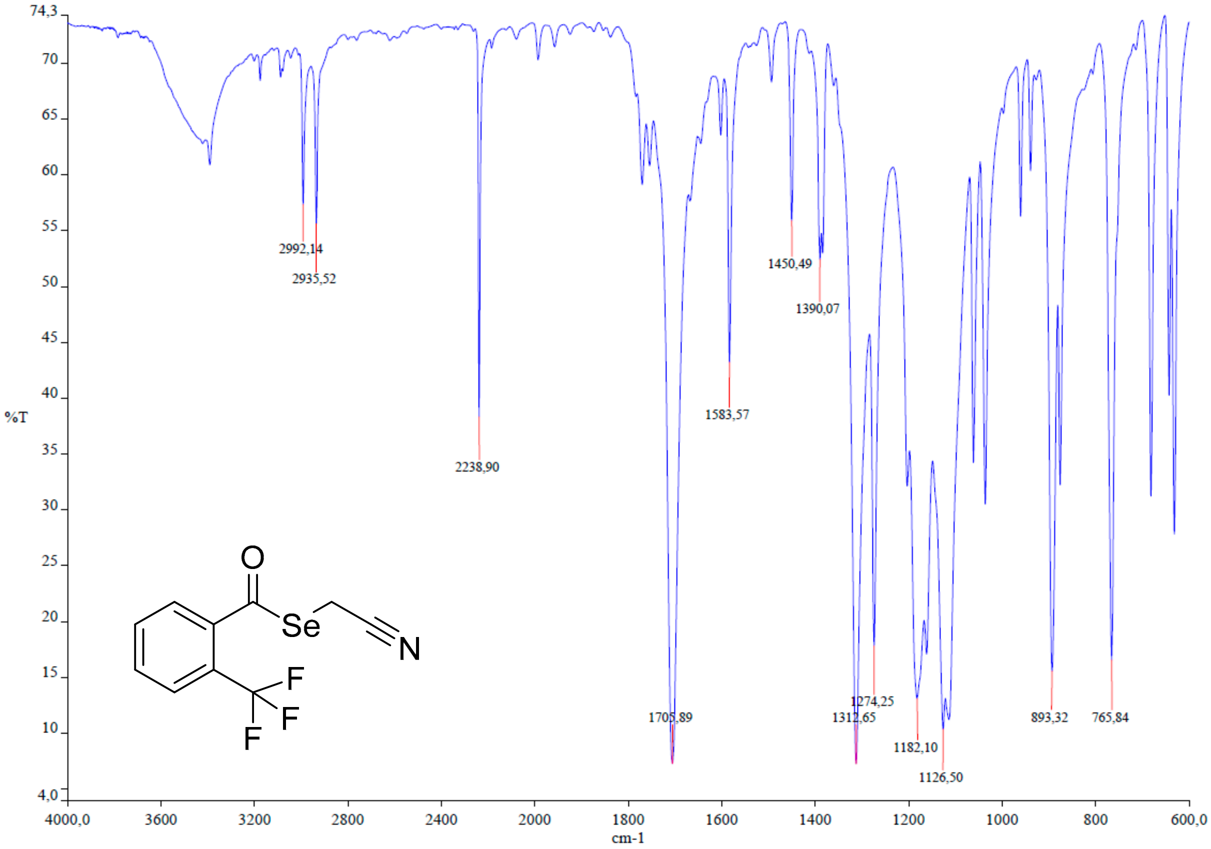

Figure S12. Compound **N4**: Se-(cyanomethyl) 2-(trifluoromethyl)benzoselenoate. S12**A**. IR spectrum (KBr) of **N4**.

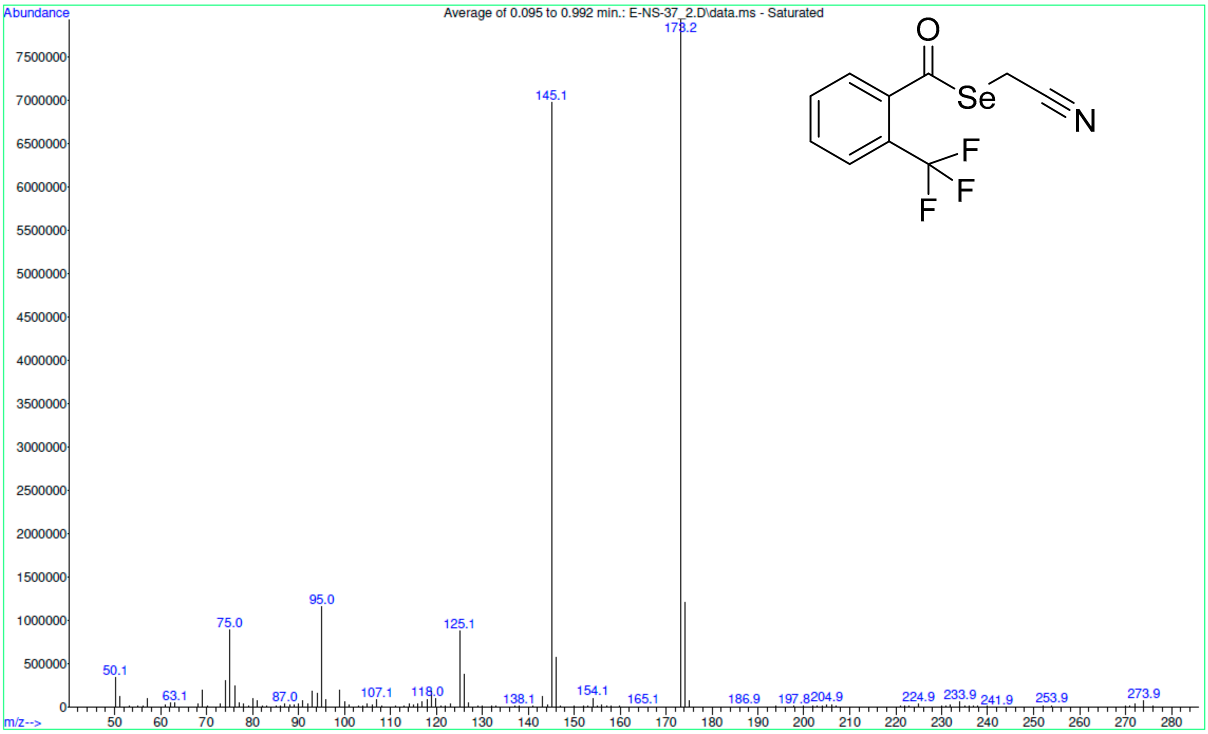

Figure S12B. DIP-MS spectrum of **N4**.

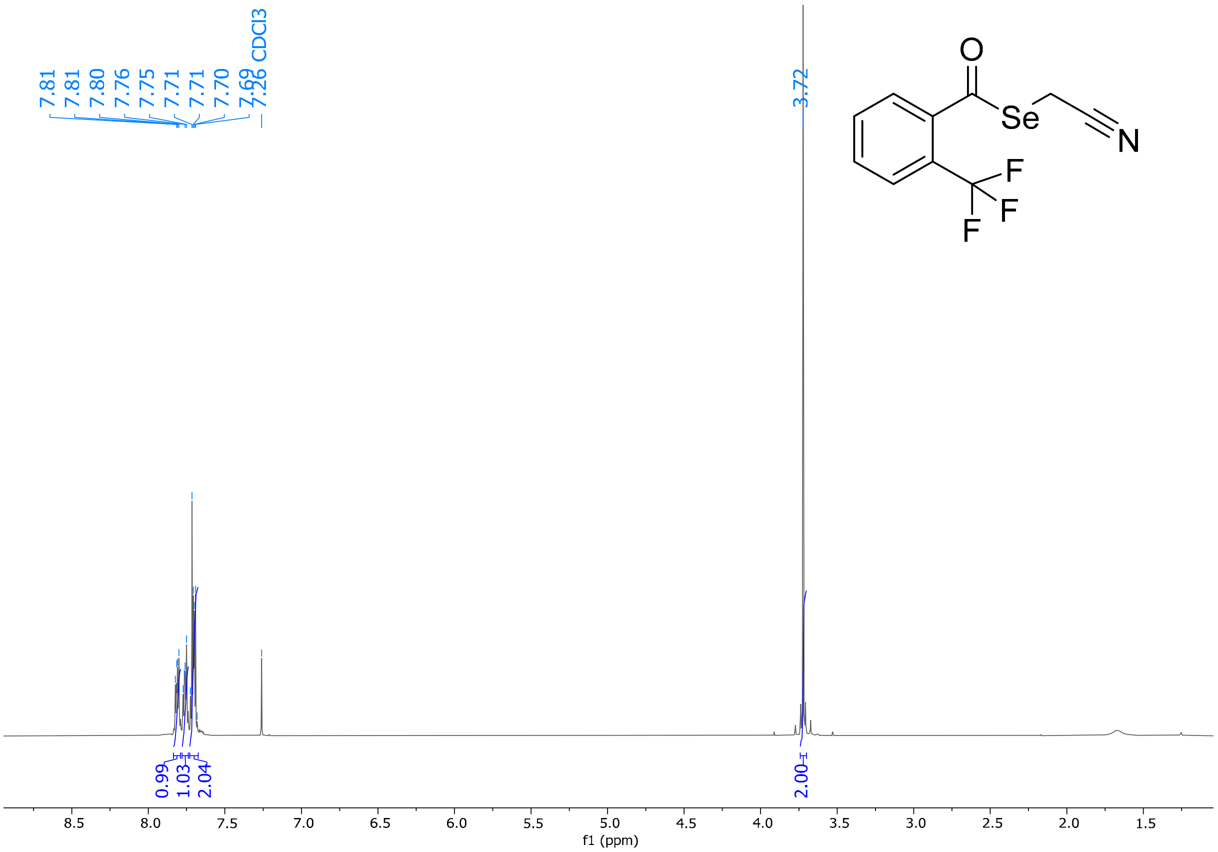

Figure S12C. ^{1}H-NMR spectrum ($CDCl_3$, 400 MHz) of **N4**.

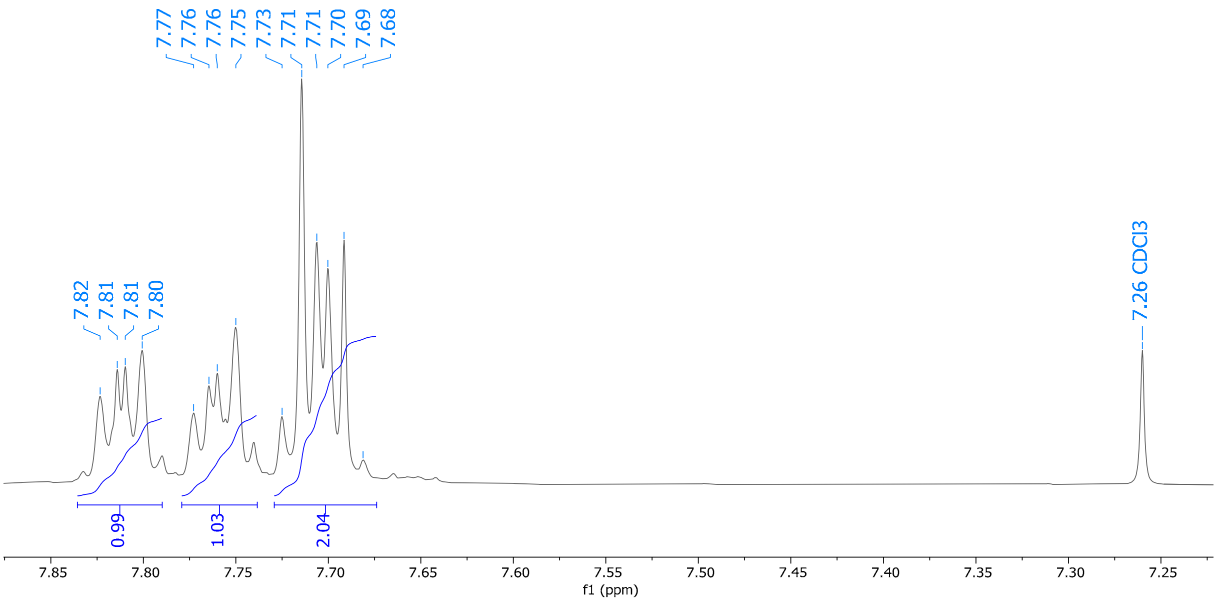

Figure S12D. ^{1}H-NMR spectrum ($CDCl_3$, 400 MHz) of **N4** (aromatics).

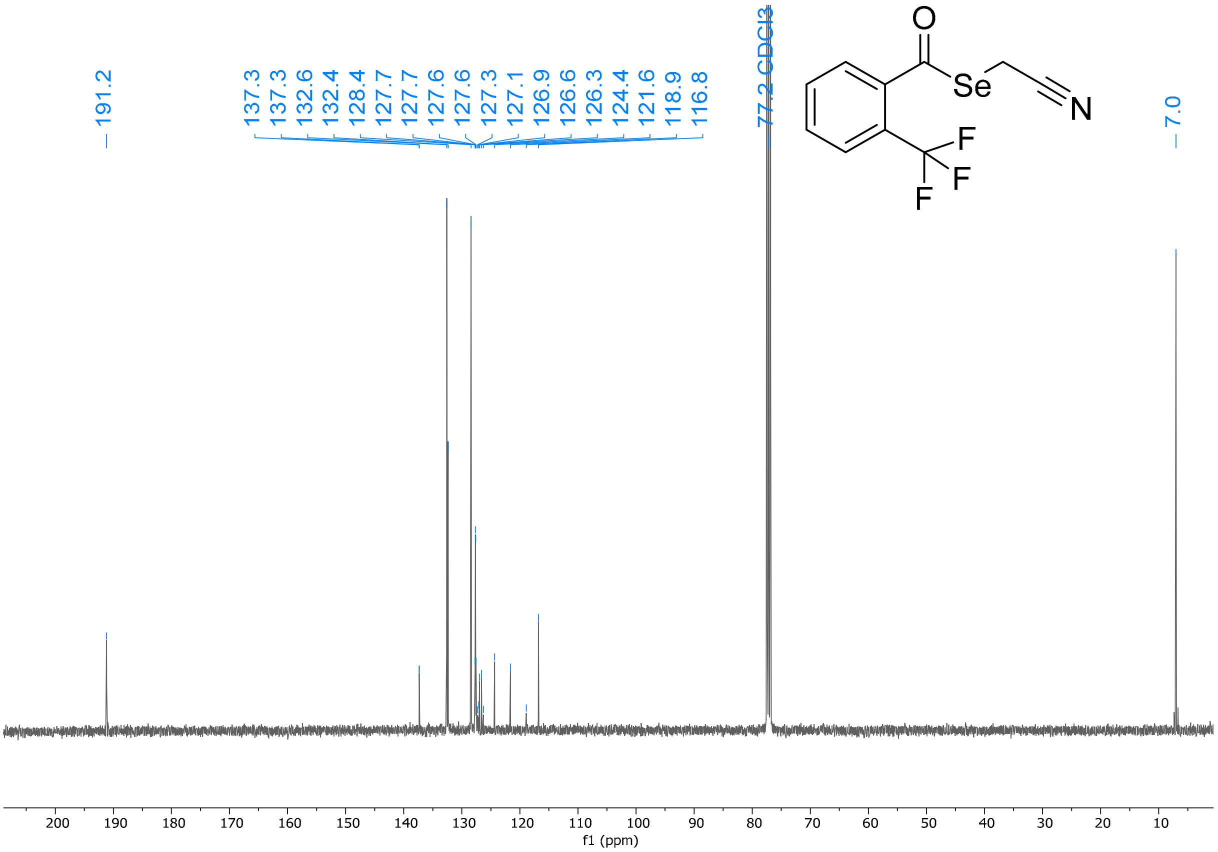

Figure S12E. ^{13}C-NMR spectrum ($CDCl_3$, 101 MHz) of **N4**.

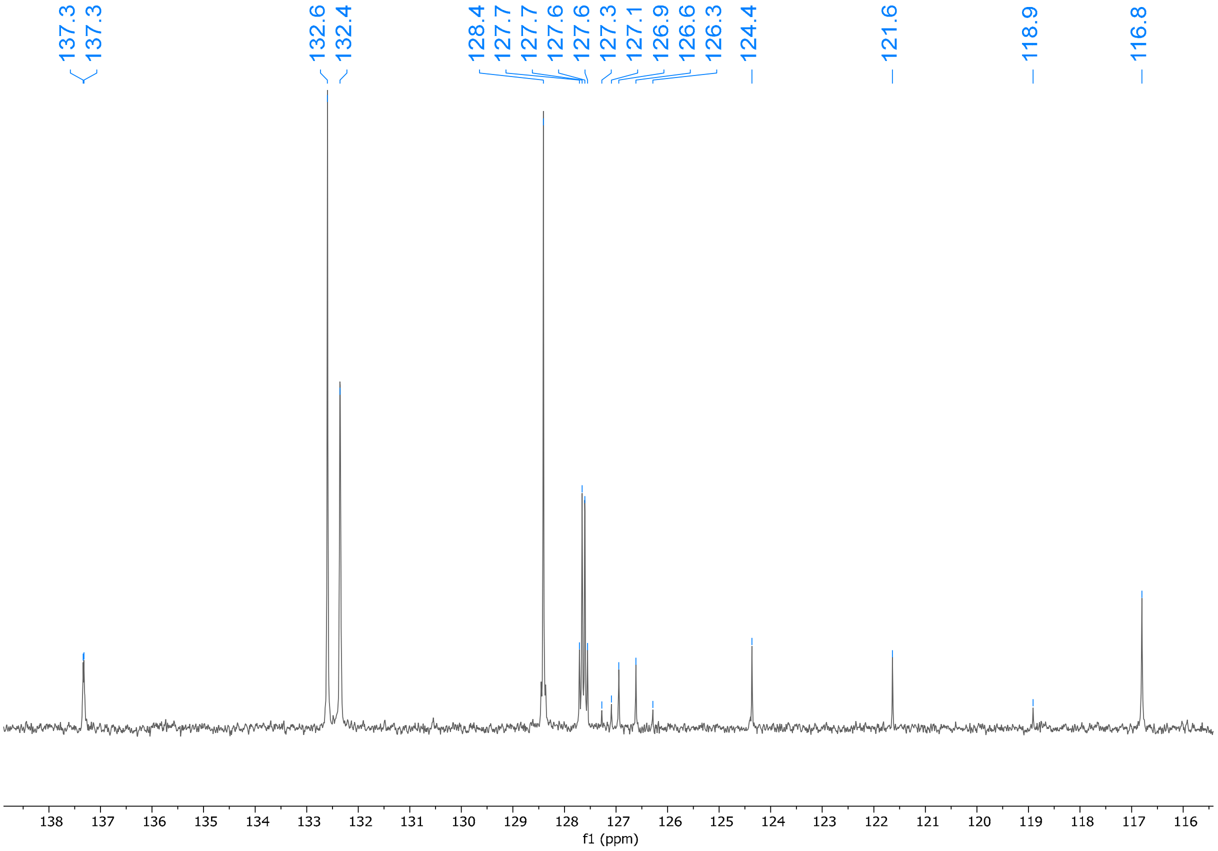

Figure S12F. ^{13}C-NMR spectrum ($CDCl_3$, 101 MHz) of **N4** (aromatics, CF_3, CN).

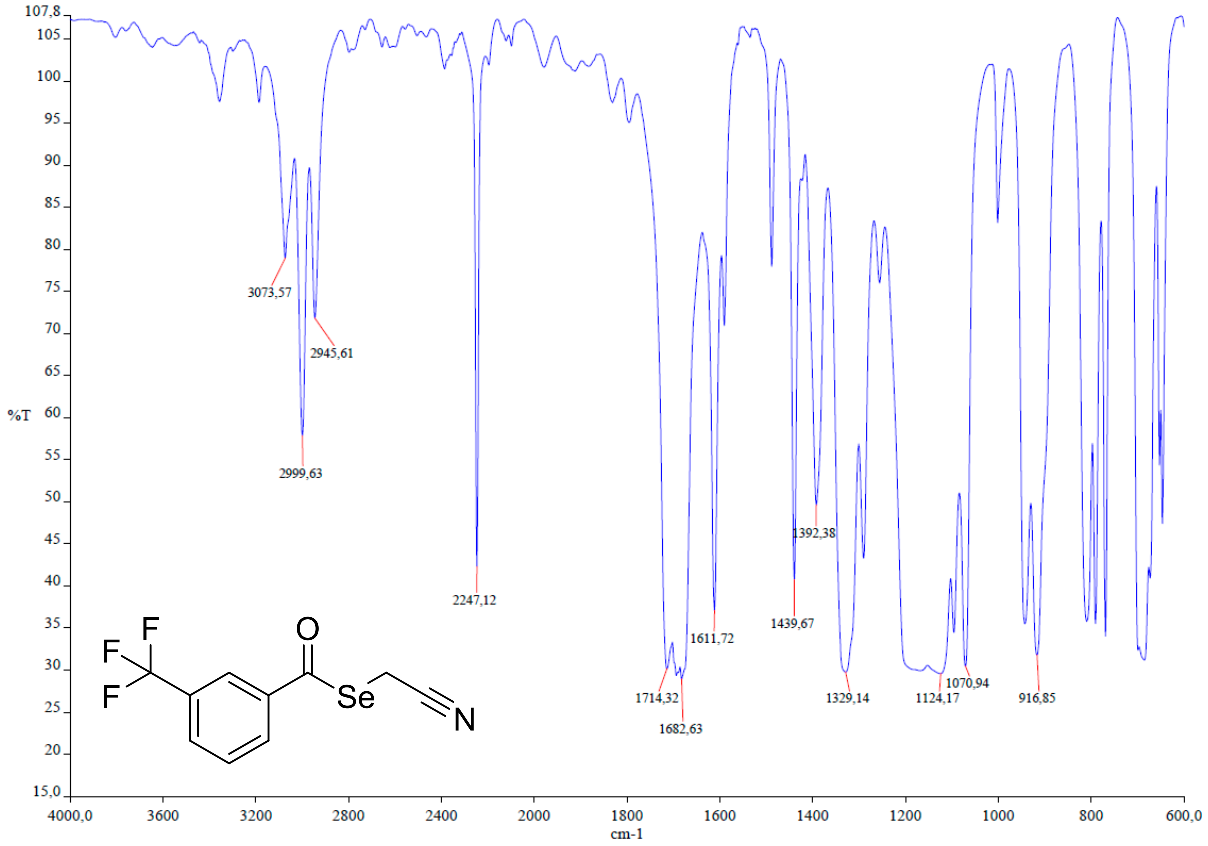

Figure S13. Compound **N5**: Se-(cyanomethyl) 3-(trifluoromethyl)benzoselenoate. S13**A**. IR spectrum (KBr) of **N5**.

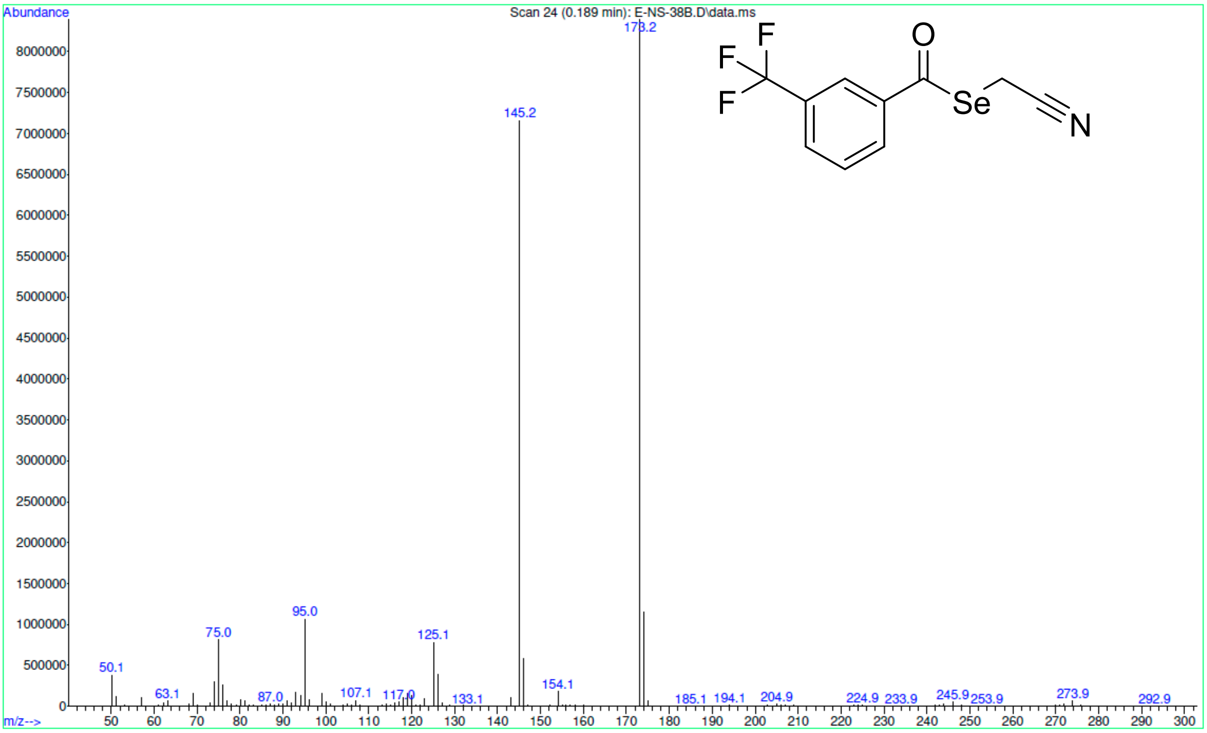

Figure S13B. DIP-MS spectrum of **N5**.

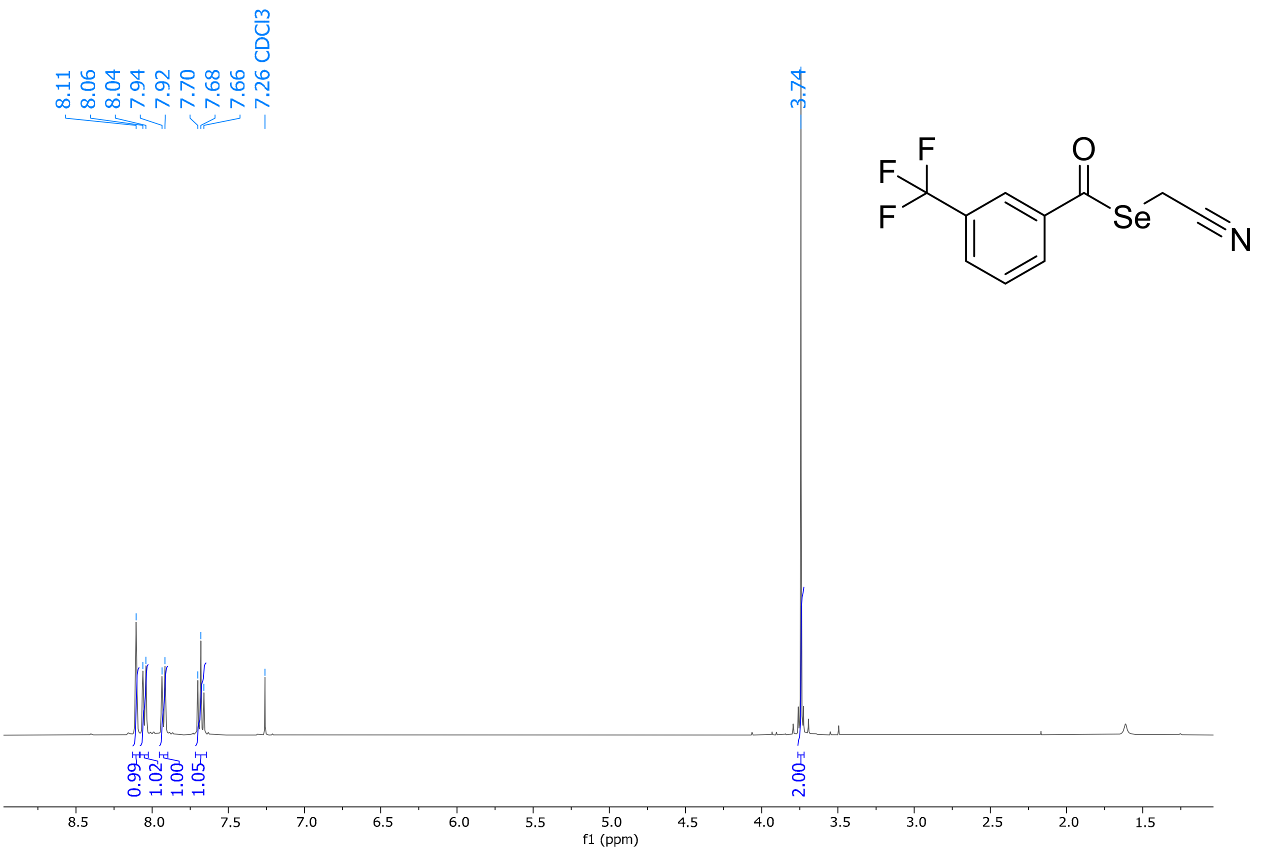

Figure S13C. ^{1}H-NMR spectrum ($CDCl_3$, 400 MHz) of **N5**.

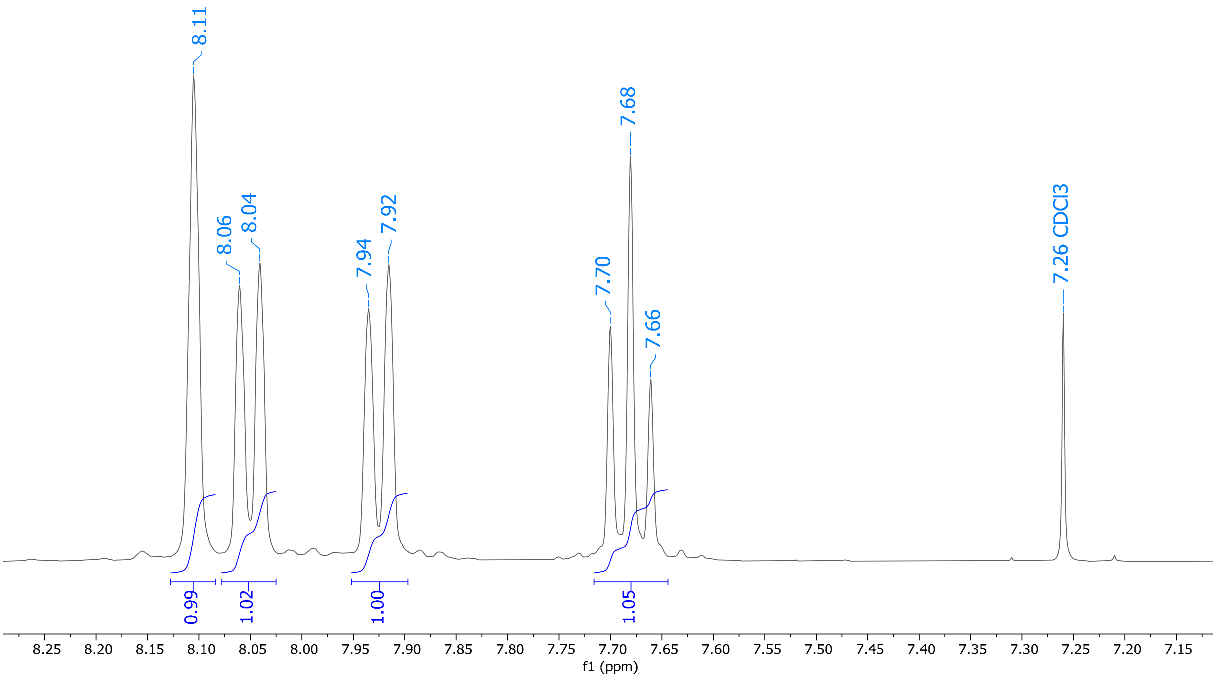

Figure S13D. ^{1}H-NMR spectrum ($CDCl_3$, 400 MHz) of **N5** (aromatics).

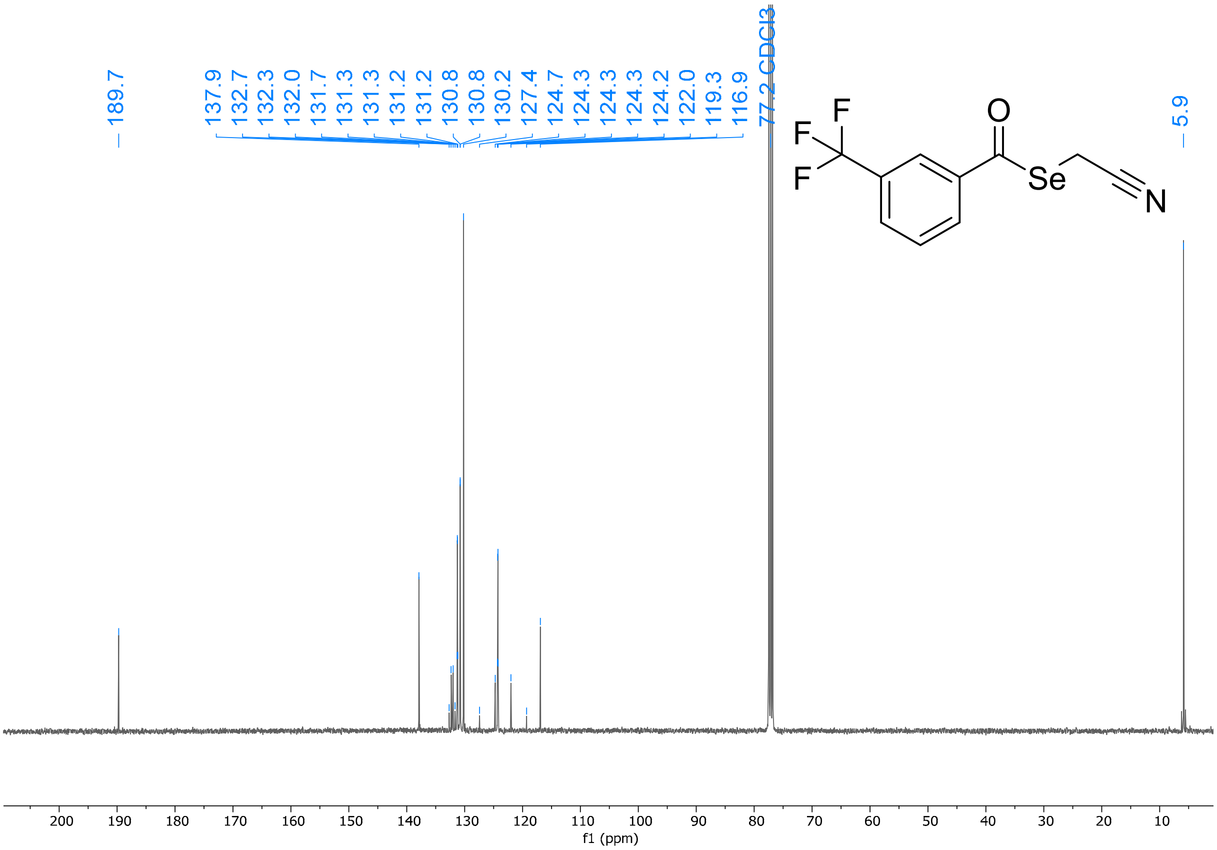

Figure S13E. ^{13}C-NMR spectrum ($CDCl_3$, 101 MHz) of **N5**.

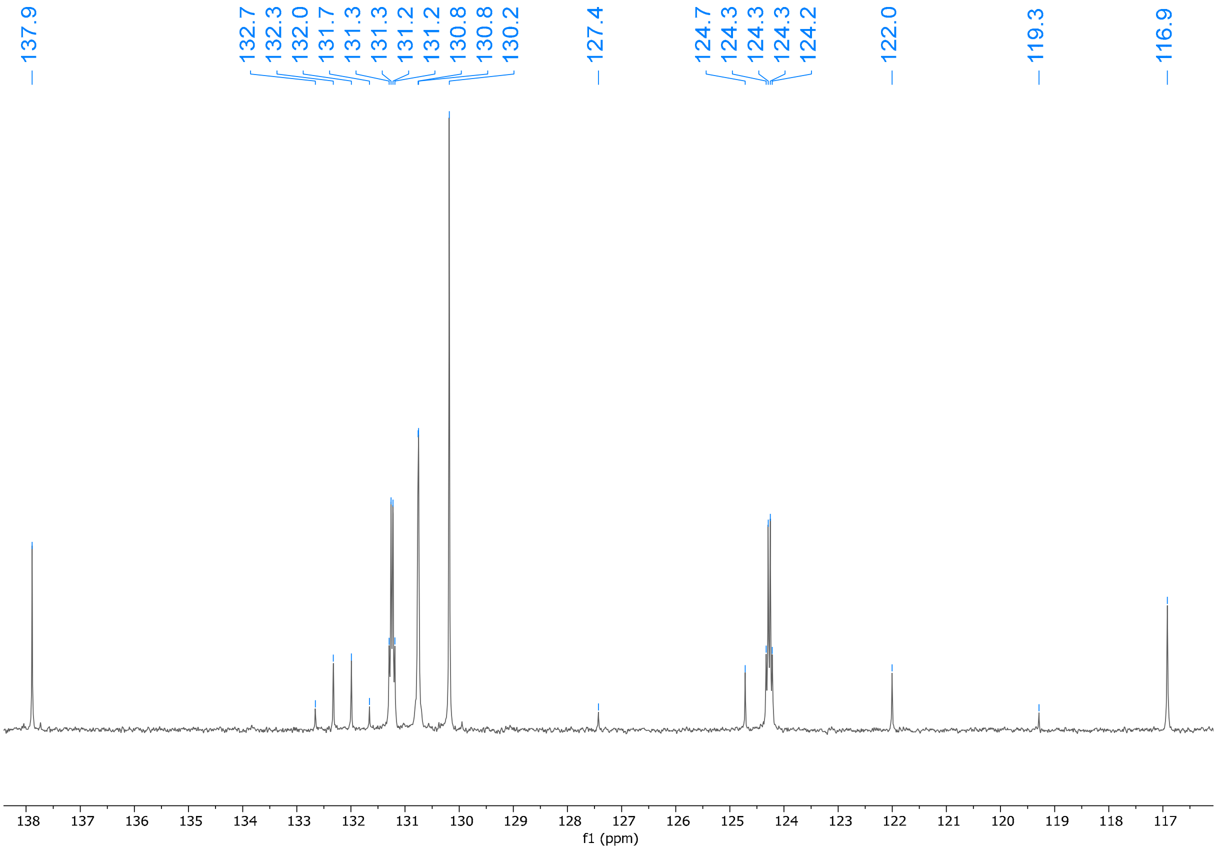

Figure S13F. ^{13}C-NMR spectrum ($CDCl_3$, 101 MHz) of **N5** (aromatics, CF_3, CN).

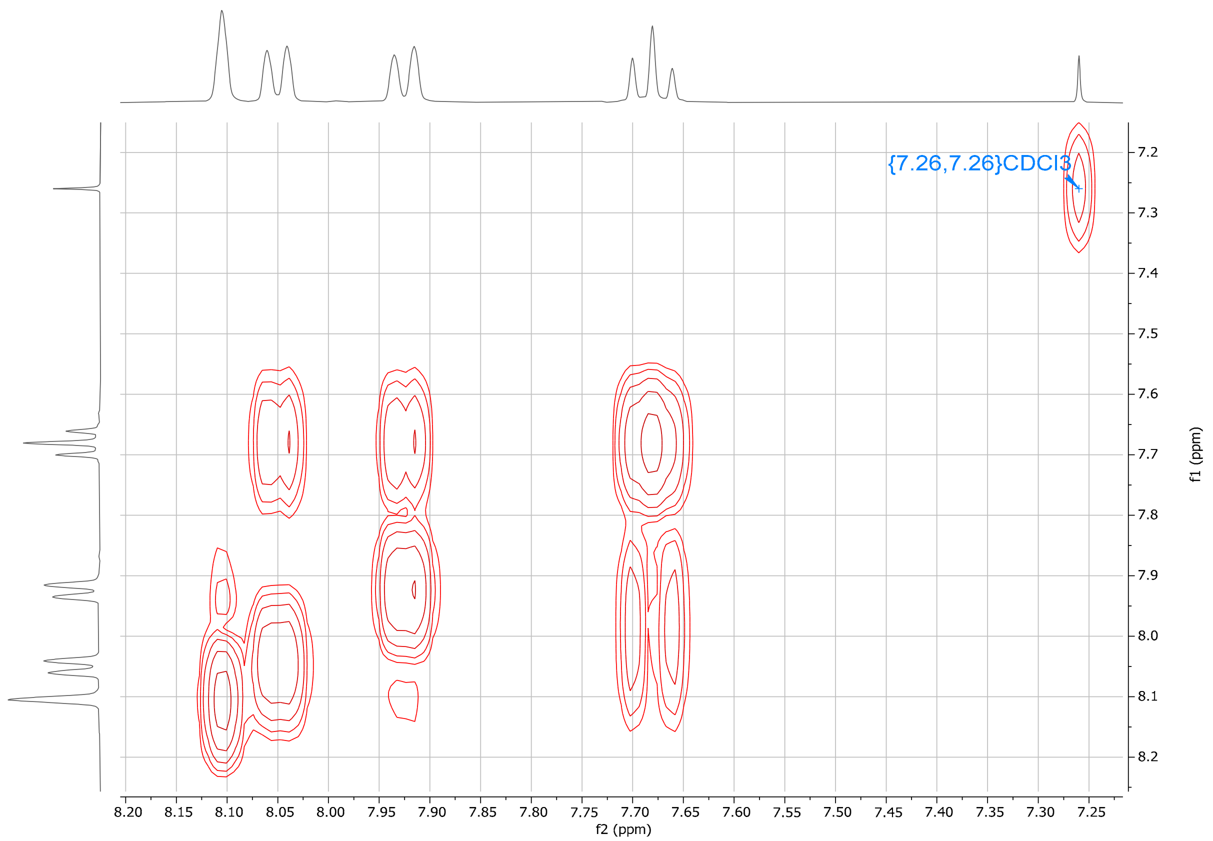

Figure S13G. ^{1}H-^{1}H COSY NMR spectrum ($CDCl_3$) of **N5** (aromatics).

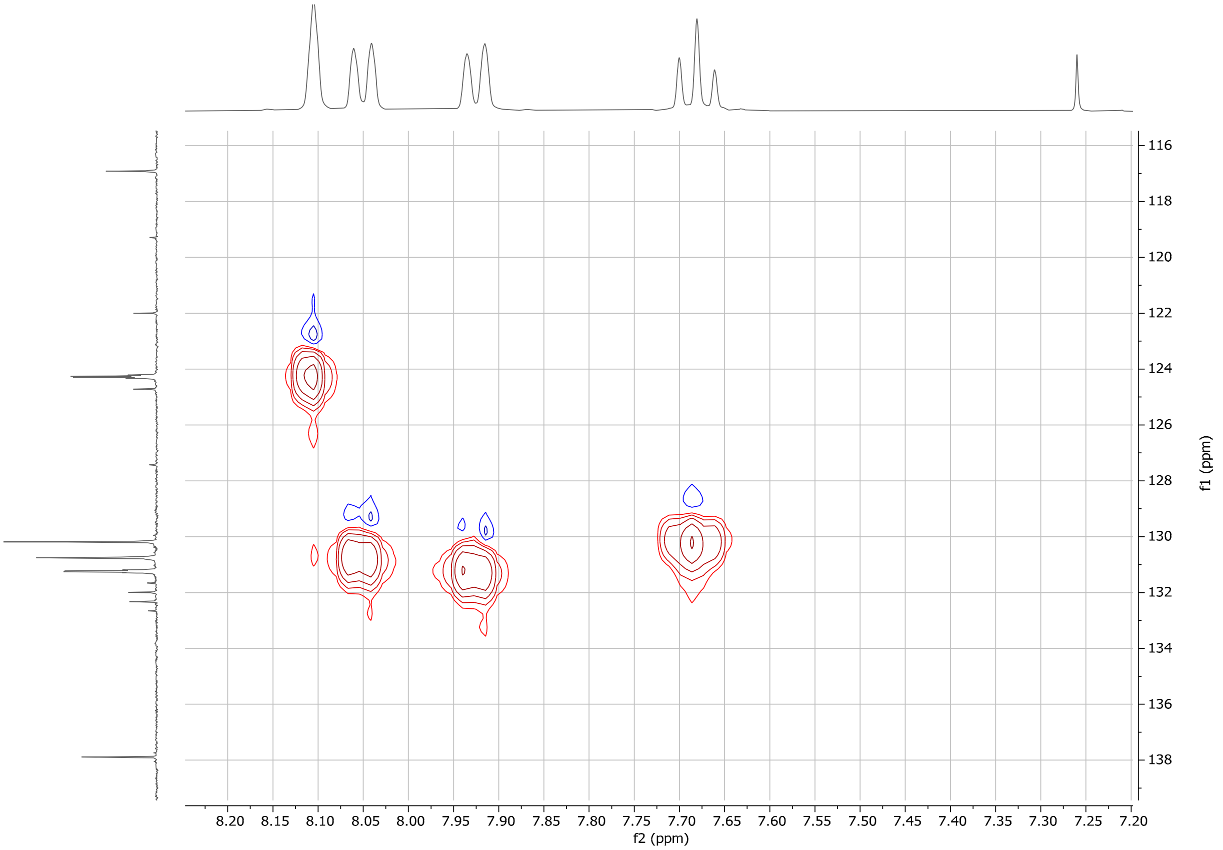

Figure S13H. ^{1}H-^{13}C HSQC NMR spectrum ($CDCl_3$) of **N5** (aromatics).

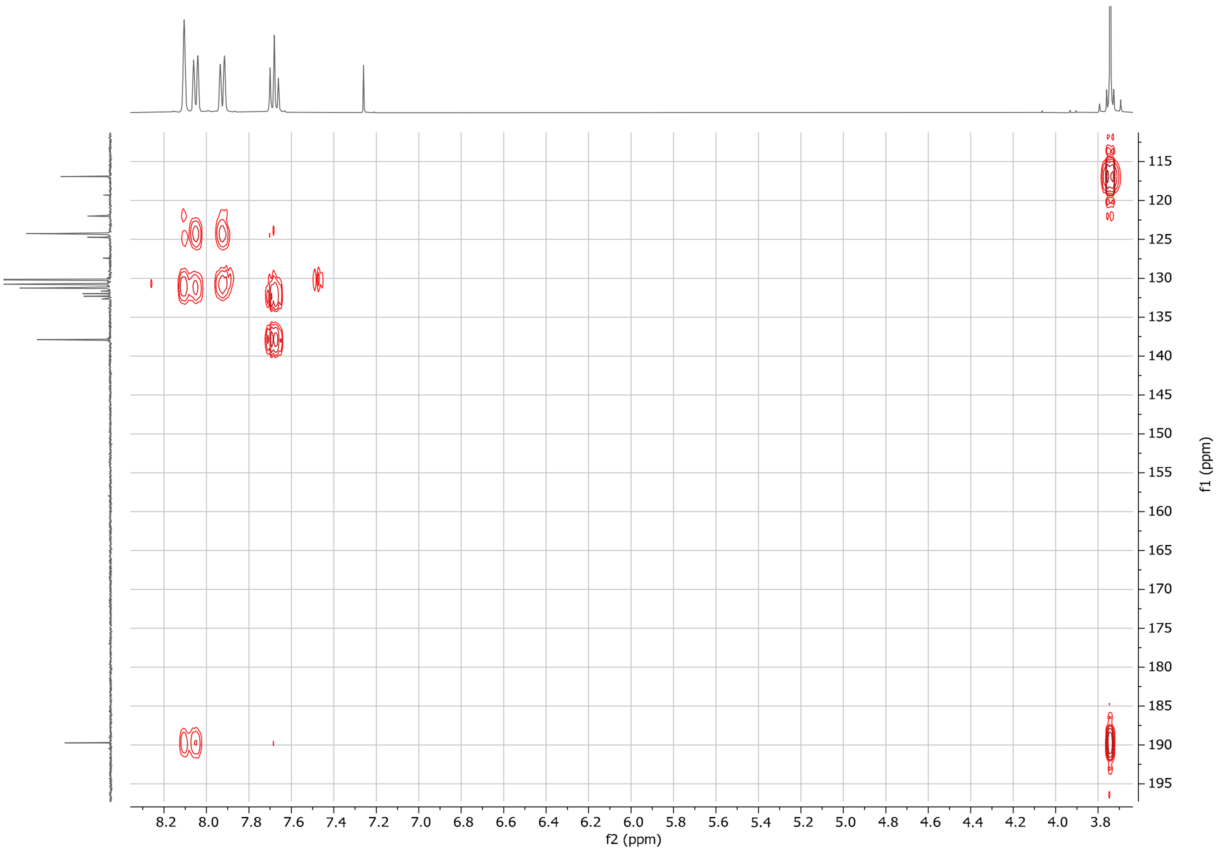

Figure S13I. ^{1}H-^{13}C HMBC NMR spectrum ($CDCl_3$) of **N5**.

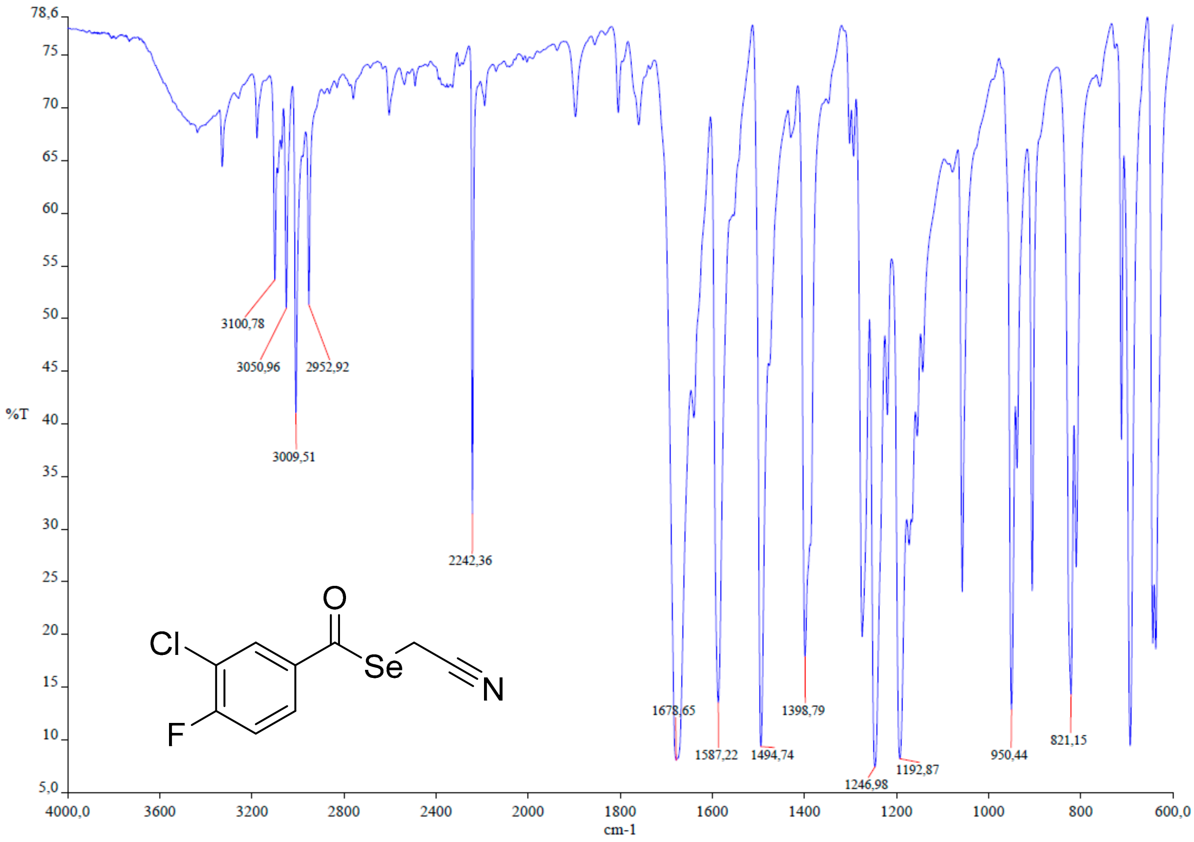

Figure S14. Compound **N6**: Se-(cyanomethyl) 3-chloro-4-fluorobenzoselenoate. S14**A**. IR spectrum (KBr) of **N6**.

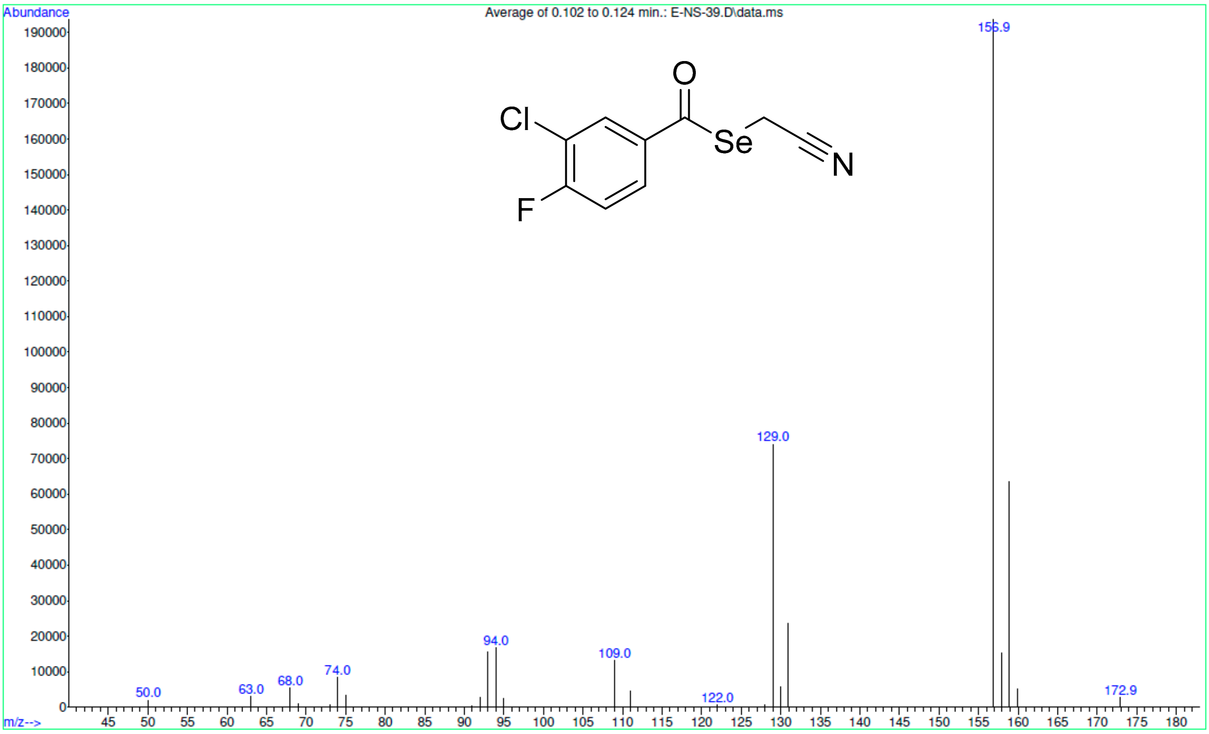

Figure S14B. DIP-MS spectrum of **N6**.

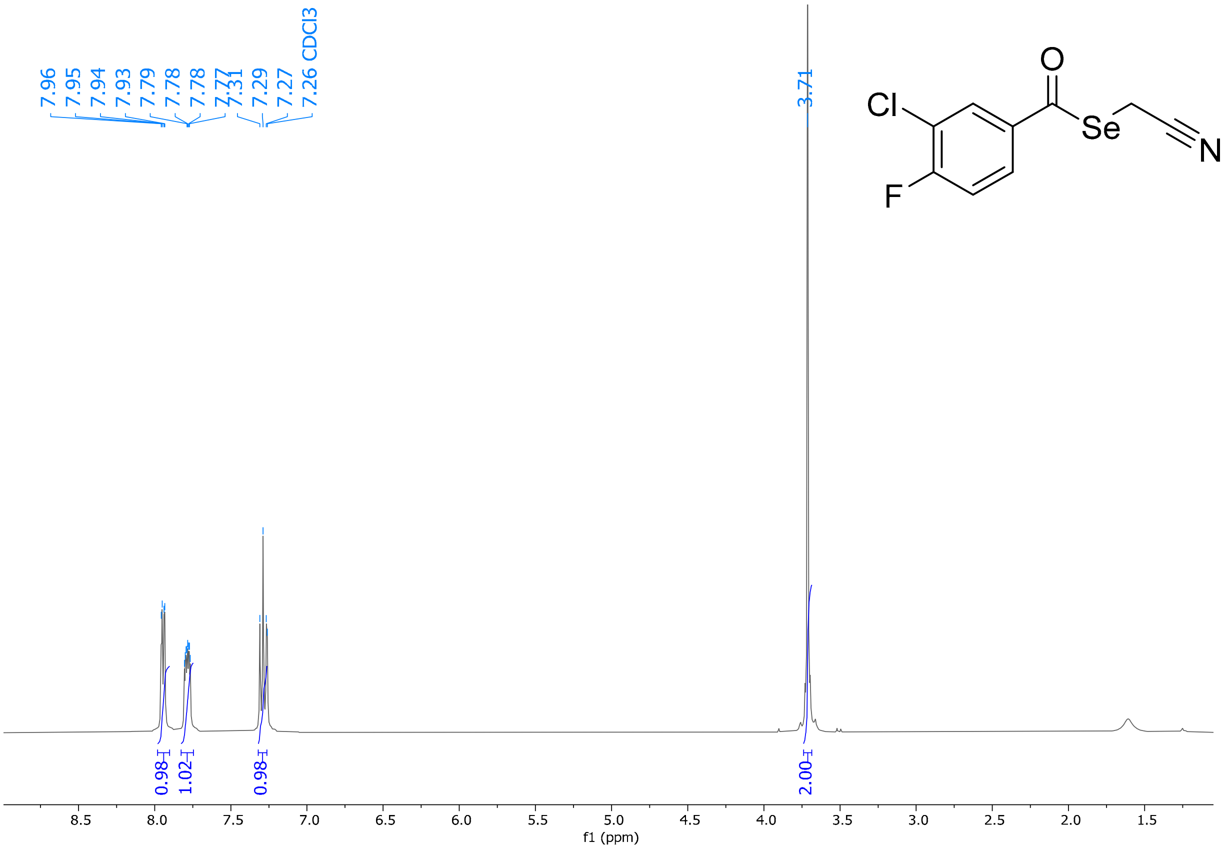

Figure S14C. ^{1}H-NMR spectrum ($CDCl_3$, 400 MHz) of **N6**.

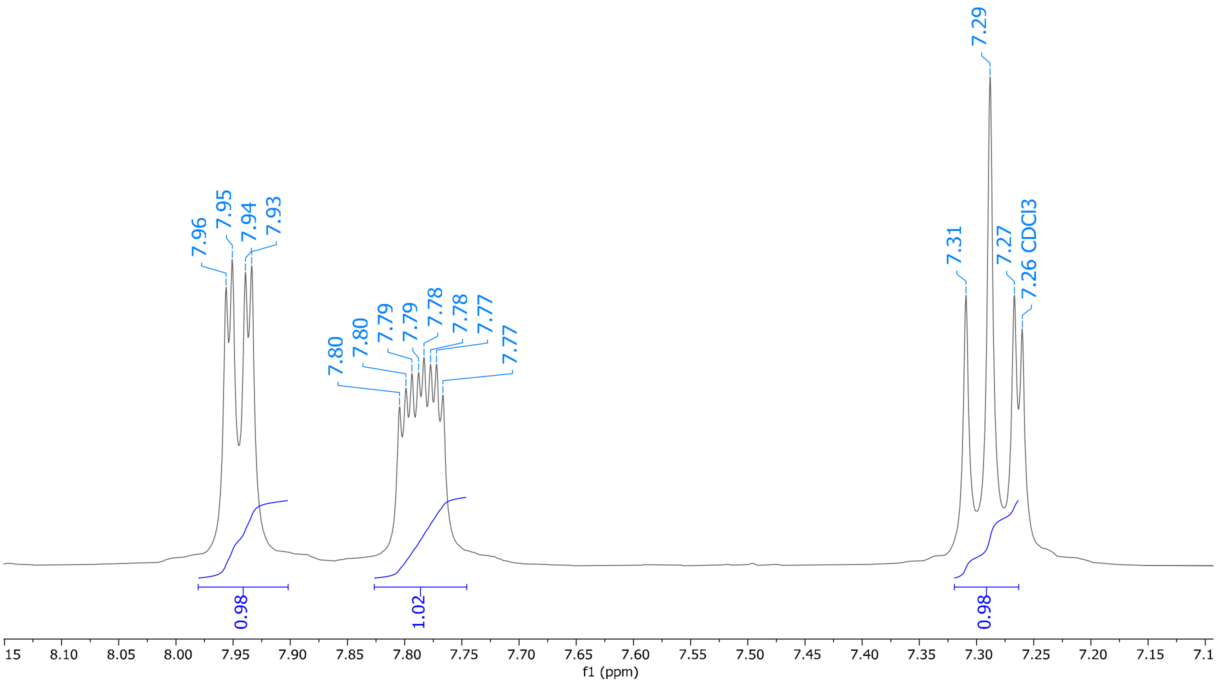

Figure S14D. ^{1}H-NMR spectrum ($CDCl_3$, 400 MHz) of **N6** (aromatics).

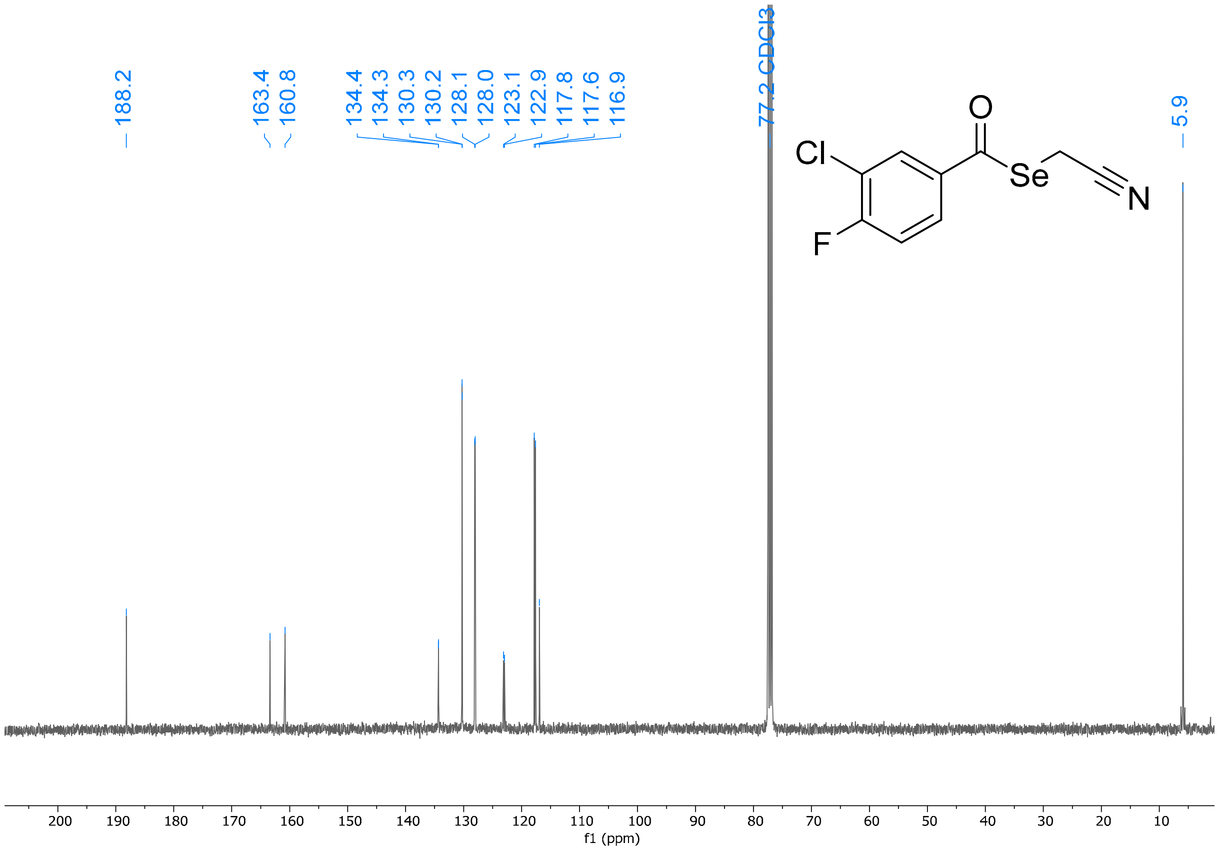

Figure S14E. ^{13}C-NMR spectrum ($CDCl_3$, 101 MHz) of **N6**.

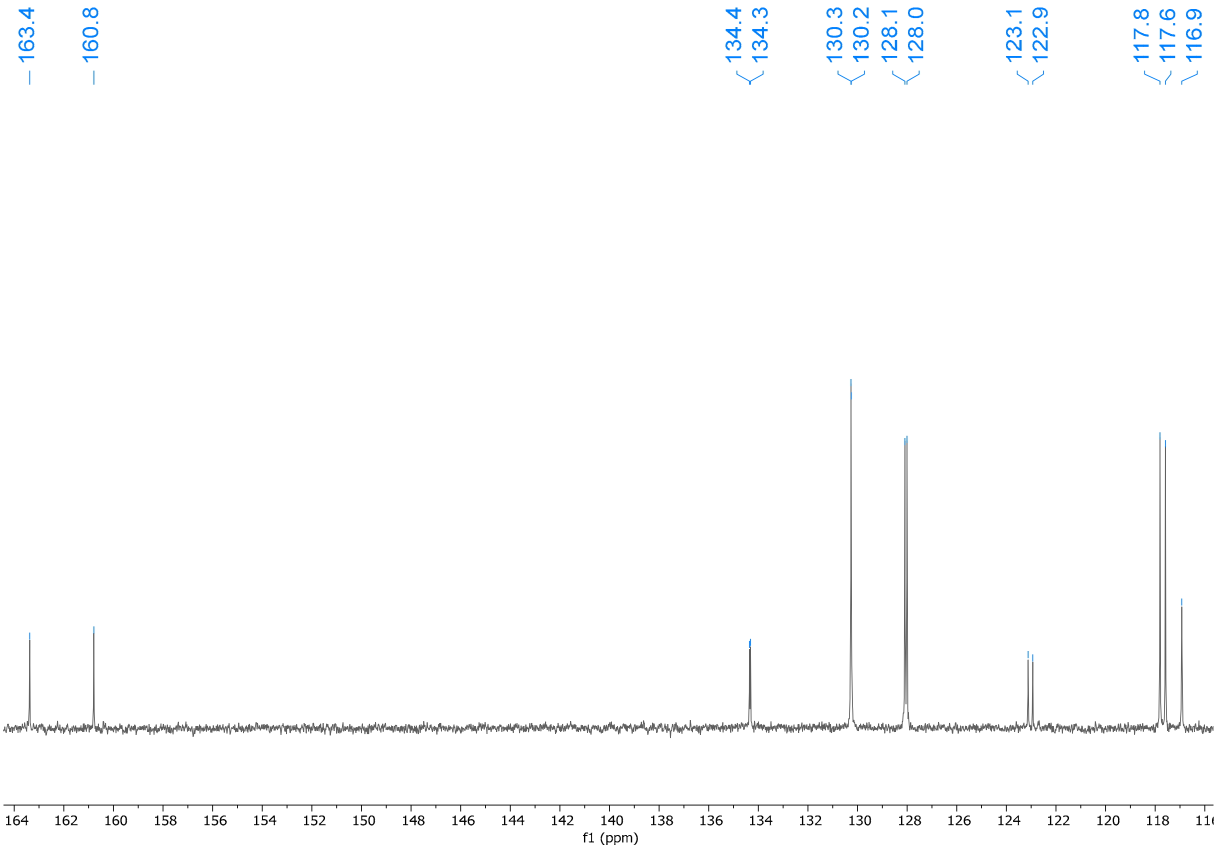

Figure S14F. ^{13}C-NMR spectrum ($CDCl_3$, 101 MHz) of **N6** (aromatics, CN).

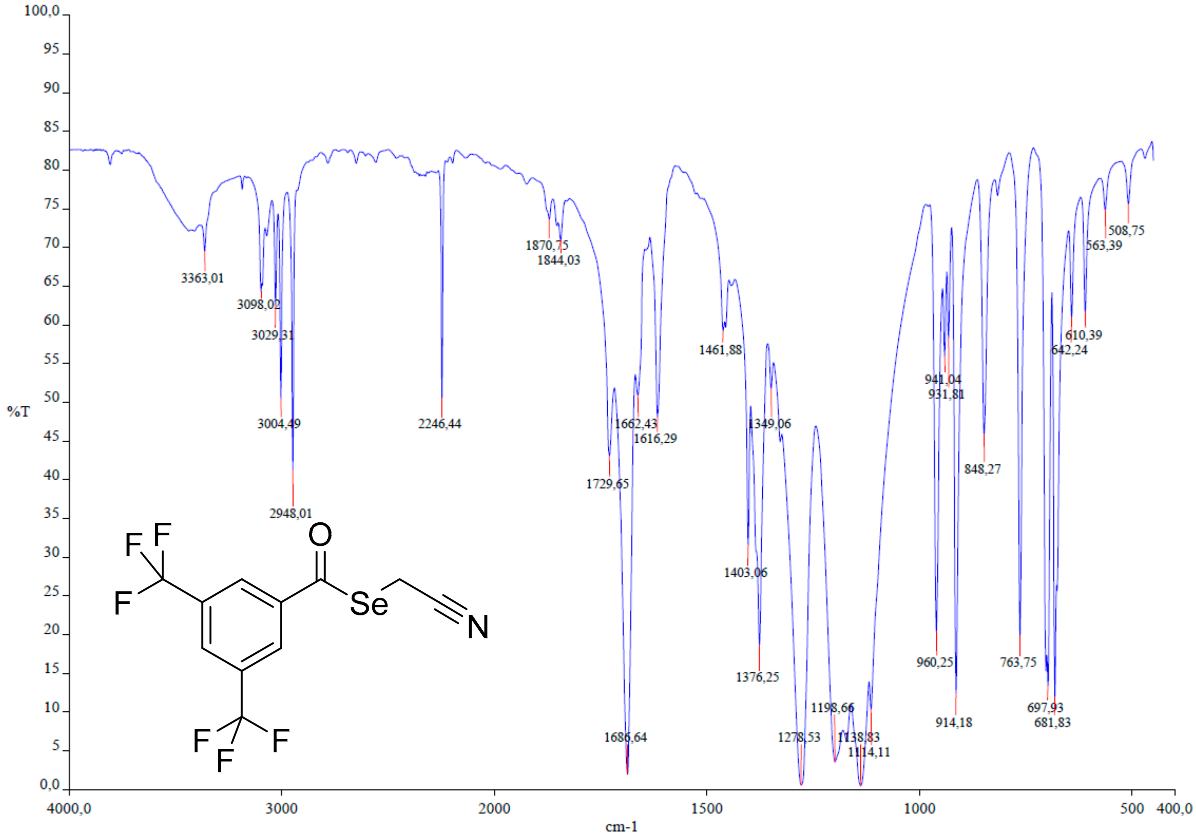

Figure S15. Compound **N7**: Se-(cyanomethyl) 3,5-bis(trifluoromethyl)benzoselenoate. S15**A**. IR spectrum (KBr) of **N7**.

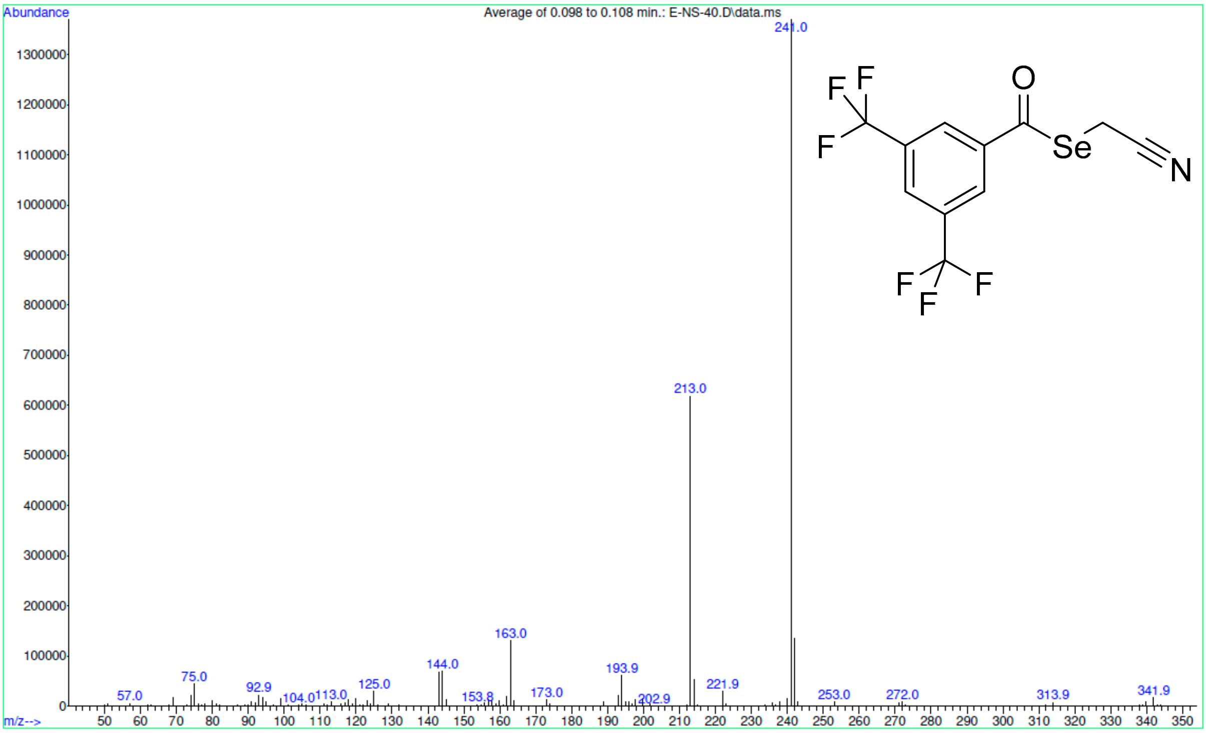

Figure S15B. DIP-MS spectrum of **N7**.

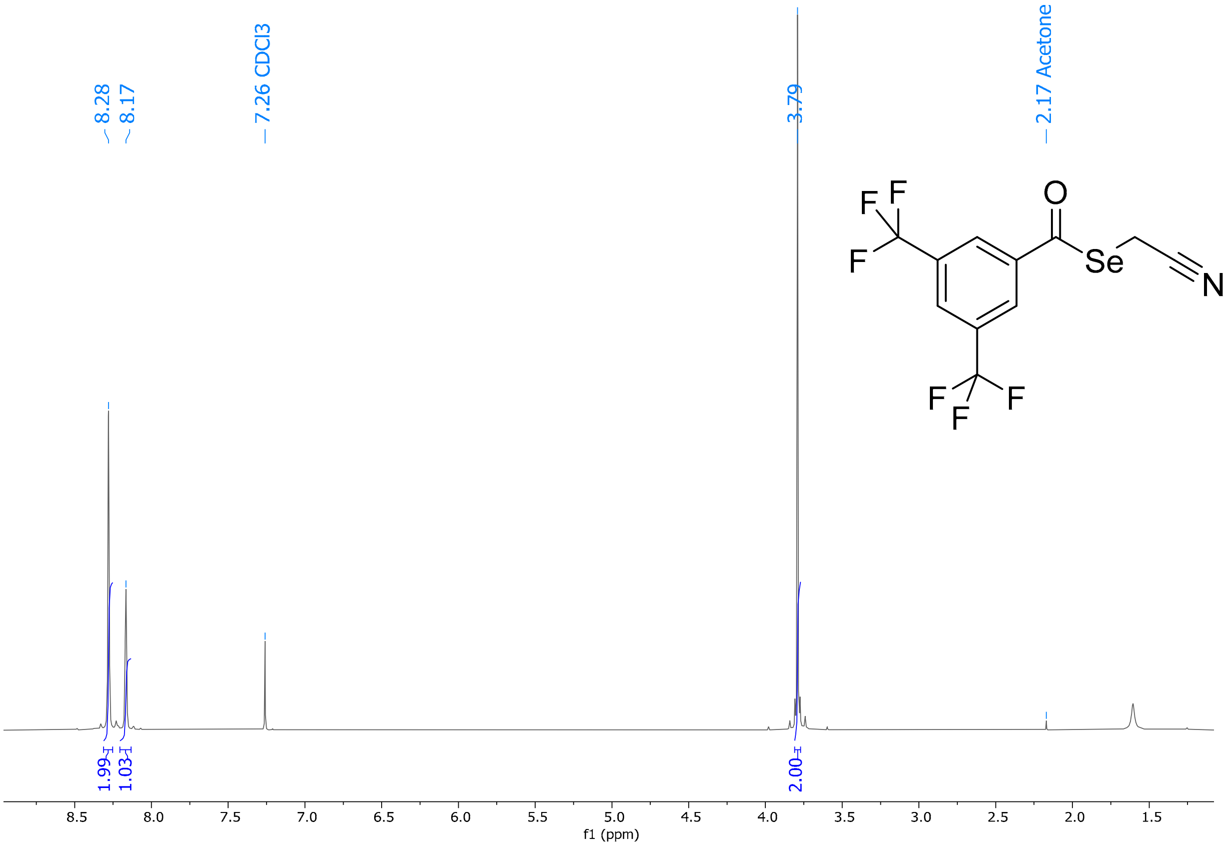

Figure S15C. ^{1}H-NMR spectrum ($CDCl_3$, 400 MHz) of **N7**.

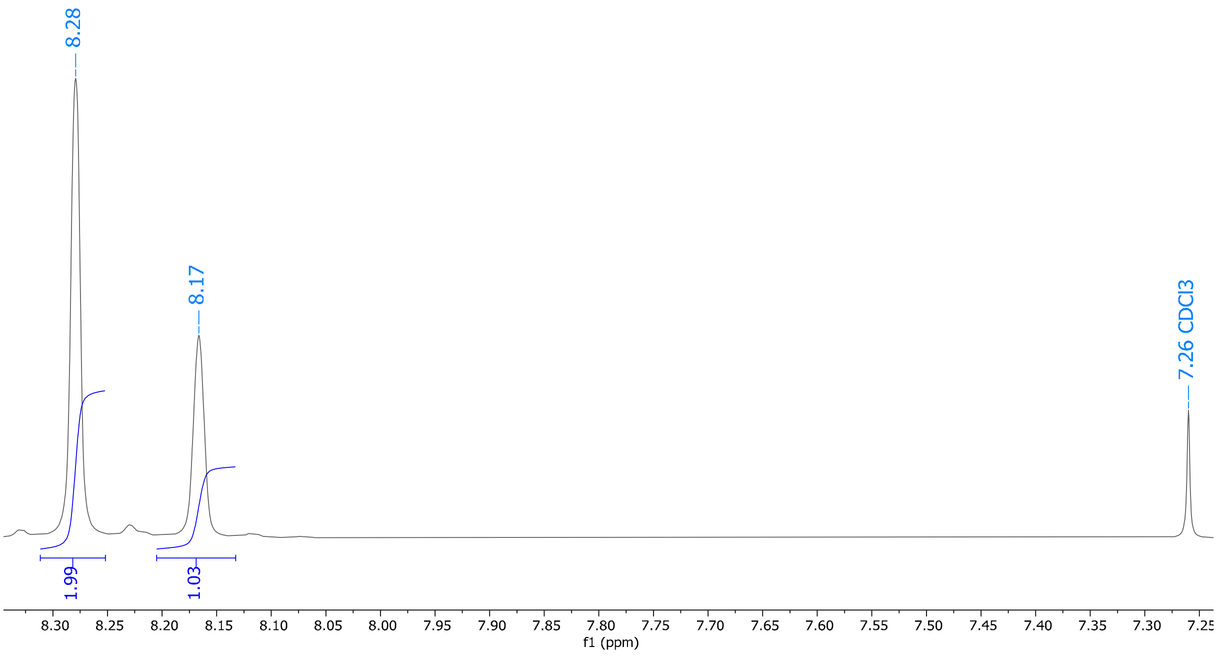

Figure S15D. ^{1}H-NMR spectrum ($CDCl_3$, 400 MHz) of **N7** (aromatics).

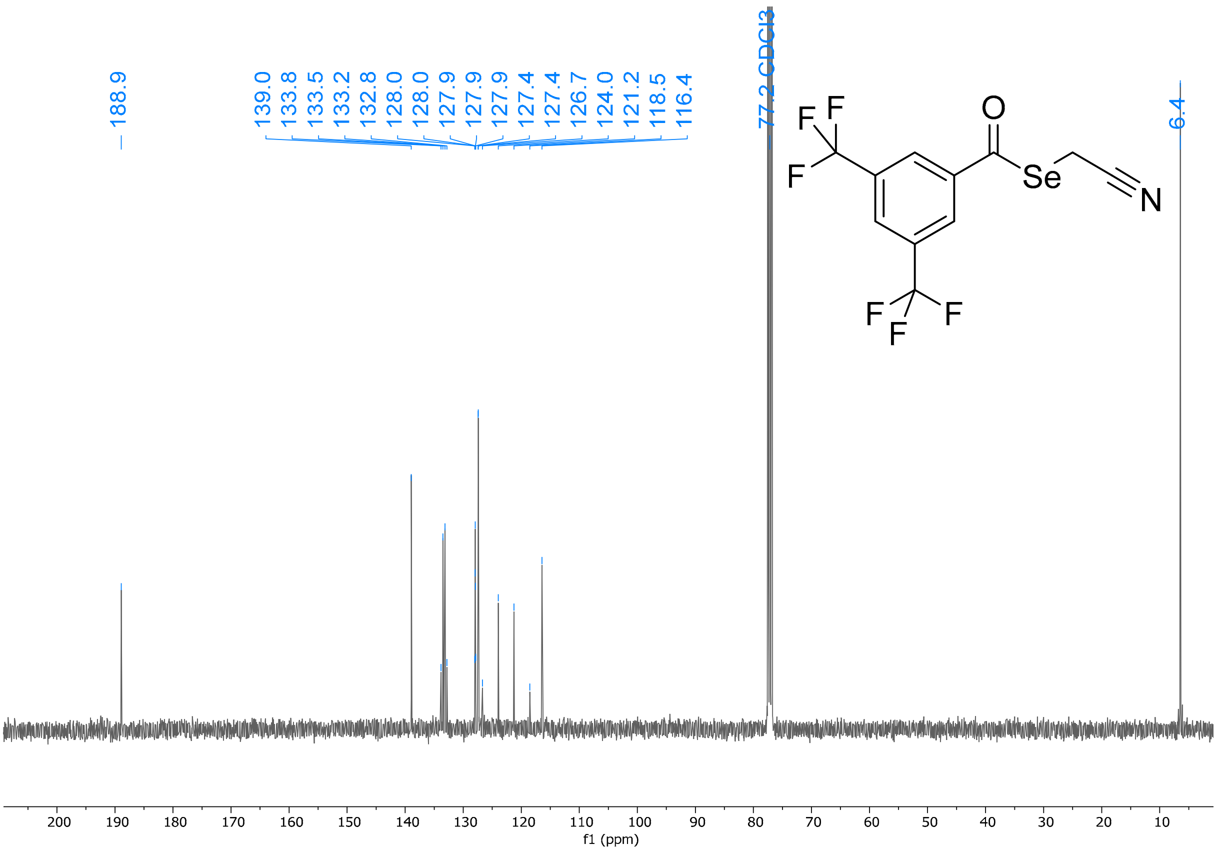

Figure S15E. ^{13}C-NMR spectrum ($CDCl_3$, 101 MHz) of **N7**.

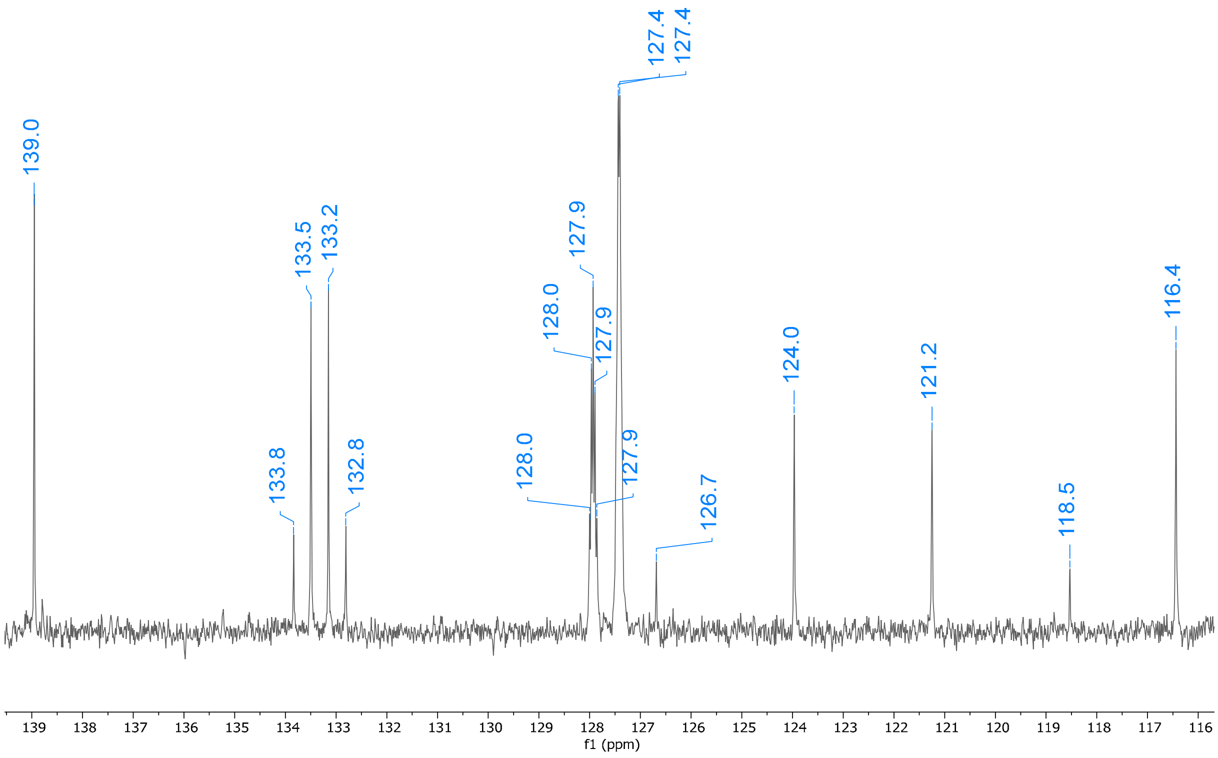

Figure S15F. ^{13}C-NMR spectrum ($CDCl_3$, 101 MHz) of **N7** (aromatics, CN).
